# Total synthesis of a key series of vinblastines modified at C4 that define the importance and surprising trends in activity†
†Electronic supplementary information (ESI) available: Full experimental details and copies of ^1^H and ^13^C NMR spectra are provided. CCDC 1475225–1475228. For ESI and crystallographic data in CIF or other electronic format see DOI: 10.1039/c6sc04146a


**DOI:** 10.1039/c6sc04146a

**Published:** 2016-11-03

**Authors:** Shouliang Yang, Kuppusamy Sankar, Colin K. Skepper, Timothy J. Barker, John C. Lukesh III, Daniel M. Brody, Manuela M. Brütsch, Dale L. Boger

**Affiliations:** a Department of Chemistry , The Skaggs Institute for Chemical Biology , The Scripps Research Institute , 10550 N. Torrey Pines Road , La Jolla , California 92037 , USA . Email: boger@scripps.edu

## Abstract

The total synthesis and evaluation of a key systematic series of vinblastines that incorporate the first deep-seated changes to the substituent at C4 are detailed. The synthetic approach features an expanded and redefined scope of a 1,3,4-oxadiazole [4 + 2]/[3 + 2] cycloaddition cascade in which electronically mismatched electron-deficient trisubstituted alkenes and unactivated trisubstituted alkenes were found to productively initiate the cycloaddition cascade with tethered electron-deficient 1,3,4-oxadiazoles. Such cycloaddition cascades were used to directly introduce altered C4 substituents, providing the basis for concise total syntheses of a series of C4 modified vindolines and their subsequent single-step incorporation into the corresponding synthetic vinblastines in routes as short as 8–12 steps. Evaluation of the synthetic vinblastines revealed a surprisingly large impact and role of the C4 substituent on activity even though it was previously not thought to intimately interact with the biological target tubulin. Only the introduction of a C4 methyl ester, a constitutional isomer of vinblastine in which the carbonyl carbon and ester oxygen of the C4 acetate are transposed, provided a synthetic vinblastine that matched the potency of the natural product. In contrast, even introduction of a C4 acetamide or *N*-methyl carboxamide, which incorporate single heavy atom exchanges (amide NH for ester oxygen) in vinblastine or the C4 methyl ester, provided compounds that were ≥10-fold less active than vinblastine. Other C4 acetate replacements, including a C4 amine, carboxylic acid, hydroxymethyl or acetoxymethyl group, led to even greater reductions in potency. Even replacement of the C4 acetoxy group or its equally active C4 methyl ester with an ethyl or isopropyl ester led to 10-fold or more reductions in activity. These remarkable trends in activity, which correlate with relative tubulin binding affinities, retrospectively may be ascribed to the role the substituent serves as a H-bond acceptor for α-tubulin Lys336 and Asn329 side chains at a site less tolerant of a H-bond donor, placing the methyl group of the C4 acetate or C4 methyl ester in a spatially restricted and well-defined hydrophobic half pocket created by a surrounding well-ordered loop. This remarkable impact of the C4 substituent, its stringency, and even the magnitude of its effect are extraordinary, and indicate that its presence was selected in Nature to enhance the effects of vinblastine and related natural products.

## Abstract 2

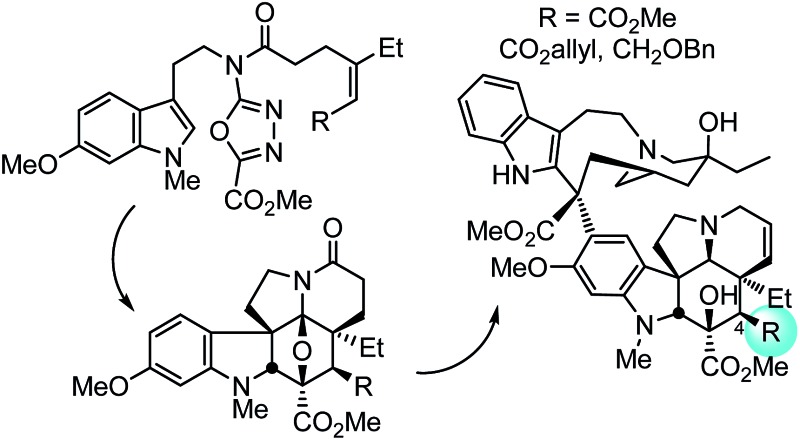
An expanded scope of a powerful oxadiazole cycloaddition cascade was used for the total synthesis of 17 synthetic vinblastines systematically modified at C4. Their evaluation defined a surprisingly significant impact and provided an unrecognized role of the C4 substituent on activity.

## Introduction

**Fig. 1:**
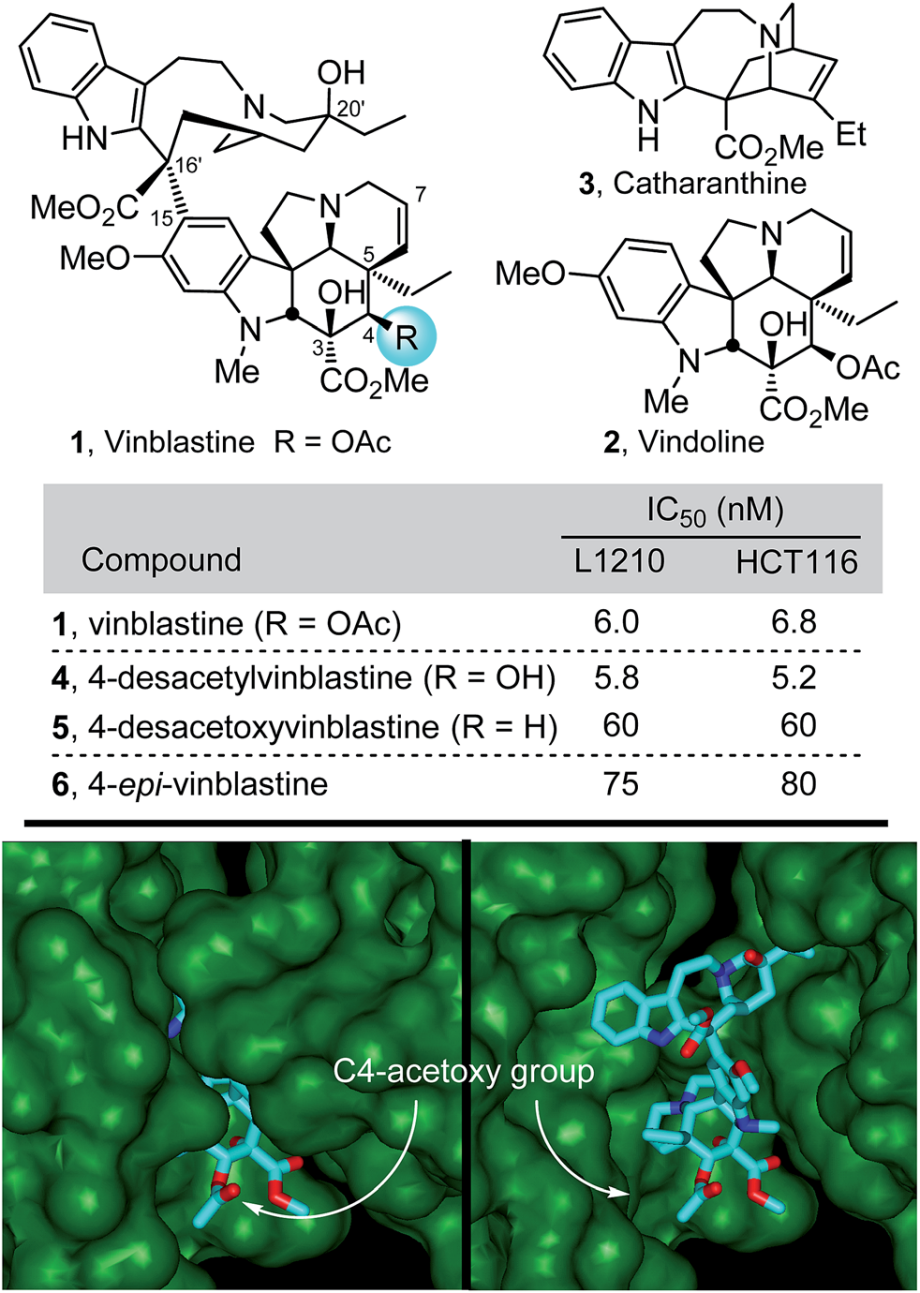
Top: Natural product structures and cell growth inhibition data. Bottom: X-ray co-crystal structure of tubulin-bound vinblastine^25*a*^ (pdb ; 1Z2B) highlighting the solvent exposed C4 acetoxy group at the tubulin head-to-tail dimer–dimer interface where vinblastine binds (left) and site of binding with top of proteins removed to visualize bound vinblastine (right).

The most widely recognized members of the Vinca alkaloids are the antitumor drugs vinblastine (**1**) and vincristine, originally isolated from *Catharanthus roseus* (L.) G. Don^1,2^ (Fig. 1). Because of this intrinsic importance, their complex structures, and their role in the discovery of an important antineoplastic mechanism of action,^3^ they have attracted extensive synthetic and mechanistic efforts since their discovery.^4–7^ In our own efforts first targeting the natural products, we reported the discovery of a powerful intramolecular [4 + 2]/[3 + 2] cycloaddition cascade of 1,3,4-oxadiazoles^8,9^ inspired by the structure of vindoline, the lower subunit of vinblastine. A concise total synthesis of (–)- and *ent*-(+)-vindoline^10^ was developed in which this reaction cascade was used to assemble the full pentacyclic skeleton of **2** with incorporation of all necessary functionality and stereochemistry in a single key step. The extension of this methodology to the preparation of a series of related natural products,^10–15^ its applications in the total syntheses of additional alkaloid natural products,^16–19^ and its use in the subsequent development of an asymmetric total synthesis^20^ of vindoline followed shortly thereafter. This work along with the use of a biomimetic Fe(iii)-promoted coupling of vindoline with catharanthine^21,22^ and the development of a subsequent *in situ* Fe(iii)/NaBH_4_-mediated free radical alkene oxidation for C20′-alcohol introduction^22–24^ allowed for the single-step incorporation of vindoline and its analogues into total syntheses of vinblastine, related natural products including vincristine, and key analogues in routes as short as 8–12 steps. In these latter efforts, we found that subtle modifications at C4 of vinblastine led to surprisingly significant changes in biological activity (Fig. 1).^15,22^ The origin of these substantial effects was not clear and must be subtle since substituents at this site were not thought to intimately interact with the biological target tubulin, rather they are believed to form an interface with solvent in the vinblastine bound complex (Fig. 1).^25^ As a result, we initiated efforts to more clearly define the impact of the C4 substituent by examination of a series of systematic deep-seated modifications at this site complimentary to early semi-synthetic modifications that examined alternative acyl groups (*vs.* Ac).^5^ Because hydrolysis of the C4 acetate represents the major *in vivo* metabolic reaction of the clinical drugs and since the corresponding C4 alcohol is often used as a productive but labile functionalization or bioconjugation site,^4c,5^ the results of these studies were anticipated to be of special interest.

## Results and discussion

**Fig. 2:**
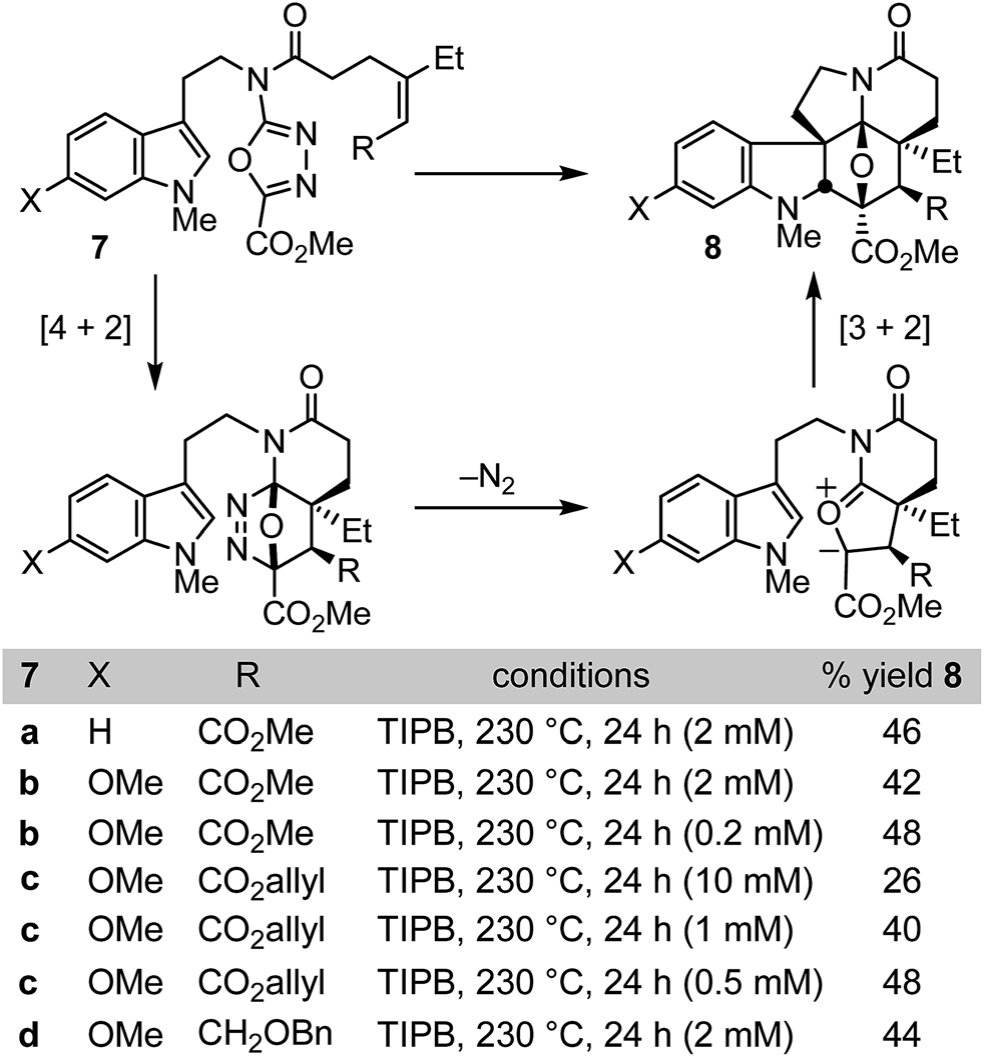
Cycloaddition cascade.

Like vinblastine itself, the targeted modifications were anticipated to be available by synthesis of vindoline analogues bearing the deep-seated C4 modifications through use of the oxadiazole cycloaddition cascade. In initial studies of the intramolecular 1,3,4-oxadiazole cycloaddition cascade, we demonstrated that the initiating inverse electron demand Diels–Alder reaction proceeds with a faster rate and under milder reaction conditions with electronically matched electron-rich dienophiles, and that increasing substitution progressively slows the reaction.^8^ Nonetheless, because of the intramolecular nature of the initiating Diels–Alder reaction, mono and disubstituted unactivated dienophiles as well as mono and disubstituted electron-deficient dienophiles were found capable of initiating the reaction cascade with the electron-deficient oxadiazole, albeit at progressively slower rates. However, two dienophile classes that failed to productively participate in the reaction cascade in our initial survey^8*a*^ were trisubstituted unactivated alkenes and trisubstituted electron-deficient alkenes. Because of our interest in exploring the impact of vinblastine C4 substituents, we reexamined such substrates and herein report conditions under which they may now be employed (Fig. 2).

Both electron-deficient trisubstituted alkenes (**7a–c**) and unactivated trisubstituted alkenes (**7d**) were found to productively initiate the 1,3,4-oxadiazole [4 + 2]/[3 + 2] cycloaddition cascade when the reaction was conducted in triisopropylbenezene (TIPB, 230 °C, 24 h) under dilute reaction conditions (Fig. 2). The concentration dependence of the reaction, which is highlighted most clearly with **7c**, is more dramatic than observed with most substrates. Little product was observed at lower reaction temperatures or at concentrations much greater than 10 mM (*e.g.* 100 mM) and these combined features are likely the reason their productive participation in the reaction cascade was overlooked in early studies.^8^ In addition, resubjection of the products **8a–d** to the reaction conditions led to complete recovery, indicating they are stable under the reaction conditions, and we observed no byproducts that indicated the final [3 + 2] cycloaddition reactions are reversible under these conditions. A single diastereomer of the reaction product was produced, providing the highly functionalized cycloadducts **8a–d** in yields (44–48%) more than sufficient to explore their conversion to synthetic vindolines modified at C4. Four C–C bonds, three rings, all six stereocenters about the newly formed central six-membered ring including four quaternary centers, all the requisite functionality required for preparation of the targeted compounds including the deep-seated C4 modifications, and the full pentacyclic skeleton found in vindoline are formed in a single transformation, offsetting the modest yields of the cycloaddition cascade.

**Scheme 1:**
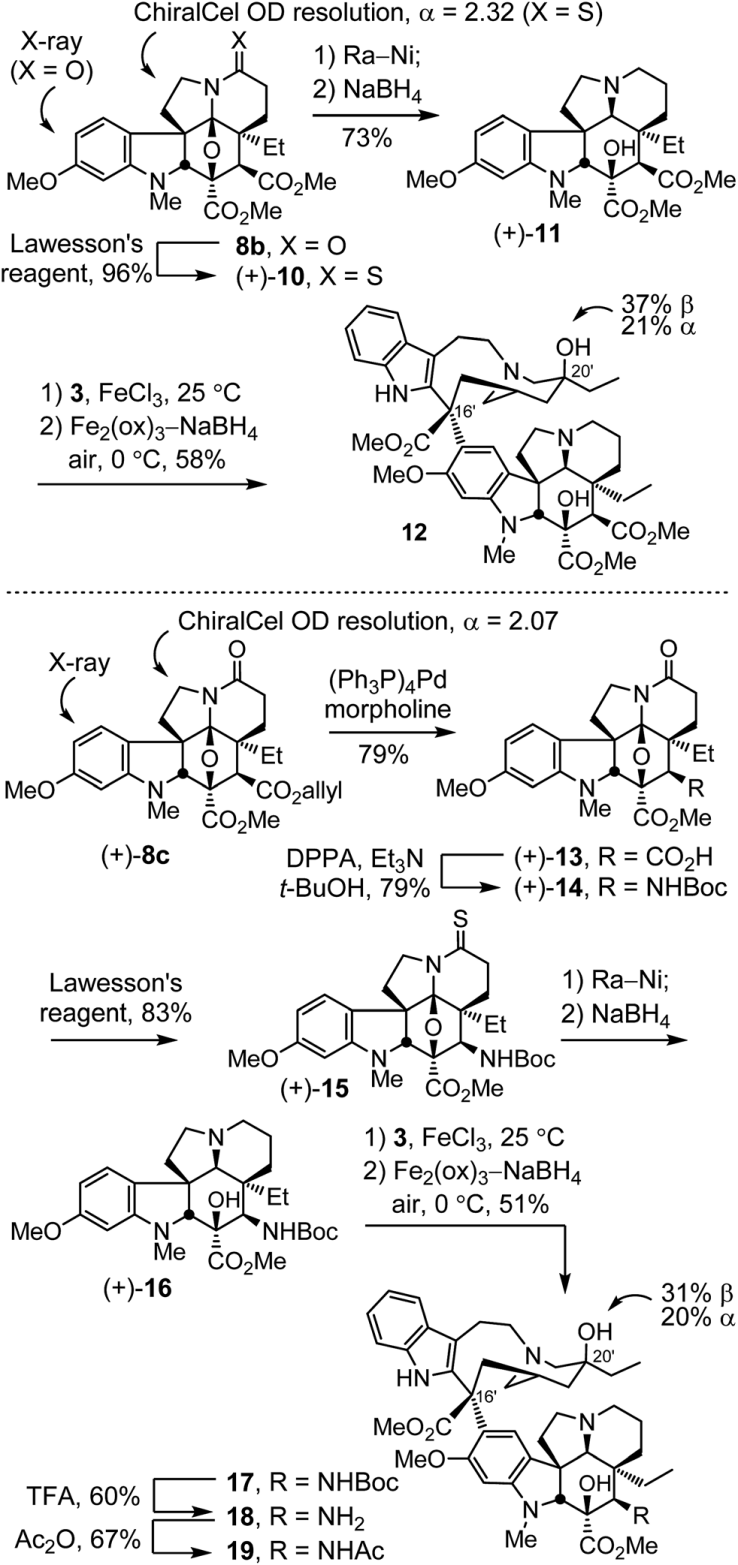


Initial studies conducted with **8b** and **8c**, targeting C4 analogues of 6,7-dihydrovinblastine (**9**),^22*b*^ are summarized in Scheme 1. Although anticipated to be less potent than vinblastine because of the removal of the 6,7-double bond,^24*e*^ these studies were conducted first to establish whether the targeted C4 modifications would prove important to pursue. Treatment of cycloadduct **8b** with Lawesson's reagent (1 equiv., toluene, 110 °C, 1 h) provided the thioamide **10** (96%), which was easily resolved into its enantiomers by chiral phase chromatography (semi-preparative ChiralCel OD, 60% *i*-PrOH/hexane, *α* = 2.32). This chromatographic separation proved both remarkable in its resolution (see ESI†) and scalability for enantiomer separation (>500 mg per injection), making it far preferable to existing^6,20^ or candidate asymmetric synthetic routes.^26^ Removal of the thioamide of (+)-**10** with RANEY® nickel (Ra-Ni, 9 : 1 THF/MeOH, 23 °C) followed by diastereoselective reductive ring opening of the oxido bridge (10 equiv. NaBH_4_, MeOH, 0 °C, 1 h) provided (+)-**11** (73%). Without optimization, single-step Fe(iii)-promoted coupling of (+)-**11** with catharanthine (**3**) proceeded with complete control of the C16′ stereochemistry (5 equiv. FeCl_3_, 10% TFE–0.05 N *aq*. HCl, 25 °C, 2 h), and *in situ* Fe(iii)/NaBH_4_-mediated introduction of the C20′ alcohol *via* free radical hydrogen atom transfer to the intermediate trisubstituted alkene (10 equiv. Fe_2_(ox)_3_, 20 equiv. NaBH_4_, 0 °C, 30 min) in the presence of air (O_2_) proceeded with a 2 : 1 C20′ diastereoselectivity to afford the 6,7-dihydrovinblastine analogue **12**, bearing a C4 methyl ester in place of the acetoxy group. As such, **12** was prepared by total synthesis in only 5 steps from **7b**, and 8 steps from 6-methoxy-1-methyltryptamine.

Through selective ester deprotection, the allyl ester **8c** ^26^ provided the opportunity to introduce additional C4 functionality of which we targeted the acetamide **19** (Scheme 1). This compound incorporates a single heavy atom replacement within the structure with substitution of an amide nitrogen for the acetoxy ester oxygen presumably providing a non-hydrolyzable amide replacement for the metabolically labile C4 acetoxy ester. Resolution of **8c** by chiral phase chromatography proceeded with a similarly remarkable and scalable separation of the enantiomers (semi-preparative ChiralCel OD, 60% *i*-PrOH/hexane, *α* = 2.07).^26^ Allyl ester cleavage of (+)-**8c** (0.1 equiv. (Ph_3_P)_4_Pd, 10 equiv. morpholine, 10 : 1 THF/DMSO, 23 °C, 1 h) cleanly provided the carboxylic acid (+)-**13** (79%). Treatment of (+)-**13** with diphenylphosphoryl azide (2 equiv. DPPA, 2.2 equiv. Et_3_N, *t*-BuOH, 85 °C, 16 h) led to *in situ* formation of the acyl azide and subsequent Curtius rearrangement to give (+)-**14** (79%) with trap of the intermediate isocyanate by *t*-BuOH. Conversion to the thioamide with Lawesson's reagent (1 equiv., toluene, 110 °C, 1 h) provided (+)-**15** (83%). Removal of the thioamide with Ra-Ni (1 : 1 THF/MeOH, 23 °C) followed by diastereoselective reductive ring opening of the oxido bridge (10 equiv. NaBH_4_, MeOH, 0 °C, 1 h) provided (+)-**16**. Without optimization, single-step Fe(iii)-promoted coupling with catharanthine (**3**) and *in situ* Fe(iii)/NaBH_4_-mediated C20′ oxidation provided **17**. Acid-catalyzed Boc removal (TFA–CH_2_Cl_2_ 1 : 4, 23 °C, 2 h) afforded **18** (60%) and amine acetylation (1 : 1 Ac_2_O/pyridine, 23 °C, 30 min; 10 equiv. K_2_CO_3_, MeOH, 23 °C, 1 h) provided the C4 acetamide **19** (67%), incorporating the single heavy atom replacement at C4 of 6,7-dihydrovinblastine (**9**).

**Fig. 3:**
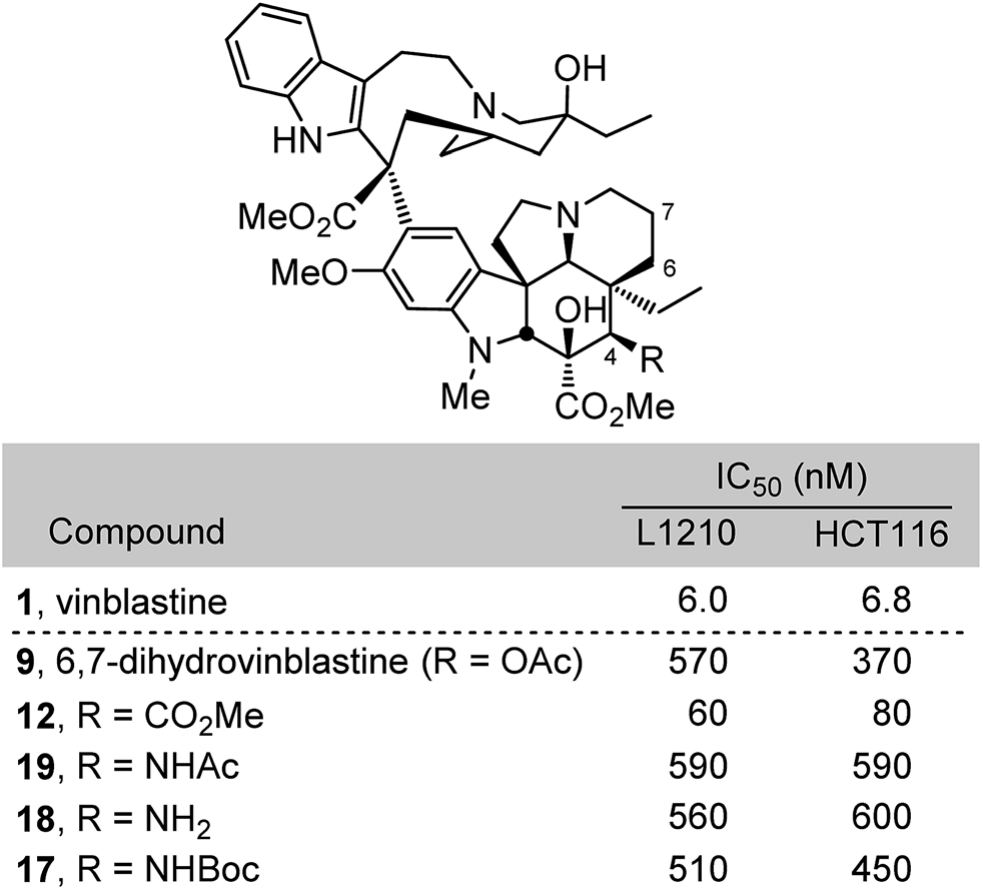
Cell growth inhibition, C4 modifications on 6,7-dihydrovinblastine.

Compounds **12** and **17–19** were assessed alongside 6,7-dihydrovinblastine (**9**) as a direct comparison in cell growth inhibition assays against both mouse leukemia (L1210) and human colon cancer (HCT116) cell lines that have been used to initially examine vinblastine analogues (Fig. 3). Each of the C4 amine derivatives including the acetamide **19** and free amine **18** matched the biological potency of **9** and mirrored the relative activity observed with **1** *vs.* **4** (acetate *vs.* alcohol). Even more significantly, the C4 methyl ester **12** (IC_50_ = 60–80 nM) exceeded the potency of **9** by nearly 10-fold and proved to be only 10-fold (*vs.* 100-fold) less active than vinblastine itself. These results, especially the potent activity of **12**, inspired the following efforts to incorporate these and related C4 modifications into synthetic vinblastines in anticipation that they would be well tolerated, potentially provide additional advantages, and reveal insights into the importance and role of the C4 substituent.

**Scheme 2:**
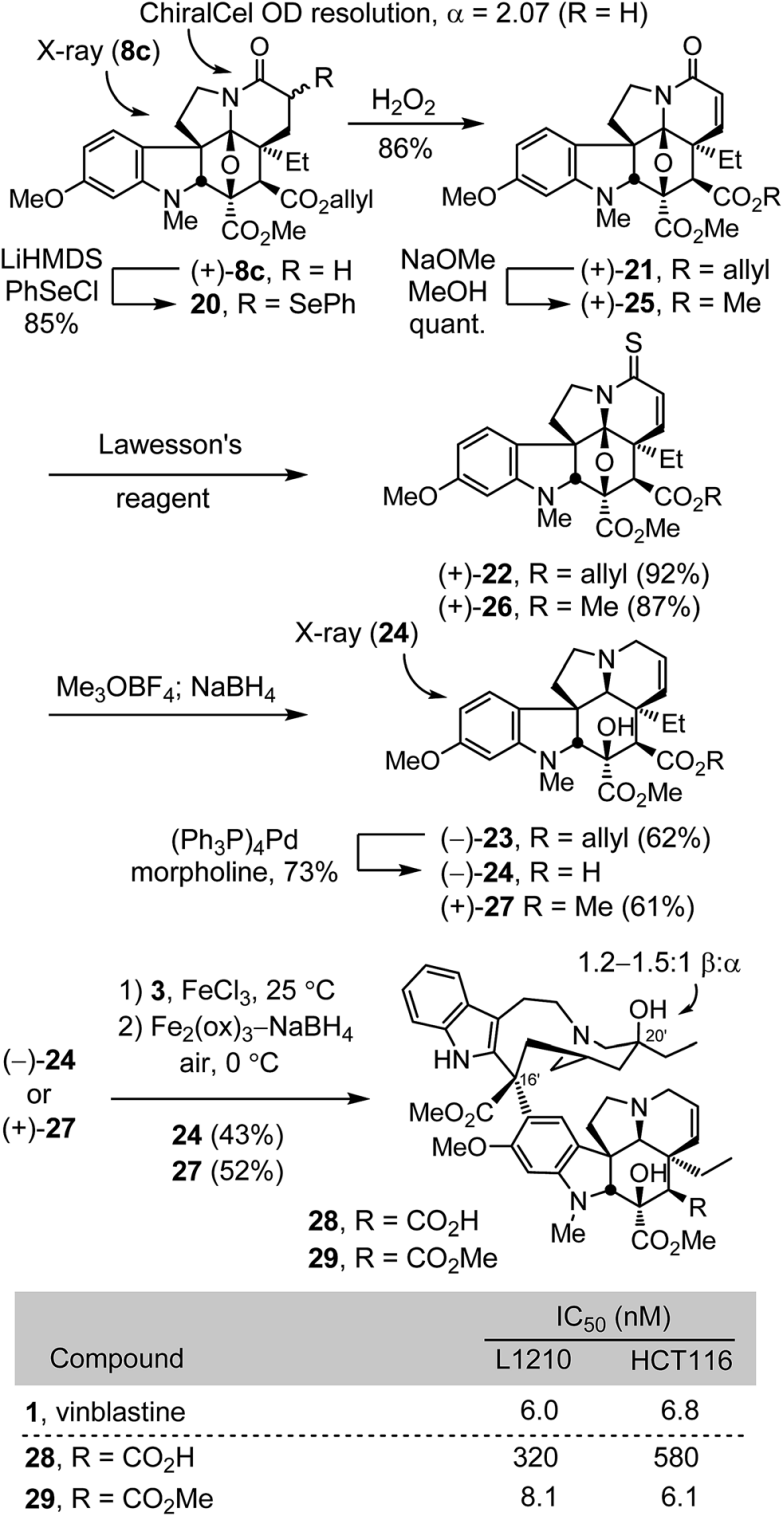


Conversion of (+)-**8c** to a diastereomeric mixture of α-selenides **20** (85%) was accomplished by treatment of the lactam enolate with phenylselenyl chloride (LiHMDS, THF, 1 h, –78 °C) (Scheme 2). The mixture of selenides **20** was treated with H_2_O_2_ (3 equiv.) in THF (0 °C) to provide the α,β-unsaturated lactam (+)-**21** in good yield (86%). Treatment of (+)-**21** with Lawesson's reagent (1.2 equiv., toluene, 100 °C, 1 h) provided thioamide (+)-**22** (92%), which was subjected to methylation with Meerwein's salt (3 equiv., Me_3_OBF_4_, CH_2_Cl_2_, 23 °C, 1 h) followed by NaBH_4_ reduction (3 equiv., MeOH, 0 °C) of the *S*-methyl iminium salt in the same vessel to provide (–)-**23** cleanly (62%). Allyl ester cleavage of (–)-**23** (0.1 equiv. (Ph_3_P)_4_Pd, 10 equiv. morpholine, 10 : 1 THF/DMSO, 23 °C, 1 h) cleanly provided (–)-**24** ^26^ (73%), bearing a C4 carboxylic acid in place of the vindoline C4 acetate. In a complementary fashion, the allyl ester (+)-**21** was converted to the corresponding methyl ester (+)-**25** by transesterification (5 equiv. NaOMe, MeOH, 23 °C, 1 h, quant) and carried through the same sequence of conversion to the thioamide (+)-**26** (1.1 equiv. Lawesson's reagent, toluene, 100 °C, 1 h, 87%), *S*-methylation with Meerwein's salt (3 equiv. Me_3_OBF_4_, CH_2_Cl_2_, 23 °C, 30 min) and NaBH_4_ reduction (9 equiv., MeOH, 0 °C) to provide (+)-**27** (61%), a synthetic vindoline bearing a C4 methyl ester. Without optimization, single-step Fe(iii)-promoted coupling of (–)-**24** and (+)-**27** with catharanthine (**3**) and *in situ* Fe_2_(ox)_3_/NaBH_4_-mediated C20′ oxidation afforded **28** and **29**, synthetic vinblastines containing the C4 carboxylic acid and methyl ester, respectively, each prepared by total synthesis in 7 steps from **7c** or 10 steps from 6-methoxy-1-methyltryptamine. In part, the conciseness of the approach may be attributed to use of an early stage^15^ *versus* penultimate^10^ introduction of the vindoline 6,7-double bond that, in this case, also avoided late stage competitive lactone formation between the C4 ester and a C7 β-alcohol.

**Fig. 4:**
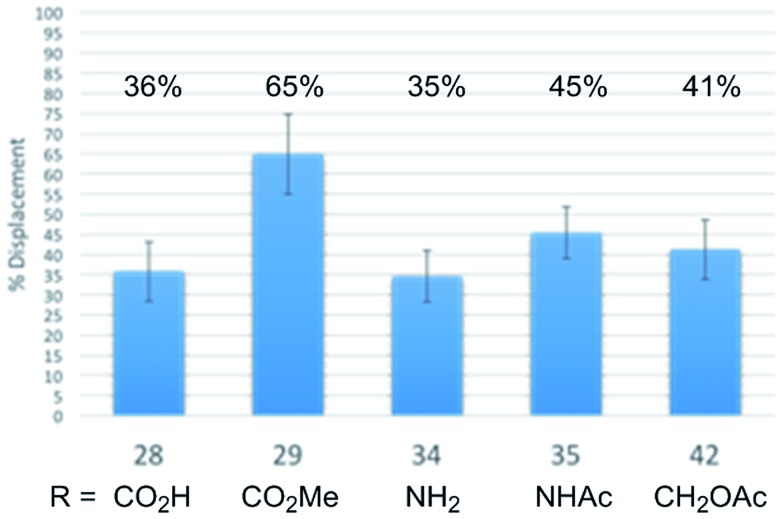
Relative tubulin binding affinities assessed by measuring competitive % displacement of tubulin-bound BODIPY-vinblastine.

The compounds **28** and **29** were assessed in cell growth inhibition assays where the methyl ester **29** (IC_50_ = 6–8 nM) matched the activity of vinblastine, whereas the carboxylic acid **28** was found to be 50–100 fold less potent. Compound **29** is a constitutional isomer of vinblastine in which the ester oxygen and carbonyl of the C4 acetate are simply transposed such that it is now the carbonyl carbon that is directly attached to C4. In addition to this nuanced constitutional isomeric relationship between **1** and **29**, a significant ramification of the change is that the C4 methyl ester, which is flanked by two quaternary centers, is not nearly as susceptible to hydrolysis as the natural C4 acetate. In fact, treatment of **29** with LiOH at room temperature led only to recovered **29**, and reaction was observed only at elevated temperatures with excess base over extended reaction times (excess LiOH, 3 : 2 : 1 THF : MeOH : H_2_O, 50 °C, 17 h), providing preferential and selective C3 (and not C4) methyl ester hydrolysis. Similar room temperature treatment of vinblastine leads to rapid and selective C4 acetate hydrolysis, suggesting **29** is likely to be metabolically much more stable toward C4 hydrolysis than vinblastine. Finally, the reduced activity of the carboxylic acid **28** represents a direct impact the C4 substituent has on tubulin binding affinity (see Fig. 4), although we cannot rule out whether poor cellular uptake also contributes to the diminished activity.

**Scheme 3:**
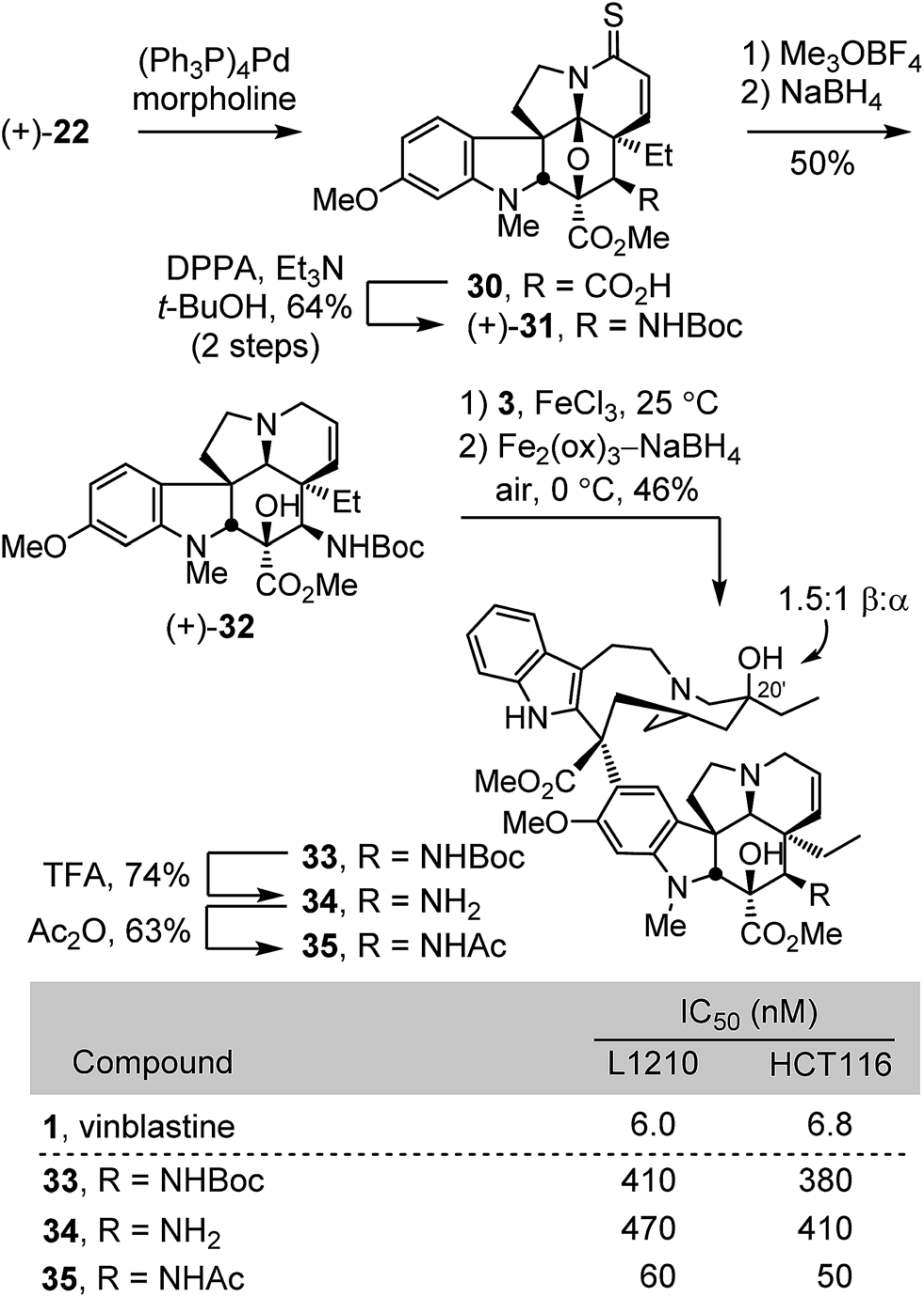


The extension of the studies to the preparation of synthetic vinblastines bearing a functionalized C4 amine, including an acetamide is summarized in Scheme 3. Allyl ester cleavage conducted on the intermediate thioamide (+)-**22** (0.1 equiv. (Ph_3_P)_4_Pd, 10 equiv. morpholine, 10 : 1 THF/DMSO, 23 °C, 1 h) was followed by Curtius rearrangement of the resulting carboxylic acid **30** (2 equiv. DPPA, 3 equiv. Et_3_N, *t*-BuOH, 85 °C, 16 h) to provide (+)-**31** (64% for two steps). *S*-Methylation with Meerwein's salt (3 equiv. Me_3_OBF_4_, 20 equiv. 2,6-di-*t*-butylpyridine, CH_2_Cl_2_, 23 °C, 30 min) followed by NaBH_4_ reduction (9 equiv., MeOH, 0 °C) in the same vessel provided (+)-**32**, a modified vindoline bearing a protected C4 amine. Without optimization, single-step Fe(iii)-promoted coupling of (+)-**32** with catharanthine (**3**) and *in situ* Fe_2_(ox)_3_/NaBH_4_-mediated free radical C20′ oxidation afforded **33**. Acid-catalyzed Boc removal (TFA, CH_2_Cl_2_, 23 °C, 2 h) afforded amine **34** (74%) and acetylation (Ac_2_O, DMAP, CH_2_Cl_2_, 23 °C, 30 min) provided the C4 acetamide **35** (63%). All three compounds **33–35** were assessed in cell growth inhibition assays where both the Boc protected derivative **33** and the free amine **34** were found to be 50 to 75-fold less potent than vinblastine. In contrast, the acetamide **35** exhibited improved activity (IC_50_ = 50–60 nM) relative to the corresponding dihydro compound **19** (*ca.* 10-fold), although it still proved to be roughly 10-fold less potent than vinblastine.

**Scheme 4:**
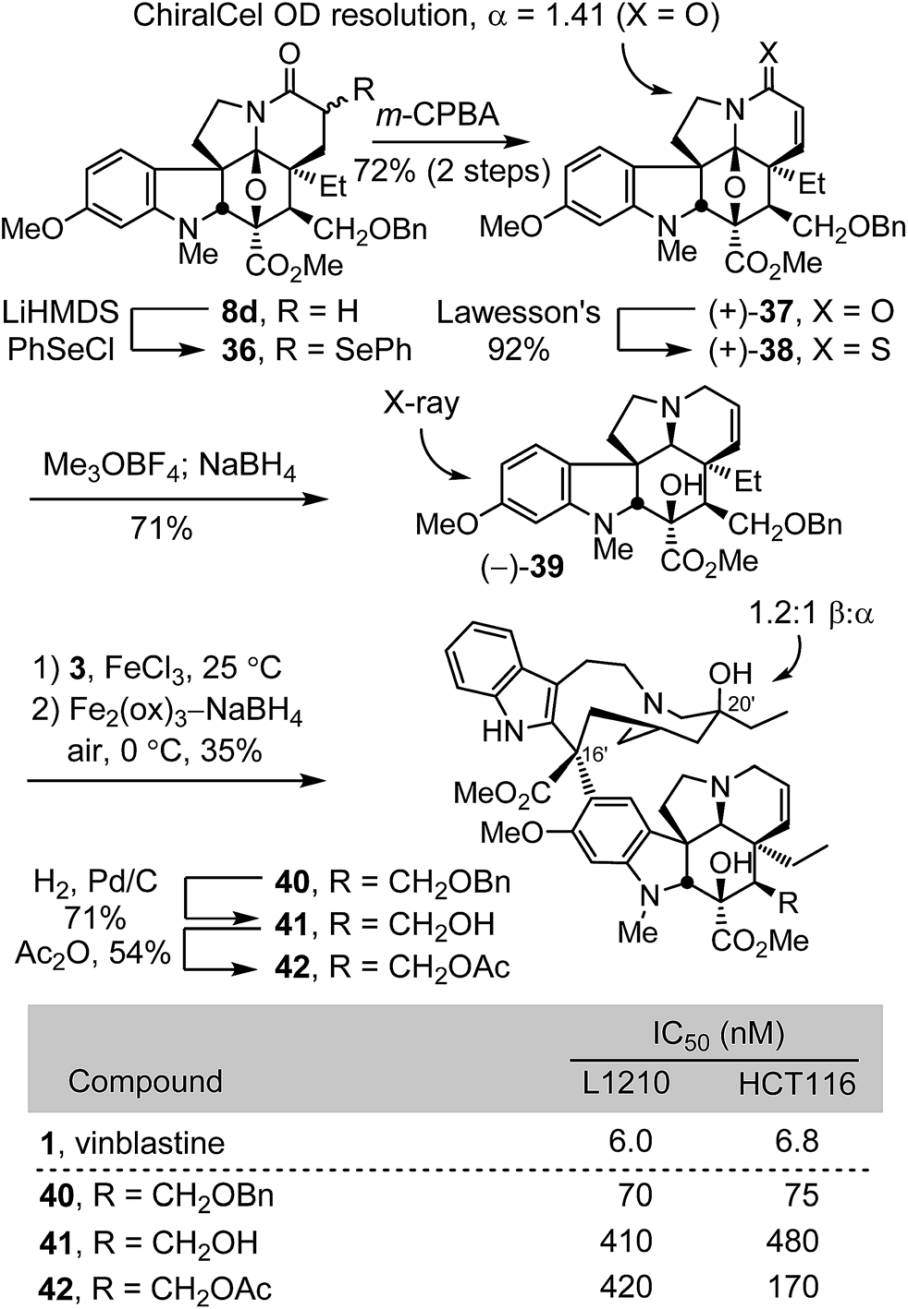


Concurrent with these studies, we prepared compounds that incorporate a C4 hydroxymethyl or acetoxymethyl substituent from the cycloadduct **8d**, introducing an additional carbon between C4 and the polar functional group found in 4-desacetylvinblastine or vinblastine, respectively (Scheme 4). Conversion of **8d** to a diastereomeric mixture of α-selenides **36** (63%) was accomplished by treatment of the lactam enolate with phenylselenyl chloride (2 equiv., 3 equiv. LiHMDS, THF, 1 h, –78 °C). The mixture of selenides **36** was subjected to treatment with *m*-CPBA (1.25 equiv.) in THF (excess pyridine, 0–23 °C, 2 h) to provide the α,β-unsaturated lactam **37** (72%). These latter two reactions were most conveniently conducted without the intermediate purification of the diastereomeric mixture **36**, providing **37** directly in further improved overall yield (72%, 2 steps). Resolution of **37** by chiral phase chromatography provided the two enantiomers (semi-preparative ChiralCel OD, 60% *i*-PrOH/hexane, *α* = 1.41). Treatment of (+)-**37** with Lawesson's reagent (1 equiv., toluene, 80 °C, 30 min) provided thioamide (+)-**38** (92%), which was subjected to methylation with Meerwein's salt (2 equiv. Me_3_OBF_4_, CH_2_Cl_2_, 23 °C, 1 h) followed by *in situ* NaBH_4_ reduction (6 equiv., 1 : 1 MeOH/CF_3_CH_2_OH, 0 °C) of the *S*-methyl iminium ion, provided (–)-**39** (71%). A single crystal X-ray structure determination conducted with the natural enantiomer of **39** confirmed its structure, relative stereochemistry, and absolute configuration.^26^ For comparison purposes and with (–)-**39** in hand, the synthetic vinblastines **40–42** were prepared. Single-step Fe(iii)-promoted coupling of (–)-**39** with catharanthine (**3**), which proceeded with complete control of the newly formed C16′ quaternary stereocenter, and subsequent *in situ* Fe_2_(ox)_3_/NaBH_4_-mediated free radical C20′ oxidation afforded **40** and its C20′ isomer. *O*-Debenzylation (H_2_, Pd/C, 50 : 1 MeOH/TFA, 71%) and subsequent *O*-acetylation of the liberated alcohol **41** (Ac_2_O, DMAP, 23 °C, CH_2_Cl_2_, 54%) provided **42**. The latter two compounds constitute analogues of desacetylvinbastine (**4**) and vinblastine (**1**) containing a carbon inserted at C4 between the natural product core and the polar substituent. Both **41** and **42** proved to be 20- to 70-fold less potent than the corresponding natural products, indicating that the added change significantly reduces activity. Interestingly, the benzyl ether precursor **40** was found to be more potent than either **41** or **42**, but remained *ca.* 10-fold less active than vinblastine and exhibited activity on par with desacetoxyvinblastine (**5**) lacking a C4 substituent.

**Scheme 5:**
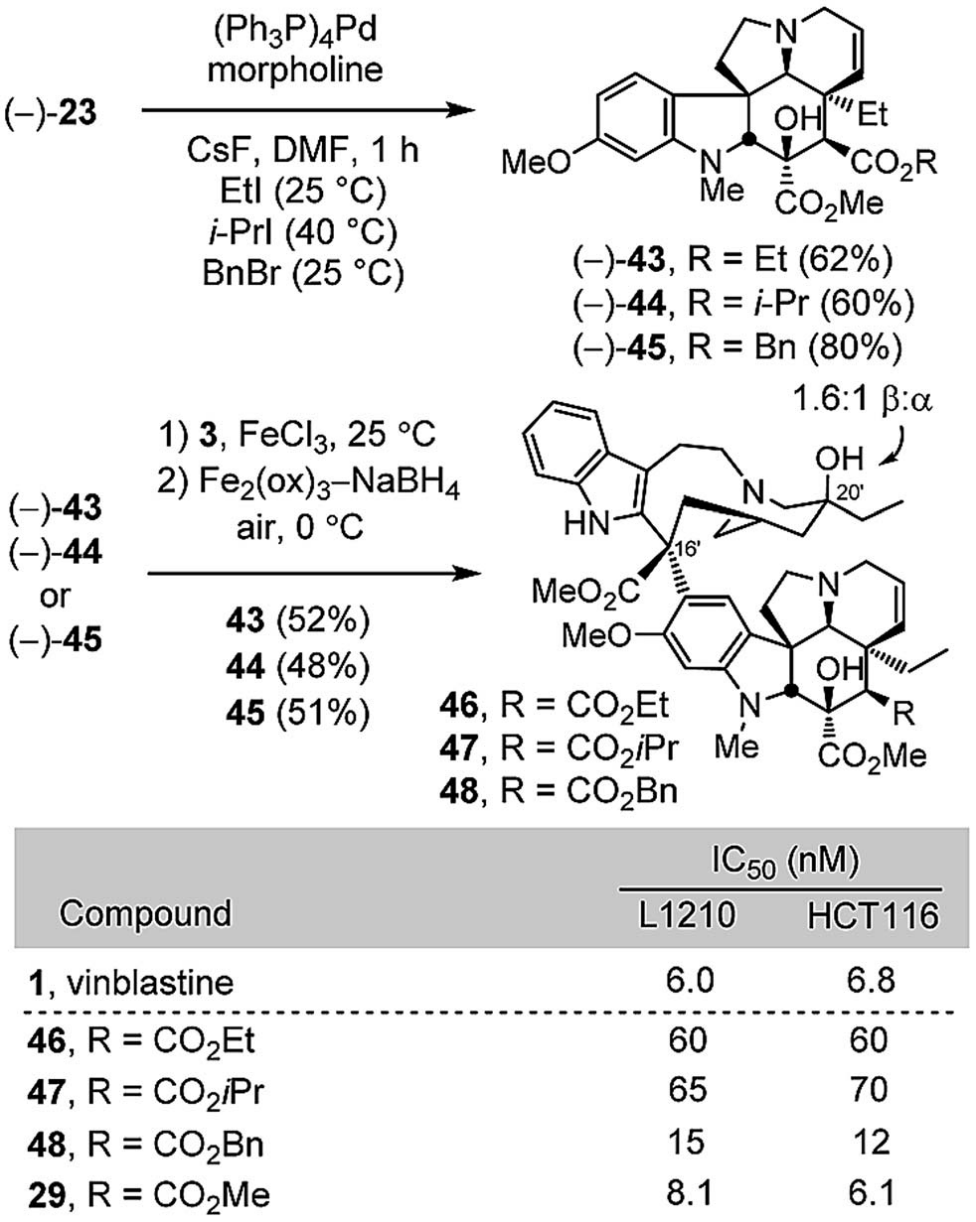


Given the unique behavior of the methyl ester **29**, being the only C4 modified compound that matched the activity of vinblastine, three additional esters were examined to define its sensitivity to modification. Thus, alkylative esterification of carboxylic acid **24**, derived from deprotection of the allyl ester (–)-**23**, with ethyl iodide (2 equiv. CsF, DMF, 25 °C, 1 h, 62%), isopropyl iodide (2 equiv. CsF, DMF, 40 °C, 1 h, 60%), or benzyl bromide (2 equiv. CsF, DMF, 25 °C, 1 h, 80%) provided **43–45** without purification of the intermediate carboxylic acid (Scheme 5). Without optimization, their single-step Fe(iii)-promoted coupling with catharanthine (**3**) and subsequent *in situ* Fe_2_(ox)_3_/NaBH_4_-mediated free radical C20′ oxidation afforded **46–48**. Stunningly, even the apparently benign changes to the ethyl or isopropyl esters **46** and **47** led to 10-fold reductions in cell growth inhibition activity. Only the benzyl ester **48** approached the activity of **29**, desacetylvinblastine (**4**), and vinblastine.

**Scheme 6:**
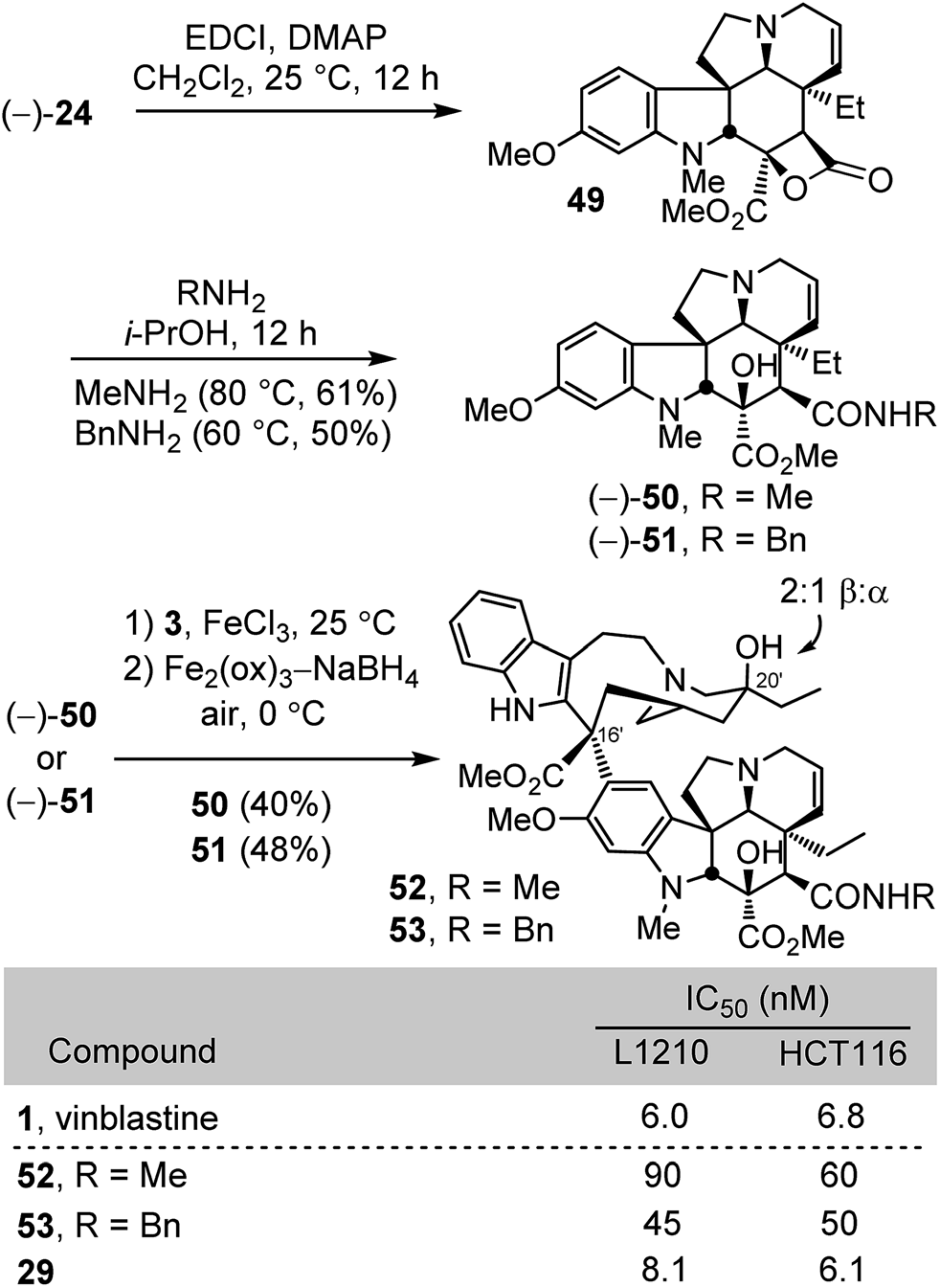


Finally, the C4 *N*-methyl carboxamide **52** and *N*-benzyl carboxamide **53** were prepared for comparison in part to establish whether the substituent polarity may contribute to the functional activity of vinblastine (Scheme 6). The former *N*-methyl carboxamide serves as a direct amide comparison with the methyl ester **29**, replacing the ester oxygen with an amide NH. Their synthesis involved a unique closure of the carboxylic acid **24** first to the characterized but reactive β-lactone **49** ^27^ (2 equiv. DMAP, 2 equiv. EDCI, CH_2_Cl_2_, 25 °C, 12 h), an intermediate preferentially generated upon activation of the carboxylic acid under a range of conditions (EDCI, HATU, or PyBOP), followed by its subsequent reaction with methylamine or benzylamine (*i*-PrOH, 80 and 60 °C respectively, 12 h) to provide **50** (61%) and **51** (50%) in good yields from **23** (3 steps) without intermediate purifications. Without optimization, their single-step Fe(iii)-promoted coupling with catharanthine (**3**) and subsequent *in situ* Fe_2_(ox)_3_/NaBH_4_-mediated free radical C20′ oxidation afforded **52** and **53**. Again and remarkably, the *N*-methyl amide proved to be more than 10-fold less active than vinblastine or the methyl ester **29** and, while more potent, the *N*-benzyl amide was also 5–10-fold less active.

In order to establish whether the substituent effects observed in the cell growth functional assays were derived from on target effects, key members of the initial series of compounds were examined in a competitive tubulin binding assay, measuring their ability to displace tubulin-bound BODIPY-vinblastine (Fig. 4).^24*h*^ Consistent with their functional activity but contrary to expectations based on their perceived placement in a tubulin-bound X-ray (see Fig. 1), these key derivatives displayed easily distinguishable relative tubulin binding affinities that correlated with their relative cell growth inhibition activity (R: CO_2_Me > NHAc > CH_2_OAc > CO_2_H, NH_2_). Thus, the vinblastine C4 substituent significantly impacts tubulin binding affinity and the trends observed correlate with the resulting functional cell growth inhibition. Moreover, the site and functionality are remarkably sensitive to seemingly benign structural modification.

**Fig. 5:**
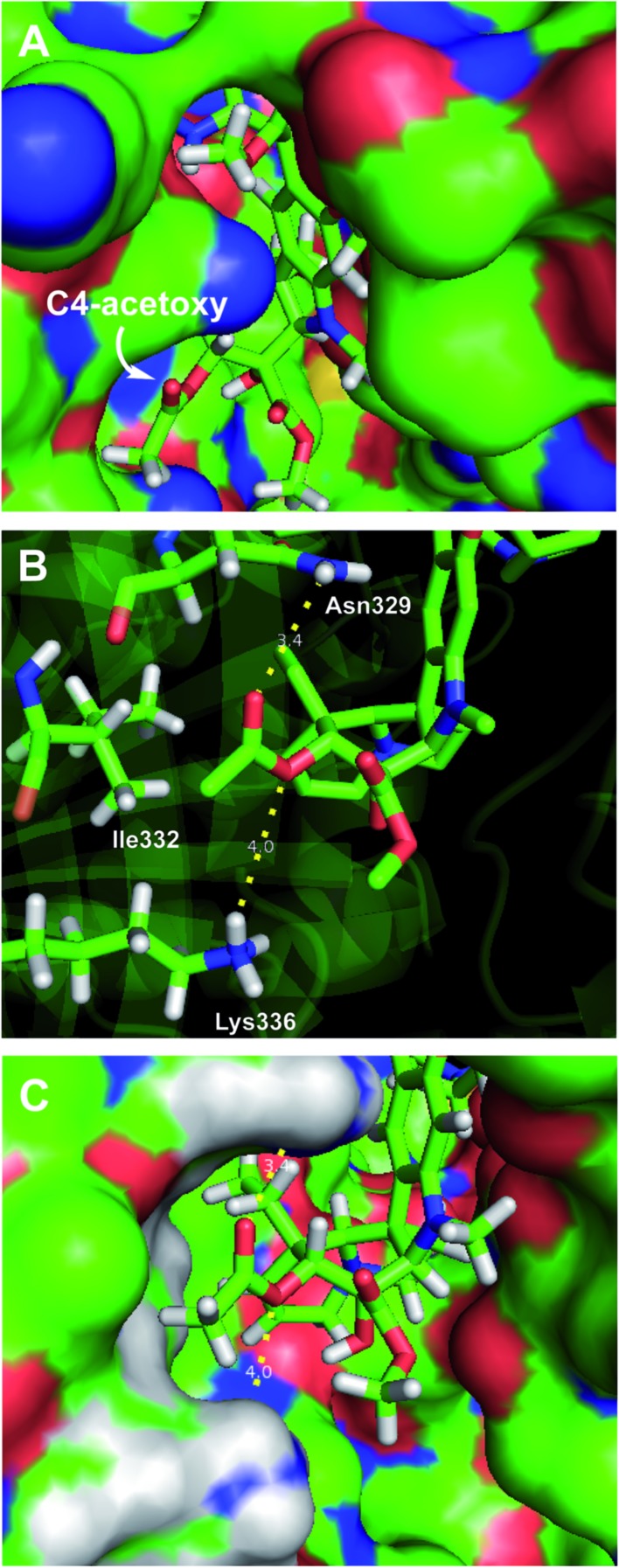
Recent X-ray co-crystal structure of tubulin-bound vinblastine^25*b*^ (pdb ; 5J2T) highlighting the (A) solvent exposed C4 acetoxy group. (B) Key residues interacting with the C4 substituent: Lys336 (H-bond to acetoxy ester oxygen), Ile332 (close contact to methyl group), and Asn329 (H-bond to acetoxy carbonyl oxygen). (C) Space filling model of B showing the fit of the acetoxy methyl group in the hydrophobic pocket defined by Ala333 and Ile332 residues.

Retrospective examination of the original >4 Å tubulin-bound X-ray of vinblastine^25*a*^ was not helpful in defining additional roles for the C4 substituent beyond serving as a polar interface for the bound complex. However, a higher resolution 2.2 Å structure just published^25*b*^ proved much more informative. In it, the C4 acetate serves as a stabilizing H-bond acceptor with the protonated amine side chain of Lys336 (ester O) and Asn329 (carbonyl O) in a now well-ordered lip on the tubulin protein surface that interacts with and wraps around the side of the acetate, placing the acetate methyl group in a spatially well-defined small hydrophobic half pocket adjacent to and lined by Ile332 and Ala333 (Fig. 5). These same H-bond acceptor roles may be functionally satisfied by swapping the protein residues interacting with the carbonyl oxygen (H-bond from Lys336) and ester oxygen (H-bond from Asn329) of the C4 methyl ester **29** within this ordered flexible loop, still placing the methyl group in the same hydrophobic half pocket adjacent to the side chains of Ile332 and Ala333. This would not be possible with C4 substituents that incorporate H-bond donors *versus* acceptors at these sites (C4 acetamide, *N*-methyl carboxamide), with substituents that displace the H-bond acceptors (the homologated C4 hydroxymethyl or acetoxymethyl groups), or with substituents that incorporate larger acyl or ester substituents. Such substituents do not preclude vinblastine binding, just that the well-ordered interaction of the C4 acetate/methyl ester with the protein loop and the resulting stabilizing interactions would be lost. As a result, it is not surprising that the alternative C4 substituents at best behave analogous to desacetoxyvinblastine lacking a C4 substituent altogether (**5** *vs.* **35**, **46**, **47**, **52**, and **53**) or may further destabilize binding (**28**, **33**, **34**, **41**, and **42**). Perhaps still superimposed on this role as a H-bond acceptor for Lys336 and Asn329, the C4 functionality may still serves as part of the polar interface for tubulin bound vinblastine. Additionally, the equipotent activity of the C4 alcohol (**4**), the surprisingly good activity of the benzyl ester **53** and even the activity of the benzyl ether **40** (*vs.* **41** and **42**), which do not conform to this interpretation, suggest there may be additional unrecognized ways in which this flexible lip of tubulin at the solvent interface of the complex may productively interact with selected C4 substituents and stabilize the binding of vinblastine analogues. Finally, it is surprising the C4 carboxylic acid binds so poorly to tubulin given the potential stabilizing electrostatic interaction with tubulin Lys336, perhaps reflecting the impact of necessarily residing alongside the hydrophobic half pocket defined by Ile332 and Ala333.

## Conclusions

**Fig. 6:**
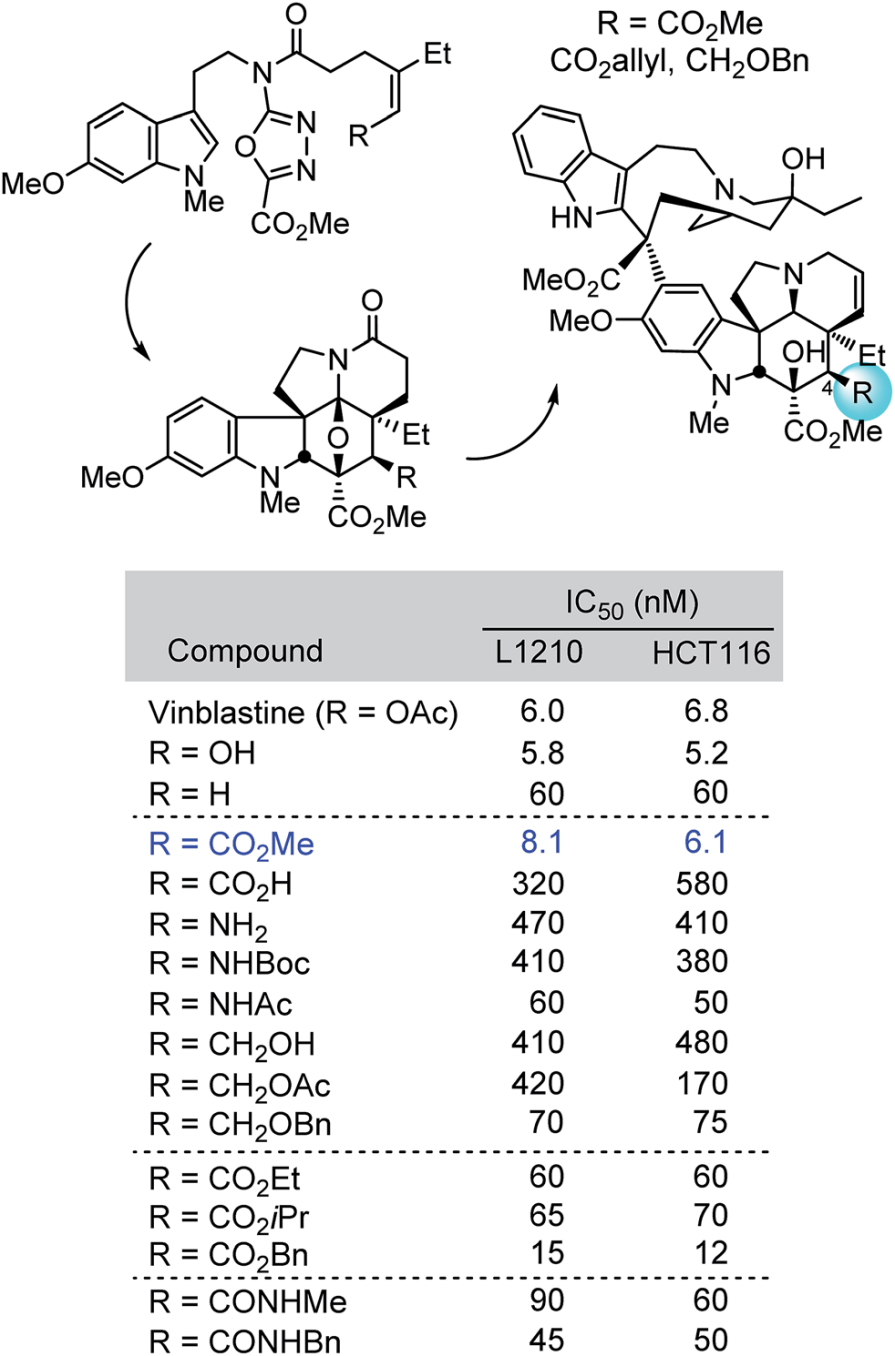
Summary of synthetic approach and cell growth inhibition properties of C4 modified vinblastines.

Herein, we described concise divergent^28^ total syntheses of a key series of C4 modified vinblastines, containing changes at a site that was not thought to intimately interact with the biological target tubulin yet impacts potency and constitutes a metabolic liability. Central to the approach was the development of an expanded scope of the 1,3,4-oxadiazole [4 + 2]/[3 + 2] cycloaddition cascade that permitted the direct installation of the desired C4 modifications in the vindoline subunit. Although originally reported to be unproductive, conditions where both electron-deficient trisubstituted alkenes and unactivated trisubstituted alkenes productively initiate the cycloaddition cascade were disclosed. The use of the cycloaddition cascade of three such substrates in concise total syntheses of a series of C4 modified vindolines, their single-step incorporation into the total synthesis of a series of 17 synthetic vinblastines, and their use in defining both the importance and surprising trends of the C4 substituent contribution to the biological properties of vinblastine were detailed (Fig. 6). Whereas the C4 acetate or a C4 alcohol contribute to the productive biological properties of vinblastine and desacetylvinblastine (**1** and **4** *vs.* **5**), replacement with a C4 amine, carboxylic acid, hydroxymethyl or acetoxymethyl substituent led to substantial reductions in potency (**28**, **34**, **41** and **42**). Introduction of an acetamide or *N*-methyl carboxamide substituent, which incorporate single heavy atom exchanges (amide NH for ester oxygen), provided compounds that matched the activity of desacetoxyvinblastine (**35** and **52** *vs.* **5**) but were still *ca.* 10-fold less active than vinblastine. In contrast, only the introduction of a C4 methyl ester (**29** *vs.* **1**), a constitutional isomer of vinblastine in which the carbonyl carbon and ester oxygen are transposed, provided a compound that matched the potency of vinblastine. Even the seemingly benign alteration of this equally active C4 methyl ester to the corresponding ethyl or isopropyl esters, and *N*-methyl carboxamide led to 10-fold reductions in activity (**29** *vs.* **46**, **47** and **52**). Examination of the early series of the C4 modified vinblastines revealed that they bind to tubulin with affinities that correlate with their functional growth inhibition. We suggest that this unanticipated impact on target binding affinity is derived from a previously unrecognized role the C4 substituent plays as a H-bond acceptor for α-tubulin Lys336 and Asn329 side chains at a site incapable of accommodating a substituent H-bond donor, placing the methyl groups of the C4 acetate and C4 methyl ester in a spatially well-defined small hydrophobic half pocket of a well-ordered loop that organizes around the side of bound substituent. This remarkable impact of the C4 substituent, its stringency, and even the magnitude of its effect are extraordinary, yet are analogous to observations made with other peripheral substituents found on vinblastine (*e.g.*, N1–Me, C16-methoxy, C20′-hydroxy,^22*b*^ C16′,^22*c*^ and C5^24*a*^). In instances when the productive properties of a natural product are directly related to its emergence in Nature and has undergone continued optimization by natural selection as is likely the case with vinblastine, it may be that such peripheral functionality is not easily subjected to structural modifications or simplifications.

Significantly, the compounds examined herein represent synthetic vinblastines prepared by total synthesis enabled by a powerful and now expanded oxadiazole cycloaddition cascade inspired by structures once thought too complex for such studies. Moreover, the compounds examined are themselves presently inaccessible by alternative methods, including natural product derivatization, late-stage functionalization, or biosynthetic methods. The examination of the key series of compounds revealed an unanticipated importance and defined the role of the C4 substituent in the expression of the natural product biological properties and provided one key analogue, the C4 methyl ester **29**, that matched the activity of the natural product. The important difference being that the C4 methyl ester, which is flanked by two quaternary centers, was found to be not nearly as susceptible to hydrolysis as the natural C4 acetate, suggesting the known metabolically labile site on the natural product might be replaced with a more stable, less accessible C4 methyl ester.
