# Supplementary material for: Total synthesis of a key series of vinblastines modified at C4 that define the importance and surprising trends in activity
Source: Chem Sci. 2016 Nov 3;8(2):1560–9. doi: 10.1039/c6sc04146a (PMC5302862; doi:10.1039/c6sc04146a)
Supplement: Supplementary file 1 [file SC-008-C6SC04146A-s001.pdf]

Electronic Supplementary Material (ESI) for Chemical Science.
This journal is © The Royal Society of Chemistry 2016

Supporting Information

Total synthesis of a key series of vinblastines modified at C4 that define the importance and surprising trends in activity

Shouliang Yang, Kuppusamy Sankar, Colin K. Skepper, Timothy J. Barker, John C. Lukesh III, Daniel M. Brody, Manuela Brutsch, and Dale L. Boger*

Department of Chemistry and the Skaggs Institute for Chemical Biology, The Scripps Research Institute, 10550 North Torrey Pines Road, La Jolla, California 92037, United States

Table of Contents

General methods--**S1**
Experimental Data --**S2-S44**
References --**S45**
NMR Spectra --**S45-S100**

General Methods. All commercial reagents were used without further purification unless otherwise noted. All reactions were performed in oven-dried (200 °C) glassware and under an inert atmosphere of anhydrous Ar unless otherwise noted. 1,3,5-Triisopropylbenzene (Oakwood) was distilled from CaH_2 and stored with neutral alumina for at least 72 h prior to use. Column chromatography was performed with silica gel 60. TLC was performed on EMD Millipore silica gel (250 μm) F254 glass plates and spots visualized by UV. PTLC was performed on EMD Millipore silica gel (250 and 500 μm) F254 glass plates. Optical rotations were determined on a Rudolph Research Analytical Autopol III automatic polarimeter using the sodium D line (λ = 589 nm) at room temperature. ^{1}H and ^{13}C NMR was recorded on a Bruker 500 MHz and 600 MHz spectrometers. Chemical shifts are reported in ppm from an internal standard of residual $CHCl_3$ (δ 7.26 for 1H). Proton chemical data are reported as follows: chemical shift (δ), multiplicity (ovlp = overlapping, br = broad, s = singlet, d = doublet, t = triplet, q = quartet, m = multiplet), coupling constant, and integration. High resolution mass spectra were obtained on an Agilent ESI-TOF/MS using Agilent ESI-L low concentration tuning mix as internal high resolution calibration standards. The purity of each tested compound (>95%) was determined on an Agilent 1100 LC/MS

instrument using a ZORBAX SB-C8 column (3.5 mm, 4.6 mm ×50 mm, with a flow rate of 0.75 mL/min and detection at 220 and 254 nm) with a 10−98% acetonitrile/water/0.1% formic acid gradient.

Experimental Data.

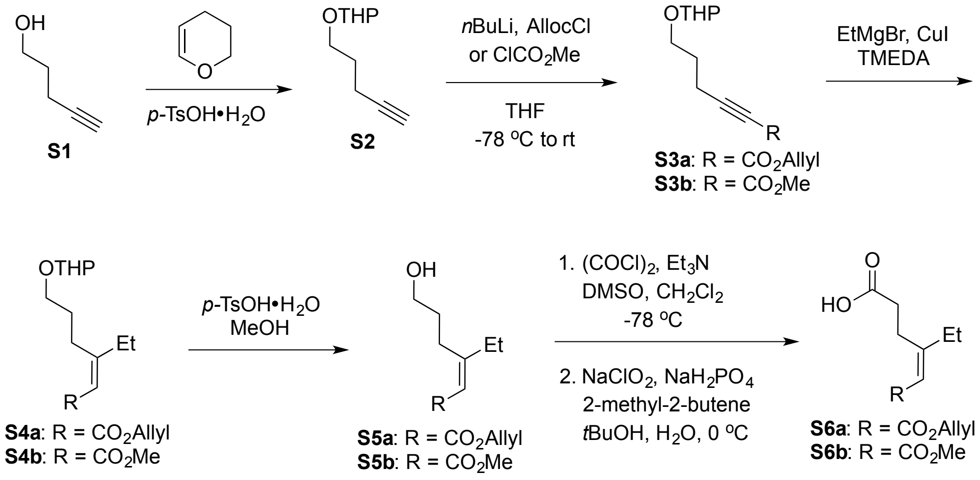

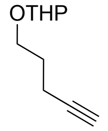

S2

Compound S2. *p*-TsOH (565 mg, 2.97 mmol) was added to a stirred mixture of pent-4-yn-1-ol (5 g, 59.4 mmol) in CH_2Cl_2 (74.3 mL) at room temperature. After 1 h the mixture was poured into saturated aqueous $NaHCO_3$ (200 mL). The layers were separated and the aqueous layer was extracted with CH_2Cl_2 (50 mL). The combined organic extracts were washed with saturated aqueous NaCl, dried (Na_2SO_4) and concentrated under reduced pressure. Flash chromatography (SiO_2, 10% Et_2O/hexanes) gave **S2** (9.13 g, 91%) as a clear, pale yellow liquid: ^{1}H NMR (300 MHz, $CDCl_3$) δ 4.54 (m, 1H), 3.78 (m, 2H), 3.44 (m, 2H), 2.25 (dt, J = 6.8, 2.5 Hz, 2H), 1.89 (t, J = 2.5 Hz, 1H), 1.85–1.40 (m, 8H); ^{13}C NMR (75 MHz, $CDCl_3$) δ 98.7, 83.8, 68.4, 65.7, 62.1, 30.6, 28.6, 25.4, 19.4, 15.2.

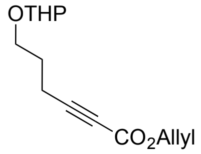

S3a

Compound S3a. *n*-BuLi (6.53 mmol, 2.61 mL of a 2.5 M solution in hexanes) was added dropwise to a solution of **S2** (1.0 g, 5.94 mmol) in THF (11.9 mL) at −78 °C. After 1 h, allyl chloroformate (2.15 g, 17.8 mmol) was added dropwise and the resulting mixture was warmed to room temperature over 4 h. The reaction mixture was poured into saturated aqueous $NaHCO_3$ (100 mL) and extracted with Et_2O (3 × 50 mL). The combined organic extracts were washed with saturated aqueous NaCl, dried (Na_2SO_4)

and concentrated under reduced pressure. Flash chromatography (SiO_2, 20% Et_2O/hexanes) gave **S3a** (1.34 g, 89%) as a pale yellow oil: ^1H NMR (600 MHz, $CDCl_3$) δ 5.88 (ddt, J = 17.0, 10.5, 6.0 Hz, 1H), 5.31 (dq, J = 17.0, 1.4 Hz, 1H), 5.23 (dq, J = 10.5, 1.4 Hz, 1H), 4.60 (dt, J = 6.0, 1.2 Hz, 2H), 4.56 (dd, J = 4.2, 2.9 Hz, 1H), 3.82 – 3.78 (m, 1H), 3.77 (dt, J = 9.6, 6.0 Hz, 1H), 3.48–3.44 (m, 1H), 3.42 (dt, J = 10.2, 6.0 Hz, 1H), 2.43 (dt, J = 7.4, 2.8 Hz, 2H), 1.87–1.82 (m, 2H), 1.79–1.71 (m, 1H), 1.68–1.64 (m, 1H), 1.56–1.44 (m, 4H); ^{13}C NMR (150 MHz, $CDCl_3$) δ 153.3, 131.2, 119.1, 98.7, 89.2, 72.9, 66.2, 65.4, 62.2, 30.5, 27.7, 25.3, 19.4, 15.6; IR (neat) ν_{max} 2941, 2870, 1709, 1442, 1357, 1322, 1237, 1200, 1157, 1134, 1120, 1062, 1032, 987, 935, 868, 813, 750 cm^{-1}; ESI-TOF HRMS *m*/*z* 275.1265 (M+Na$^+$, $C_{14}H_{20}O_4Na$ requires 275.1254).

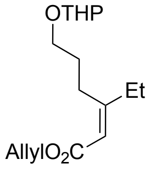

S4a

Compound S4a. Ethylmagnesium bromide (59.2 mmol, 59.2 mL of a 1 M solution in THF) was added dropwise to a suspension of CuI (11.3 g, 59.2 mmol) in THF (164 mL) at −45 °C. TMEDA (17.2 g, 148 mmol) was added dropwise. The resulting mixture was stirred for 30 min at −45 °C then cooled to −78 °C. Alkyne **S3a** (12.45 g, 49.3 mmol) was added slowly as a solution in THF (ca.10 mL). After stirring for 1.5 h at −78 °C, the reaction was quenched by addition of MeOH (15 mL) via syringe pump at a rate of 5 mL/h. Saturated aqueous NH_4Cl (60 mL) was added dropwise and the mixture warmed to room temperature and poured into saturated aqueous NH_4Cl (600 mL). The layers were separated and the aqueous layer was extracted with Et_2O (2 × 200 mL). The combined organic extracts were washed with saturated aqueous NH_4Cl (3 × 200 mL) and saturated aqueous NaCl then dried (Na_2SO_4) and concentrated under reduced pressure. Flash chromatography (SiO_2, 10% EtOAc/hexanes) gave **S4a** (10.48 g, 75%) as a clear, colorless oil. Good selectivity for the *Z* over *E* isomer was strongly dependent upon the slow quench with MeOH. It can be difficult to maintain magnetic stirring for during the quench due to precipitated copper salts, and there may be a correlation between a lack of stirring during the quench and low selectivity: ^1H NMR (500 MHz, $CDCl_3$) δ 5.93 (ddt, J = 17.3, 10.5, 5.6 Hz, 1H), 5.66 (s, 1H), 5.30 (dq, J = 17.3, 1.4 Hz, 1H), 5.21 (dq, J = 10.5, 1.4 Hz, 1H), 4.57 (dt, J = 5.5, 1.5 Hz, 2H), 4.56 (m, 1H), 3.85 (ddd, J = 11.5, 7.8, 3.5 Hz, 1H), 3.75 (dt, J = 9.5, 7.0 Hz, 1H), 3.50–3.45 (m, 1H), 3.41 (dt, J = 10.0, 6.8 Hz, 1H), 2.67 (m, 2H), 2.20 (dq, J = 7.5, 1.5 Hz, 2H), 1.85–1.65 (m, 4H), 1.58–1.47 (m, 4H), 1.05 (t, J = 7.5 Hz, 3H); ^{13}C NMR (125 MHz, $CDCl_3$) δ 166.1, 166.0, 132.6, 117.8, 114.2, 98.8, 67.4, 64.3, 62.3, 31.2, 30.7, 29.0, 28.7, 25.5, 19.6, 12.0; IR (neat) ν_{max} 2939, 2872, 1714, 1642, 1453, 1383, 1318, 1227, 1176, 1137, 1075, 1029, 989, 928, 866, 813, 751 cm^{-1}; ESI-TOF HRMS *m*/*z* 305.1736 (M+Na$^+$, $C_{16}H_{26}O_4Na$ requires 305.1723).

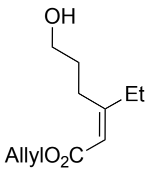

S5a

Compound S5a. *p*-TsOH (356 mg, 1.87 mmol) was added to a mixture of **S4a** (5.29 g, 18.7 mmol) in MeOH (93.5 mL). After stirring for 2 h, the mixture was poured into saturated aqueous $NaHCO_3$ (500 mL) and extracted with Et_2O (3×100 mL). The combined organic extracts were washed with saturated aqueous NaCl, dried (Na_2SO_4) and concentrated under reduced pressure. Flash chromatography (SiO_2, 30% EtOAc/hexanes) provided **S5a** (3.73 g, quant.) as a clear, colorless oil: ^{1}H NMR (400 MHz, $CDCl_3$) δ 5.92 (ddt, *J* = 17.2, 10.4, 5.6 Hz, 1H), 5.76 (s, 1H), 5.31 (dq, *J* = 17.2, 1.4 Hz, 1H), 5.22 (dq, *J* = 10.4, 1.4 Hz, 1H), 4.59 (dt, *J* = 6.0, 1.4 Hz, 2H), 3.55 (m, 2H), 2.70 (t, *J* = 7.0 Hz, 2H), 2.17 (dq, *J* = 7.4, 1.4 Hz, 2H), 1.73 (m, 2H), 1.06 (t, *J* = 7.4 Hz, 3H); ^{13}C NMR (150 MHz, $CDCl_3$) δ 167.2, 165.7, 132.2, 118.3, 115.0, 64.8, 60.7, 30.7, 30.6, 27.8, 12.0; IR (neat) ν_{max} 3411, 2967, 2938, 2877, 1713, 1641, 1454, 1421, 1389, 1314, 1216, 1165, 1143, 1058, 1030, 994, 930, 867 cm^{-1}; ESI-TOF HRMS *m/z* 199.1332 (M+H$^+$, $C_{11}H_{19}O_3$ requires 199.1329).

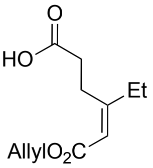

S6a

Compound S6a. DMSO (5.88 g, 75.2 mmol) was added to a mixture of oxalyl chloride (4.78 g, 37.6 mmol) in CH_2Cl_2 (188 mL) at −78 °C. After 30 min, alcohol **S5a** (3.73 g, 18.8 mmol) was added and after a further 30 min Et_3N (11.41 g, 112.8 mmol) was added. The mixture was allowed to warm to room temperature over 1 h, then poured into water (500 mL). The layers were separated and the aqueous layer was extracted with CH_2Cl_2 (2×100 mL). The combined organic extracts were washed with aqueous 0.1 N HCl (100 mL), saturated aqueous $NaHCO_3$ (100 mL) and saturated aqueous NaCl then dried (Na_2SO_4) and concentrated under reduced pressure. The crude aldehyde was used immediately. A solution of $NaClO_2$ (5.10 g, 56.4 mmol) and NaH_2PO_4 (10.38 g, 75.2 mmol) in water (51 mL) was added to a mixture of aldehyde (18.8 mmol), 2-methyl-2-butene (376 mL) and *t*-BuOH (376 mL) at 0 °C. The mixture was warmed to room temperature over 4 h, then poured into water (1.5 L). The layers were separated and the aqueous layer was extracted with EtOAc (2×300 mL). The combined organic extracts were washed with saturated aqueous NaCl, dried (Na_2SO_4) and concentrated under reduced pressure. Flash chromatography (SiO_2, 20% EtOAc/hexanes then 40:59:1 EtOAc/hexanes/AcOH) gave **S6a** (3.46 g, 87%) as a clear, pale yellow oil: ^{1}H NMR (400 MHz, $CDCl_3$) δ 5.93 (ddt, *J* = 17.2, 10.4, 5.8 Hz, 1H), 5.71 (s, 1H), 5.31 (dq, *J* = 17.2, 1.6 Hz, 1H), 5.22 (dq, *J* = 10.4, 1.6 Hz, 1H), 4.59 (dt, *J* = 6.0, 1.4 Hz, 2H), 2.87 (m, 2H), 2.53 (m, 2H), 2.20 (dq, *J* = 7.4, 1.4 Hz, 2H), 1.07 (t, *J* = 7.4 Hz, 3H); ^{13}C NMR

(150 MHz, $CDCl_3$) δ 178.1, 165.9, 163.8, 132.4, 118.2, 115.3, 64.6, 32.8, 31.4, 27.5, 11.9; IR (neat) ν_{max} 2969, 1709, 1419, 1263, 1219, 1166, 1084, 1018, 994, 931, 869, 800 cm^{-1}; ESI-TOF HRMS *m/z* 213.1128 ($M+H^+$, $C_{11}H_{17}O_4$ requires 213.1121).

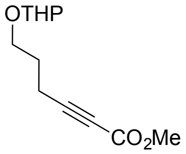

S3b

Compound S3b. *n*-BuLi (14.8 mmol, 6.22 mL of a 2.5 M solution in hexanes) was added dropwise to a solution of **S2** (2.49 g, 14.8 mmol) in THF (30 mL) at −78 °C. After 1 h, methyl chloroformate (4.20 g, 44.4 mmol) was added dropwise and the resulting mixture was warmed to room temperature over 4 h. The reaction mixture was poured into saturated aqueous $NaHCO_3$ (250 mL) and extracted with Et_2O (3 × 100 mL). The combined organic extracts were washed with saturated aqueous NaCl, dried (Na_2SO_4) and concentrated under reduced pressure. Flash chromatography (SiO_2, 20% Et_2O/hexanes) gave **S3b** (3.23 g, 96%) as a pale yellow oil: ^{1}H NMR (300 MHz, $CDCl_3$) δ 4.52 (br t, *J* = 3.3 Hz, 1H), 3.81–3.72 (m, 2H), 3.69 (s, 3H), 3.48–3.37 (m, 2H), 2.41 (t, *J* = 7.2 Hz, 2H), 1.80 (p, *J* = 6.6 Hz, 2H), 1.74–1.43 (m, 6H); ^{13}C NMR (75 MHz, $CDCl_3$) δ 154.0, 98.7, 89.1, 72.9, 65.4, 62.1, 52.4, 30.5, 27.7, 25.3, 19.4, 15.6; IR (neat) ν_{max} 2944, 2872, 1712, 1434, 1248, 1199, 1120, 1135, 1062, 1032, 986, 868, 813, 751 cm^{-1}; ESI-TOF HRMS *m/z* 227.1279 ($M+H^+$, $C_{12}H_{18}O_4$ requires 227.1278).

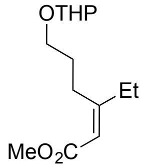

S4b

Compound S4b. Ethylmagnesium bromide (12.2 mmol, 12.2 mL of a 1 M solution in THF) was added dropwise to a suspension of CuI (2.53 g, 13.3 mmol) in THF (37 mL) at −45 °C. TMEDA (3.87 g, 33.3 mmol) was added dropwise. The resulting mixture was stirred for 30 min at −45 °C, then cooled to −78 °C. Alkyne **S3b** (2.5 g, 11.1 mmol) was added slowly as a solution in THF (ca. 5 mL). After stirring for 1.5 h at −78 °C, the reaction was quenched by addition of MeOH (5 mL) via syringe pump at a rate of 5 mL/h. Saturated aqueous NH_4Cl (10 mL) was added dropwise and the mixture warmed to room temperature and poured into saturated aqueous NH_4Cl (300 mL). The layers were separated and the aqueous layer was extracted with Et_2O (3 × 100 mL). The combined organic extracts were washed with saturated aqueous NH_4Cl (3 × 100 mL) and saturated aqueous NaCl, then dried (Na_2SO_4) and concentrated under reduced pressure. Flash chromatography (SiO_2, 10% EtOAc/hexanes) gave **S4b** (2.3 g, 82%) as a clear, colorless oil: ^{1}H NMR (600 MHz, $CDCl_3$) δ 5.60 (s, 1H), 4.53 (dd, *J* = 4.4, 3.3 Hz, 1H), 3.82 (ddd, *J* = 10.8, 8.1, 3.0 Hz, 1H), 3.72 (dt, *J* = 9.6, 6.6 Hz, 1H), 3.63 (s, 3H), 3.47–3.43 (m, 1H), 3.39 (dt, *J* = 9.6, 6.6 Hz, 1H), 2.63 (m, 2H), 2.16 (dq, *J* = 7.4, 1.2 Hz, 2H), 1.80–1.76 (m, 1H), 1.72 (p, *J* = 7.4 Hz, 2H), 1.68–1.63 (m, 1H), 1.55–1.45 (m, 4H), 1.01 (t, *J* = 7.2 Hz, 1H); ^{13}C NMR (150 MHz, $CDCl_3$) δ 166.9, 165.7, 114.0,

98.7 , 67.4, 62.2, 50.7, 31.1, 30.7, 28.9, 28.6, 25.4, 19.6, 12.0; IR (neat) ν_{max} 2941, 2871, 1716, 1643, 1435, 1369, 1319, 1227, 1177, 1145, 1119, 1075, 1029, 989, 903, 866, 813, 733 cm^{-1}; ESI-TOF HRMS *m/z* 279.1566 ($M+Na^+$, $C_{14}H_{24}O_4Na$ requires 279.1567).

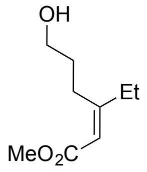

S5b

Compound S5b. *p*-TsOH (347 mg, 1.82 mmol) was added to a mixture of **S4b** (4.67 g, 18.2 mmol) in MeOH (91 mL). After stirring for 2 h, the mixture was poured into saturated aqueous $NaHCO_3$ (500 mL) and extracted with Et_2O (3×100 mL). The combined organic extracts were washed with saturated aqueous NaCl, dried (Na_2SO_4) and concentrated under reduced pressure. Flash chromatography (30–50% EtOAc/hexanes gradient) provided **S5b** (3.03 g, quant.) as a clear, colorless oil: ^1H NMR (300 MHz, $CDCl_3$) δ 5.73 (s, 1H), 3.68 (s, 3H), 3.54 (q, *J* = 6.0 Hz, 2H), 3.05 (t, *J* = 6.6 Hz, 1H), 2.70 (t, *J* = 7.1 Hz, 2H), 2.17 (q, *J* = 7.5 Hz, 2H), 1.73 (p, *J* = 6.4 Hz, 2H), 1.06 (t, *J* = 7.4 Hz, 3H); ^{13}C NMR (150 MHz, $CDCl_3$) δ 168.0, 165.4, 114.9 , 60.7, 51.2, 30.60, 30.58, 27.7, 12.0; IR (neat) ν_{max} 3411 (br), 2945, 2876, 1713, 1641, 1434, 1373, 1316, 1220, 1166, 1027, 978, 909, 865, 801, 730 cm^{-1}; ESI-TOF HRMS *m/z* 173.1173 ($M+H^+$, $C_9H_{17}O_3$ requires 173.1172).

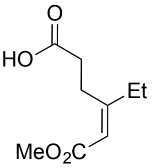

S6b

Compound S6b. DMSO (5.44 g, 69.9 mmol) was added to a mixture of oxalyl chloride (4.42 g, 34.8 mmol) in CH_2Cl_2 (174 mL) at −78 °C. After 30 min, alcohol **S5b** (3.0 g, 17.4 mmol) was added and after a further 30 min Et_3N (10.56 g, 104 mmol) was added. The mixture was allowed to warm to room temperature over 1 h, then poured into water (500 mL). The layers were separated and the aqueous layer was extracted with CH_2Cl_2 (2×100 mL). The combined organic extracts were washed with aqueous 0.1 N HCl (150 mL), saturated aqueous $NaHCO_3$ (150 mL) and saturated aqueous NaCl then dried (Na_2SO_4) and concentrated under reduced pressure. The crude aldehyde was used immediately. A solution of $NaClO_2$ (4.72 g, 52.2 mmol) and NaH_2PO_4 (9.60 g, 69.6 mmol) in water (47 mL) was added to a mixture of aldehyde (17.4 mmol), 2-methyl-2-butene (348 mL) and *t*-BuOH (348 mL) at 0 °C. The mixture was warmed to room temperature over 4 h, then poured into water (1.5 L). The layers were separated and the aqueous layer was extracted with EtOAc (2×300 mL). The combined organic extracts were washed with saturated aqueous NaCl, dried (Na_2SO_4) and concentrated under reduced pressure. Flash chromatography (SiO_2, 20% EtOAc/hexanes then 40:59:1 EtOAc/hexanes/AcOH) gave **S6b** (2.51 g, 78%) as a clear, pale yellow oil: ^1H NMR (600 MHz, $CDCl_3$) δ 5.67 (br s, 1H), 3.67 (s, 3H), 2.86 (m, 2H), 2.52 (m, 2H), 2.19 (q,

J = 7.2 Hz, 2H), 1.05 (t, J = 7.4 Hz, 3H); ^{13}C NMR (150 MHz, $CDCl_3$) δ 178.9, 166.7, 163.4, 115.2, 51.0, 32.8, 31.4, 27.4, 11.9; IR (neat) ν_{max} 2969, 1706, 1435, 1392, 1284, 1221, 1168, 1085, 1061, 1028, 923, 869, 838, 803 cm^{-1}; ESI-TOF HRMS *m/z* 187.0969 ($M+H^+$, $C_9H_{15}O_4$ requires 187.0965).

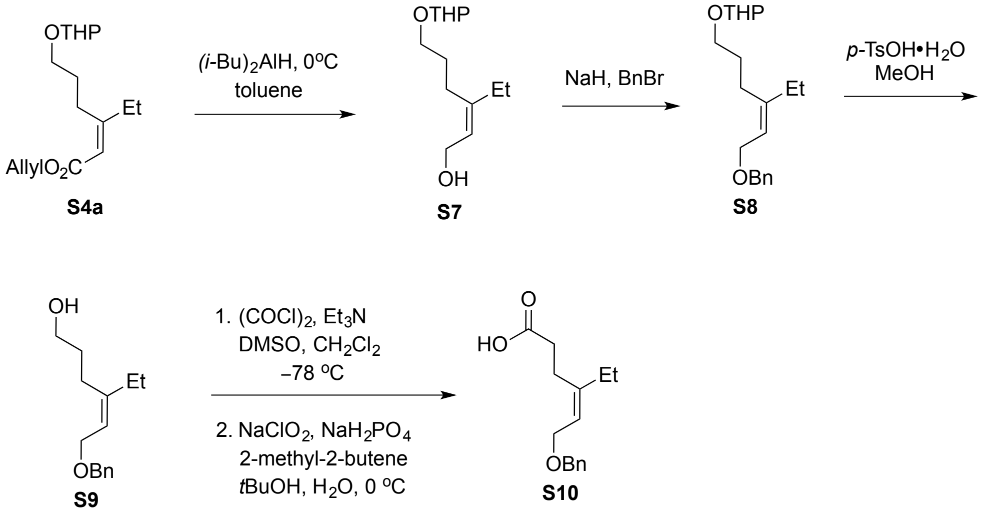

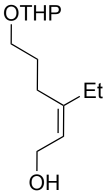

S7

Compound S7. Diisobutylaluminum hydride (35 mmol, 35 mL of 1 M solution in hexanes) was added dropwise to a solution of **S4a** (4.7 g, 16.7 mmol) in toluene (56 mL) at 0 °C. The resulting mixture was stirred for 30 min at 0 °C. The reaction was quenched by addition of MeOH (100 mL) and filtered through celite. Flash chromatography (SiO_2, 50% EtOAc/hexanes) gave **7** (3.43 g, 90%) as a clear, colorless oil: ^1H NMR (500 MHz, $CDCl_3$) δ 5.49 (t, J = 7.3 Hz, 1H), 4.53–4.51 (m, 1H), 4.21 (dd, J = 12.2, 7.9 Hz, 1H), 4.06 (dd, J = 12.2, 6.6 Hz, 1H), 3.95–3.84 (m, 1H), 3.72 (ddd, J = 9.7, 7.8, 5.3 Hz, 1H), 3.51–3.46 (m, 1H), 3.39 (dt, J = 9.7, 5.7 Hz, 1H), 2.30 (dt, J = 13.5, 7.7 Hz, 1H), 2.20–2.09 (m, 2H), 2.04 (q, J = 7.5 Hz, 2H), 1.88–1.77 (m, 1H), 1.77–1.61 (m, 3H), 1.61–1.46 (m, 4H), 1.02 (t, J = 7.5 Hz, 3H); ^{13}C NMR (125 MHz, $CDCl_3$) δ 143.8, 123.7, 99.3, 66.4, 63.1, 58.6, 30.9, 29.0, 27.9, 26.3, 25.4, 20.1, 12.5; IR (neat) ν_{max} 3512, 2939, 2871, 1456, 1176, 1136, 1024 cm^{-1}; ESI-TOF HRMS *m/z* 228.1801 ($M+H^+$, $C_{13}H_{24}O$ requires 228.1798).

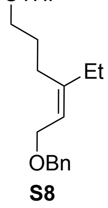

S8

Compound S8. Alcohol **S7** (202 mg, 0.89 mmol) was added to a suspension of NaH (24 mg, 1.00 mmol) in THF (6 mL) at 0 °C. After 20 min, benzyl bromide (119 μL,

1.00 mmol) was added and the resulting mixture was allowed to warm to room temperature and stir for 24 h. The reaction was quenched with the addition of saturated aqueous NH_4Cl and the reaction mixture was extracted with CH_2Cl_2 then dried (Na_2SO_4) and concentrated under reduced pressure. Flash chromatography (SiO_2, 10% EtOAc/hexanes) provided **S8** (246 mg, 87%) as a clear, yellow oil: ^{1}H NMR (600 MHz, $CDCl_3$) δ 7.36–7.26 (m, 5H), 5.41 (t, *J* = 6.8 Hz, 1H), 4.56–4.54 (m, 1H), 4.51 (s, 2H), 4.05 (d, *J* = 6.8 Hz, 2H), 3.85 (ddd, *J* = 11.1, 7.6, 3.3 Hz, 1H), 3.69 (dt, *J* = 9.6, 6.6 Hz, 1H), 3.49 (dt, *J* = 10.7, 5.1 Hz, 1H), 3.34 (dt, *J* = 9.6, 6.6 Hz, 1H), 2.17–2.05 (m, 4H), 1.85–1.53 (m, 8H), 1.03 (t, *J* = 7.4 Hz, 3H); ^{13}C NMR (150 MHz, $CDCl_3$) δ 145.6, 138.7, 128.5, 127.9, 127.7, 120.4, 99.0, 72.3, 67.2, 66.7, 62.5, 30.9, 29.5, 28.8, 27.4, 25.6, 19.8, 12.6; IR (neat) ν_{max} 2937, 2868, 1453, 1072, 1030 cm^{-1}; ESI-TOF HRMS *m*/*z* 319.2268 (M+H$^+$, $C_{20}H_{30}O_3$ requires 319.2268).

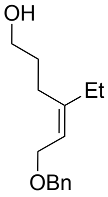

S9

Compound S9. *p*-TsOH (104 mg, 0.55 mmol) was added to a mixture of **S8** (3.51 g, 11 mmol) in MeOH (50 mL). After stirring for 2 h, the mixture was diluted with saturated aqueous NH_4Cl (4 mL) and extracted with CH_2Cl_2 (3×20 mL). The combined organic extracts were washed with saturated aqueous $NaHCO_3$ (2 mL) and saturated aqueous NaCl then dried (Na_2SO_4) and concentrated under reduced pressure. Flash chromatography (SiO_2, 35% EtOAc/hexanes) gave **9** (2.24 g, 87%) as a clear, colorless oil: ^{1}H NMR (500 MHz, $CDCl_3$) δ 7.36–7.27 (m, 5H), 5.49 (t, *J* = 7.2 Hz, 1H), 4.53 (s, 2H), 4.02 (d, *J* = 7.1 Hz, 2H), 3.56 (q, *J* = 6.0 Hz, 2H), 2.19 (t, *J* = 7.4 Hz, 2H), 2.09–2.04 (m, 2H), 1.67–1.61 (m, 2H), 1.03 (t, *J* = 7.4 Hz, 3H); ^{13}C NMR (125 MHz, $CDCl_3$) δ 146.8, 138.3, 128.5, 128.2, 127.8, 120.1, 72.6, 66.1, 61.9, 30.8, 29.1, 26.4, 12.5; IR (neat) ν_{max} 3402 (br), 2961, 2869, 1454, 1059, 1029 cm^{-1}; ESI-TOF HRMS *m*/*z* 235.1693 (M+H$^+$, $C_{15}H_{22}O_2$ requires 235.1692).

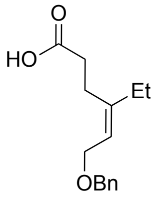

S10

Compound S10. DMSO (2.67 mL, 37.6 mmol) was added to a mixture of oxalyl chloride (1.59 mL, 18.8 mmol) in CH_2Cl_2 (150 mL) at −78 °C. After 30 min, alcohol **S9** (2.20 g, 9.4 mmol) was added and after a further 30 min Et_3N (7.86 mL, 56.4 mmol) was added. The mixture was allowed to warm to room temperature over 1 h, then poured into water (100 mL). The layers were separated and the aqueous layer was extracted with CH_2Cl_2 (2×100 mL). The combined organic extracts were washed with aqueous 0.1 N HCl and saturated aqueous $NaHCO_3$ (100 mL each) and saturated aqueous NaCl then dried (Na_2SO_4) and concentrated under reduced pressure. The crude

aldehyde was used immediately. A solution of $NaClO_2$ (3.19 g, 28.2 mmol) and NaH_2PO_4 (5.87 g, 37.6 mmol) in water (10 mL) was added to a mixture of aldehyde (9.4 mmol), 2-methyl-2-butene (50 mL) and *t*-BuOH (160 mL) at 0 °C. The mixture was warmed to room temperature over 2 h, then poured into water (1.5 L). The layers were separated and the aqueous layer was extracted with EtOAc (2×300 mL). The combined organic extracts were washed with saturated aqueous NaCl, dried (Na_2SO_4) and concentrated under reduced pressure. Flash chromatography (SiO_2, 20% EtOAc/hexanes) gave **S10** (1.67 g, 72%) as a clear, pale yellow oil: ^{1}H NMR (500 MHz, $CDCl_3$) δ 7.37–7.27 (m, 5H), 5.47 (t, *J* = 6.7 Hz, 1H), 4.52 (s, 2H), 4.07 (d, *J* = 6.7 Hz, 2H), 2.41–2.40 (m, 4H), 2.07 (q, *J* = 7.3 Hz, 2H), 1.03 (t, *J* = 7.4 Hz, 3H); ^{13}C NMR (150 MHz, $CDCl_3$) δ 179.3, 143.8, 138.3, 128.5, 128.0, 127.7, 121.5, 72.4, 66.4, 33.2, 29.2, 25.8, 12.5; IR (neat) ν_{max} 3500–2500 (br), 2964, 2872, 1704, 1453, 1069 cm^{-1}; ESI-TOF HRMS *m/z* 249.1485 (M+H$^+$, $C_{15}H_{20}O_3$ requires 249.1485).

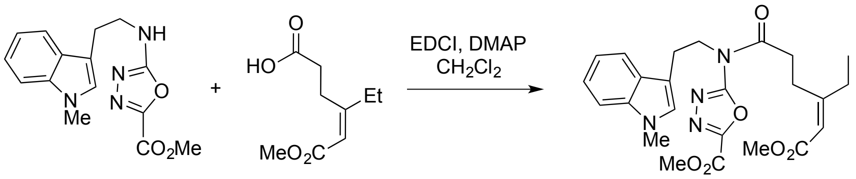

S11 **S6b** **7a**

Compound 7a. EDCI (516 mg, 2.69 mmol) and DMAP (328 g, 2.69 mmol) were added to a solution of **S6b** (500 mg, 2.69 mmol) in CH_2Cl_2 (50 mL) and oxadiazole **S11**[S1] (269 mg, 0.90 mmol) was added. After stirring for 2.5 h, the mixture was concentrated under reduced pressure. Flash chromatography (SiO_2, 25–35% EtOAc/hexanes gradient) gave **7a** (300 mg, 71%) as a white solid: ^{1}H NMR (500 MHz, $CDCl_3$) δ 7.69 (dt, *J* = 7.9, 1.1 Hz, 1H), 7.26–7.24 (m, 1H), 7.20 (ddd, *J* = 8.1, 6.8, 1.2 Hz, 1H), 7.11 (ddd, *J* = 8.0, 6.8, 1.2 Hz, 1H), 6.87 (s, 1H), 5.68 (s, 1H), 4.22 (t, *J* = 7.6 Hz, 1H), 3.99 (s, 3H), 3.72 (s, 3H), 3.64 (s, 3H), 3.12 (dd, *J* = 8.5, 6.6 Hz, 2H), 3.04–2.97 (m, 2H), 2.97–2.88 (m, 2H), 2.21 (q, *J* = 7.3 Hz, 2H), 1.08 (t, *J* = 7.3 Hz, 3H); ^{13}C NMR (150 MHz, $CDCl_3$) δ 171.8, 166.8, 163.6, 162.1, 154.2, 153.5, 137.1, 127.7, 127.6, 121.8, 119.2, 119.0, 115.4, 110.1, 109.3, 53.7, 51.1, 48.0, 35.1, 32.7, 31.5, 27.9, 24.4, 12.1; IR (neat) ν_{max} 2947, 1749, 1711, 1565, 1153 cm^{-1}; ESI-TOF HRMS *m/z* 469.2078 (M+H$^+$, $C_{24}H_{28}N_4O_6$ requires 469.2082).

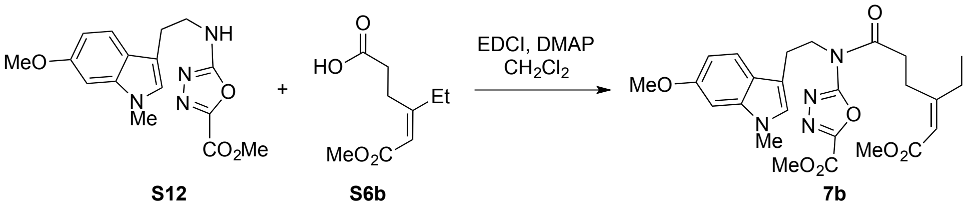

S12 **S6b** **7b**

Compound 7b. EDCI (2.59 g, 13.5 mmol) and DMAP (13.5 mmol) were added to a mixture of **S12**[S1] (2.23 g, 6.74 mmol) and **S6b** (2.51 g, 13.5 mmol) in CH_2Cl_2 (67.4 mL). After stirring for 1.25 h, the solvent was evaporated under reduced pressure to give the crude residue. Flash chromatography (SiO_2, 50% EtOAc/hexanes) gave **7b** (2.71 g, 81%) as a pale yellow solid: ^{1}H NMR (600 MHz, $CDCl_3$) δ 7.52 (d, *J* = 8.6 Hz, 1H), 6.75 (dd, *J* = 8.6, 2.3 Hz, 1H), 6.72 (s, 1H), 6.66 (d, *J* = 2.3 Hz, 1H), 5.65 (s, 1H),

4.18 (t, *J* = 7.5 Hz, 2H), 3.96 (s, 3H), 3.84 (s, 3H), 3.622 (s, 3H), 3.617 (s, 3H), 3.04 (t, *J* = 7.5 Hz, 2H), 2.97 (t, *J* = 7.7 Hz, 2H), 2.89 (t, *J* = 7.7 Hz, 2H), 2.18 (dq, *J* = 7.2, 1.2 Hz, 2H), 1.05 (t, *J* = 7.2 Hz, 3H); ^{13}C NMR (150 MHz, $CDCl_3$) δ 171.6, 166.6, 163.5, 161.9, 156.4, 154.0, 153.2, 137.6, 126.4, 121.7, 119.5, 115.2, 109.8, 109.0, 92.6, 55.6, 53.5, 50.9, 47.7, 34.9, 32.5, 31.3, 27.7, 24.3, 11.9; mp 112–113 °C; IR (neat) ν_{max} 2050, 1748, 1709, 1644, 1623, 1564, 1439, 1412, 1385, 1332, 1256, 1225, 1154, 1067, 1033, 911, 870, 813, 729, 677, 638 cm^{-1}; ESI-TOF HRMS *m*/*z* 499.2183 (M+H$^+$, $C_{25}H_{31}N_4O_7$ requries 499.2187).

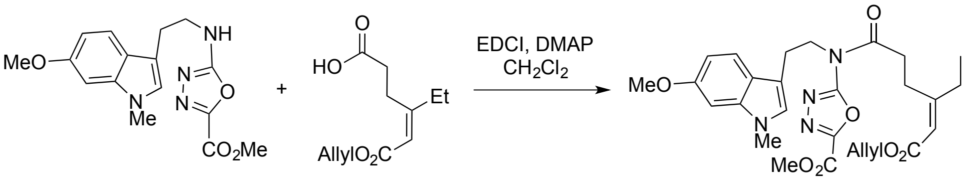

Compound 7c. EDCI (1.39 g, 7.27 mmol), DMAP (887 mg, 7.27 mmol) and **S6a** (800 mg, 2.42 mmol) were added to a mixture of **S12** (1.54 g, 7.27 mmol) in CH_2Cl_2 (72.7 mL). After stirring for 1.5 h, the volatiles were evaporated under reduced pressure and the crude residue purified directly by flash chromatography (SiO_2, 40% EtOAc/hexanes) to give **7c** (1.06 g, 84%) as a pale yellow solid: ^{1}H NMR (600 MHz, $CDCl_3$) δ 7.53 (d, *J* = 8.5 Hz, 1H), 6.76 (dd, *J* = 8.5, 2.3 Hz, 1H), 6.72 (s, 1H), 6.67 (d, *J* = 2.3 Hz, 1H), 5.89 (ddt, *J* = 16.8, 10.8, 6.0 Hz, 1H), 6.72 (s, 1H), 5.30–5.26 (m, 1H), 5.20–5.18 (m, 1H), 4.53 (dt, *J* = 6.0, 1.2 Hz, 2H), 4.18 (t, *J* = 7.5 Hz, 2H), 3.97 (s, 3H), 3.84 (s, 3H), 3.63 (s, 3H), 3.04 (t, *J* = 7.5 Hz, 2H), 2.97 (t, *J* = 7.8 Hz, 2H), 2.90 (dd, *J* = 7.8 Hz, 2H), 2.19 (q, *J* = 7.2 Hz, 2H), 1.06 (t, *J* = 7.2 Hz, 3H); ^{13}C NMR (150 MHz, $CDCl_3$) δ 171.6, 165.7, 163.9, 161.9, 156.4, 154.0, 153.2, 137.6, 132.4, 126.4, 121.7, 119.5, 118.0, 115.3, 109.9, 109.0, 92.7, 64.4, 55.6, 53.5, 47.8, 34.9, 32.5, 31.3, 27.8, 24.3, 11.8; IR (neat) ν_{max} 2938, 1748, 1706, 1644, 1624, 1562, 1440, 1411, 1386, 1255, 1223, 1151, 1067, 1034, 991, 911, 869, 812, 729, 637 cm^{-1}; ESI-TOF HRMS *m*/*z* 525.2345 (M+H$^+$, $C_{27}H_{33}N_4O_7$ requires 525.2345).

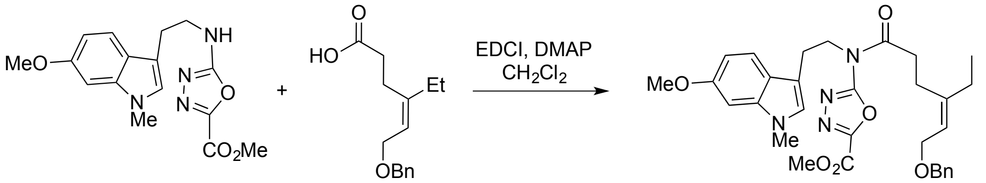

Compound 7d. EDCI (1.07 g, 5.6 mmol) and DMAP (683 mg, 5.6 mmol) were added to a solution of **S10** (1.4 g, 5.6 mmol) in CH_2Cl_2 (50 mL) and oxadiazole **S12** (618 mg, 1.87 mmol) was added. After stirring for 2.5 h, the mixture was concentrated under reduced pressure. Flash chromatography (SiO_2, 20–50% EtOAc/hexanes gradient) gave **7d** (850 mg, 81%) as a white solid: ^{1}H NMR (500 MHz, $CDCl_3$) δ 7.53 (d, *J* = 8.6 Hz, 1H), 7.34–7.23 (m, 5H), 6.78 (dd, *J* = 8.6, 2.2 Hz, 1H), 6.72 (s, 1H), 6.69 (d, *J* = 2.2 Hz, 1H), 5.44 (t, *J* = 6.8 Hz, 1H), 4.50 (s, 2H), 4.21–4.18 (m, 2H), 4.07 (d, *J* = 6.7 Hz 2H), 3.99 (s, 3H), 3.87 (s, 3H), 3.64 (s, 3H), 3.06 (t, *J* = 7.4 Hz, 2H), 2.85 (dd, *J* = 9.0, 7.0 Hz, 2H), 2.45 (dd, *J* = 8.9, 7.0 Hz, 2H), 2.04 (qd, *J* = 7.7, 3.8 Hz, 2H), 1.03 (t, *J* =

7.4 Hz, 3H); ^{13}C NMR (150 MHz, $CDCl_3$) δ 172.0, 162.0, 156.6, 154.1, 153.3, 143.9, 138.6, 137.8, 128.4, 127.9, 126.6, 121.8, 121.6, 119.6, 109.9, 109.2, 92.8, 72.4, 66.5, 55.8, 53.7, 47.9, 35.3, 32.7, 29.3, 26.2, 24.5, 12.5; IR (neat) ν_{max} 2960, 1747, 1702, 1623, 1560, 1439, 1408, 1255, 1149, 1067, 1035, 812, 729, 699 cm^{-1}; ESI-TOF HRMS *m/z* 561.2703 (M+H$^+$, $C_{31}H_{36}N_4O_6$ requires 561.2707).

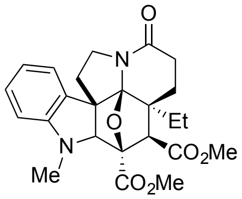

8a

Compound 8a. Argon was bubbled through a mixture of **7a** (60 mg, 0.128 mmol) in triisopropylbenzene (64 mL). The vessel was sealed and heated at 230 °C for 24 h. Upon cooling to room temperature, the reaction mixture was filtered through a plug of silica gel which was washed thoroughly with hexanes before products were eluted with 10% MeOH/CH_2Cl_2. The solvent was evaporated under reduced pressure. Flash chromatography (SiO_2, 30–50% EtOAc/hexanes gradient) provided **8a** (26 mg, 46%) as a pale yellow foam: ^{1}H NMR (500 MHz, $CDCl_3$) δ 7.19 (t, *J* = 7.7 Hz, 1H), 6.87 (d, *J* = 7.4 Hz, 1H), 6.68 (t, *J* = 7.3 Hz, 1H), 6.53 (d, *J* = 8.0 Hz, 1H), 4.00 (s, 2H), 3.98–3.93 (m, 1H), 3.86 (s, 3H), 3.66 (s, 3H), 3.59 (s, 1H), 2.92 (s, 3H), 2.51–2.45 (m, 1H), 2.40 (dd, *J* = 17.6, 5.2 Hz, 1H), 2.33 (dd, *J* = 13.2, 5.2 Hz, 1H), 2.27–2.21 (m, 2H), 1.46 (dd, *J* = 13.1, 5.3 Hz, 1H), 1.03–0.97 (m, 1H), 0.70 (t, *J* = 7.5 Hz, 3H), 0.59–0.53 (m, 1H); ^{13}C NMR (150 MHz, $CDCl_3$) δ 171.2, 170.9, 170.0, 153.2, 129.7, 128.4, 123.7, 118.8, 108.2, 105.5, 87.5, 83.5, 65.2, 55.6, 52.7, 52.0, 48.3, 47.1, 37.7, 35.9, 28.7, 24.7, 23.5, 8.9; IR (neat) ν_{max} 2951, 1736, 1668, 1376, 1214 cm^{-1}; ESI-TOF HRMS *m/z* 441.2037 (M+H$^+$, $C_{24}H_{28}N_2O_6$ requires 441.2026).

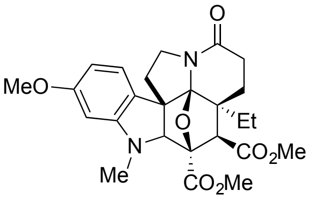

8b

Compound 8b. Ar was bubbled through a mixture of **7b** (100 mg, 200 μmol) in triisopropylbenzene (100 mL) for 20 min. The reaction vessel was sealed and heated at 230 °C for 24 h. Upon cooling the reaction was filtered through a plug of SiO_2. Residual triisopropylbenzene was washed from the plug with hexanes and products were eluted with 10% MeOH/CH_2Cl_2. The solvent was evaporated under reduced pressure and flash chromatography (SiO_2, 40–100% EtOAc/hexanes gradient) gave **8b** (4.0 mg, 42%) as an off-white foam: ^{1}H NMR (600 MHz, $CDCl_3$) δ 6.72 (d, *J* = 8.4 Hz, 1H), 6.19 (dd, *J* = 8.4, 2.4 Hz, 1H), 6.06 (d, *J* = 2.4, Hz, 1H), 3.99 (s, 1H), 3.96–3.89 (m, 2H), 3.84 (s, 3H), 3.77 (s, 3H), 3.64 (s, 3H), 3.55 (s, 1H), 2.89 (s, 3H), 2.45 (dt, *J* = 12.6, 10.5 Hz, 1H), 2.38 (dd, *J* = 17.4, 5.4 Hz, 1H), 2.31 (td, *J* = 12.6, 5.4 Hz, 1H), 2.22 (ddd, *J* = 17.7, 12.9, 5.4 Hz, 1H), 2.16 (m, 1H), 1.45 (dd, *J* = 13.2, 5.4 Hz, 1H), 1.01 (dq, *J* = 14.4, 7.2 Hz, 1H), 0.72 (t, *J* = 7.2 Hz, 3H), 0.65 (dq, *J* = 14.4, 7.2 Hz, 1H); ^{13}C NMR (150 MHz,

$CDCl_3$) δ 171.1, 170.8, 169.8, 161.6, 154.4, 123.9, 120.7, 105.1, 103.2, 94.7, 87.2, 83.6, 64.3, 55.4, 54.4, 52.6, 51.8, 48.1, 46.8, 37.5, 35.5, 28.5, 24.6, 23.3, 8.9; IR (neat) ν_{max} 2954, 1763, 1732, 1663, 1618, 1500, 1435, 1399, 1376, 1334, 1249, 1199, 1172, 1123, 1096, 1059, 1029, 936, 910, 890, 818, 728 cm^{-1}; ESI-TOF HRMS *m/z* 471.2125 (M+H^+, $C_{25}H_{31}N_2O_7$ requires 471.2126).

Compound (–)-**8b**. A solution of (–)-**8c** (100 mg, 201 μmol) in MeOH (3 mL) was treated with MeONa (186 μL, 1.0 mmol, 5.4 M in MeOH) at room temperature. The mixture was heated at 60 °C for 24 h. Upon cooling to room temperature, dry toluene (10 mL) and $TMSCH_2N_2$ (101 μL, 201 μmol, 2 M in hexanes) were added. After striing for 30 min, the solvent was removed under vaccum. Flash chromatography (SiO_2, 40–100% EtOAc/hexanes gradient) gave (–)-**8b** (93 mg, 98%) as an off-white foam: $[\alpha]_D^{28}$ –77 (*c* 1.0, $CHCl_3$); ^1H NMR (600 MHz, $CDCl_3$) δ 6.72 (d, *J* = 8.4 Hz, 1H), 6.19 (dd, *J* = 8.4, 2.4 Hz, 1H), 6.06 (d, *J* = 2.4, Hz, 1H), 3.99 (s, 1H), 3.96–3.89 (m, 2H), 3.84 (s, 3H), 3.77 (s, 3H), 3.64 (s, 3H), 3.55 (s, 1H), 2.89 (s, 3H), 2.45 (dt, *J* = 12.6, 10.5 Hz, 1H), 2.38 (dd, *J* = 17.4, 5.4 Hz, 1H), 2.31 (td, *J* = 12.6, 5.4 Hz, 1H), 2.22 (ddd, *J* = 17.7, 12.9, 5.4 Hz, 1H), 2.16 (m, 1H), 1.45 (dd, *J* = 13.2, 5.4 Hz, 1H), 1.01 (dq, *J* = 14.4, 7.2 Hz, 1H), 0.72 (t, *J* = 7.2 Hz, 3H), 0.65 (dq, *J* = 14.4, 7.2 Hz, 1H); ^{13}C NMR (150 MHz, $CDCl_3$) δ 171.1, 170.8, 169.8, 161.6, 154.4, 123.9, 120.7, 105.1, 103.2, 94.7, 87.2, 83.6, 64.3, 55.4, 54.4, 52.6, 51.8, 48.1, 46.8, 37.5, 35.5, 28.5, 24.6, 23.3, 8.9; IR (neat) ν_{max} 2954, 1763, 1732, 1663, 1618, 1500, 1435, 1399, 1376, 1334, 1249, 1199, 1172, 1123, 1096, 1059, 1029, 936, 910, 890, 818, 728 cm^{-1}; ESI-TOF HRMS *m/z* 471.2125 (M+H^+, $C_{25}H_{31}N_2O_7$ requires 471.2126). The structure and the absolute configuration of (–)-**8b** were unambiguously established with a single crystal X-ray structure determination conducted on a colorless prism crystal obtained from vapor diffusion of hexane into a EtOAc solution of (–)-**8b** (CCDC 1475228).

(–)-**8b** ≡

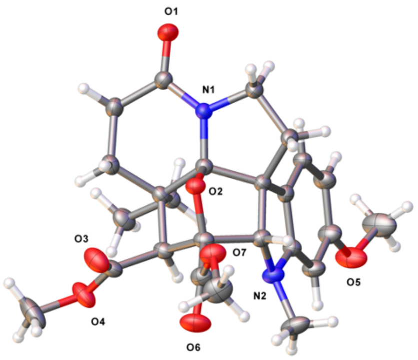

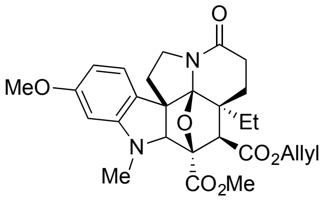

8c

Compound 8c. Ar was bubbled through a mixture of **7c** (38 mg, 0.072 mmol) in triisopropylbenzene (145 mL). The vessel was sealed and heated at 230 °C for 24 h. Upon cooling to room temperature, the reaction mixture was filtered through a plug of silica gel which was washed thoroughly with hexanes before products were eluted with

10% MeOH/CH_2Cl_2. The solvent was evaporated under reduced pressure. Flash chromatography (SiO_2, 40–70% EtOAc/hexanes gradient) provided **8c** (17.4 mg, 48%) as a pale yellow foam. Chiral HPLC (chiralcel OD, 60:40 *i*-PrOH/hexanes, 7 mL/min) allowed for separation of the enantiomers of **8c**: t_R (natural) = 15.4 min, t_R (unnatural) = 31.9 min. The scale for enantiomer separation was up to 500 mg per injection. For (+)-**8c**: $[\alpha]_D^{23}$ +96 (*c* 0.36, $CHCl_3$); ^1H NMR (600 MHz, $CDCl_3$) δ 6.72 (d, *J* = 7.8 Hz, 1H), 6.19 (dd, *J* = 8.1, 2.1 Hz, 1H), 6.07 (d, *J* = 2.4 Hz, 1H), 5.86 (ddt, *J* = 16.8, 10.5, 6.2 Hz, 1H), 5.33–5.29 (m, 1H), 5.25–5.23 (m, 1H), 4.56 (dd, *J* = 12.5, 6.1 Hz, 1H), 4.51 (dd, *J* = 12.5, 6.1 Hz, 1H), 4.00 (s, 1H), 3.96–3.91 (m, 2H), 3.83 (s, 3H), 3.78 (s, 3H), 3.56 (s, 1H), 2.89 (s, 3H), 2.46 (m, 1H), 2.41–2.31 (m, 2H), 2.25–2.20 (m, 1H), 2.22 (ddd, *J* = 17.4, 12.6, 5.4 Hz, 1H), 2.16 (ddd, *J* = 12.6, 7.2, 1.8 Hz, 1H), 1.48 (dd, *J* = 13.2, 5.4 Hz, 1H), 1.01 (p, *J* = 7.2 Hz, 1H), 0.73 (t, *J* = 7.2 Hz, 3H), 0.66 (p, *J* = 7.2 Hz, 1H); ^{13}C NMR (150 MHz, $CDCl_3$) δ 170.7, 170.3, 169.8, 161.6, 154.4, 131.4, 123.9, 120.8, 119.5, 105.2, 103.3, 94.8, 87.2, 83.7, 65.9, 64.3, 55.4, 54.5, 52.6, 48.2, 46.8, 37.5, 35.5, 28.6, 24.6, 23.3, 8.9; IR (neat) ν_{max} 2955, 1762, 1731, 1663, 1618, 1499, 1436, 1399, 1375, 1329, 1247, 1171, 1122, 1096, 1059, 1027, 910, 814, 729, 645, 598, 551 cm^{-1}; ESI-TOF HRMS *m*/*z* 497.2273 ($M+H^+$, $C_{27}H_{33}N_2O_7$ requires 497.2282).

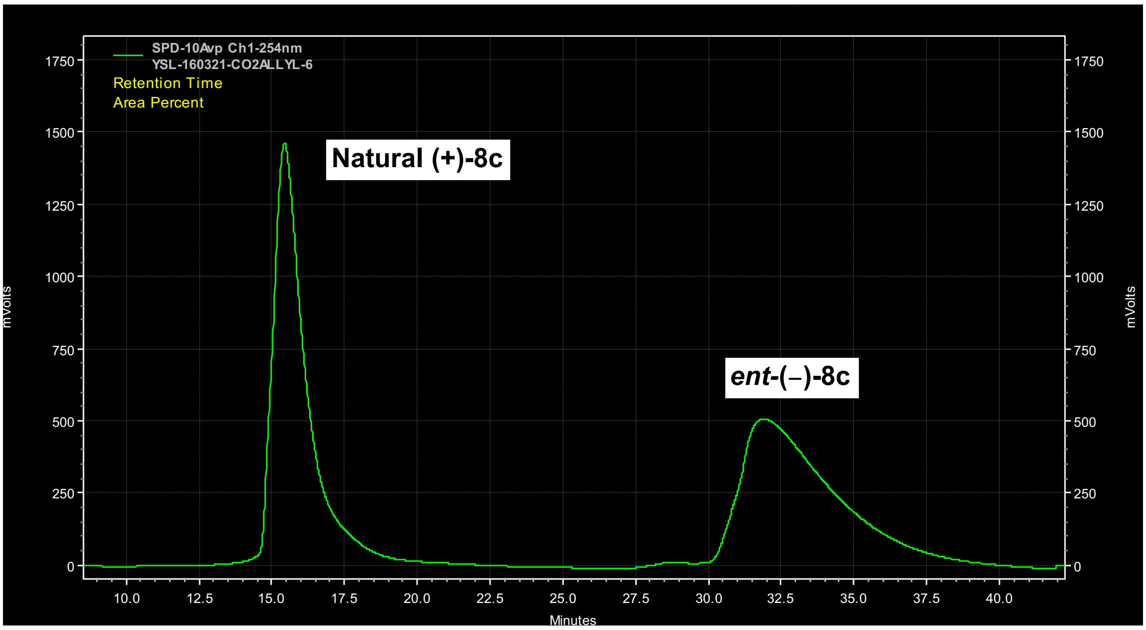

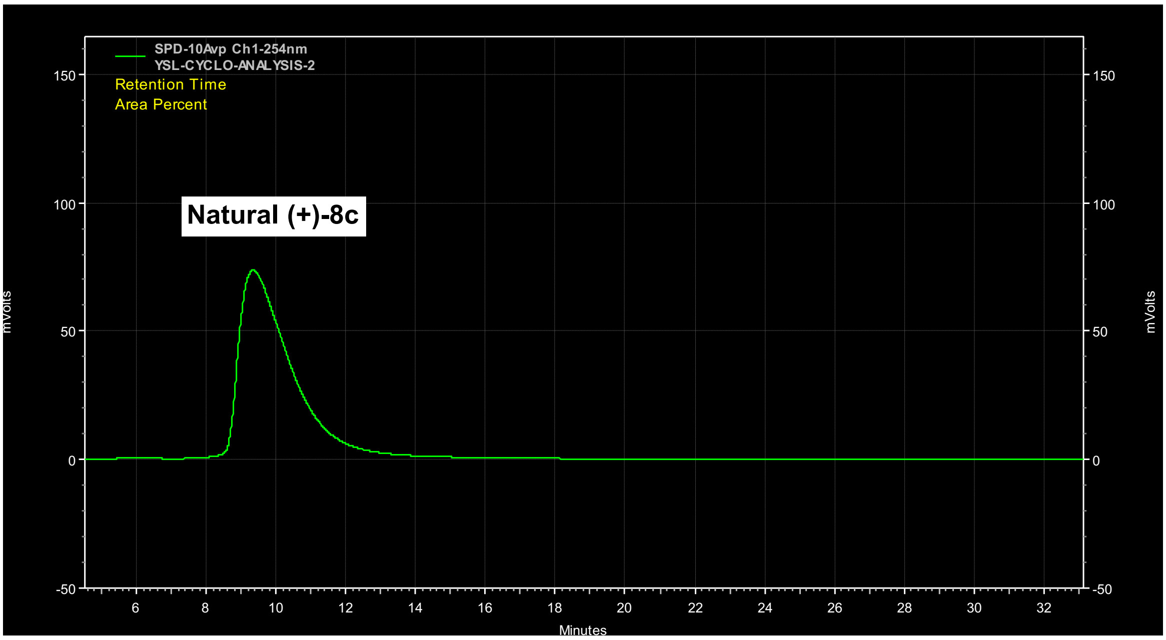

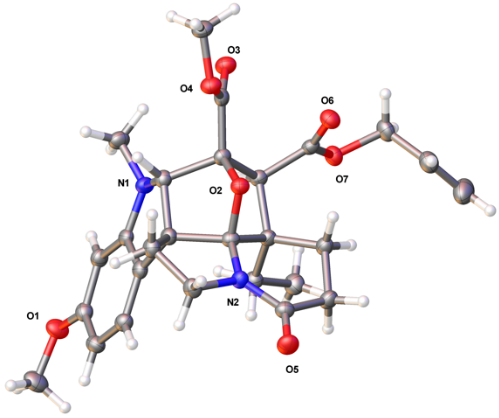

The structure and the relative stereochemistry of **8c** were unambiguously established with a single crystal X-ray structure determination conducted on a colorless prism crystal obtained from 60% *i*-PrOH/hexanes (CCDC 1475225). The absolute stereochemistry of (–)-**8c** was assigned by correlation with (–)-**8b** (CCDC 1475228) through conversion to (–)-**8b** (NaOMe, MeOH, 60 °C, 24 h; $TMSCH_2N_2$, MeOH/toluene, rt, 30 min, 98%).

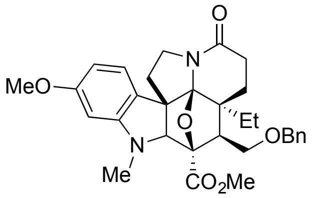

8d

Compound 8d. Ar was bubbled through a mixture of **7d** (154 mg, 0.275 mmol) in triisopropylbenzene (137 mL). The vessel was sealed and heated at 230 °C for 24 h. Upon cooling to room temperature, the reaction mixture was filtered through a plug of silica gel which was washed thoroughly with hexanes before products were eluted with

10% MeOH/CH_2Cl_2. The solvent was evaporated under reduced pressure. Flash chromatography (SiO_2, 15–50% Et_2O/CH_2Cl_2 gradient) provided **8d** (64 mg, 44%) as a pale yellow foam: ^{1}H NMR (500 MHz, $CDCl_3$) δ 7.34–7.31 (m, 2H), 7.28–7.26 (m, 3H), 6.70 (dd, *J* = 8.1, 1.7 Hz, 1H), 6.18 (d, *J* = 8.2 Hz, 1H), 6.06 (d, *J* = 2.0 Hz, 1H), 4.40, (s, 2H), 4.11 (s, 1H), 3.98–3.86 (m, 2H), 3.79 (s, 3H), 3.69 (s, 3H), 3.64–3.58 (m, 2H), 3.02 (s, 3H), 2.83 (dd, *J* = 7.7, 5.7 Hz, 1H), 2.44–2.35 (m, 3H), 2.29–2.24 (m, 1H), 2.16 (ddd, *J* = 12.5, 7.8, 1.5 Hz, 1H), 1.70 (dd, *J* = 12.3, 5.5 Hz, 1H), 1.02–0.94 (m, 1H), 0.65 (t, *J* = 7.3 Hz, 3H), 0.66–0.55 (m, 1H); ^{13}C NMR (150 MHz, $CDCl_3$) δ 170.6, 170.4, 161.7, 154.9, 137.9, 128.5, 127.7, 127.3, 123.9, 121.4, 105.4, 102.7, 94.5, 88.5, 83.1, 73.2, 67.8, 64.5, 55.5, 52.5, 48.3, 46.8, 46.3, 37.7, 35.1, 28.7, 24.2, 21.8, 9.6; IR (neat) ν_{max} 2953, 1737, 1661, 1616, 1500, 1369, 1091, 1054, 1027 cm^{-1}; ESI-TOF HRMS *m*/*z* 533.2653 (M+H$^+$, $C_{31}H_{36}N_2O_6$ requires 533.2646).

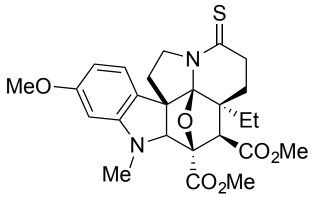

(+)-10

Compound (+)-10. A mixture of **8b** (9.3 mg, 19.8 μmol) and Lawesson's reagent (8 mg, 19.8 μmol) in toluene (0.99 mL) was heated at 110 °C for 1 h. The volatiles were removed under a stream of N_2 and the crude material purified by flash chromatography (SiO_2, 40% EtOAc/hexanes) to give **10** (9.3 mg, 96%) as a yellow solid. Chiral HPLC (chiralcel OD, 60:40 *i*-PrOH/hexanes, 7 mL/min) allowed for separation of the enantiomers of **10** (t_R [natural] = 17.2 min, t_R [unnatural] = 39.9 min). The scale for enantiomer separation was more than 500 mg per injection. For (+)-**10**: $[\alpha]_D^{25}$ +226 (*c* 0.42, $CHCl_3$); ^{1}H NMR (600 MHz, $CDCl_3$) δ 6.74 (d, *J* = 8.0 Hz, 1H), 6.20 (dd, *J* = 8.0, 2.0 Hz, 1H), 6.08 (d, *J* = 2.0 Hz, 1H), 4.32–4.26 (m, 1H), 4.21 (dd, *J* = 13.5, 9.9 Hz, 1H), 4.03 (s, 1H), 3.85 (s, 3H), 3.78 (s, 3H), 3.65 (s, 3H), 3.59 (s, 1H), 3.08 (dd, *J* = 19.8, 4.8 Hz 1H), 2.89 (s, 3H), 2.72 (ddd, *J* = 19.5, 13.2, 6.0 Hz, 1H), 2.52 (app. q, *J* = 11.2 Hz, 1H), 2.24 (m, 2H),1.40 (dd, *J* = 13.5, 5.7 Hz, 1H), 0.99 (dq, *J* = 14.1, 6.7 Hz, 1H), 0.71 (t, *J* = 6.7 Hz, 3H), 0.68 (m, 1H); ^{13}C NMR (150 MHz, $CDCl_3$) δ 201.7, 170.8, 170.4, 161.7, 154.5, 123.9, 120.2, 103.4, 102.7, 94.9, 87.7, 83.9, 65.0, 55.4, 54.5, 53.9, 52.7, 51.9, 48.4, 38.4, 37.0, 35.5, 24.5, 23.4, 8.8; IR (neat) ν_{max} 2953, 1763, 1733, 1618, 1591, 1500, 1438, 1405, 1352, 1248, 1204, 1168, 1117, 1099, 1082, 1054, 1028, 912, 866, 798, 729, 645 cm^{-1}; ESI-TOF HRMS *m*/*z* 487.1892 (M+H$^+$, $C_{25}H_{31}N_2O_6S$ requires 487.1897).

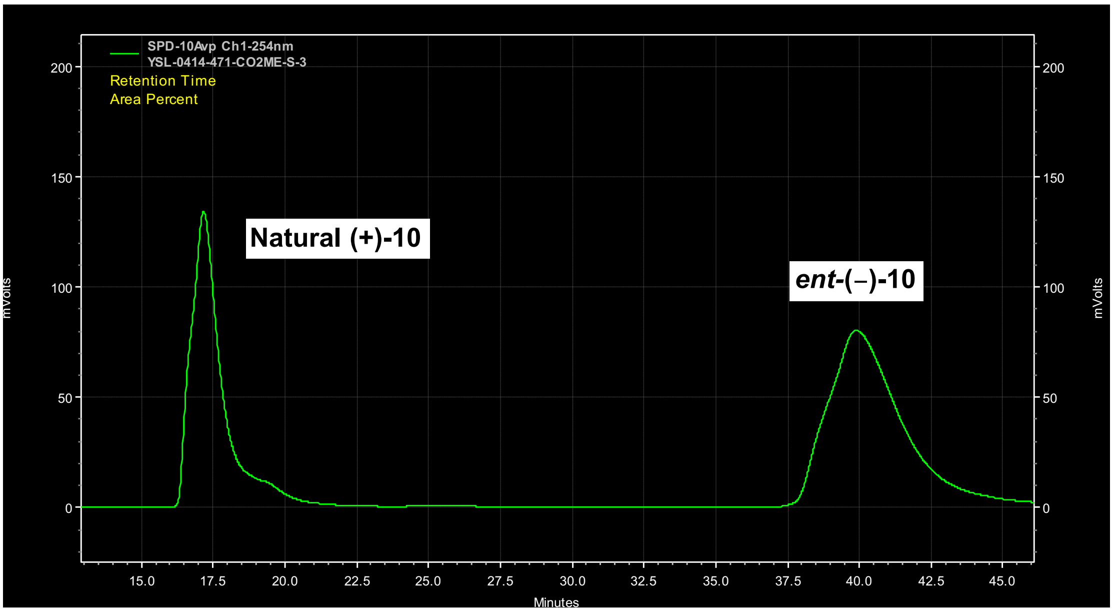

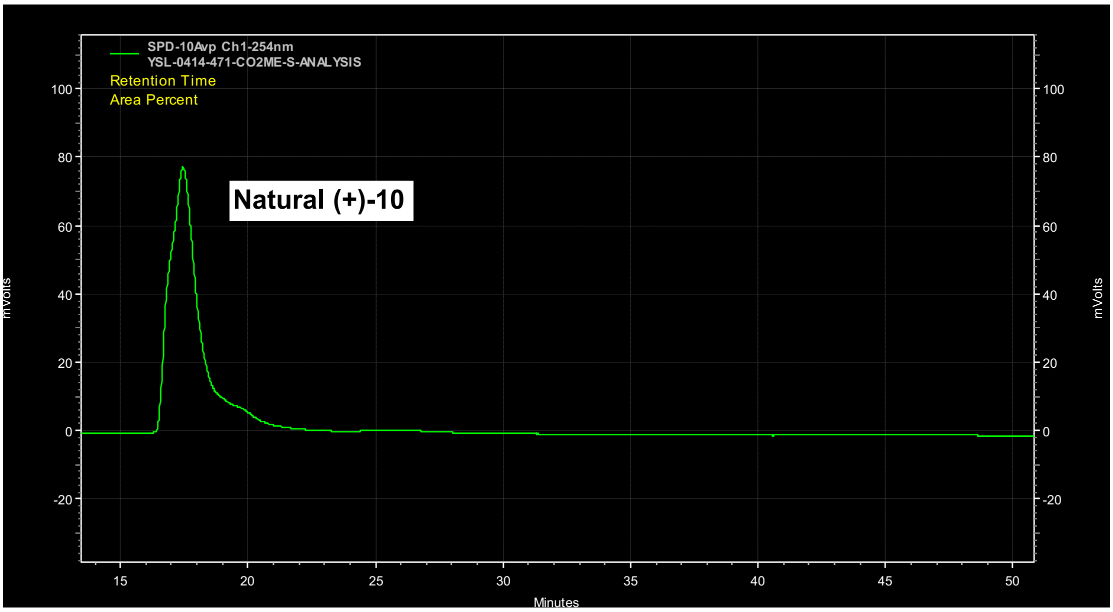

The absolute stereochemistry of (–)-**10** was assigned by conversion of (–)-**8b** to (–)-**10** through thionation (Lawesson's reagent, toluene, 110 °C, 1 h, 95%). For (–)-**10**: $[\alpha]_D^{26}$ –230 (*c* 1.0, $CHCl_3$); ^{1}H NMR (600 MHz, $CDCl_3$) δ 6.74 (d, *J* = 8.0 Hz, 1H), 6.20 (dd, *J* = 8.0, 2.0 Hz, 1H), 6.08 (d, *J* = 2.0 Hz, 1H), 4.32–4.26 (m, 1H), 4.21 (dd, *J* = 13.5, 9.9 Hz, 1H), 4.03 (s, 1H), 3.85 (s, 3H), 3.78 (s, 3H), 3.65 (s, 3H), 3.59 (s, 1H), 3.08 (dd, *J* = 19.8, 4.8 Hz 1H), 2.89 (s, 3H), 2.72 (ddd, *J* = 19.5, 13.2, 6.0 Hz, 1H), 2.52 (app. q, *J* = 11.2 Hz, 1H), 2.24 (m, 2H),1.40 (dd, *J* = 13.5, 5.7 Hz, 1H), 0.99 (dq, *J* = 14.1, 6.7 Hz, 1H), 0.71 (t, *J* = 6.7 Hz, 3H), 0.68 (m, 1H); ^{13}C NMR (150 MHz, $CDCl_3$) δ 201.7, 170.8, 170.4, 161.7, 154.5, 123.9, 120.2, 103.4, 102.7, 94.9, 87.7, 83.9, 65.0, 55.4, 54.5, 53.9, 52.7, 51.9, 48.4, 38.4, 37.0, 35.5, 24.5, 23.4, 8.8; IR (neat) ν_{max} 2953, 1763, 1733, 1618, 1591, 1500, 1438, 1405, 1352, 1248, 1204, 1168, 1117, 1099, 1082, 1054, 1028, 912, 866, 798, 729, 645 cm^{-1}; ESI-TOF HRMS *m/z* 487.1892 (M+H$^+$, $C_{25}H_{31}N_2O_6S$ requires 487.1897).

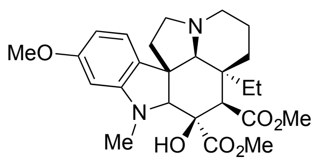

(+)-11

Compound (+)-11. Thioamide (+)-**10** (20 mg, 41.1 μmol) was passed as a solution in 9:1 THF/MeOH through a short (ca.1 cm) plug of Raney-Nickel in a Monsterpette. The eluent was concentrated to dryness under a stream of N_2 and then re-dissoved in anhydrous MeOH (0.82 mL) under Ar and cooled to 0 °C. $NaBH_4$ (15.5 mg, 0.411 mmol) was added. After 5 min, the mixture was warmed to room temperature and stirred for another 1 h. 0.1 N HCl (ca. 3 mL) was added, then the mixture was neutralized with the addition of saturated aqueous $NaHCO_3$ (ca. 5 mL) and extracted with EtOAc (3×2 mL). The combined organic extracts were washed with saturated aqueous NaCl, dried (Na_2SO_4) and concentrated under reduced pressure. PTLC (SiO_2, 90:10:3 EtOAc/hexanes/Et_3N) gave (+)-**11** (13.7 mg, 73%) as a clear, colorless oil: $[\alpha]_D^{24}$ +66 (*c* 0.89, $CHCl_3$); ^{1}H NMR (600 MHz, $CDCl_3$) δ 10.09 (br s, 1H), 6.86 (d, *J* = 8.0 Hz, 1H), 6.25 (dd, *J* = 8.0, 2.0 Hz, 1H), 6.00 (d, *J* = 2.0 Hz, 1H), 3.77 (s, 3H), 3.73 (s, 3H), 3.65 (s, 3H), 3.61 (s, 1H), 3.35 (s, 1H), 3.20–3.15 (m, 1H), 3.04 (m, 1H), 2.58 (s, 3H), 2.39–2.30 (m, 2H), 2.28–2.22 (m, 1H), 2.16 (s, 1H), 1.99–1.89 (m, 2H), 1.50–1.47 (m, 2H), 1.39 (dq, *J* = 14.4, 7.2 Hz, 1H), 1.26–1.21 (m, 1H), 0.85 (dq, *J* = 14.4, 7.2 Hz, 1H), 0.59 (t, *J* = 7.2 Hz, 3H); ^{13}C NMR (150 MHz, $CDCl_3$) δ 174.1, 173.0, 160.9, 154.1, 126.3, 122.9, 104.2, 95.7, 83.9, 78.2, 71.4, 55.3, 53.3, 52.3, 52.1, 51.5, 51.1, 51.0, 44.2, 38.23, 38.16, 36.6, 31.3, 21.8, 8.2; IR (neat) ν_{max} 2946, 1733, 1615, 1501, 1452, 1434, 1355, 1331, 1246, 1226, 1167, 1088, 1042, 972, 907, 799, 750, 665 cm^{-1}; ESI-TOF HRMS *m/z* 459.2486 (M+H$^+$, $C_{25}H_{31}N_2O_6S$ requires 459.2490).

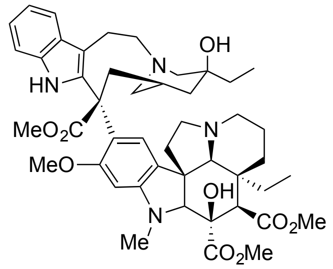

12

Compound 12. (+)-**11** (6 mg, 13.1 μmol) and catharanthine sulfate (5 mg, 6.54 μmol) were combined with TFE (60 μL), aqueous 0.1 N HCl (0.30 mL) and water (0.30 mL) in a 2-dram vial. The vial was stoppered and flushed with Ar. $FeCl_3·6H_2O$ (17.7 mg, 65.5 μmol) was added producing a bright yellow precipitate. The mixture was stirred for 2 h during which time the precipitate re-dissolved leaving a clear, yellow solution. Meanwhile, a suspension of $Fe_2(ox)_3·6H_2O$ (63.4 mg, 131 μmol) in water (54.6 mL) was stirred for 2 h, by which time the $Fe_2(ox)_3·6H_2O$ was completely dissolved. This mixture was cooled to 0 °C and air was bubbled vigorously through the solution for 15–20 min. The $FeCl_3$ coupling mixture was transferred to the $Fe_2(ox)_3/O_2$ solution by pipette, then $NaBH_4$ (9.9 mg, 262 μmol) was added as a solution in water (1 mL). After stirring for 30 min, the reaction was quenched by the addition of aqueous NH_4OH (16

mL) and the resulting mixture extracted with 10% MeOH/CH_2Cl_2 (4×50 mL). The combined organic extracts were dried (Na_2SO_4) and concentrated under reduced pressure. PTLC (SiO_2, 97:3:3 EtOAc/MeOH/Et_3N) gave **12** (3.9 mg, 37%) as an off-white film, along with the corresponding leurosidine analogue (2.2 mg, 21%) and anhydrovinblastine analogue (1.7 mg, 16%) also as off-white films. For **12**: $[\alpha]_D^{25}$ +74 (*c* 0.27, $CHCl_3$); ^{1}H NMR (600 MHz, $CDCl_3$) δ10.39 (br s, 1H), 8.06 (br s, 1H), 7.48 (d, *J* = 7.8 Hz, 1H), 7.19 (t, *J* = 7.5 Hz, 1H), 7.11 (m, 2H), 6.47 (br s, 1H), 6.04 (s, 1H), 3.90 (t, *J* = 14.1 Hz, 1H), 3.79 (s, 3H), 3.75 (s, 3H), 3.69 (s, 3H), 3.72–3.65 (m, 1H), 3.58 (s, 3H), 3.65–3.50 (m, 2H), 3.29 (s, 1H), 3.20 (br m, 1H), 3.03 (m, 1H), 2.92 (d, *J* = 8.4 Hz, 2H), 2.31 (m, 2H), 2.20 (s, 1H), 2.18–2.13 (m, 2H), 1.98–1.90 (m, 3H), 1.80 (t, *J* = 12.0 Hz, 1H), 1.68 (dq, *J* = 14.4, 7.2Hz, 1H), 1.55–1.30 (m, 7H), 1.09 (dq, *J* = 13.2, 7.2 Hz, 1H), 0.92 (t, *J* = 6.6 Hz, 3H), 0.88 (t, *J* = 7.5 Hz, 3H); ^{13}C NMR (150 MHz, $CDCl_3$) δ 174.1, 173.0, 157.8, 153.2, 134.9, 131.1, 129.0, 124.2, 123.6, 122.8, 119.3, 118.3, 110.2, 94.0, 84.0, 77.9, 68.4, 55.9, 55.8, 53.8, 52.5, 52.4, 52.1, 51.2, 50.8, 50.7, 44.6, 38.32, 38.23, 35.6, 34.5, 30.5, 21.6, 9.2, 6.8; IR (neat) ν_{max} 2948, 1735, 1617, 1503, 1434, 1362, 1332, 1301, 1227, 1174, 1042, 1004 cm^{-1}; ESI-TOF HRMS *m*/*z* 813.4418 (M+H$^+$, $C_{46}H_{61}N_4O_9$ requires 813.4433).

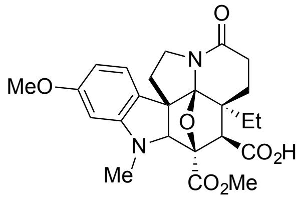

(+)-13

Compound (+)-13. Morpholine (44.6 mg, 0.51 mmol) and Pd(PPh_3)$_4$ (5.9 mg, 5.12 μmol) were added to a mixture of (+)-**8c** (25.4 mg, 51.2 μmol) in THF (0.93 mL) and DMSO (90 μL). After stirring for 1 h, the mixture was diluted with EtOAc (3 mL) and washed with aqueous 2 N HCl (3 × 1 mL), then saturated aqueous NaCl. The combined aqueous layers were re-extacted with EtOAc and the combined EtOAc extracts were dried (Na_2SO_4) and concentrated. PTLC (SiO_2, 1% AcOH/EtOAc) gave (+)-**13** (18.5 mg, 79%) as a clear, colorless oil: $[\alpha]_D^{26}$ +86 (*c* 0.41, $CHCl_3$); ^{1}H NMR (600 MHz, $CDCl_3$) δ 6.72 (d, *J* = 8.3 Hz, 1H), 6.20 (dd, *J* = 8.3, 2.3 Hz, 1H), 6.07 (d, *J* = 2.3 Hz, 1H), 3.99 (s, 1H), 3.97–3.88 (m, 2H), 3.81 (s, 3H), 3.77 (s, 3H), 3.56 (s, 1H), 2.89 (s, 3H), 2.48–2.32 (m, 3H), 2.25 (ddd, *J* = 17.4, 12.6, 5.4 Hz, 1H), 2.17 (m, 1H), 1.61 (dd, *J* = 12.9, 5.7 Hz, 1H), 1.01 (dq, *J* = 14.4, 7.2 Hz, 1H), 0.74 (t, *J* = 7.2 Hz, 3H), 0.66 (dq, *J* = 14.4, 7.2 Hz, 1H); ^{13}C NMR (150 MHz, $CDCl_3$) δ 175.0, 170.6 , 170.3, 161.6, 154.4, 123.9, 120.6, 105.0, 103.4, 94.8, 87.2, 83.7, 64.4, 55.4, 54.2, 52.6, 48.1, 46.8, 37.4, 35.6, 28.4, 24.6, 23.2, 8.9; IR (neat) ν_{max} 3372, 2955, 1760, 1724, 1619, 1461, 1438, 1406, 1371, 1329, 1247, 1200, 1173, 1125, 1098, 1060, 1027, 968, 938, 896, 823 cm^{-1}; ESI-TOF HRMS *m*/*z* 457.1976 (M+H$^+$, $C_{24}H_{28}N_2O_7$ requires 457.1969).

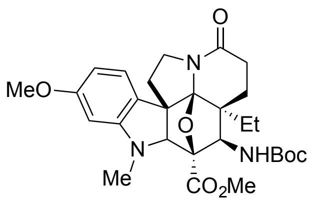

(+)-14

Compound (+)-14. Et_3N (4.9 mg, 48.2 μmol) and DPPA (12.1 mg, 43.8 μmol) were added to a mixture of (+)-**13** (10 mg, 21.9 μmol) in *t*-BuOH (2.2 mL, distilled from CaH_2 and stored over 4Å MS). The resulting mixture was heated at 85 °C for 16 h. Upon cooling to room temperature, the volatiles were evaporated under a stream of N_2 and the residue partitioned between EtOAc and aqueous 2 N HCl. The aqueous layer was extracted twice with EtOAc, the combined organic extracts were washed with saturated aqueous $NaHCO_3$ and saturated aqueous NaCl then dried (Na_2SO_4) and concentrated under reduced pressure. PTLC (SiO_2, 2% MeOH/EtOAc) gave (+)-**14** (9.1 mg, 79%) as a white film: $[\alpha]_D^{25}$ +72 (*c* 0.85, $CHCl_3$); ^1H NMR (600 MHz, $CDCl_3$) δ 6.67 (d, *J* = 8.0 Hz, 1H), 6.14 (dd, *J* = 8.0, 1.9 Hz, 1H), 6.06 (d, *J* = 1.9 Hz, 1H), 4.71 (m, 2H), 4.09 (s, 1H), 3.93 (dt, *J* = 11.4, 8.4 Hz, 1H), 3.87 (app. t, *J* = 11.1 Hz, 1H), 3.82 (s, 3H), 3.76 (s, 3H), 3.02 (s, 3H), 2.44–2.35 (m, 2H), (2.27 (ddd, *J* = 18.6, 13.2, 5.4 Hz, 1H), 2.14 (dd, *J* = 12.3, 7.5 Hz, 1H), 2.03 (dt, *J* = 6.0, 5.7 Hz, 1H), 1.73 (dd, *J* = 13.2, 4.8 Hz, 1H), 1.37 (s, 9H), 0.92–0.87 (m, 1H), 0.61–0.56 (m, 4H); ^{13}C NMR (150 MHz, $CDCl_3$) δ 169.7, 169.0, 161.6, 154.5, 154.3, 123.5, 120.3, 104.7, 103.2, 94.3, 91.2, 81.1, 79.9, 64.2, 58.6, 55.3, 52.4, 47.9, 46.8, 37.1, 35.2, 28.23, 28.18, 22.6, 21.9, 8.9; IR (neat) ν_{max} 2972, 1744, 1712, 1662, 1618, 1501, 1459, 1434, 1400, 1366, 1320, 1245, 1201, 1165, 1122, 1095, 1054, 1032, 1008, 892, 872, 818, 730 cm^{-1}; ESI-TOF HRMS *m/z* 528.2700 ($M+H^+$, $C_{28}H_{37}N_3O_7$ requires 528.2704).

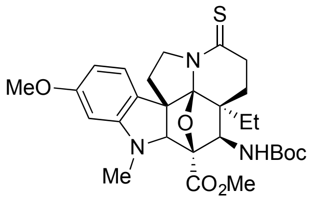

(+)-15

Compound (+)-15. A mixture of (+)-**14** (22 mg, 41.7 μmol) and Lawesson's reagent (16.9 mg, 41.7 μmol) in toluene (2.1 mL) was heated at 110 °C for 1 h. The volatiles were removed under a stream of N_2 and the crude material purifed directly by PTLC (SiO_2, 40% EtOAc/hexanes) to give (+)-**15** (18.8 mg, 83%) as a cloudy white film: $[\alpha]_D^{23}$ +184 (*c* 0.69, $CHCl_3$); ^1H NMR (600 MHz, $CDCl_3$) δ 6.70 (d, *J* = 8.3 Hz, 1H), 6.17 (dd, *J* = 8.3, 2.3 Hz, 1H), 6.08 (d, *J* = 2.3 Hz, 1H), 4.75 (d, *J* = 11.4 Hz, 1H), 4.71 (d, *J* = 11.4 Hz, 1H), 4.32–4.26 (m, 1H), 4.18 (dd, *J* = 13.8, 10.2 Hz, 1H), 4.13 (s, 1H), 3.83 (s, 3H), 3.77 (s, 3H), 3.13 (dd, *J* = 19.8, 4.2 Hz, 1H), 3.04 (s, 3H), 2.77 (ddd, *J* = 19.2, 13.2, 5.9 Hz, 1H), 2.46 (app. q, *J* = 11.2 Hz, 1H), 2.23 (dd, *J* = 12.6, 7.2 Hz, 1H), 1.96 (dt, *J* = 12.9, 5.2 Hz, 1H), 1.69 (dd, *J* = 13.2, 4.2 Hz, 1H), 1.38 (s, 9H), 0.91–0.86 (m, 1H), 0.62–0.57 (m, 4H); ^{13}C NMR (150 MHz, $CDCl_3$) δ 201.5, 168.7, 161.8, 154.6, 154.3, 123.6, 119.7, 103.4, 102.4, 94.5, 91.8, 81.3, 80.1, 64.9, 58.5, 55.4, 53.9, 52.5, 48.3, 38.1, 36.7, 35.2, 28.3, 22.6, 22.0, 9.0; IR (neat) ν_{max} 2974, 1745, 1702, 1618, 1592,

1501, 1440, 1405, 1365, 1350, 1321, 1298, 1246, 1206, 1160, 1111, 1077, 1051, 1007, 983, 968, 956, 913, 875, 831, 729 cm^{-1}; ESI-TOF HRMS *m*/*z* 544.2480 ($M+H^+$, $C_{28}H_{38}N_3O_6S$ requires 544.2476).

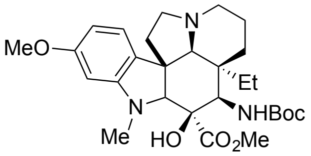

(+)-16

Compound (+)-16. (+)-**15** (10.8 mg, 19.9 μmol) was filtered through a 1 cm plug of Raney-Nickel in a Monsterpette as a solution in 1:1 THF/MeOH. The solvent was evaporated under a stream of N_2 and the residue dissolved in anhydrous MeOH (0.4 mL) and cooled to 0 °C. $NaBH_4$ (7.5 mg, 0.199 mmol) was added and the resulting mixture was stirred at 0 °C for 1 h. Saturated aqueous NH_4Cl (ca. 3 mL) was added and the mixture extracted with EtOAc (3 × ca.1 mL). The combined organic extracts were washed with saturated aqueous NaCl, dried (Na_2SO_4) and concentrated under a stream of N_2. PTLC (SiO_2, 3% Et_3N/EtOAc) gave (+)-**16** (3.8 mg, 37%) as a colorless film: $[\alpha]_D^{25}$ +31 (*c* 0.39, $CHCl_3$); ^{1}H NMR (600 MHz, $CDCl_3$) δ 9.76 (br s, 1H), 6.86 (d, *J* = 8.0 Hz, 1H), 6.27 (dd, *J* = 8.0, 2.3 Hz, 1H), 6.01 (d, *J* = 2.3 Hz, 1H), 5.28 (d, *J* = 9.6 Hz, 1H), 4.36 (d, *J* = 9.6 Hz, 1H), 3.77 (s, 3H), 3.74 (s, 3H), 3.59 (s, 1H), 3.16 (m, 1H), 3.05 (m, 1H), 2.59 (s, 3H), 2.35–2.26 (m, 3H), 2.06 (s, 1H), 1.94 (dt, *J* = 11.1, 3.8 Hz, 1H), 1.67–1.58 (m, 3H), 1.40–1.34 (m, 10H), 1.07 (dt, *J* = 13.2, 5.2 Hz, 1H), 0.98 (dq, *J* = 14.1, 7.2 Hz, 1H), 0.53 (t, *J* = 7.2 Hz, 3H); With minor (~15%) NHBoc rotamer peaks observed at: δ 6.09 (d, *J* = 2.2 Hz, 1H), 5.12 (d, *J* = 9.5 Hz, 1H), 4.25 (d, *J* = 9.4 Hz, 1H), 3.80 (s, 3H), 3.77 (s, 3H), 3.60 (s, 1H), 2.62 (s, 3H), 2.06 (s, 1H), 1.49 (s, 9H), 0.47 (t, *J* = 7.4 Hz, 3H). ^{13}C NMR (150 MHz, $CDCl_3$) δ 173.3, 160.9, 155.9, 154.6, 126.8, 123.0, 104.3, 96.0, 83.9, 79.5, 78.7, 73.6, 55.4, 55.2, 53.0, 52.6, 52.4, 52.0, 44.2, 40.2, 38.8, 34.9, 30.2, 28.4, 23.5, 8.08; With minor (~15%) NHBoc rotamer peaks observed at: δ 172.8, 160.9, 156.0, 154.6, 127.0, 123.1, 104.4, 104.3, 96.4, 83.8, 79.9, 79.2, 72.9, 56.6, 55.4, 53.2, 52.4, 52.2, 44.3, 39.0, 34.2, 30.1, 29.7, 28.5, 23.5, 8.3. IR (neat) ν_{max} 2931, 1741, 1716, 1615, 1495, 1390, 1364, 1323, 1302, 1227, 1169, 1114, 1088, 1063, 1040, 1000, 969, 889, 821, 797, 734, 669, 648 cm^{-1}; ESI-TOF HRMS *m*/*z* 516.3074 ($M+H^+$, $C_{28}H_{41}N_3O_6$ requires 516.3068).

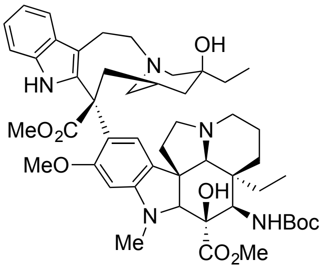

17

Compound (+)-17. (+)-**16** (6.4 mg, 12.4 μmol) and catharanthine sulfate (4.8 mg, 6.21 μmol) were combined with TFE (57 μL), aqueous 0.1 N HCl (0.29 mL) and water (0.29 mL) in a 2-dram vial. The vial was fitted with a rubber septum and flushed with Ar. $FeCl_3 \cdot 6H_2O$ (16.8 mg, 62.0 μmol) was added producing a bright yellow precipitate. The

mixture was stirred for 2 h during which time the precipitate re-dissolved leaving a clear, yellow solution. Meanwhile, a suspension of $Fe_2(ox)_3{\cdot}6H_2O$ (60 mg, 124 μmol) in water (52 mL) was stirred for 2 h, by which time the $Fe_2(ox)_3{\cdot}6H_2O$ was completely dissolved. This mixture was cooled to 0 °C and air was bubbled vigorously through the solution for 15–20 min. The $FeCl_3$ coupling mixture was transferred to the $Fe_2(ox)_3/O_2$ solution by pipette, then $NaBH_4$ (9.4 mg, 248 μmol) was added as a solution in water (1 mL). After stirring for 30 min, the reaction was quenched by the addition of aqueous NH_4OH (16 mL) and the resulting mixture extracted with 10% $MeOH/CH_2Cl_2$ (4 × 50 mL). The combined organic extracts were dried (Na_2SO_4) and concentrated under reduced pressure. PTLC (SiO_2, 97:3:3 EtOAc/MeOH/Et_3N) gave (+)-**17** (3.3 mg, 31%) as a white film, along with the corresponding leurosidine analogue (2.2 mg, 20%) and anhydrovinblastine analogue (1.1 mg, 10%) also as white films. For (+)-**17**: $[\alpha]_D^{26}$ +36 (*c* 0.21, $CHCl_3$); ^1H NMR (600 MHz, $CDCl_3$) δ 10.0 (br s, 1H), 8.04 (br s, 1H), 7.50 (d, *J* = 7.8 Hz, 1H), 7.17 (t, *J* = 7.5 Hz, 1H), 7.13–7.07 (m, 2H), 6.54 (br s, 1H), 6.04 (s, 1H), 5.27 (d, *J* = 9.6 Hz, 1H), 4.33 (d, *J* = 9.6 Hz, 1H), 3.91 (t, *J* = 14.1 Hz, 1H), 3.78 (s, 3H), 3.78 (m, 1H), 3.75 (s, 3H), 3.60–3.55 (m, 5H), 3.45–3.25 (br m, 2H), 3.17–3.07 (br m, 2H), 3.01 (dt, *J* = 9.3, 3.7 Hz, 1H), 2.93 (d, *J* = 10.2 Hz, 1H), 2.81 (br s, 2H), 2.64 (s, 3H), 2.46–2.38 (br s, 1H), 2.28 (d, *J* = 14.4 Hz, 1H), 2.21 (q, *J* = 9.3 Hz, 1H), 2.16–2.10 (m, 1H), 2.08 (s, 1H), 1.90 (dt, *J* = 11.0, 4.0 Hz, 1H), 1.84 (m, 1H), 1.77–1.55 (m, 7H), 1.41 (s, 9H), 1.35–1.28 (br m, 2H), 1.25–1.20 (m, 1H), 1.16 (dt, *J* = 13.4, 5.2 Hz, 1H), 0.88 (t, *J* = 7.5 Hz, 3H), 0.83 (t, *J* = 7.5 Hz, 3H); With minor (~10%) NHBoc rotamer peaks observed at: δ 9.89 (s, 1H), 6.13 (s, 1H), 5.11 (d, *J* = 9.2 Hz, 1H), 4.23 (d, *J* = 9.2 Hz, 1H), 1.52 (s, 9H). IR (neat) ν_{max} 2944, 1720, 1616, 1495, 1459, 1434, 1391, 1365, 1332, 1305, 1227, 1171, 1111, 1092, 1061, 1011, 739 cm^{-1}; ESI-TOF HRMS *m/z* 870.5009 ($M+H^+$, $C_{49}H_{68}N_5O_9$ requires 870.5011).

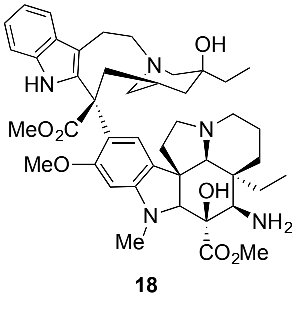

18

Compound (+)-18. (+)-**17** (1.2 mg, 1.38 μmol) was dissolved in 4:1 CH_2Cl_2/TFA (280 μL) and the mixture was stirred for 2 h, then diluted with saturated aqueous $NaHCO_3$ (3 mL). The mixture was extracted with 10% $MeOH/CH_2Cl_2$ (3 × 2 mL). The combined organic extracts were dried (Na_2SO_4) and concentrated under a stream of N_2. The reaction was repeated using 1.1 mg of (+)-**17** (2.3 mg total) and the combined crude products were purified by PTLC (SiO_2, 94:3:3 EtOAc/MeOH/Et_3N) to give (+)-**18** (1.23 mg, 60%) as a white film: $[\alpha]_D^{26}$ +75 (*c* 0.10, $CHCl_3$); ^1H NMR (600 MHz, $CDCl_3$) δ 9.84 (br s, 1H), 7.96 (br s, 1H), 7.50 (d, *J* = 7.8 Hz, 1H), 7.18 (t, *J* = 6.9 Hz, 1H), 7.13 (d, *J* = 7.8 Hz, 1H), 7.10 (t, *J* = 7.5 Hz, 1H), 6.52 (br s, 1H), 6.08 (s, 1H), 3.93 (t, *J* = 14.4 Hz, 1H), 3.84 (s, 3H), 3.76 (s, 3H), 3.64 (s, 1H), 3.56 (s, 3H), 3.38 (s, 1H), 3.13 (br s, 1H), 3.05 (dt, *J* = 9.0, 3.2 Hz, 1H), 2.94 (d, *J* = 10.8 Hz, 1H), 2.81 (br s, 1H), 2.67

(s, 3H), 2.44 (d, J = 14.4 Hz, 1H), 2.30–2.20 (m, 1H), 2.10 (d, J = 13.2, 8.6 Hz, 1H), 2.04 (s, 1H), 1.99–1.94 (m, 1H), 1.78 (m, 1H), 1.70 (br s, 1H), 1.35–1.25 (m, 2H), 0.95–0.92 (m, 3H), 0.89–0.86 (m, 4H); IR (neat) ν_{max} 3468, 2932, 1731, 1616, 1503, 1459, 1432, 1363, 1331, 1307, 1228, 1174, 1136, 1061, 1038, 1006, 908, 878, 856, 812, 732, 670 cm^{-1}; ESI-TOF HRMS m/z 770.4468 (M+H$^+$, $C_{44}H_{60}N_5O_7$ requires 770.4487).

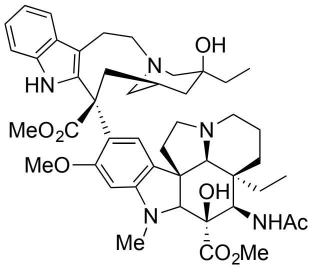

19

Compound (+)-19. (+)-**18** (0.9 mg, 1.17 μmol) was dissolved in 100 μL of anhydrous pyridine under Ar. Acetic anhydride (100 μL) was added and the resulting mixture stirred for 30 min at which time volatiles were evaporated under a stream of N_2. The residue was re-dissolved in toluene/CH_2Cl_2 and concentrated once more to give a mixture of mono- and diacetylated products. The residue was then dissolved in MeOH (0.2 mL) and K_2CO_3 (1.62 mg, 11.7 μmol, delivered in 44 μL H_2O) was added. After stirring for 1 h, the mixture was diluted with water and saturated aqueous NaCl and then extracted with 10% MeOH/CH_2Cl_2 (3 × ca.1 mL) and with EtOAc (3 × ca.1 mL). The combined organic extracts were washed with saturated aqueous NaCl. The combined aqueous layers were re-extracted with EtOAc. The combined organic extracts were washed once more with saturated aqueous NaCl, then dried (Na_2SO_4) and concentrated under a stream of N_2. PTLC (SiO_2, 94:3:3 EtOAc/MeOH/Et_3N) gave (+)-**19** (0.64 mg, 67%) as a colorless film: $[\alpha]_D^{23}$ +68 (c 0.05, $CHCl_3$); ^1H NMR (600 MHz, $CDCl_3$) δ 10.17 (br s, 1H), 8.04 (br s, 1H), 7.48 (br s, 1H), 7.18 (br s, 1H), 7.12 (m, 2H), 6.57 (br s, 1H), 6.14 (d, J = 9.0 Hz, 1H), 6.07 (s, 1H), 4.64 (d, J = 9.6 Hz), 3.90 (m, 1H), 3.78 (s, 3H), 3.77 (s, 3H), 3.60 (s, 1H), 3.58 (s, 3H), 3.03 (dt, J = 9.0, 1.8 Hz, 1H), 2.95 (d, J = 11.4 Hz, 1H), 2.64 (s, 3H), 2.23 (q, J = 9.0 Hz, 1H), 2.14 (m, 1H), 2.08 (br s, 1H), 1.96 (s, 3H), 1.94–1.83 (m, 2H), 1.90 (dt, J = 8.4, 4.0 Hz, 1H), 0.89 (br m, 3H), 0.82 (t, J = 7.2 Hz, 3H); IR (neat) ν_{max} 2950, 1738, 1664, 1617, 1502, 1459, 1434, 1366, 1323, 1306, 1227, 1178, 1150, 1130, 1091, 1062, 1040, 1003, 920, 821, 732, 671 cm^{-1}; ESI-TOF HRMS m/z 812.4593 (M+H$^+$, $C_{46}H_{61}N_5O_8$ requires 812.4593).

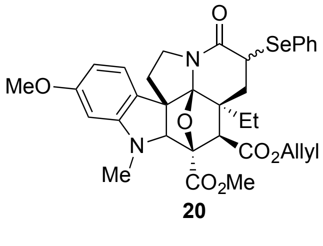

20

Compound (+)-20. A solution of **8c** (140 mg, 0.28 mmol) in THF (3 mL) was treated with LiHMDS (0.56 mL, 1.0 M in THF) at –78 °C. After stirring at the same temperature for 30 min, PhSeCl was added in one portion followed by a second portion of LiHMDS. After stirring at –78 °C for another 1 h, the reaction was quenched by

addition of saturated aqueous $NaHCO_3$. The reaction mixture was extracted with EtOAc (3×5 mL). The combined organic extracts were washed with saturated aqueous NaCl, dried (Na_2SO_4) and concentrated under reduced pressure. Flash chromatography (SiO_2, 33% EtOAc/hexanes) provided (+)-**20** as a mixture of two diastereomers (**20a**/**20b** = 3:1, 156 mg, 85%) as light yellow oil. For (+)-**20a**: $[\alpha]_D^{25}$ +138 (*c* 1.0, $CHCl_3$); ^1H NMR (600 MHz, $CDCl_3$) δ 7.67–7.61 (m, 2H), 7.31–7.22 (m, 3H), 6.72 (d, *J* = 8.2 Hz, 1H), 6.19 (dd, *J* = 8.2, 2.2 Hz, 1H), 6.05 (d, *J* = 2.2 Hz, 1H), 5.71 (ddt, *J* = 17.2, 10.3, 6.1 Hz, 1H), 5.26 (dd, *J* = 17.1, 1.4 Hz, 1H), 5.20 (dd, *J* = 10.4, 1.2 Hz, 1H), 4.41–4.35 (m, 2H), 4.06–3.97 (m, 2H), 3.97–3.89 (m, 2H), 3.83 (s, 3H), 3.78 (s, 3H), 3.49 (s, 1H), 2.87 (s, 3H), 2.58–2.46 (m, 2H), 2.18 (ddd, *J* = 12.6, 7.8, 1.4 Hz, 1H), 1.55 (dd, *J* = 13.5, 5.7 Hz, 1H), 1.02–0.91 (m, 1H), 0.64–0.58 (m, 1H), 0.58–0.53 (m, 3H); ^{13}C NMR (151 MHz, $CDCl_3$) δ 170.1, 169.4, 168.7, 161.2, 153.9, 135.3, 130.8, 128.5, 128.0, 127.8, 123.4, 120.1, 119.0, 104.7, 102.9, 94.3, 86.7, 83.2, 65.4, 63.8, 54.9, 54.2, 52.1, 48.7, 46.9, 38.1, 37.2, 35.1, 31.7, 24.5, 8.3; IR (neat) ν_{max} 2953, 1731, 1660, 1619, 1499, 1400, 1374, 1246, 1173, 1124, 1100, 750 cm^{-1}; ESI-TOF HRMS *m*/*z* 653.1763 ($M+H^+$, $C_{33}H_{36}N_2O_7Se$ requires 653.1760). For (+)-**20b**: $[\alpha]_D^{26}$ +122 (*c* 0.35, $CHCl_3$); ^1H NMR (600 MHz, $CDCl_3$) δ 7.75–7.69 (m, 2H), 7.31–7.27 (m, 3H), 6.82 (d, *J* = 8.2 Hz, 1H), 6.26 (dd, *J* = 8.2, 2.2 Hz, 1H), 6.10 (d, *J* = 2.2 Hz, 1H), 5.80 (ddt, *J* = 16.6, 10.3, 6.1 Hz, 1H), 5.29 (dd, *J* = 17.2, 1.4 Hz, 1H), 5.21 (dd, *J* = 10.4, 1.2 Hz, 1H), 4.57–4.51 (m, 1H), 4.51–4.45 (m, 1H), 4.08–3.97 (m, 4H), 3.83 (s, 3H), 3.81 (s, 3H), 3.54 (s, 1H), 2.95 (ddd, *J* = 15.2, 7.9, 1.8 Hz, 1H), 2.90 (s, 3H), 2.53–2.46 (m, 1H), 2.28–2.15 (m, 2H), 2.05 (dd, *J* = 15.0, 1.3 Hz, 1H), 1.18 (dt, *J* = 15.8, 7.1 Hz, 1H), 0.92 (t, *J* = 7.2 Hz, 3H), 0.89–0.81 (m, 1H); ^{13}C NMR (151 MHz, $CDCl_3$) δ 170.6, 170.2, 169.9, 161.7, 154.4, 133.6, 131.5, 131.3, 129.1, 127.7, 123.9, 120.8, 119.5, 105.1, 103.5, 95.0, 87.2, 83.8, 66.0, 65.0, 55.4, 54.7, 52.6, 49.0, 47.6, 39.3, 37.8, 35.7, 30.9, 25.6, 10.1; IR (neat) ν_{max} 2953, 1731, 1663, 1619, 1499, 1402, 1376, 1248, 1174, 1126, 1078, 1025, 752 cm^{-1}; ESI-TOF HRMS *m*/*z* 653.1763 ($M+H^+$, $C_{33}H_{36}N_2O_7Se$ requires 653.1760).

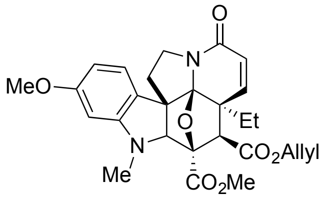

(+)-21

Compound (+)-21. A solution of (+)-**20** (74 mg, 0.11 mmol) in THF (3.5 mL) was treated with aqueous H_2O_2 solution (28 μL, 35% w/w, 0.34 mmol) at 0 °C, and the reaction mixture was stirred at the same temperature until the consumption of starting material by TLC. The reaction was quenched by addition of saturated aqueous $Na_2S_2O_3$ and the mixture was extracted with EtOAc. The combined organic extracts were washed with saturated aqueous NaCl, dried (Na_2SO_4) and concentrated under reduced pressure. Flash chromatography (SiO_2, 50% EtOAc/hexanes) provided (+)-**21** (48.1 mg, 86%) as a colorless oil: $[\alpha]_D^{25}$ +110 (*c* 1.0, $CHCl_3$); ^1H NMR (600 MHz, $CDCl_3$) δ 6.91 (d, *J* = 8.2 Hz, 1H), 6.22 (dd, *J* = 8.2, 2.3 Hz, 1H), 6.08 (d, *J* = 2.3 Hz, 1H), 5.99 (d, *J* = 10.1 Hz, 1H), 5.93 (d, *J* = 10.1 Hz, 1H), 5.92–5.84 (m, 1H), 5.34 (dd, *J* = 17.2, 1.4 Hz, 1H), 5.26 (dd, *J* = 10.4, 1.3 Hz, 1H), 4.63 (ddt, *J* = 13.1, 6.0, 1.3 Hz, 1H), 4.57 (ddt, *J* = 13.0,

6.0, 1.3 Hz, 1H), 4.15 (ddd, *J* = 11.5, 10.0, 1.3 Hz, 1H), 4.06 (s, 1H), 3.99–3.90 (m, 1H), 3.83 (s, 3H), 3.79 (s, 3H), 3.65 (s, 1H), 2.91 (s, 3H), 2.51 (dt, *J* = 12.6, 10.5 Hz, 1H), 2.23 (ddd, *J* = 12.6, 7.6, 1.3 Hz, 1H), 1.17 (dq, *J* = 15.1, 7.6 Hz, 1H), 0.97 (dq, *J* = 14.4, 7.2 Hz, 1H), 0.57 (t, *J* = 7.4 Hz, 3H); ^{13}C NMR (151 MHz, $CDCl_3$) δ 170.3, 169.6, 163.2, 161.8, 154.7, 143.5, 131.2, 124.2, 123.8, 119.6, 119.6, 104.9, 103.1, 94.5, 86.1, 83.8, 65.9, 64.9, 56.4, 56.4, 55.4, 52.8, 52.6, 46.6, 37.7, 35.6, 28.1, 8.7; IR (neat) ν_{max} 2953, 1765, 1732, 1677, 1613, 1500, 1413, 1359, 1243, 1199, 1174, 1132, 1103, 1031, 816 cm^{-1}; ESI-TOF HRMS *m/z* 495.2125 (M+H$^+$, $C_{27}H_{30}N_2O_7$ requires 495.2126).

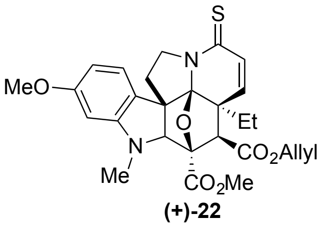

(+)-22

Compound (+)-22. A mixture of (+)-**21** (23.6 mg, 47.7 μmol) and Lawesson's reagent (23.6 mg, 58.3 μmol) in toluene (3.0 mL) was heated at 100 °C for 1 h. The solvent was removed under reduced pressure and the crude residue was directly loaded onto silica gel column. Flash chromatography (SiO_2, 33% EtOAc/hexanes) provided (+)-**22** (22.3 mg, 92%) as a yellow oil: $[\alpha]_D^{23}$ +60 (*c* 1.0, $CHCl_3$); ^{1}H NMR (600 MHz, $CDCl_3$) δ 6.96 (d, *J* = 8.2 Hz, 1H), 6.47 (d, *J* = 9.8 Hz, 1H), 6.22 (dd, *J* = 8.2, 2.3 Hz, 1H), 6.09 (d, *J* = 2.2 Hz, 1H), 5.89 (ddt, *J* = 16.6, 10.6, 5.9 Hz, 1H), 5.63 (d, *J* = 9.7 Hz, 1H), 5.35 (dd, *J* = 17.3, 1.6 Hz, 1H), 5.27 (dd, *J* = 10.4, 1.4 Hz, 1H), 4.67–4.56 (m, 2H), 4.46 (dd, *J* = 13.4, 9.6 Hz, 1H), 4.17 (ddd, *J* = 13.4, 11.3, 7.5 Hz, 1H), 4.11 (s, 1H), 3.84 (s, 3H), 3.80 (s, 3H), 3.68 (s, 1H), 2.93 (s, 3H), 2.58 (td, *J* = 12.0, 9.9 Hz, 1H), 2.29 (dd, *J* = 12.6, 7.4 Hz, 1H), 1.21 (dq, *J* = 15.1, 7.7 Hz, 1H), 0.98 (dq, *J* = 14.4, 7.1 Hz, 1H), 0.54 (t, *J* = 7.4 Hz, 3H); ^{13}C NMR (151 MHz, $CDCl_3$) δ 190.6, 170.0, 169.4, 162.0, 154.8, 134.7, 131.2, 131.1, 123.9, 119.6, 119.3, 103.2, 102.5, 94.6, 86.2, 84.0, 66.0, 65.5, 56.4, 55.4, 53.3, 53.1, 52.7, 37.3, 35.7, 28.2, 8.7; IR (neat) ν_{max} 2951, 1765, 1732, 1612, 1500, 1429, 1355, 1324, 1247, 1174, 1128, 1102, 1028, 897, 734 cm^{-1}; ESI-TOF HRMS *m/z* 511.1896 (M+H$^+$, $C_{27}H_{30}N_2O_6S$ requires 511.1897).

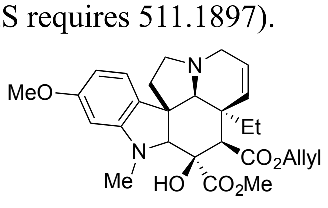

(–)-23

Compound (–)-23. A solution of (+)-**22** (6.1 mg, 11.9 μmol) in CH_2Cl_2 (0.5 mL) was treated with Me_3OBF_4 (5.2 mg, 35 μmol) at room temperature. The reaction mixture was stirred for 30 min and the solvent was removed under a stream of nitrogen. The residue was dissolved in MeOH (0.5 mL), cooled to 0 °C and treated with $NaBH_4$ (1.4 mg, 35.7 μmol). After two more additions of $NaBH_4$ in 15 min intervals, the reaction was quenched with the addition of saturated aqueous $NaHCO_3$ (2.0 mL). The reaction mixture was extracted with CH_2Cl_2 (4 × 2.0 mL). The combined organic phase was washed with saturated aqueous NaCl and dried over Na_2SO_4. The solvent was removed

under vacuum and the residue was purified by PTLC (SiO_2, 75% EtOAc/hexanes) to afford (–)-**23** (3.6 mg, 62%) as a colorless oil: $[\alpha]_D^{28}$ –2.6 (*c* 1.0, $CHCl_3$); ^{1}H NMR (600 MHz, $CDCl_3$) δ 9.49 (s, 1H), 6.87 (d, *J* = 8.1 Hz, 1H), 6.28 (dd, *J* = 8.1, 2.2 Hz, 1H), 6.05 (d, *J* = 2.2 Hz, 1H), 5.92 (ddt, *J* = 16.5, 11.0, 5.8 Hz, 1H), 5.85 (ddd, *J* = 10.2, 5.2, 1.5 Hz, 1H), 5.34 (dd, *J* = 17.2, 1.8 Hz, 1H), 5.23–5.15 (m, 2H), 4.66 (dd, *J* = 13.3, 5.9 Hz, 1H), 4.55 (dd, *J* = 13.3, 5.8 Hz, 1H), 3.78 (s, 3H), 3.78 (s, 3H), 3.66 (s, 1H), 3.49–3.35 (m, 2H), 3.11 (s, 1H), 2.79 (dt, *J* = 15.9, 2.1 Hz, 1H), 2.73 (s, 1H), 2.68 (s, 3H), 2.52 (td, *J* = 10.3, 7.2 Hz, 1H), 2.35–2.20 (m, 2H), 1.82 (dq, *J* = 14.7, 7.4 Hz, 1H), 1.00 (dq, *J* = 14.4, 7.2 Hz, 1H), 0.59 (t, *J* = 7.3 Hz, 3H); ^{13}C NMR (151 MHz, $CDCl_3$) δ 173.6, 171.8, 161.1, 153.7, 132.3, 131.2, 125.0, 125.0, 122.6, 118.4, 104.3, 95.6, 83.2, 78.5, 65.7, 65.0, 55.4, 53.3, 52.3, 51.8, 51.2, 50.8, 44.6, 40.9, 38.4, 32.0, 8.0; IR (neat) ν_{max} 3733, 3628, 2933, 1735, 1615, 1503, 1457, 1247, 1167, 1087, 669, 650 cm^{-1}; ESI-TOF HRMS *m*/*z* 483.2491 (M+H$^+$, $C_{27}H_{34}N_2O_6$ requires 483.2490).

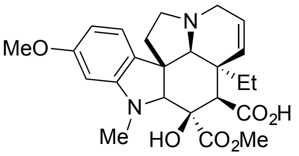

(–)-24

Compound (–)-24. Morpholine (8.0 μL, 92.7 μmol) and $Pd(PPh_3)_4$ (1.1 mg, 0.9 μmol) were added to a mixture of (–)-**23** (4.5 mg, 9.0 μmol) in THF (0.5 mL) and DMSO (50 μL). After stirring for 1 h, the solvent was removed under a stream of N_2. PTLC (SiO_2, 10% MeOH/CH_2Cl_2) provided (–)-**24** (3.0 mg, 73%) as a clear, colorless oil: $[\alpha]_D^{26}$ –6.7 (*c* 1.0, $CHCl_3$); ^{1}H NMR (600 MHz, $CDCl_3$) δ 6.86 (d, *J* = 8.2 Hz, 1H), 6.29 (dd, *J* = 8.2, 2.3 Hz, 1H), 6.05 (d, *J* = 2.2 Hz, 1H), 5.88 (ddd, *J* = 10.2, 5.0, 1.7 Hz, 1H), 5.40 (dt, *J* = 10.2, 2.1 Hz, 1H), 3.82 (s, 3H), 3.76 (s, 3H), 3.70 (s, 1H), 3.50–3.41 (m, 2H), 3.04 (s, 1H), 2.92 (dt, *J* = 16.3, 2.2 Hz, 1H), 2.79 (s, 1H), 2.69 (s, 3H), 2.67–2.60 (m, 1H), 2.35–2.22 (m, 2H), 1.68 (dq, *J* = 14.7, 7.4 Hz, 1H), 0.97 (dq, *J* = 14.4, 7.3 Hz, 1H), 0.58 (t, *J* = 7.4 Hz, 3H); ^{13}C NMR (151 MHz, $CDCl_3$) δ 172.2, 172.1, 161.3, 153.5, 132.2, 124.7, 123.6, 122.5, 104.8, 96.0, 80.6, 78.6, 64.8, 55.4, 53.0, 52.9, 52.1, 50.8, 50.7, 43.5, 40.1, 38.7, 32.3, 7.8; IR (neat) ν_{max} 2924, 1734, 1613, 1597, 1501, 1226, 1168, 1088, 1038, 821, 729, 701 cm^{-1}; ESI-TOF HRMS *m*/*z* 443.2188 (M+H$^+$, $C_{24}H_{30}N_2O_6$ requires 443.2177). The structure and the absolute configuration of (+)-**24** were unambiguously established with a single crystal X-ray structure determination conducted on a colorless prism crystal obtained from vapor diffusion of hexane into a EtOAc solution of (+)-**24** (CCDC 1475226).

(+)-**24** ≡

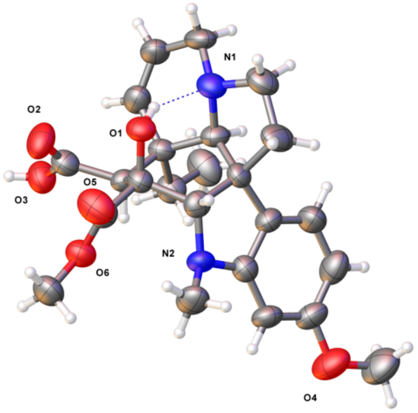

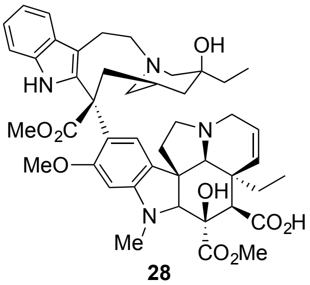

28

Compound 28. (−)-**24** (11.9 mg, 27 μmol) and catharanthine sulfate (23.5 mg, 54 μmol) were combined with TFE (100 μL), aqueous 0.1 N HCl (0.5 mL) and water (0.5 mL) in a 2-dram vial. The vial was fitted with a rubber septum and flushed with Ar. $FeCl_3 \cdot 6H_2O$ (36.5 mg, 135 μmol) was added producing a bright yellow precipitate. The mixture was stirred for 2 h. Meanwhile, a suspension of $Fe_2(ox)_3 \cdot 6H_2O$ (130 mg, 269 μmol) in water (98 mL) was stirred for 2 h, by which time the $Fe_2(ox)_3 \cdot 6H_2O$ was completely dissolved. This mixture was cooled to 0 °C and air was bubbled vigorously through the solution for 15–20 min. The $FeCl_3$ coupling mixture was transferred to the $Fe_2(ox)_3/O_2$ solution by pipette, then $NaBH_4$ (10.5 mg, 529 μmol) was added as a solution in water (1 mL). After stirring for 30 min, the reaction mixture was extracted with 10% MeOH/CH_2Cl_2 (4 × 80 mL). The combined organic extracts were dried (Na_2SO_4) and concentrated under reduced pressure. PTLC (SiO_2, 83:16:1 CH_2Cl_2/MeOH/AcOH) gave **28** (5.2 mg, 24%) as a colorless film, along with the corresponding leurosidine analogue (4.0 mg, 19%) and anhydrovinblastine analogue (2.5 mg, 12%) also as colorless films. For **28**: $[\alpha]_D^{22}$ +2.1 (*c* 0.08, MeOH); ^{1}H NMR (600 MHz, CD_3OD) δ 9.44 (br s, 1H), 7.52 (d, *J* = 8.0 Hz, 1H), 7.29 (d, *J* = 8.1 Hz, 1H), 7.14 (ddd, *J* = 8.2, 7.0, 1.1 Hz, 1H), 7.07 (t, *J* = 7.4 Hz, 1H), 6.73 (s, 1H), 6.43 (s, 1H), 5.95 (ddd, *J* = 10.4, 5.5, 1.7 Hz, 1H), 5.56–5.51 (m, 1H), 4.66 (dd, *J* = 17.4, 10.5 Hz, 1H), 3.98–3.88 (m, 3H), 3.86 (s, 3H), 3.83 (s, 3H), 3.78 (s, 1H), 3.75–3.71 (m, 1H), 3.69 (s, 3H), 3.67–3.62 (m, 1H), 3.62 (s, 1H), 3.53–3.48 (m, 1H), 3.38–3.32 (m, 1H), 3.25 (td, *J* = 11.5, 5.3 Hz, 1H), 3.19 (s, 2H), 2.99 (s, 1H), 2.89 (dd, *J* = 14.5, 6.1 Hz, 1H), 2.78 (s, 3H), 2.48 (d, *J* = 15.8 Hz, 1H), 2.35 (ddd, *J* = 14.1, 9.2, 5.5 Hz, 1H), 2.21–2.13 (m, 1H), 2.07 (ddd, *J* = 13.2, 11.5, 6.2 Hz, 1H), 1.72–1.61 (m, 2H), 1.57–1.46 (m, 3H), 1.43–1.34 (m, 1H), 0.98 (t, *J* = 7.5 Hz, 3H), 0.89 (t, *J* = 7.4 Hz, 3H); IR (neat) ν_{max} 3419, 2950, 1673, 1615, 1459, 1435, 1198, 1136, 1038, 998, 799, 721, 668 cm^{-1}; ESI-TOF HRMS *m/z* 797.4122 (M+H$^+$, $C_{45}H_{56}N_4O_9$ requires 797.4120).

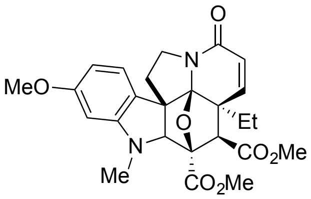

(+)-25

Compound (+)-25. A solution of (+)-**21** (21.7 mg, 44 μmol) in MeOH (2 mL) was treated with MeONa (11.9 mg, 0.21 mmol) at room temperature. After stirring for 1 h, the solvent was removed under vaccum. Flash chromatography (SiO_2, 50% EtOAc/hexanes) provided (+)-**25** (20.5 mg, quant.) as a colorless oil: $[\alpha]_D^{27}$ +34 (*c* 1.0, $CHCl_3$); ^1H NMR (600 MHz, $CDCl_3$) δ 6.91 (d, *J* = 8.3 Hz, 1H), 6.22 (dd, *J* = 8.2, 2.3 Hz, 1H), 6.08 (d, *J* = 2.3 Hz, 1H), 6.00 (d, *J* = 10.0 Hz, 1H), 5.89 (d, *J* = 10.1 Hz, 1H), 4.15 (ddd, *J* = 11.6, 9.9, 1.3 Hz, 1H), 4.07 (s, 1H), 3.98–3.90 (m, 1H), 3.85 (s, 3H), 3.79 (s, 3H), 3.71 (s, 3H), 3.65 (s, 1H), 2.92 (s, 3H), 2.52 (dt, *J* = 12.7, 10.4 Hz, 1H), 2.27–2.18 (m, 1H), 1.17 (dq, *J* = 15.1, 7.6 Hz, 1H), 0.97 (dq, *J* = 14.4, 7.1 Hz, 1H), 0.57 (t, *J* = 7.4 Hz, 3H); ^{13}C NMR (151 MHz, $CDCl_3$) δ 170.4, 170.4, 163.3, 154.7, 143.5, 124.3, 123.8, 119.6, 104.9, 103.1, 94.5, 86.1, 83.8, 64.9, 56.4, 55.4, 52.8, 52.7, 52.1, 46.7, 37.7, 35.6, 28.0, 8.7; IR (neat) ν_{max} 2953, 1764, 1734, 1679, 1613, 1500, 1437, 1416, 1359, 1249, 1201, 1175, 1104, 1033, 817, 732 cm^{-1}; ESI-TOF HRMS *m*/*z* 469.1961 ($M+H^+$, $C_{25}H_{28}N_2O_7$ requires 469.1969).

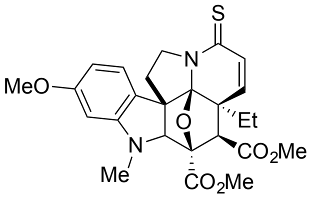

(+)-26

Compound (+)-26. A mixture of (+)-**25** (20 mg, 43μmol) and Lawesson's reagent (19 mg, 47 μmol) in toluene (2.0 mL) was heated at 100 °C for 1 h. Solvent was removed under reduced pressure and crude residue was directly loaded onto a silica gel column. Flash chromatography (SiO_2, 33% EtOAc/hexanes) provided (+)-**26** (18 mg, 87%) as a yellow oil: $[\alpha]_D^{26}$ +66 (*c* 1.0, $CHCl_3$); ^1H NMR (600 MHz, $CDCl_3$) δ 6.96 (d, *J* = 8.2 Hz, 1H), 6.48 (d, *J* = 9.8 Hz, 1H), 6.22 (dd, *J* = 8.2, 2.4 Hz, 1H), 6.09 (d, *J* = 2.3 Hz, 1H), 5.58 (d, *J* = 9.8 Hz, 1H), 4.49–4.41 (m, 1H), 4.22–4.12 (m, 1H), 4.10 (s, 1H), 3.86 (s, 3H), 3.80 (s, 3H), 3.72 (s, 3H), 3.67 (s, 1H), 2.93 (s, 3H), 2.58 (td, *J* = 12.2, 9.9 Hz, 1H), 2.29 (dd, *J* = 12.6, 7.4 Hz, 1H), 1.26–1.16 (m, 2H), 0.97 (dq, *J* = 14.4, 7.2 Hz, 1H), 0.54 (t, *J* = 7.4 Hz, 3H); ^{13}C NMR (151 MHz, $CDCl_3$) δ 190.6, 170.2, 170.1, 162.0, 154.8, 134.7, 131.2, 123.9, 119.3, 103.2, 102.5, 94.6, 86.2, 84.0, 65.5, 56.4, 55.4, 53.2, 53.1, 52.7, 52.2, 37.3, 35.7, 28.1, 8.6; IR (neat) ν_{max} 2953, 1765, 1735, 1614, 1500, 1431, 1355, 1249, 1200, 1174, 1102, 1031, 909, 731 cm^{-1}; ESI-TOF HRMS *m*/*z* 485.1740 ($M+H^+$, $C_{25}H_{28}N_2O_6S$ requires 485.1741).

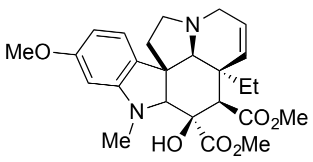

(+)-27

Compound (+)-27. A solution of (+)-**26** (18 mg, 37 μmol) in CH_2Cl_2 (1 mL) was treated with Me_3OBF_4 (16.5 mg, 0.11 mmol) at room temperature. The reaction mixture was stirred for 30 min and the solvent was removed under a stream of nitrogen. The residue was dissolved in MeOH (1 mL), cooled to 0 °C and treated with $NaBH_4$ (4.2 mg, 0.11 mmol). After two more additions of $NaBH_4$ in 15 min intervals, the reaction was quenched with the addition of saturated aqueous $NaHCO_3$ (2.0 mL). The reaction mixture was extracted with CH_2Cl_2 (4 × 2.0 mL). The combined organic phase was washed with saturated aqueous NaCl and dried over Na_2SO_4. The solvent was removed under vacuum and the residue was purified by PTLC (SiO_2, 75% EtOAc/hexanes) to afford (+)-**27** (10.3 mg, 61%): $[\alpha]_D^{25}$ +2.6 (*c* 0.33, $CHCl_3$), $[\alpha]_D^{28}$ −28.1 (*c* 0.334, MeOH); ^1H NMR (600 MHz, $CDCl_3$) δ 9.56 (br s, 1H), 6.87 (d, *J* = 8.4 Hz, 1H), 6.26 (dd, *J* = 8.4, 2.1 Hz, 1H), 6.02 (d, *J* = 2.1 Hz, 1H), 5.85 (dd, *J* = 10.2, 4.2 Hz, 1H), 5.13 (d, *J* = 10.2 Hz, 1H), 3.77 (s, 3H), 3.75 (s, 3H), 3.66 (s, 3H), 3.66 (s, 1H), 3.50–3.44 (m, 2H), 3.08 (s, 1H), 2.85 (d, *J* = 15.6 Hz, 1H), 2.78 (s, 1H), 2.66 (s, 3H), 2.60–2.55 (m, 1H), 2.33–2.23 (m, 2H), 1.77 (dq, *J* = 14.4, 7.2 Hz, 1H), 0.98 (dq, *J* = 14.4, 7.2 Hz, 1H), 0.57 (t, *J* = 7.2 Hz, 3H); ^{13}C NMR (150 MHz, $CDCl_3$) δ 173.5, 172.7, 161.1, 153.6, 131.2, 124.79, 124.78, 122.6, 104.4, 95.6, 82.8, 78.6, 65.8, 55.3, 53.3, 52.3, 51.8, 51.4, 51.2, 50.7, 44.4, 40.8, 38.4, 32.0, 7.9; IR (neat) ν_{max} 3375, 2950, 2876, 1734, 1614, 1502, 1454, 1435, 1360, 1332, 1168, 1087, 1041, 979, 942, 915, 825, 730 cm^{-1}; ESI-TOF HRMS *m*/*z* 457.2331 ($M+H^+$, $C_{25}H_{33}N_2O_6$ requires 457.2333).

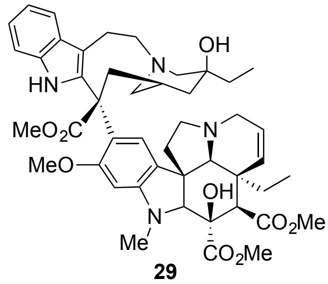

29

Compound 29. (+)-**27** (6.3 mg, 13.8 μmol) and catharanthine sulfate (5.3 mg, 6.90 μmol) were combined with TFE (63 μL), aqueous 0.1 N HCl (0.32 mL) and water (0.32 mL) in a 2-dram vial. The vial was fitted with a rubber septum and flushed with Ar. $FeCl_3 \cdot 6H_2O$ (18.7 mg, 69.0 μmol) was added producing a bright yellow precipitate. The mixture was stirred for 2 h during which time the precipitate re-dissolved leaving a clear, yellow solution. Meanwhile, a suspension of $Fe_2(ox)_3 \cdot 6H_2O$ (66.8 mg, 138 μmol) in water (58 mL) was stirred for 2 h, by which time the $Fe_2(ox)_3 \cdot 6H_2O$ was completely dissolved. This mixture was cooled to 0 °C and air was bubbled vigorously through the solution for 15–20 min. The $FeCl_3$ coupling mixture was transferred to the $Fe_2(ox)_3/O_2$ solution by pipette, then $NaBH_4$ (10.5 mg, 330 μmol) was added as a solution in water (1 mL). After stirring for 30 min, the reaction was quenched by the addition of aqueous NH_4OH (16 mL) and the resulting mixture was extracted with 10% $MeOH/CH_2Cl_2$ (4

× 50 mL). The combined organic extracts were dried (Na_2SO_4) and concentrated under reduced pressure. PTLC (SiO_2, 97:3:3 EtOAc/MeOH/Et_3N) gave **29** (3.5 mg, 31%) as a colorless film, along with the corresponding leurosidine analogue (2.3 mg, 21%) and anhydrovinblastine analogue (1.9 mg, 17%) also as colorless films. For **29**: $[\alpha]_D^{25}$ +49 (*c* 0.18, $CHCl_3$); ^1H NMR (600 MHz, $CDCl_3$) δ 9.75 (br s, 1H), 8.05 (s, 1H), 7.53 (d, *J* = 7.9 Hz, 1H), 7.20–7.06 (m, 3H), 6.65 (s, 1H), 6.08 (s, 1H), 5.88–5.80 (m, 1H), 5.15 (d, *J* = 10.2 Hz, 1H), 3.95 (t, *J* = 14.1 Hz, 1H), 3.80 (s, 3H), 3.78 (s, 3H), 3.71 (d, 3H), 3.65 (s, 1H), 3.61 (s, 3H), 3.41–3.23 (m, 2H), 3.13 (d, *J* = 14.6 Hz, 2H), 2.83–2.77 (m, 2H), 2.70 (s, 3H), 2.69 (s, 1H), 2.48–2.43 (m, 1H), 2.43–2.37 (m, 1H), 2.29 (d, *J* = 15.2 Hz, 1H), 2.16 (ddd, *J* = 14.9, 9.3, 6.1 Hz, 1H), 2.00 (dq, *J* = 14.7, 7.5 Hz, 1H), 1.87–1.78 (m, 1H), 1.47 (d, *J* = 14.3 Hz, 1H), 1.40 (dd, *J* = 14.3, 6.1 Hz, 1H), 0.94–0.84 (m, 6H); IR (neat) ν_{max} 2940, 1731, 1657, 1613, 1502, 1458, 1432, 1362, 1333, 1295, 1224, 1169, 1142, 1090, 1039, 1004, 944, 921, 895, 848, 819, 795, 763, 730, 700 cm^{-1}; ESI-TOF HRMS *m*/*z* 811.4263 ($M+H^+$, $C_{46}H_{59}N_4O_9$ requires 811.4276).

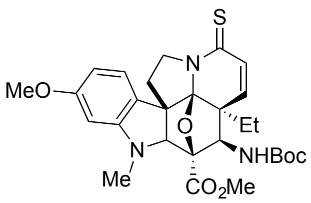

(+)-31

Compound (+)-31. Morpholine (54 μL, 0.62 mmol) and $Pd(PPh_3)_4$ (7.2 mg, 6.2 μmol) were added to a mixture of (+)-**22** (31.8 mg, 62.3 μmol) in THF (1 mL) and DMSO (0.1 mL). After stirring for 1 h, the mixture was diluted with EtOAc (3 mL) and washed with aqueous 2 N HCl (3 × ca.1 mL), then saturated aqueous NaCl. The combined aqueous layers were re-extacted with EtOAc, then the combined EtOAc extracts were dried (Na_2SO_4) and concentrated to give the crude product. The crude product was directly dissolved in *t*-BuOH (1 mL, distilled from CaH_2 and stored over 4Å MS) followed by addition of Et_3N (18.8 mg, 0.186 mmol) and DPPA (34 mg, 0.124 mmol). The resulting mixture was heated at 85 °C for 16 h. Upon cooling to room temperature volatiles were evaporated under a stream of N_2. Flash chromatography (SiO_2, 33% EtOAc/hexanes) provided (+)-**31** (21.5 mg, 64%) as a yellow oil: $[\alpha]_D^{24}$ +217 (*c* 0.6, $CHCl_3$); ^1H NMR (600 MHz, $CDCl_3$) δ 6.91 (d, *J* = 8.2 Hz, 1H), 6.55 (d, *J* = 9.8 Hz, 1H), 6.20 (dd, *J* = 8.2, 2.3 Hz, 1H), 6.09 (d, *J* = 2.3 Hz, 1H), 6.01 (d, *J* = 9.8 Hz, 1H), 4.83 (d, *J* = 11.5 Hz, 1H), 4.71 (d, *J* = 11.5 Hz, 1H), 4.45 (ddd, *J* = 13.5, 9.7, 1.1 Hz, 1H), 4.24–4.14 (m, 2H), 3.85 (s, 3H), 3.79 (s, 3H), 3.06 (s, 3H), 2.58–2.48 (m, 1H), 2.28 (ddd, *J* = 12.5, 7.6, 1.1 Hz, 1H), 1.43 (s, 9H), 1.07 (dq, *J* = 15.0, 7.5 Hz, 1H), 0.97–0.89 (m, 1H), 0.51 (t, *J* = 7.4 Hz, 3H); ^{13}C NMR (151 MHz, $CDCl_3$) δ 190.4, 168.6, 162.0, 155.1, 154.6, 134.1, 131.3, 123.7, 119.2, 103.3, 102.3, 94.4, 90.2, 81.5, 80.4, 65.3, 61.4, 55.4, 53.9, 53.3, 52.6, 36.9, 35.6, 28.3, 28.1, 27.0, 8.6; IR (neat) ν_{max} 2974, 1741, 1714, 1612, 1500, 1430, 1352, 1319, 1245, 1157, 1099, 1050, 891, 753 cm^{-1}; ESI-TOF HRMS *m*/*z* 542.2320 ($M+H^+$, $C_{28}H_{35}N_3O_6S$ requires 542.2319).

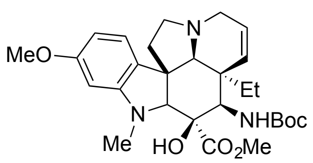

(+)-32

Compound (+)-32. A solution of (+)-**31** (13 mg, 24 μmol) in CH_2Cl_2 (0.5mL) was treated with 4Å MS (260 mg), 2,6-di-*tert*-butylpyridine (104 μL, 0.48 mmol) and Me_3OBF_4 (10.6 mg, 0.72 μmol) at room temperature. The reaction mixture was stirred for 30 min and the solvent was removed under a stream of nitrogen. The residue was dissolved in MeOH (1 mL), cooled to 0 °C and treated with $NaBH_4$ (2.7 mg, 72 μmol). After two more additions of $NaBH_4$ in 15 min intervals, the reaction was quenched with the addition of saturated aqueous $NaHCO_3$ (2.0 mL). The reaction mixture was extracted with CH_2Cl_2 (4 × 2.0 mL). The combined organic phase was washed with saturated aqueous NaCl and dried over Na_2SO_4. The solvent was removed under vacuum and the residue was purified by PTLC (SiO_2, 50% acetone/hexanes) to afford (+)-**32** (6.1 mg, 50%) as a colorless oil: $[\alpha]_D^{24}$ +18 (*c* 0.75, $CHCl_3$); ^{1}H NMR (600 MHz, $CDCl_3$) δ 9.32 (s, 1H), 6.87 (d, *J* = 8.2 Hz, 1H), 6.27 (dd, *J* = 8.1, 2.3 Hz, 1H), 6.04 (d, *J* = 2.2 Hz, 1H), 5.79 (ddd, *J* = 10.2, 4.7, 1.7 Hz, 1H), 5.31 (dt, *J* = 10.2, 2.0 Hz, 1H), 5.03 (d, *J* = 10.3 Hz, 1H), 4.29 (d, *J* = 10.2 Hz, 1H), 3.78 (s, 3H), 3.77 (s, 3H), 3.64 (s, 1H), 3.50–3.43 (m, 1H), 3.43–3.35 (m, 1H), 2.81 (dd, *J* = 16.1, 2.3 Hz, 1H), 2.66 (s, 3H), 2.60 (s, 1H), 2.52–2.45 (m, 1H), 2.30 (dd, *J* = 9.5, 6.0 Hz, 2H), 1.62–1.55 (m, 1H), 1.41 (s, 9H), 1.12 (dq, *J* = 14.4, 7.3 Hz, 1H), 0.46 (t, *J* = 7.4 Hz, 3H); With minor (~10%) NHBoc rotamer peaks observed at: δ 6.90 (d, *J* = 8.2 Hz, 1H), 6.30 (dd, *J* = 8.1, 2.3 Hz, 1H), 6.10 (d, *J* = 2.3 Hz, 1H), 4.84 (d, *J* = 10.1 Hz, 1H), 4.21 (d, *J* = 10.1 Hz, 1H), 3.62 (s, 1H), 1.47 (s, 9H). ^{13}C NMR (151 MHz, $CDCl_3$) δ 172.9, 161.1, 155.5, 154.1, 132.4, 125.4, 123.4, 122.6, 104.5, 95.8, 83.1, 80.1, 78.8, 67.3, 55.4, 55.3, 52.4, 52.4, 51.5, 44.0, 43.2, 38.8, 32.1, 28.4, 7.8; With minor (~10%) NHBoc rotamer peaks observed at: δ 96.2, 67.5, 45.8; IR (neat) ν_{max} 2966, 2929, 1715, 1614, 1501, 1458, 1366, 1242, 1170, 1043, 727, 533 cm^{-1}; ESI-TOF HRMS *m/z* 514.2911 (M+H$^+$, $C_{28}H_{39}N_3O_6$ requires 514.2911).

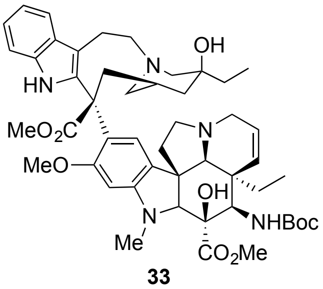

33

Compound 33. (+)-**32** (4.2 mg, 8 μmol) and catharanthine sulfate (7.1 mg, 16 μmol) were combined with TFE (34 μL), aqueous 0.1 N HCl (0.17 mL) and water (0.17 mL) in a 2-dram vial. The vial was fitted with a rubber septum and flushed with Ar. $FeCl_3·6H_2O$ (11.1 mg, 41 μmol) was added producing a bright yellow precipitate. The mixture was stirred for 2 h. Meanwhile, a suspension of $Fe_2(ox)_3·6H_2O$ (40 mg, 83 μmol) in water (33 mL) was stirred for 2 h, by which time the $Fe_2(ox)_3·6H_2O$ was completely

dissolved. This mixture was cooled to 0 °C and air was bubbled vigorously through the solution for 15–20 min. The $FeCl_3$ coupling mixture was transferred to the $Fe_2(ox)_3/O_2$ solution by pipette, then $NaBH_4$ (6 mg, 158 μmol) was added as a solution in water (0.5 mL). After stirring for 30 min, the reaction was quenched by the addition of aqueous NH_4OH (2 mL) and the resulting mixture was extracted with 10% $MeOH/CH_2Cl_2$ (4 × 30 mL). The combined organic extracts were dried (Na_2SO_4) and concentrated under reduced pressure. PTLC (SiO_2, 95:5:3 EtOAc/MeOH/Et_3N) gave **33** (2.0 mg, 28%) as a white film, along with the corresponding leurosidine analogue (1.3 mg, 18%) and anhydrovinblastine analogue (1.6 mg, 24%) also as white films: $[\alpha]_D^{25}$ +18 (*c* 0.40, $CHCl_3$); ^1H NMR (600 MHz, $CDCl_3$) δ 9.54 (br s, 1H), 8.03 (s, 1H), 7.51 (d, *J* = 7.9 Hz, 1H), 7.19–7.05 (m, 3H), 6.59 (s, 1H), 6.08 (s, 1H), 5.79 (ddd, *J* = 10.2, 4.8, 1.7 Hz, 1H), 5.35 (dt, *J* = 10.2, 2.1 Hz, 1H), 5.00 (d, *J* = 10.4 Hz, 1H), 4.25 (d, *J* = 10.3 Hz, 1H), 3.95 (t, *J* = 14.2 Hz, 1H), 3.78 (d, *J* = 1.6 Hz, 6H), 3.61 (s, 1H), 3.59 (s, 3H), 3.40 (d, *J* = 13.7 Hz, 1H), 3.37–3.28 (m, 2H), 3.28–3.21 (m, 1H), 3.18–3.08 (m, 2H), 2.86–2.76 (m, 2H), 2.70 (s, 3H), 2.62 (s, 1H), 2.48–2.36 (m, 2H), 2.33–2.25 (m, 1H), 2.15–2.06 (m, 1H), 1.81 (ddd, *J* = 13.6, 11.2, 5.0 Hz, 1H), 1.73 (dq, *J* = 14.9, 7.5 Hz, 2H), 1.54–1.45 (m, 2H), 1.43 (s, 9H), 1.36–1.27 (m, 3H), 0.88 (t, *J* = 7.5 Hz, 3H), 0.81 (t, *J* = 7.3 Hz, 3H); With minor (~4%) NHBoc rotamer peaks observed at: δ 6.14 (s, 1H), 5.30 (d, *J* = 10.5 Hz, 1H), 4.84 (d, *J* = 10.5 Hz, 1H), 1.51 (s, 9H); IR (neat) ν_{max} 2930, 1737, 1716, 1614, 1498, 1457, 1431, 1366, 1330, 1297, 1223, 1172, 1098, 1035, 1011, 801, 751 cm^{-1}; ESI-TOF HRMS *m/z* 868.4851 ($M+H^+$, $C_{49}H_{65}N_5O_9$ requires 868.4855).

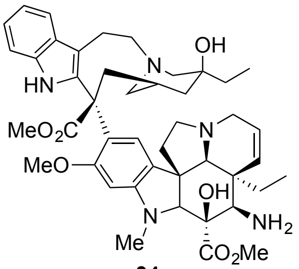

34

Compound 34. Compound **33** (3.2 mg, 3.7 μmol) was dissolved in 5:1 CH_2Cl_2/TFA (0.6 mL) and stirred for 2 h, then diluted with saturated aqueous $NaHCO_3$ (3 mL). The mixture was extracted with 10% $MeOH/CH_2Cl_2$ (3 × 2 mL). The combined organic extracts were dried (Na_2SO_4) and concentrated under reduced pressure. The crude products were purified by PTLC (SiO_2, 95:5:3 EtOAc/MeOH/Et_3N) to give **34** (2.1 mg, 74%) as a white film: $[\alpha]_D^{26}$ +54 (*c* 0.10, $CHCl_3$); ^1H NMR (600 MHz, $CDCl_3$) δ 9.46 (br s, 1H), 8.04 (s, 1H), 7.51 (d, *J* = 7.9 Hz, 1H), 7.21–7.07 (m, 3H), 6.59 (s, 1H), 6.10 (s, 1H), 5.80 (ddd, *J* = 10.4, 4.8, 1.6 Hz, 1H), 5.66 (dt, *J* = 10.3, 2.1 Hz, 1H), 3.94 (t, *J* = 14.4 Hz, 1H), 3.84 (s, 3H), 3.78 (s, 3H), 3.65 (s, 1H), 3.60 (s, 3H), 3.48–3.41 (m, 1H), 3.41–3.33 (m, 2H), 3.31 (dt, *J* = 5.2, 2.6 Hz, 1H), 3.30–3.23 (m, 2H), 3.21–3.11 (m, 3H), 2.84 (dt, *J* = 16.0, 2.2 Hz, 1H), 2.72 (s, 3H), 2.62 (s, 1H), 2.49–2.40 (m, 2H), 2.31 (d, *J* = 15.5 Hz, 1H), 2.12–2.05 (m, 1H), 1.96 (s, 1H), 1.77 (ddd, *J* = 13.9, 11.6, 5.6 Hz, 1H), 1.71 (dt, *J* = 14.7, 7.4 Hz, 1H), 1.50 (d, *J* = 14.5 Hz, 1H), 1.46–1.39 (m, 2H), 1.34 (d, *J* = 8.1 Hz, 2H), 1.23–1.16 (m, 1H), 0.93–0.84 (m, 6H); IR (neat) ν_{max} 2922, 1729, 1503, 1459, 1230, 1094, 1071, 1036, 748, 693, 587, 530 cm^{-1}; ESI-TOF HRMS *m/z* 768.4332 ($M+H^+$, $C_{44}H_{57}N_5O_7$ requires 768.4331).

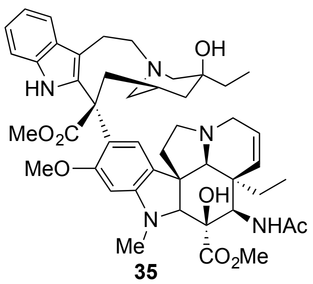

35

Compound 35. Compound **34** (1.5 mg, 1.95 μmol) was dissolved in 0.2 mL of anhydrous CH_2Cl_2. DMAP (0.5 mg, 4 μmol) and acetic anhydride (0.4 mg, 3.9 μmmol) were added and the resulting mixture stirred for 30 min, then diluted with saturated aqueous $NaHCO_3$ (2 mL). The mixture was extracted with 10% MeOH/CH_2Cl_2 (4 × 2 mL). The combined organic extracts were dried (Na_2SO_4) and concentrated under reduced pressure. The crude products were purified by PTLC (SiO_2, 95:5:3 EtOAc/MeOH/Et_3N) to give **35** (1.0 mg, 63%) as a white film: $[\alpha]_D^{29}$ +21 (*c* 0.10, $CHCl_3$); ^1H NMR (600 MHz, $CDCl_3$) δ 9.79 (s, 1H), 8.04 (s, 1H), 7.50 (d, *J* = 8.1 Hz, 1H), 7.21–7.13 (m, 1H), 7.15–7.07 (m, 2H), 6.57 (d, *J* = 7.4 Hz, 1H), 6.10 (s, 1H), 5.88 (d, *J* = 10.0 Hz, 1H), 5.78 (ddd, *J* = 10.2, 4.7, 1.7 Hz, 1H), 5.27 (dt, *J* = 10.1, 2.1 Hz, 1H), 4.60 (d, *J* = 10.0 Hz, 1H), 3.94 (t, *J* = 14.3 Hz, 1H), 3.80 (s, 3H), 3.78 (s, 3H), 3.64 (s, 1H), 3.60 (s, 3H), 3.46 (d, *J* = 14.1 Hz, 1H), 3.42–3.35 (m, 1H), 3.37–3.31 (m, 1H), 3.30–3.22 (m, 2H), 3.23–3.12 (m, 2H), 3.08 (s, 1H), 2.92–2.79 (m, 3H), 2.69 (s, 3H), 2.61 (s, 1H), 2.49 (d, *J* = 13.4 Hz, 1H), 2.43 (td, *J* = 10.5, 6.4 Hz, 1H), 2.32 (d, *J* = 15.1 Hz, 1H), 2.19–2.09 (m, 1H), 1.97–1.95 (m, 4H), 1.91–1.83 (m, 1H), 1.86–1.77 (m, 1H), 1.52 (d, *J* = 14.1 Hz, 1H), 1.48–1.40 (m, 1H), 1.39–1.29 (m, 3H), 0.90 (t, *J* = 7.5 Hz, 3H), 0.79 (t, *J* = 7.4 Hz, 3H); IR (neat) ν_{max} 3466, 2921, 2852, 1737, 1661, 1614, 1503, 1459, 1433, 1259, 1224, 1095, 1224, 1095, 1037, 1017, 802, 745 cm^{-1}; ESI-TOF HRMS *m*/*z* 810.4438 ($M+H^+$, $C_{46}H_{59}N_5O_8$ requires 810.4436).

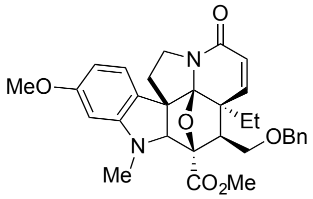

(+)-37

Compound (+)-37. A solution of **8d** (91 mg, 0.17 mmol) in THF (8.5 mL) was treated with LiHMDS (0.51 mL, 1.0 M in THF, 0.51 mmol) at –78 °C. After stirring for 30 min, a solution of PhSeCl (59 mg, 0.31 mmol) in THF (1 mL) was added at –78 °C and the mixture was stirred for 1 h at the same temperature before the reaction was quenched with the addition of water. The reaction mixture was extracted with EtOAc (3 × 50 mL), and dried over Na_2SO_4 and evaporated to dryness. The crude residue in THF (5 mL) was treated with pyridine (0.1 mL) and *m*CPBA (37 mg, 0.215 mmol) at 0 °C, and slowly the temperature was warmed to room temperature over 2 h before the reaction was quenched with the addition of saturated aqueous $Na_2S_2O_3$. The reaction mixture was extracted with CH_2Cl_2, and dried with Na_2SO_4. The solvent was removed under vacuum. PTLC (SiO_2, 50% EtOAc/hexanes) provided (+)-**37** (41 mg, 72%) as colorless foam. Chiral HPLC (chiralcel OD, 60:40 *i*PrOH/hexanes, 7 mL/min) allowed for

separation of the enantiomers of **37**: t_R (natural) = 20.2 min, t_R (unnatural) = 28.4 min. For (+)-**37**: $[\alpha]_D^{25}$ +126 (*c* 1.0, $CHCl_3$); ^{1}H NMR (500 MHz, $CDCl_3$) δ 7.28–7.23 (m, 2H), 7.22–7.17 (m, 3H), 6.82 (d, *J* = 8.1 Hz, 1H), 6.20 (d, *J* = 10.5 Hz, 1H), 6.11 (dd, *J* = 8.2, 2.2 Hz, 1H), 5.98 (d, *J* = 2.5 Hz, 1H), 5.91 (d, *J* = 10.0 Hz, 1H), 4.36 (s, 2H), 4.04 (s, 1H), 4.03 (t, *J* = 11.0 Hz, 1H), 3.84 (dt, *J* = 7.5, 11.0 Hz, 1H), 3.70 ((s, 3H), 3.64 (s, 3H), 3.57 (dd, *J* = 7.5, 9.0 Hz, 1H), 3.47 (dd, *J* = 6.5, 9.0 Hz, 1H), 2.94 (s, 3H), 2.87 (t, *J* = 6.5 Hz, 1H), 2.38 (q, *J* = 11.0 Hz, 1H), 2.12 (dd, *J* = 7.5, 13.0 Hz, 1H), 0.98 (dq, *J* = 14.5, 7.5 Hz, 1H), 0.80 (dq, *J* = 14.5, 7.0 Hz, 1H), 0.43 (t, *J* = 7.5 Hz, 3H); ^{13}C NMR (125 MHz, $CDCl_3$) δ 170.4, 163.7, 161.8, 155.2, 144.7, 137.6, 128.4, 127.7, 127.4, 123.9, 123.8, 120.1, 105.0, 102.5, 94.3, 86.8, 83.4, 73.2, 67.9, 64.8, 55.4, 52.4, 51.2, 50.6, 46.5, 37.8, 35.4, 27.7, 8.9; IR (neat) ν_{max} 2978, 1733, 1677, 1611, 1499, 1414, 1371, 1235, 1095, 1042, 882, 825, 735, 637, 609 cm^{-1}; ESI-TOF HRMS *m*/*z* 531.2491 (M+H$^+$, $C_{31}H_{34}N_2O_6$ requires 531.2490).

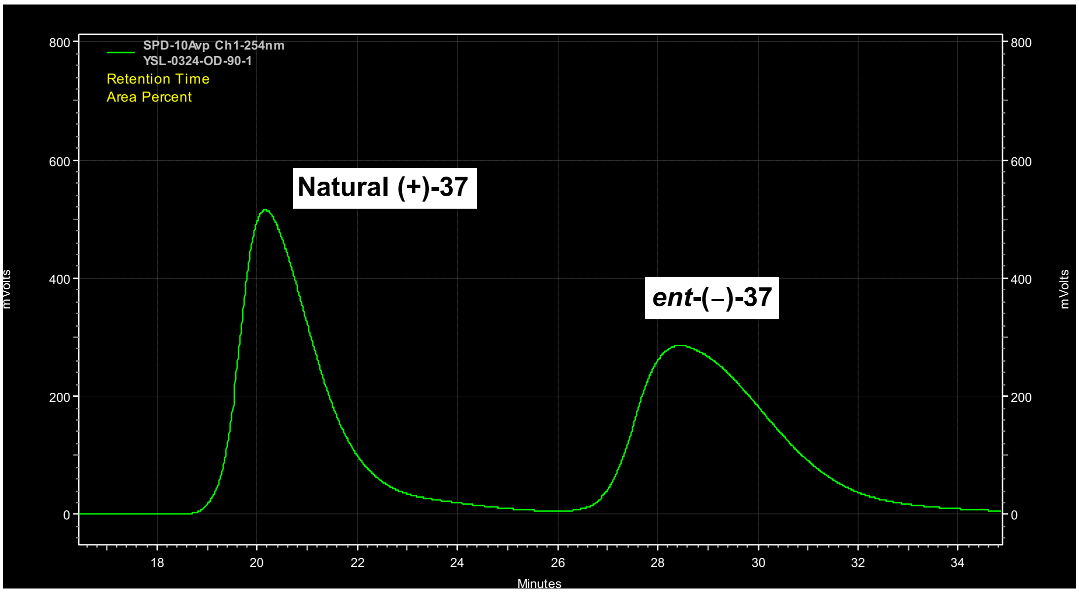

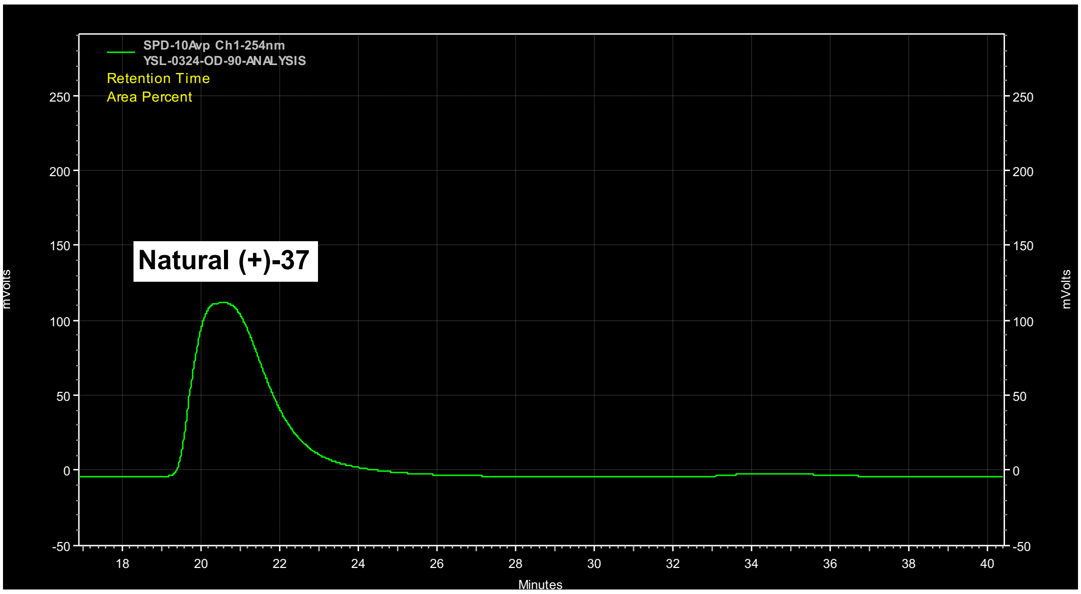

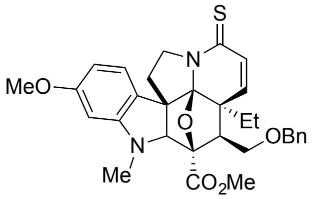

(+)-38

Compound (+)-38. A solution of (+)-**37** (28 mg, 0.053 mmol) in toluene (5 mL) was treated with Lawesson's reagent (21 mg, 0.053 mmol) and the solution was heated at 100°C for 1 h. After cooling the reaction mixture to room temperature, the solvent was removed under reduced pressure. The crude mixture was purified by PTLC (SiO_2, 30% EtOAc/hexanes) to give (+)-**38** (26.5 mg, 92%) as a yellow oil: $[\alpha]_D^{24}$ +392 (*c* 1.0, $CHCl_3$); ^{1}H NMR (500 MHz, $CDCl_3$) δ 7.27–7.22 (m, 2H), 7.21–7.16 (m, 3H), 6.85 (d, *J* = 8.3 Hz, 1H), 6.40 (d, *J* = 9.8 Hz, 1H), 6.10 (dd, *J* = 8.2, 2.3 Hz, 1H), 5.99 (d, *J* = 2.2 Hz, 1H), 5.90 (d, *J* = 9.8 Hz, 1H), 4.37 (s, 2H), 4.33 (dd, *J* = 13.5, 9.5 Hz, 1H), 4.11–4.02 (m, 2H), 3.70 (s, 3H), 3.64 (s, 3H), 3.59 (dd, *J* = 9.3, 7.4 Hz, 1H), 3.49 (dd, *J* = 9.3, 6.4 Hz, 1H), 2.95 (s, 3H), 2.89 (t, *J* = 6.9 Hz, 1H), 2.44 (td, *J* = 11.9, 9.8 Hz, 1H), 2.17 (dd, *J* = 12.5, 7.3 Hz, 1H), 1.02 (dq, *J* = 15.0, 7.6 Hz, 1H), 0.81 (dq, *J* = 14.1, 7.0 Hz, 1H), 0.39 (t, *J* = 7.3 Hz, 3H); ^{13}C NMR (125 MHz, $CDCl_3$) δ 190.7, 169.9, 161.8, 155.1, 137.5, 136.1, 130.8, 128.4 (2C), 127.7, 127.3 (2C), 123.8, 119.7, 102.6 (2C), 94.3, 86.8, 83.4, 73.1, 67.7, 65.4, 55.4, 53.0, 52.4, 51.6, 50.5, 37.3, 35.4, 27.7, 8.7; IR (neat) ν_{max} 2949, 1734, 1610, 1499, 1431, 1200, 1101, 1051 cm^{-1}; ESI-TOF HRMS *m*/*z* 547.2267 (M+H$^+$, $C_{31}H_{34}N_2O_5S$ requires 547.2261).

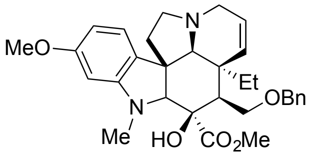

(–)-39

Compound (–)-39. A solution of (+)-**38** (17 mg, 0.031 mmol) in CH_2Cl_2 (1 mL) was treated with Me_3OBF_4 (9.2 mg, 0.062 mmol) at 0 °C. The reaction mixture was allowed to warm to room temperature over 1 h and the solvent was removed under a stream of nitrogen. The residue was dissolved in a mixture of MeOH (1 mL) and TFE (1 mL), cooled to 0 °C and treated with $NaBH_4$ (2.3 mg, 0.062 mmol). After two more additions of $NaBH_4$ in 15 min intervals, the reaction was quenched by the addition of saturated aqueous $NaHCO_3$ (2 mL). The aqueous phase was extracted with CH_2Cl_2 (4 × 10 mL). The combined organic phase was washed with saturated aqueous NaCl and dried over Na_2SO_4. The solvent was removed under vacuum and the residue was purified by PTLC (SiO_2, 5% MeOH/CH_2Cl_2) to afford (–)-**39** (11 mg, 71%): $[\alpha]_D^{24}$ –7.7 (*c* 1.0, $CHCl_3$); ^{1}H NMR (500 MHz, $CDCl_3$) δ 8.91 (s, 1H), 7.33–7.27 (m, 4H), 7.25–7.20 (m, 1H), 6.90 (d, *J* = 8.2 Hz, 1H), 6.29 (dd, *J* = 8.1, 2.2 Hz, 1H), 6.08 (d, *J* = 2.2 Hz, 1H), 5.77–5.64 (m, 2H), 4.50–4.39 (m, 2H), 3.77 (s, 3H), 3.76 (s, 3H), 3.73 (d, *J* = 9.9 Hz, 1H), 3.53–3.43 (m, 3H), 3.40–3.32 (m, 1H), 2.79 (dt, *J* = 16.3, 2.2 Hz, 1H), 2.66 (s, 3H), 2.61 (dd, *J* = 9.5, 2.6 Hz, 1H), 2.53 (s, 1H), 2.44 (q, *J* = 9.4 Hz, 1H), 2.36–2.26 (m, 2H),

1.45 (dq, J = 14.9, 7.5 Hz, 1H), 1.09 (dq, J = 14.4, 7.3 Hz, 1H), 0.45 (t, J = 7.3 Hz, 3H); ^{13}C NMR (125 MHz, $CDCl_3$) δ 174.3, 161.0, 154.6, 138.9, 133.7, 128.2 (2C), 127.5 (2C), 127.2, 126.6, 123.7, 122.8, 104.4, 96.1, 83.9, 79.6, 72.3, 72.1, 68.0, 55.4, 53.2, 52.4, 52.3, 44.8, 44.4, 41.7, 39.6, 34.1, 8.4; IR (neat) ν_{max} 2949, 2873, 1732, 1614, 1499, 1453, 1222, 1085, 1046 cm^{-1}; ESI-TOF HRMS m/z 519.2851 ($M+H^+$, $C_{31}H_{38}N_2O_5$ requires 519.2853). The structure and the absolute configuration of (–)-**39** were unambiguously established with a single crystal X-ray structure determination conducted on a white needle crystal obtained from vapor diffusion of hexane into a EtOAc solution of (–)-**39** (CCDC 1475227).

(–)-39 ≡

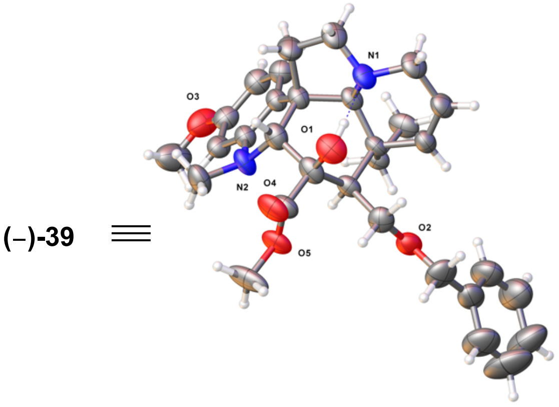

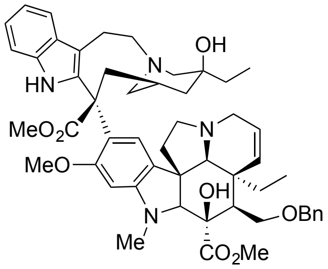

40

Compound 40. (–)-**39** (7 mg, 13.5 μmol) and catharanthine sulfate (11.7 mg, 26.9 μmol) were combined with TFE (56 μL), aqueous 0.1 N HCl (0.28 mL) and H_2O (0.28 mL) in a 2-dram vial. The vial was fitted with a rubber septum and flushed with Ar. $FeCl_3{\cdot}6H_2O$ (18.2 mg, 67 μmol) was added producing a bright yellow precipitate. The reaction mixture was stirred for 2 h at 25 °C. Meanwhile, in a separate flask, $Fe_2(ox)_3{\cdot}6H_2O$ (67 mg, 0.138 mmol) in H_2O (55 mL) was stirred for 2 h, by which time the $Fe_2(ox)_3{\cdot}6H_2O$ was completely dissolved. This mixture was cooled to 0 °C and air was bubbled vigorously through the solution for 15–20 min. The $FeCl_3$ coupling mixture was transferred to the $Fe_2(ox)_3/O_2$ solution by pipette, then $NaBH_4$ (10.3 mg, 0.27 mmol) was added as a solution in water (0.5 mL). After stirring for 30 min, the reaction was quenched by the addition of aqueous NH_4OH (2 mL) and the resulting mixture was extracted with 10% $MeOH/CH_2Cl_2$ (4 × 50 mL). The combined organic extracts were dried (Na_2SO_4) and concentrated under reduced pressure. PTLC (SiO_2, 95:5:3 EtOAc/MeOH/Et_3N) gave **40** (2.3 mg, 19%) as a white film, along with the

corresponding leurosidine analogue (1.9 mg, 16%) and anhydrovinblastine analogue (1.0 mg, 10%) also as white films: $[\alpha]_D^{22}$ +30 (*c* 0.80, $CHCl_3$); ^1H NMR (600 MHz, $CDCl_3$) δ 9.31 (s, 1H), 8.07 (s, 1H), 7.52 (d, *J* = 7.8 Hz, 1H), 7.36–7.31 (m, 4H), 7.29–7.26 (m, 1H), 7.16 (t, *J* = 7.4 Hz, 1H), 7.14–7.07 (m, 2H), 6.62 (s, 1H), 6.12 (s, 1H), 5.78–5.72 (m, 1H), 5.72–5.67 (m, 1H), 4.52–4.44 (m, 2H), 3.95 (t, *J* = 14.2 Hz, 1H), 3.78 (s, 3H), 3.77 (s, 3H), 3.59 (s, 3H), 3.52 (dd, *J* = 10.0, 2.5 Hz, 1H), 3.48 (s, 1H), 3.34 (dd, *J* = 16.6, 4.4 Hz, 1H), 3.23 (td, *J* = 9.5, 4.6 Hz, 1H), 3.15 (d, *J* = 14.7 Hz, 1H), 2.88–2.76 (m, 2H), 2.69 (s, 3H), 2.60–2.53 (m, 2H), 2.43 (s, 1H), 2.36 (q, *J* = 10.1 Hz, 1H), 2.30 (d, *J* = 15.0 Hz, 1H), 2.17–2.06 (m, 1H), 1.88 (ddd, *J* = 13.7, 11.0, 4.6 Hz, 1H), 1.68 (dq, *J* = 14.5, 7.3 Hz, 1H), 1.52–1.46 (m, 1H), 1.46–1.39 (m, 1H), 1.33 (d, *J* = 8.2 Hz, 2H), 1.28–1.19 (m, 1H), 0.89 (t, *J* = 7.5 Hz, 3H), 0.82 (t, *J* = 7.3 Hz, 3H); IR (neat) ν_{max} 3467, 2962, 2928, 2874, 1735, 1614, 1529, 1458, 1379, 1223, 1156, 1088, 1066, 745 cm^{-1}; ESI-TOF HRMS *m*/*z* 873.4792 ($M+H^+$, $C_{52}H_{64}N_4O_8$ requires 873.4797).

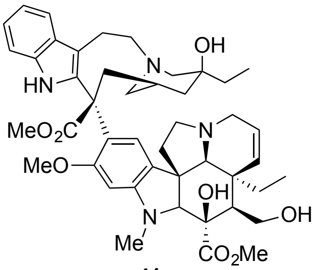

41

Compound 41. A solution of **40** (5 mg, 5.7 μmol) in MeOH (0.5 mL) was treated with CF_3COOH (0.01 mL) and Pd/C (1 mg, 10% on activated carbon, reduced dry powder) under Ar. The reaction mixture was degassed with hydrogen and stirred for 12 h under the atmosphere of hydrogen before the addition of saturated aqueous $NaHCO_3$. The resulting mixture was extracted with 10% MeOH/CH_2Cl_2 (4 × 2 mL). The combined organic extracts were dried (Na_2SO_4) and concentrated under reduced pressure. PTLC (SiO_2, 95:5:3 EtOAc/MeOH/Et_3N) gave **41** (3.2 mg, 71%) as a white film: $[\alpha]_D^{25}$ +49 (*c* 0.46, $CHCl_3$); ^1H NMR (600 MHz, $CDCl_3$) δ 10.02 (s, 1H), 8.05 (s, 1H), 7.52 (d, *J* = 7.9 Hz, 1H), 7.22–7.08 (m, 3H), 6.62 (s, 1H), 6.11 (s, 1H), 5.81 (ddd, *J* = 10.2, 4.6, 1.7 Hz, 1H), 5.73 (dt, *J* = 10.1, 2.0 Hz, 1H), 4.13–4.05 (m, 1H), 3.95 (t, *J* = 14.2 Hz, 1H), 3.90–3.81 (m, 4H), 3.78 (s, 3H), 3.59 (s, 3H), 3.49 (s, 1H), 3.40 (d, *J* = 13.8 Hz, 1H), 3.32 (ddd, *J* = 16.0, 4.7, 1.7 Hz, 2H), 3.30–3.22 (m, 2H), 3.14 (d, *J* = 13.9 Hz, 2H), 2.90–2.77 (m, 3H), 2.72 (s, 3H), 2.64 (s, 1H), 2.50–2.36 (m, 2H), 2.35–2.25 (m, 1H), 2.18 (dd, *J* = 5.7, 2.3 Hz, 1H), 2.07 (ddd, *J* = 14.4, 9.1, 5.8 Hz, 1H), 1.80 (ddd, *J* = 13.6, 11.4, 5.5 Hz, 1H), 1.58–1.50 (m, 1H), 1.48 (d, *J* = 14.7 Hz, 1H), 1.41 (dd, *J* = 14.3, 6.1 Hz, 1H), 1.32 (q, *J* = 7.5 Hz, 2H), 0.88 (q, *J* = 7.7 Hz, 6H); IR (neat) ν_{max} 3469, 2928, 2874, 1731, 1614, 1503, 1458, 1432, 1224, 1009, 746, 588 cm^{-1}; ESI-TOF HRMS *m*/*z* 783.4317 ($M+H^+$, $C_{45}H_{58}N_4O_8$ requires 783.4327).

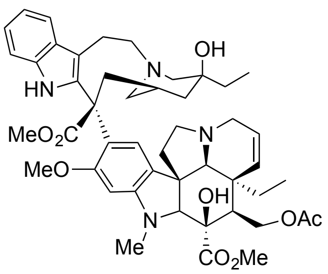

42

Compound 42. A solution of **41** (1.4 mg, 1.8 μmol) in anhydrous CH_2Cl_2 (0.2 mL) was treated with DMAP (0.4 mg, 3.6 μmol) and acetic anhydride (0.4 mg, 3.9 μmol). The resulting mixture was stirred for 30 min before the reaction was quenched with the addition of saturated aqueous $NaHCO_3$ (2 mL). The mixture was extracted with 10% $MeOH/CH_2Cl_2$ (4 × 2 mL) and the combined organic extracts were dried (Na_2SO_4) and concentrated under reduced pressure. PTLC (SiO_2, 95:5:3 EtOAc/MeOH/Et_3N) provided **42** (0.8 mg, 54%) as a white film: $[\alpha]_D^{23}$ +48 (*c* 0.13, $CHCl_3$); ^{1}H NMR (600 MHz, $CDCl_3$) δ 9.27 (s, 1H), 8.06 (s, 1H), 7.51 (d, *J* = 7.8 Hz, 1H), 7.21–7.08 (m, 3H), 6.12 (s, 1H), 5.81 (dd, *J* = 10.7, 4.1 Hz, 1H), 5.46 (dt, *J* = 10.2, 2.1 Hz, 1H), 4.33–4.19 (m, 2H), 3.92 (t, *J* = 15.6 Hz, 1H), 3.84 (s, 3H), 3.78 (s, 3H), 3.60 (s, 3H), 3.51 (s, 1H), 3.41–3.34 (m, 1H), 3.29 (td, *J* = 7.1, 5.4 Hz, 1H), 3.24 (td, *J* = 9.6, 4.6 Hz, 1H), 3.21–3.11 (m, 1H), 2.89–2.77 (m, 2H), 2.69 (s, 3H), 2.56 (d, *J* = 9.4 Hz, 1H), 2.53 (s, 1H), 2.41–2.28 (m, 2H), 2.19–2.11 (m, 1H), 2.06 (s, 3H), 1.90 (ddd, *J* = 13.8, 11.0, 4.5 Hz, 1H), 1.53 (dt, *J* = 14.2, 7.3 Hz, 1H), 1.40 (t, *J* = 7.3 Hz, 1H), 1.38–1.32 (m, 2H), 0.91 (t, *J* = 7.3 Hz, 3H), 0.82 (t, *J* = 7.3 Hz, 3H); IR (neat) ν_{max} 2925, 1731, 1614, 1501, 1459, 1432, 1223, 1142, 1040, 797, 745, 677, 666 cm^{-1}; ESI-TOF HRMS *m*/*z* 825.4437 ($M+H^+$, $C_{47}H_{60}N_4O_9$ requires 825.4433).

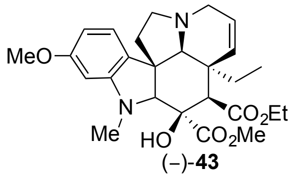

(–)-**43**

Compound (–)-43. Morpholine (9.4 μL, 109 μmol) and $Pd(PPh_3)_4$ (1.3 mg, 1.1 μmol) were added to a mixture of (–)-**23** (5.3 mg, 10.6 μmol) in THF (0.5 mL) and DMSO (50 μL). After stirring for 1 h, the solvent was removed under a stream of N_2. DMF (0.3 mL) was added followed by CsF (3.2 mg, 21.2 μmol), EtI (1.7 μL, 21.2 μmol). The mixture was stirred at room temperature for 1 h before the addition of saturated aqueous $NaHCO_3$ (2 mL). The reaction was extracted with EtOAc (4 × 2 mL). The organic layer was dried (Na_2SO_4) and concentrated under reduced pressure. PTLC (SiO_2, 50% hexanes/acetone) gave **43** (3.2 mg, 62%) as a white solid: $[\alpha]_D^{23}$ –4 (*c* 0.36, $CHCl_3$); ^{1}H NMR (600 MHz, $CDCl_3$) δ 9.48 (s, 1H), 6.88 (d, *J* = 8.1 Hz, 1H), 6.27 (dd, *J* = 8.2, 2.0 Hz, 1H), 6.04 (s, 1H), 5.89–5.82 (m, 1H), 5.18 (d, *J* = 10.0 Hz, 1H), 4.27–4.19 (m, 1H), 4.17–4.07 (m, 1H), 3.78 (s, 3H), 3.77 (s, 3H), 3.65 (s, 1H), 3.51–3.37 (m, 2H), 3.06 (s, 1H), 2.82 (d, *J* = 16.3 Hz, 1H), 2.76 (s, 1H), 2.67 (s, 3H), 2.60–2.50 (m, 1H), 2.34–2.21 (m, 2H), 1.81 (ddd, *J* = 14.4, 7.2, 1.9 Hz, 1H), 1.24 (t, *J* = 7.1 Hz, 3H), 1.04–0.95 (m, 1H), 0.59 (t, *J* = 7.3 Hz, 3H); ^{13}C NMR (151 MHz, $CDCl_3$) δ 173.5, 172.1, 161.1, 153.7,

131.2, 124.9, 124.8, 122.6, 104.3, 95.6, 83.0, 78.5, 65.7, 60.1, 55.3, 53.3, 52.2, 51.7, 51.2, 50.7, 44.6, 40.8, 38.4, 32.0, 14.2, 8.0; IR (neat) ν_{max} 2926, 1732, 1613, 1501, 1452, 1434, 1331, 1245, 1167, 1085, 1028, 819, 747 cm^{-1}; ESI-TOF HRMS *m/z* 471.2489 ($M+H^+$, $C_{26}H_{34}N_2O_6$ requires 471.2490).

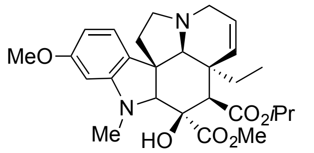

(–)-**44**

Compound (–)-44. Morpholine (9.0 μL, 104 μmol) and $Pd(PPh_3)_4$ (1.2 mg, 1.0 μmol) were added to a mixture of (–)-**23** (5.0 mg, 10.4 μmol) in THF (0.5 mL) and DMSO (50 μL). After stirring for 1 h, the solvent was removed under a stream of N_2. DMF (0.3 mL) was added followed by CsF (3.2 mg, 20.8 μmol), *i*PrI (2.1 μL, 20.8 μmol). The mixture was stirred at room temperature for 1 h before the addition of saturated aqueous $NaHCO_3$ (2 mL). The reaction was extracted with EtOAc (4 × 2 mL). The organic layer was dried (Na_2SO_4) and concentrated under reduced pressure. PTLC (SiO_2, 50% hexanes/acetone) gave **44** (3.0 mg, 60%) as a white solid: $[\alpha]_D^{23}$ –7.5 (*c* 0.50, $CHCl_3$); ^1H NMR (600 MHz, $CDCl_3$) δ 9.39 (s, 1H), 6.87 (d, *J* = 8.2 Hz, 1H), 6.26 (dd, *J* = 8.2, 2.3 Hz, 1H), 6.04 (d, *J* = 2.3 Hz, 1H), 5.83 (ddd, *J* = 10.1, 5.2, 1.6 Hz, 1H), 5.20 (dt, *J* = 10.0, 2.0 Hz, 1H), 5.02 (p, *J* = 6.3 Hz, 1H), 3.77 (d, *J* = 1.4 Hz, 6H), 3.62 (s, 1H), 3.47–3.42 (m, 1H), 3.39 (td, *J* = 9.3, 4.6 Hz, 1H), 3.01 (s, 1H), 2.82–2.76 (m, 1H), 2.73 (s, 1H), 2.67 (s, 3H), 2.52 (td, *J* = 10.4, 7.3 Hz, 1H), 2.32–2.19 (m, 2H), 1.81 (dq, *J* = 14.7, 7.1 Hz, 1H), 1.23 (d, *J* = 6.3 Hz, 3H), 1.21 (d, *J* = 6.3 Hz, 3H), 0.98 (dq, *J* = 14.5, 7.2 Hz, 1H), 0.58 (t, *J* = 7.3 Hz, 3H); ^{13}C NMR (151 MHz, $CDCl_3$) δ 173.5, 171.5, 161.0, 153.7, 131.2, 125.1, 124.7, 122.6, 104.2, 95.5, 83.1, 78.5, 67.4, 65.7, 55.3, 53.2, 52.1, 51.6, 51.2, 50.7, 44.6, 40.8, 38.4, 32.1, 21.8, 21.7, 8.0; IR (neat) ν_{max} 2969, 1734, 1613, 1502, 1454, 1247, 1173, 1142, 1108, 1087, 1042, 752 cm^{-1}; ESI-TOF HRMS *m/z* 485.2647 ($M+H^+$, $C_{27}H_{36}N_2O_6$ requires 485.2646).

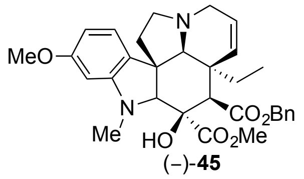

(–)-**45**

Compound (–)-45. Morpholine (9.4 μL, 109 μmol) and $Pd(PPh_3)_4$ (1.3 mg, 1.1 μmol) were added to a mixture of (–)-**23** (5.3 mg, 10.6 μmol) in THF (0.5 mL) and DMSO (50 μL). After stirring for 1 h, the solvent was removed under a stream of N_2. DMF (0.3 mL) was added followed by CsF (3.2 mg, 21.2 μmol), BnBr (2.5 μL, 21.2 μmol). The mixture was stirred at room temperature for 1 h before the addition of saturated aqueous $NaHCO_3$ (2 mL). The reaction was extracted with EtOAc (4 × 2 mL). The organic layer was dried (Na_2SO_4) and concentrated under reduced pressure. PTLC (SiO_2, 50% hexanes/acetone) gave **45** (4.7 mg, 80%) as a white solid: $[\alpha]_D^{23}$ –17 (*c* 0.5, $CHCl_3$); ^1H NMR (600 MHz, $CDCl_3$) δ 9.50 (s, 1H), 7.40–7.36 (m, 2H), 7.35–7.29 (m, 2H), 7.29–7.27 (m, 1H), 6.86 (d, *J* = 8.2 Hz, 1H), 6.26 (dd, *J* = 8.1, 2.2 Hz, 1H), 6.03 (d, *J* = 2.3 Hz, 1H), 5.76 (ddd, *J* = 10.1, 5.1, 1.6 Hz, 1H), 5.25 (d, *J* = 12.4 Hz, 1H), 5.05 (d, *J* =

12.4 Hz, 1H), 5.01 (ddd, J = 10.1, 2.6, 1.5 Hz, 1H), 3.76 (s, 3H), 3.65–3.61 (m, 4H), 3.46–3.35 (m, 2H), 3.12 (s, 1H), 2.79 (dt, J = 16.0, 2.1 Hz, 1H), 2.71 (s, 1H), 2.65 (s, 3H), 2.53 (td, J = 10.4, 7.2 Hz, 1H), 2.33–2.20 (m, 2H), 1.78 (dq, J = 14.7, 7.4 Hz, 1H), 0.97 (dq, J = 14.5, 7.3 Hz, 1H), 0.54 (t, J = 7.3 Hz, 3H); ^{13}C NMR (151 MHz, $CDCl_3$) δ 173.4, 171.9, 161.0, 153.6, 136.0, 131.0, 128.6, 128.3, 127.9, 124.9, 124.8, 122.6, 104.3, 95.5, 83.0, 78.4, 65.8, 65.7, 55.3, 53.2, 52.1, 51.7, 51.2, 50.7, 44.5, 40.9, 38.4, 32.0, 7.9; IR (neat) ν_{max} 2956, 1733, 1614, 1501, 1458, 1249, 1167, 1086, 1043, 750 cm^{-1}; ESI-TOF HRMS *m/z* 533.2647 ($M+H^+$, $C_{31}H_{36}N_2O_6$ requires 533.2646).

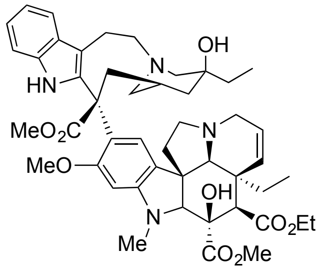

46

Compound 46. (–)-**43** (3.2 mg, 6.8 μmol) and catharanthine sulfate (3.0 mg, 6.90 μmol) were combined with TFE (35 μL), aqueous 0.1 N HCl (0.18 mL) and water (0.18 mL) in a 2-dram vial. The vial was fitted with a rubber septum and flushed with Ar. $FeCl_3 \cdot 6H_2O$ (9 mg, 33.3 μmol) was added producing a bright yellow precipitate. The mixture was stirred for 2 h during which time the precipitate re-dissolved leaving a clear, yellow solution. Meanwhile, a suspension of $Fe_2(ox)_3 \cdot 6H_2O$ (33.2 mg, 68.6 μmol) in water (30 mL) was stirred for 2 h, by which time the $Fe_2(ox)_3 \cdot 6H_2O$ was completely dissolved. This mixture was cooled to 0 °C and air was bubbled vigorously through the solution for 15–20 min. The $FeCl_3$ coupling mixture was transferred to the $Fe_2(ox)_3/O_2$ solution by pipette, then $NaBH_4$ (5.2 mg, 163 μmol) was added as a solution in water (0.3 mL). After stirring for 30 min, the reaction was quenched by the addition of aqueous NH_4OH (5 mL) and the resulting mixture was extracted with 10% $MeOH/CH_2Cl_2$ (4 × 30 mL). The combined organic extracts were dried (Na_2SO_4) and concentrated under reduced pressure. PTLC (SiO_2, 97:3:3 EtOAc/MeOH/Et_3N) gave **46** (1.8 mg, 32%) as a colorless film, along with the corresponding leurosidine analogue (1.1 mg, 20%) and anhydrovinblastine analogue (0.9 mg, 17%) also as colorless films. For **46**: $[\alpha]_D^{21}$ +44 (*c* 0.18, $CHCl_3$); ^1H NMR (600 MHz, $CDCl_3$) δ 9.71 (s, 1H), 8.05 (s, 1H), 7.51 (d, J = 8.0 Hz, 1H), 7.21–7.07 (m, 3H), 6.60 (s, 1H), 6.08 (d, J = 1.5 Hz, 1H), 5.83 (dd, J = 10.3, 5.0 Hz, 1H), 5.24–5.13 (m, 1H), 4.34–4.22 (m, 1H), 4.22–4.11 (m, 1H), 3.94 (t, J = 14.3 Hz, 1H), 3.79 (s, 3H), 3.78 (s, 3H), 3.65–3.59 (m, 4H), 3.50–3.40 (m, 1H), 3.37–3.30 (m, 2H), 3.30–3.22 (m, 2H), 3.22–3.12 (m, 1H), 3.07 (s, 1H), 2.86 (s, 1H), 2.79 (d, J = 16.2 Hz, 1H), 2.76–2.65 (m, 5H), 2.51–2.42 (m, 2H), 2.31 (d, J = 15.1 Hz, 1H), 2.14 (dt, J = 14.9, 8.1 Hz, 1H), 2.02 (dq, J = 14.7, 7.5 Hz, 1H), 1.81 (td, J = 12.5, 5.0 Hz, 1H), 1.56–1.47 (m, 1H), 1.47–1.39 (m, 1H), 1.34 (s, 2H), 1.33–1.22 (m, 5H), 0.93–0.82 (m, 6H); IR (neat) ν_{max} 2928, 1729, 1665, 1613, 1503, 1459, 1432, 1333, 1256, 1178, 1143, 1093, 1021, 802, 745 cm^{-1}; ESI-TOF HRMS *m/z* 825.4421 ($M+H^+$, $C_{47}H_{60}N_4O_9$ requires 825.4433).

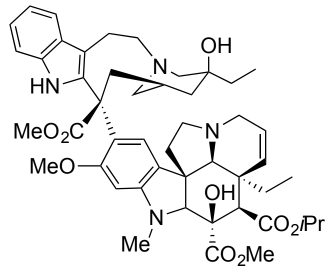

47

Compound 47. (–)-**44** (3 mg, 6.2 μmol) and catharanthine sulfate (2.7 mg, 6.2 μmol) were combined with TFE (32 μL), aqueous 0.1 N HCl (0.16 mL) and water (0.16 mL) in a 2-dram vial. The vial was fitted with a rubber septum and flushed with Ar. $FeCl_3 \cdot 6H_2O$ (8.4 mg, 31.1 μmol) was added producing a bright yellow precipitate. The mixture was stirred for 2 h during which time the precipitate re-dissolved leaving a clear, yellow solution. Meanwhile, a suspension of $Fe_2(ox)_3 \cdot 6H_2O$ (30.2 mg, 62.4 μmol) in water (27 mL) was stirred for 2 h, by which time the $Fe_2(ox)_3 \cdot 6H_2O$ was completely dissolved. This mixture was cooled to 0 °C and air was bubbled vigorously through the solution for 15–20 min. The $FeCl_3$ coupling mixture was transferred to the $Fe_2(ox)_3/O_2$ solution by pipette, then $NaBH_4$ (4 mg, 126 μmol) was added as a solution in water (0.3 mL). After stirring for 30 min, the reaction was quenched by the addition of aqueous NH_4OH (5 mL) and the resulting mixture was extracted with 10% MeOH/CH_2Cl_2 (4 × 30 mL). The combined organic extracts were dried (Na_2SO_4) and concentrated under reduced pressure. PTLC (SiO_2, 97:3:3 EtOAc/MeOH/Et_3N) gave **47** (1.6 mg, 30%) as a colorless film, along with the corresponding leurosidine analogue (0.9 mg, 18%) and anhydrovinblastine analogue (0.8 mg, 15%) also as colorless films. For **47**: $[\alpha]_D^{23}$ +34 (*c* 0.20, $CHCl_3$); ^1H NMR (600 MHz, $CDCl_3$) δ 9.66 (s, 1H), 8.05 (s, 1H), 7.51 (d, *J* = 7.9 Hz, 1H), 7.20–7.08 (m, 3H), 6.59 (s, 1H), 6.09 (s, 1H), 5.81 (ddd, *J* = 10.2, 5.1, 1.6 Hz, 1H), 5.20 (d, *J* = 10.1 Hz, 1H), 5.07 (p, *J* = 6.3 Hz, 1H), 3.94 (t, *J* = 14.1 Hz, 1H), 3.78 (s, 6H), 3.72 (d, *J* = 5.9 Hz, 1H), 3.63–3.58 (m, 4H), 3.51–3.43 (m, 1H), 3.43–3.35 (m, 1H), 3.34–3.27 (m, 2H), 3.27–3.21 (m, 2H), 3.21–3.12 (m, 2H), 3.03 (s, 1H), 2.93–2.83 (m, 2H), 2.79 (d, *J* = 16.0 Hz, 1H), 2.72–2.65 (m, 4H), 2.54–2.47 (m, 1H), 2.44 (td, *J* = 10.5, 5.9 Hz, 1H), 2.31 (d, *J* = 15.5 Hz, 1H), 2.18–2.09 (m, 1H), 2.07–1.99 (m, 1H), 1.80 (ddd, *J* = 13.8, 11.4, 5.0 Hz, 1H), 1.54–1.46 (m, 1H), 1.43 (dt, *J* = 14.4, 7.3 Hz, 1H), 1.39–1.31 (m, 2H), 1.27 (d, *J* = 6.3 Hz, 3H), 1.25 (d, *J* = 6.3 Hz, 4H), 0.88 (dt, *J* = 11.7, 7.4 Hz, 6H); IR (neat) ν_{max} 2963, 1732, 1614, 1504, 1460, 1433, 1249, 1181, 1143, 1107, 1039, 806, 749 cm^{-1}; ESI-TOF HRMS *m*/*z* 839.4590 ($M+H^+$, $C_{48}H_{62}N_4O_9$ requires 839.4589).

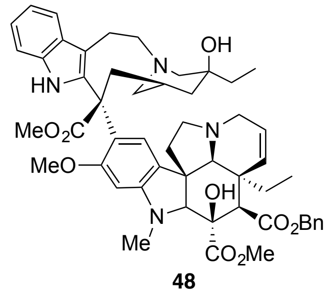

48

Compound 48. (–)-**45** (4.5 mg, 8.4 μmol) and catharanthine sulfate (3.6 mg, 8.4 μmol) were combined with TFE (36 μL), aqueous 0.1 N HCl (0.18 mL) and water (0.18 mL) in a 2-dram vial. The vial was fitted with a rubber septum and flushed with Ar. $FeCl_3 \cdot 6H_2O$ (11.4 mg, 42 μmol) was added producing a bright yellow precipitate. The mixture was stirred for 2 h during which time the precipitate re-dissolved leaving a clear, yellow solution. Meanwhile, a suspension of $Fe_2(ox)_3 \cdot 6H_2O$ (40.5 mg, 84 μmol) in water (37 mL) was stirred for 2 h, by which time the $Fe_2(ox)_3 \cdot 6H_2O$ was completely dissolved. This mixture was cooled to 0 °C and air was bubbled vigorously through the solution for 15–20 min. The $FeCl_3$ coupling mixture was transferred to the $Fe_2(ox)_3/O_2$ solution by pipette, then $NaBH_4$ (5.4 mg, 168 μmol) was added as a solution in water (0.3 mL). After stirring for 30 min, the reaction was quenched by the addition of aqueous NH_4OH (5 mL) and the resulting mixture was extracted with 10% $MeOH/CH_2Cl_2$ (4 × 30 mL). The combined organic extracts were dried (Na_2SO_4) and concentrated under reduced pressure. PTLC (SiO_2, 97:3:3 EtOAc/MeOH/Et_3N) gave **48** (2.3 mg, 31%) as a colorless film, along with the corresponding leurosidine analogue (1.5 mg, 20%) and anhydrovinblastine analogue (1.3 mg, 18%) also as colorless films. For **48**: $[\alpha]_D^{21}$ +22 (*c* 0.10, $CHCl_3$); ^1H NMR (600 MHz, $CDCl_3$) δ 9.71 (s, 1H), 8.04 (s, 1H), 7.50 (d, *J* = 7.9 Hz, 1H), 7.44–7.40 (m, 2H), 7.38–7.33 (m, 2H), 7.33–7.29 (m, 1H), 7.19–7.07 (m, 3H), 6.58 (s, 1H), 6.07 (s, 1H), 5.75 (dd, *J* = 10.5, 4.9 Hz, 1H), 5.31 (d, *J* = 12.3 Hz, 1H), 5.07 (d, *J* = 12.3 Hz, 1H), 5.01 (dt, *J* = 9.8, 1.9 Hz, 1H), 3.94 (t, *J* = 14.2 Hz, 1H), 3.77 (s, 3H), 3.65 (s, 3H), 3.62 (s, 1H), 3.60 (s, 3H), 3.35–3.21 (m, 3H), 3.22–3.13 (m, 2H), 3.12 (s, 1H), 2.87 (s, 2H), 2.78 (d, *J* = 16.1 Hz, 1H), 2.68 (s, 3H), 2.65 (s, 1H), 2.45 (d, *J* = 7.6 Hz, 1H), 2.31 (d, *J* = 15.0 Hz, 1H), 2.14 (ddd, *J* = 14.6, 9.2, 6.1 Hz, 1H), 2.00 (dt, *J* = 14.2, 7.2 Hz, 1H), 1.81 (ddd, *J* = 13.7, 11.3, 5.0 Hz, 1H), 1.55–1.46 (m, 1H), 1.45 (d, *J* = 14.3 Hz, 1H), 1.37 (d, *J* = 23.3 Hz, 2H), 1.20 (dt, *J* = 13.7, 6.8 Hz, 1H), 0.89 (t, *J* = 7.5 Hz, 3H), 0.82 (t, *J* = 7.4 Hz, 3H); IR (neat) ν_{max} 2923, 1731, 1613, 1459, 1258, 1168, 1038, 801, 747 cm^{-1}; ESI-TOF HRMS *m*/*z* 887.4588 ($M+H^+$, $C_{52}H_{62}N_4O_9$ requires 887.4589).

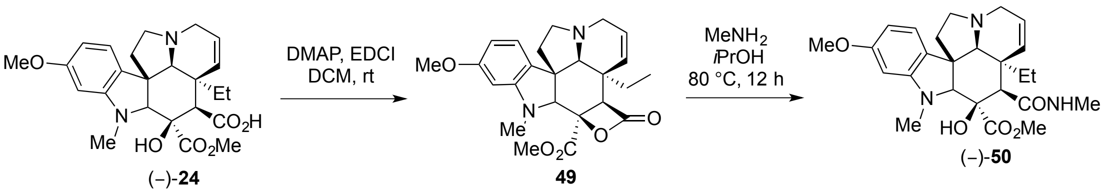

Compound (–)-50. EDCI (3.6 mg, 23 μmol) and DMAP (2.8 mg, 23 μmol) were added to a solution of (–)-**24** (5.1 mg, 11.5 μmol) in CH_2Cl_2 (0.5 mL). After stirring for 12 h at room temperature, the solvent was removed under a stream of N_2. The residue was treated with *i*PrOH (0.4 mL), $NaHCO_3$ (29 mg, 345 μmol) and $MeNH_2 \cdot HCl$ (15.5 mg, 230 μmol). The mixture was heated at 80 °C for 12 h, before the reaction mixture was filtered through a short pad of Celite and the solvent was removed under a stream of N_2. PTLC (SiO_2, 75% EtOAc/hexanes) provided (–)-**50** (3.2 mg, 61%) as a colorless oil: $[\alpha]_D^{23}$ –51 (*c* 0.53, $CHCl_3$); ^1H NMR (600 MHz, $CDCl_3$) δ 10.36 (s, 1H), 7.30 (d, *J* = 5.1 Hz, 1H), 6.83 (d, *J* = 8.3 Hz, 1H), 6.24 (dd, *J* = 8.2, 2.3 Hz, 1H), 5.98 (d, *J* = 2.3 Hz, 1H), 5.76 (ddd, *J* = 10.1, 5.0, 1.7 Hz, 1H), 5.22 (dt, *J* = 10.1, 2.1 Hz, 1H), 3.75 (d, *J* = 3.0 Hz, 6H), 3.58 (s, 1H), 3.43–3.35 (m, 2H), 2.88 (s, 1H), 2.84 (dt, *J* = 16.1, 2.1

Hz, 1H), 2.73 (s, 1H), 2.67 (d, *J* = 4.8 Hz, 3H), 2.66 (s, 3H), 2.54 (q, *J* = 9.2 Hz, 1H), 2.22 (dd, *J* = 9.0, 6.9 Hz, 2H), 1.81 (dq, *J* = 14.7, 7.4 Hz, 1H), 0.97 (dq, *J* = 14.5, 7.2 Hz, 1H), 0.54 (t, *J* = 7.3 Hz, 3H); ^{13}C NMR (151 MHz, $CDCl_3$) δ 173.2, 173.1, 161.1, 153.5, 132.7, 124.3, 123.4, 122.4, 104.3, 95.4, 82.3, 78.6, 65.0, 55.3, 53.1, 52.5, 52.2, 50.9, 50.8, 44.2, 40.8, 38.4, 32.0, 25.5, 7.8; IR (neat) ν_{max} 3350, 2961, 1738, 1659, 1615, 1539, 1502, 1454, 1434, 1244, 1087, 1041, 750 cm^{-1}; ESI-TOF HRMS *m*/*z* 456.2493 (M+H$^+$, $C_{25}H_{33}N_3O_5$ requires 456.2493).

For Compound **49**: ^{1}H NMR (600 MHz, $CDCl_3$) δ 7.00 (d, *J* = 8.2 Hz, 1H), 6.38 (dd, *J* = 8.2, 2.3 Hz, 1H), 6.12 (d, *J* = 2.3 Hz, 1H), 5.92 (ddd, *J* = 9.8, 4.4, 1.9 Hz, 1H), 5.42 (dt, *J* = 9.8, 2.3 Hz, 1H), 3.89 (s, 3H), 3.77 (s, 3H), 3.77 (s, 1H), 3.70 (s, 1H), 3.43 (ddd, *J* = 16.6, 4.4, 1.9 Hz, 1H), 3.32–3.24 (m, 1H), 2.81 (dd, *J* = 16.6, 2.3 Hz, 1H), 2.69 (s, 3H), 2.49 (s, 1H), 2.43 (ddd, *J* = 10.8, 8.9, 7.5 Hz, 1H), 2.34 (ddd, *J* = 13.0, 7.5, 1.6 Hz, 1H), 2.23 (ddd, *J* = 13.0, 10.6, 7.8 Hz, 1H), 1.64 (dt, *J* = 14.3, 7.3 Hz, 1H), 1.24 (dq, *J* = 14.7, 7.6 Hz, 1H), 0.41 (t, *J* = 7.4 Hz, 3H); ESI-TOF HRMS *m*/*z* 425.2069 (M+H$^+$, $C_{24}H_{28}N_2O_5$ requires 425.2071). Note: Due to the stability of **49**, only a small amount of pure compound was obtained through PTLC purification (SiO_2, 50% EtOAc/hexanes).

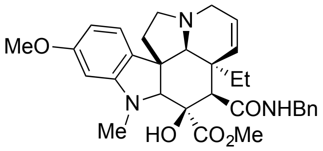

(–)-**51**

Compound (–)-51. EDCI (2.8 mg, 18 μmol) and DMAP (2.2 mg, 18 μmol) were added to a solution of (–)-**24** (4 mg, 9 μmol) in CH_2Cl_2 (0.5 mL). After stirring for 12 h at room temperature, the solvent was removed under a stream of N_2 to give crude **49**. The residue was treated with *i*PrOH (0.3 mL) and $BnNH_2$ (0.1 mL). The mixture was heated at 60 °C for 12 h, before the solvent was removed under a stream of N_2. PTLC (SiO_2, 50% hexanes/acetone) provided (–)-**51** (2.4 mg, 50%) as an off-white solid: $[\alpha]_D^{26}$ –216 (*c* 0.36, $CHCl_3$); ^{1}H NMR (600 MHz, $CDCl_3$) δ 10.47 (s, 1H), 7.84 (t, *J* = 5.8 Hz, 1H), 7.33–7.26 (m, 2H), 7.25–7.19 (m, 3H), 6.85 (d, *J* = 8.2 Hz, 1H), 6.26 (dd, *J* = 8.2, 2.3 Hz, 1H), 6.00 (d, *J* = 2.3 Hz, 1H), 5.76–5.68 (m, 1H), 5.27 (dt, *J* = 10.2, 2.0 Hz, 1H), 4.51 (dd, *J* = 14.8, 6.4 Hz, 1H), 4.19 (dd, *J* = 14.8, 5.4 Hz, 1H), 3.76 (s, 3H), 3.58 (s, 3H), 3.57 (s, 1H), 3.41–3.33 (m, 2H), 2.94 (s, 1H), 2.83 (dt, *J* = 16.0, 2.2 Hz, 1H), 2.73 (s, 1H), 2.65 (s, 3H), 2.55 (td, *J* = 10.0, 7.5 Hz, 1H), 2.26–2.19 (m, 2H), 1.84 (dq, *J* = 14.7, 7.4 Hz, 1H), 0.99 (dq, *J* = 14.5, 7.2 Hz, 1H), 0.55 (t, *J* = 7.3 Hz, 3H); ^{13}C NMR (151 MHz, $CDCl_3$) δ 173.0, 172.2, 161.2, 153.6, 138.7, 132.8, 128.3, 127.9, 126.9, 124.3, 123.5, 122.5, 104.3, 95.5, 82.5, 78.5, 65.1, 55.3, 53.1, 52.4, 52.3, 50.9, 50.8, 44.2, 42.7, 40.9, 38.4, 32.2, 7.9; IR (neat) ν_{max} 3340, 2929, 1737, 1658, 1614, 1531, 1501, 1454, 1244, 1167, 1086, 1040, 801, 750 cm^{-1}; ESI-TOF HRMS *m*/*z* 532.2797 (M+H$^+$, $C_{31}H_{37}N_3O_5$ requires 532.2806).

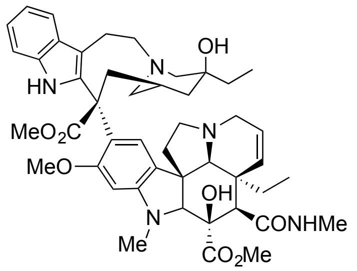

52

Compound 52. (–)-**50** (3.2 mg, 7.0 μmol) and catharanthine sulfate (3.1 mg, 7.0 μmol) were combined with TFE (30 μL), aqueous 0.1 N HCl (0.15 mL) and water (0.15 mL) in a 2-dram vial. The vial was fitted with a rubber septum and flushed with Ar. $FeCl_3 \cdot 6H_2O$ (9.5 mg, 35 μmol) was added producing a bright yellow precipitate. The mixture was stirred for 2 h during which time the precipitate re-dissolved leaving a clear, yellow solution. Meanwhile, a suspension of $Fe_2(ox)_3 \cdot 6H_2O$ (34 mg, 70 μmol) in water (31 mL) was stirred for 2 h, by which time the $Fe_2(ox)_3 \cdot 6H_2O$ was completely dissolved. This mixture was cooled to 0 °C and air was bubbled vigorously through the solution for 15–20 min. The $FeCl_3$ coupling mixture was transferred to the $Fe_2(ox)_3/O_2$ solution by pipette, then $NaBH_4$ (4.5 mg, 140 μmol) was added as a solution in water (0.3 mL). After stirring for 30 min, the reaction was quenched by the addition of aqueous NH_4OH (5 mL) and the resulting mixture was extracted with 10% $MeOH/CH_2Cl_2$ (4 × 30 mL). The combined organic extracts were dried (Na_2SO_4) and concentrated under reduced pressure. PTLC (SiO_2, 95:5:3 EtOAc/MeOH/Et_3N) gave **52** (1.5 mg, 26%) as a colorless film, along with the corresponding leurosidine analogue (0.8 mg, 14%) and anhydrovinblastine analogue (0.4 mg, 7%) also as colorless films. For **52**: $[\alpha]_D^{24}$ +17 (*c* 0.13, $CHCl_3$); ^1H NMR (600 MHz, $CDCl_3$) δ 10.57 (s, 1H), 8.07 (s, 1H), 7.53 (d, *J* = 7.9 Hz, 1H), 7.37–7.32 (m, 1H), 7.20–7.09 (m, 3H), 6.62 (s, 1H), 6.08 (s, 1H), 5.78 (ddd, *J* = 10.2, 5.0, 1.6 Hz, 1H), 5.26 (dt, *J* = 10.0, 2.0 Hz, 1H), 3.97 (t, *J* = 14.2 Hz, 1H), 3.81 (d, *J* = 1.3 Hz, 6H), 3.76 (s, 1H), 3.63 (s, 3H), 3.59 (s, 1H), 3.47–3.35 (m, 2H), 3.35–3.26 (m, 2H), 3.26–3.13 (m, 2H), 2.95–2.82 (m, 4H), 2.73 (d, *J* = 4.4 Hz, 7H), 2.56–2.46 (m, 2H), 2.34 (d, *J* = 14.8 Hz, 1H), 2.12 (ddd, *J* = 14.5, 9.2, 5.5 Hz, 1H), 2.06 (dt, *J* = 14.3, 7.2 Hz, 1H), 1.83 (ddd, *J* = 13.8, 11.6, 5.6 Hz, 1H), 1.53 (d, *J* = 14.4 Hz, 1H), 1.44 (dd, *J* = 14.4, 6.2 Hz, 1H), 1.40–1.32 (m, 2H), 1.32–1.24 (m, 2H), 0.91 (t, *J* = 7.5 Hz, 3H), 0.87 (t, *J* = 7.4 Hz, 3H); IR (neat) ν_{max} 3341, 2935, 1736, 1655, 1615, 1503, 1460, 1433, 1245, 1141, 1092, 1038, 749 cm^{-1}; ESI-TOF HRMS *m/z* 810.4438 ($M+H^+$, $C_{48}H_{62}N_4O_9$ requires 810.4436).

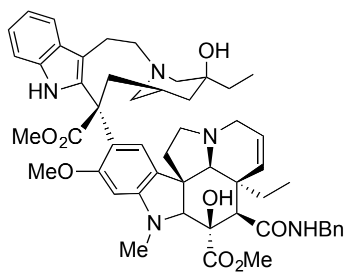

53

Compound 53. (–)-**51** (3 mg, 5.6 μmol) and catharanthine sulfate (2.4 mg, 5.6 μmol) were combined with TFE (24 μL), aqueous 0.1 N HCl (0.12 mL) and water (0.12 mL) in a 2-dram vial. The vial was fitted with a rubber septum and flushed with Ar. $FeCl_3 \cdot 6H_2O$ (7.6 mg, 28 μmol) was added producing a bright yellow precipitate. The mixture was stirred for 2 h during which time the precipitate re-dissolved leaving a clear, yellow solution. Meanwhile, a suspension of $Fe_2(ox)_3 \cdot 6H_2O$ (27 mg, 56 μmol) in water (25 mL) was stirred for 2 h, by which time the $Fe_2(ox)_3 \cdot 6H_2O$ was completely dissolved. This mixture was cooled to 0 °C and air was bubbled vigorously through the solution for 15–20 min. The $FeCl_3$ coupling mixture was transferred to the $Fe_2(ox)_3/O_2$ solution by pipette, then $NaBH_4$ (3.6 mg, 113 μmol) was added as a solution in water (0.3 mL). After stirring for 30 min, the reaction was quenched by the addition of aqueous NH_4OH (5 mL) and the resulting mixture was extracted with 10% $MeOH/CH_2Cl_2$ (4 × 30 mL). The combined organic extracts were dried (Na_2SO_4) and concentrated under reduced pressure. PTLC (SiO_2, 97:3:3 EtOAc/MeOH/Et_3N) gave **53** (1.6 mg, 32%) as a colorless film, along with the corresponding leurosidine analogue (0.8 mg, 16%) and anhydrovinblastine analogue (0.9 mg, 18%) also as colorless films. For **53**: $[\alpha]_D^{24}$ +3 (*c* 0.15, $CHCl_3$); ^1H NMR (600 MHz, $CDCl_3$) δ 10.67 (s, 1H), 8.08 (s, 1H), 7.85 (t, *J* = 5.9 Hz, 1H), 7.53 (d, *J* = 7.9 Hz, 1H), 7.41–7.21 (m, 6H), 7.22–7.09 (m, 3H), 6.61 (s, 1H), 6.08 (s, 1H), 5.73–5.65 (m, 1H), 5.29 (dt, *J* = 10.1, 2.0 Hz, 1H), 4.56 (dd, *J* = 14.8, 6.4 Hz, 1H), 4.23 (dd, *J* = 14.8, 5.3 Hz, 1H), 4.01–3.92 (m, 1H), 3.81 (s, 3H), 3.63 (d, *J* = 6.8 Hz, 6H), 3.57 (s, 1H), 3.49–3.35 (m, 2H), 3.34–3.14 (m, 3H), 2.96 (s, 1H), 2.93–2.84 (m, 2H), 2.71 (s, 3H), 2.51 (td, *J* = 10.7, 10.1, 5.2 Hz, 2H), 2.35 (d, *J* = 15.2 Hz, 1H), 2.15–2.02 (m, 2H), 1.84 (ddd, *J* = 13.8, 11.5, 5.6 Hz, 1H), 1.57–1.51 (m, 1H), 1.45 (dd, *J* = 14.4, 6.2 Hz, 1H), 1.41–1.32 (m, 2H), 1.32–1.23 (m, 2H), 1.20–1.14 (m, 2H), 0.92 (t, *J* = 7.5 Hz, 3H), 0.87 (t, *J* = 7.4 Hz, 3H); IR (neat) ν_{max} 2921, 1736, 1654, 1458, 1258, 1088, 1020, 798, 744, 698, 663 cm^{-1}; ESI-TOF HRMS *m*/*z* 886.4742 ($M+H^+$, $C_{52}H_{63}N_5O_8$ requires 886.4749).

Experimental procedure for the tubulin binding assay

To 850 μL of Buffer (PEM + 1 mM GTP) was added 100 μL of tubulin (1 mg/mL) and 25 μL BODIPY-VBL (72 μM solution). After incubating at 37 °C for 15 min, 25 μL of competitive ligand (720 μM solution) was added and the solution was incubated again for an additional 60 min at 37 °C. Final concentrations: [tubulin] = 0.91 μM, [BODIPY-VBL] = 1.8 μM, [competitive ligand] = 18 μM. The solution was then transferred (4 × 100 μL aliquots of each) into the wells of a 96-well plate and the fluorescence intensity (FI) (Ex 480 nm, Em 514 nm, Cutoff 495 nm) was measured in a plate reader that was preset to 37 °C. Two controls were run (solution with BODIPY-VBL but no protein or competitive ligand and a solution with BODIPY-VBL and tubulin but no competitive ligand) in order to determine minimum and maximum fluorescence intensities. Percent displacement was then determined using the formula: % displacement = (Control 2 FI – Experimental FI)/(Control 2 – Control 1) ×100.

References:

S1. Ishikawa, H.; Elliott, G. I.; Velcicky, J.; Choi, Y.; Boger, D. L. *J. Am. Chem. Soc.* **2006**, *128*, 10596.

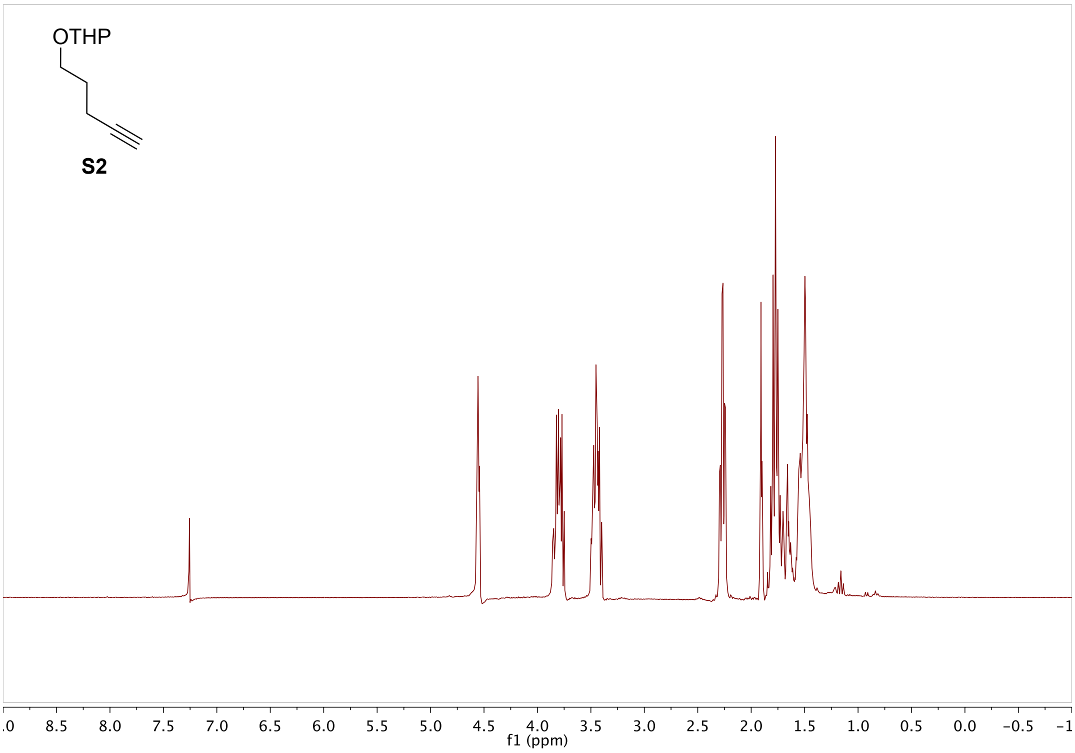

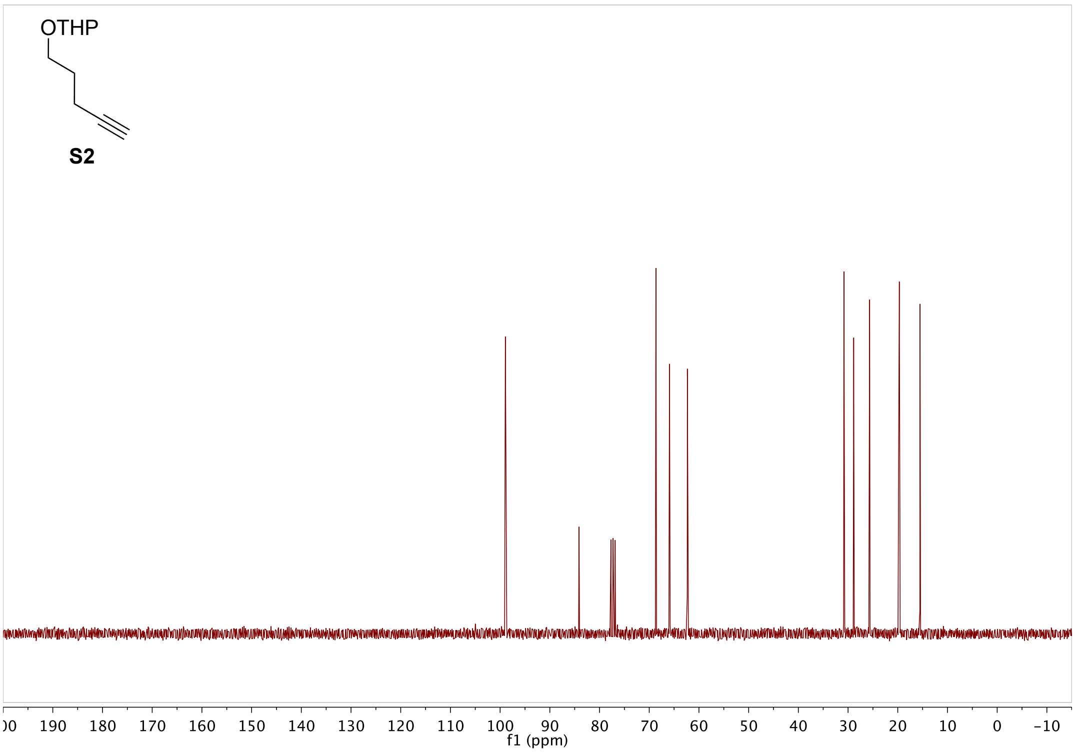

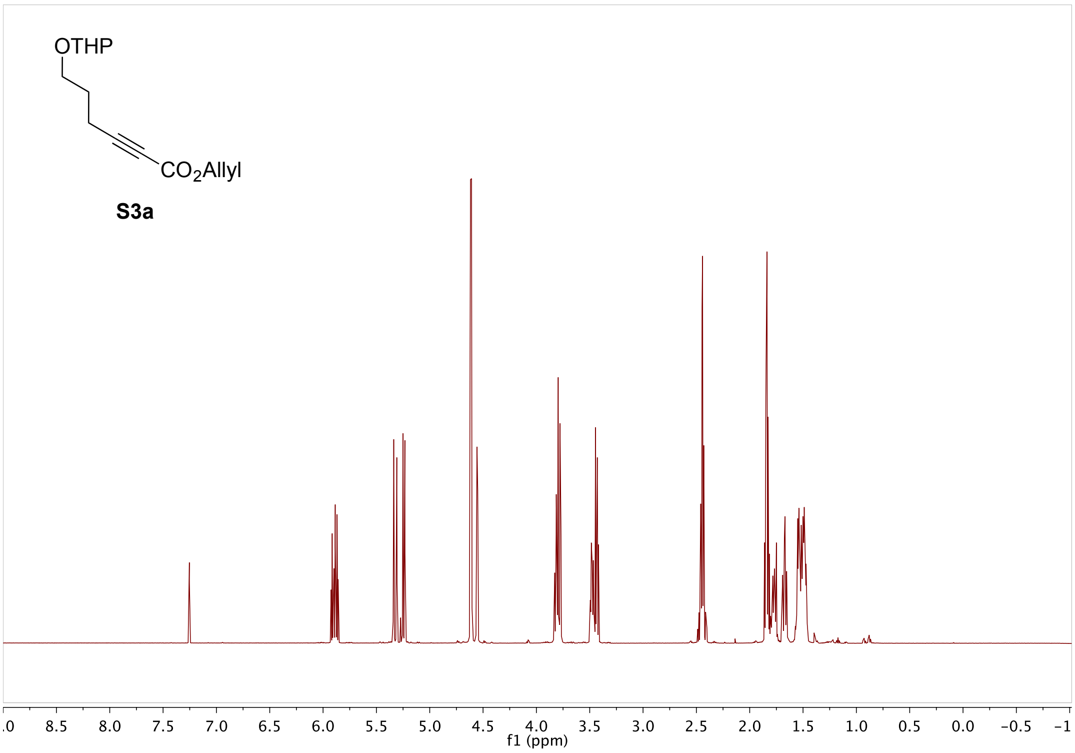
OTHP
CO2Allyl
S3a
f1 (ppm)

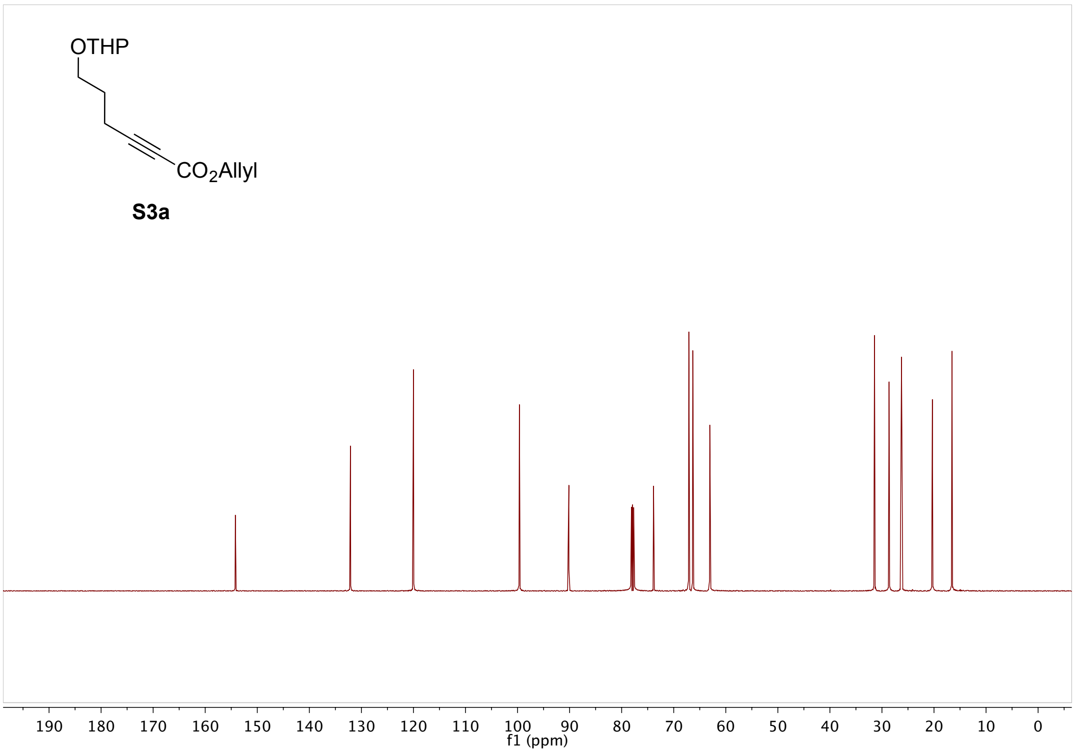
OTHP
CO2Allyl
S3a
f1 (ppm)

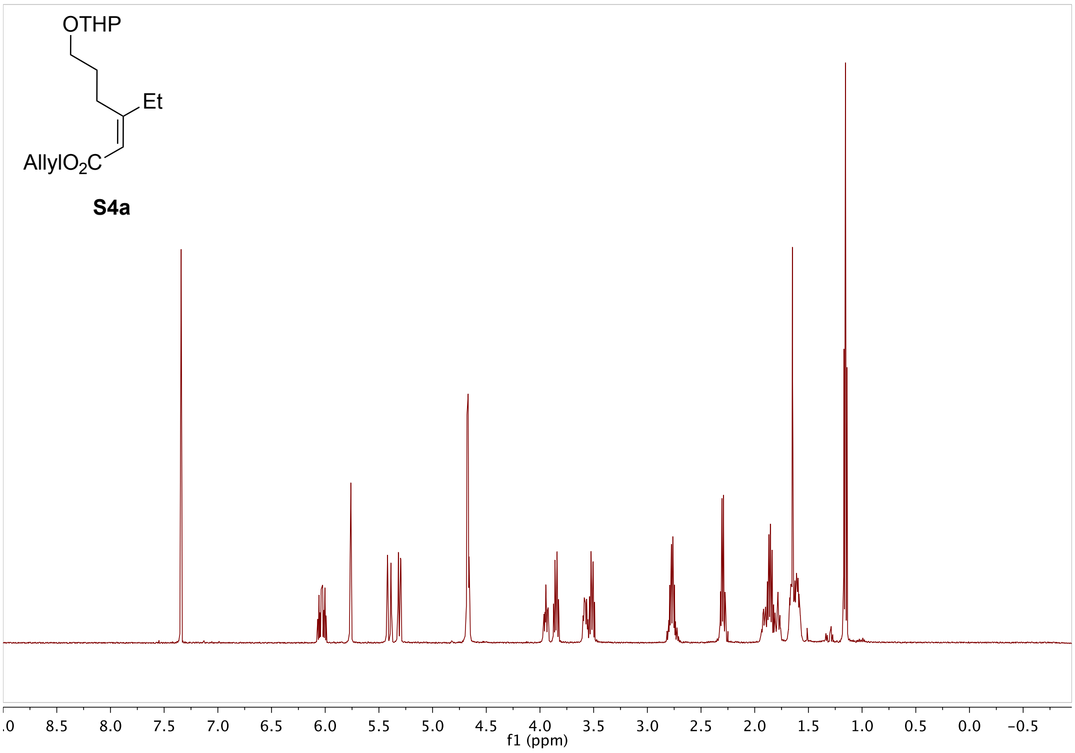
OTHP
Et
AllylO2C
S4a
f1 (ppm)

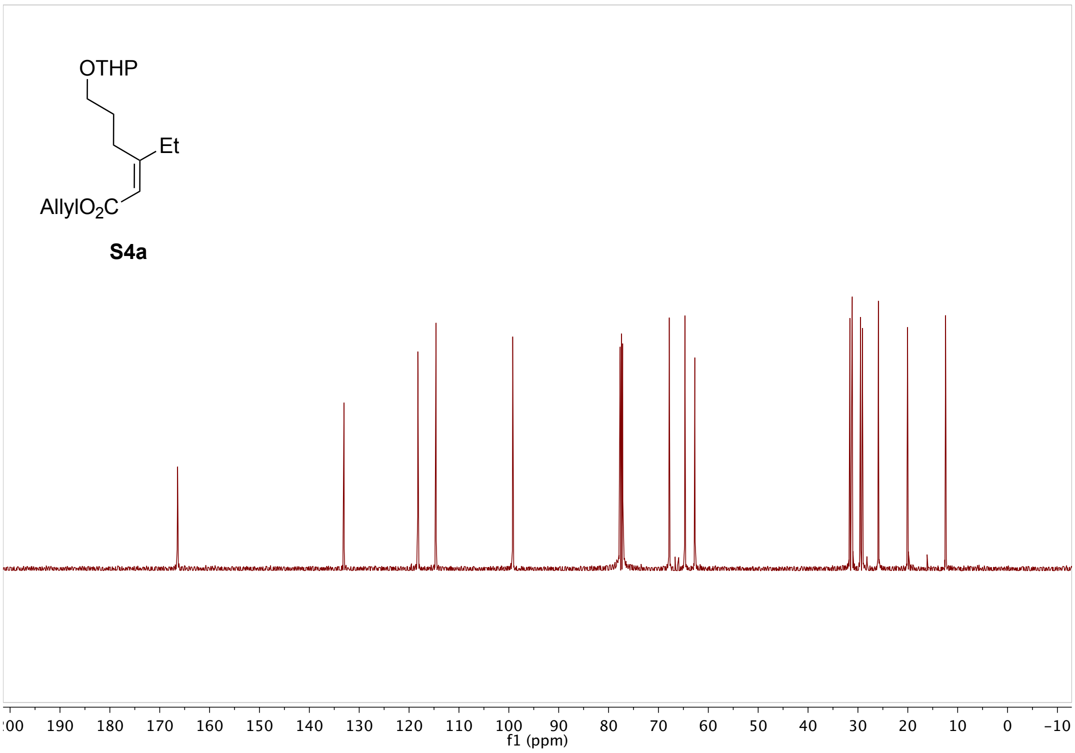
OTHP
Et
AllylO2C
S4a
f1 (ppm)

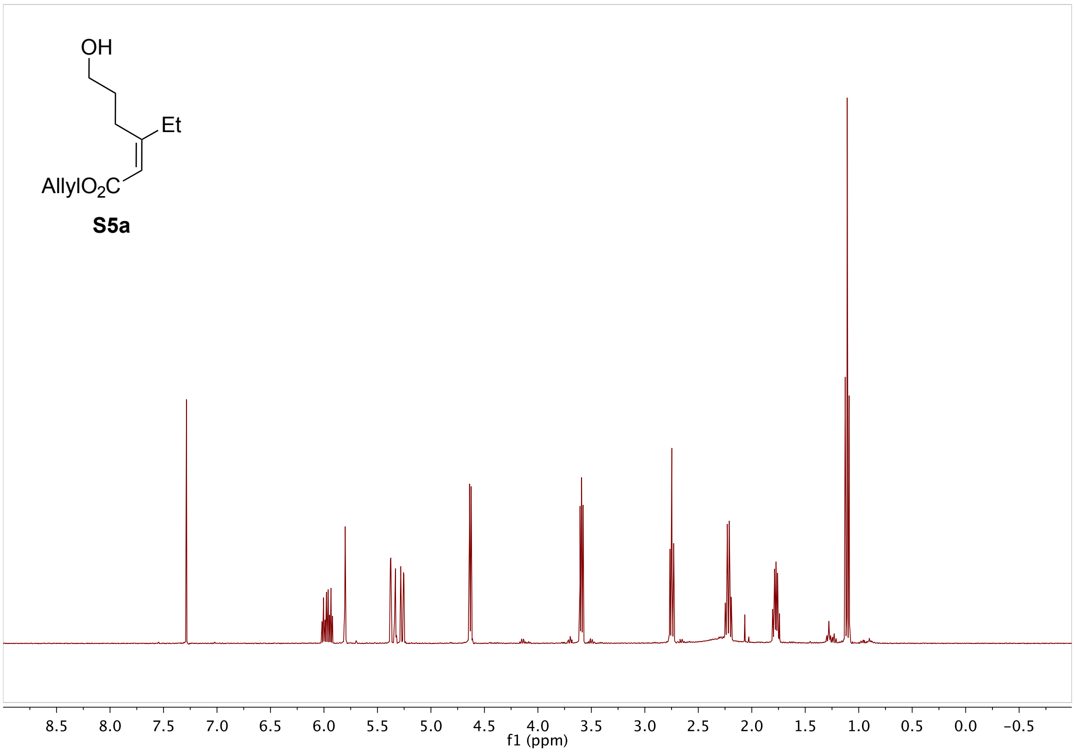
OH
Et
AllylO2C
S5a
8.5 8.0 7.5 7.0 6.5 6.0 5.5 5.0 4.5 4.0 3.5 3.0 2.5 2.0 1.5 1.0 0.5 0.0 −0.5
f1 (ppm)

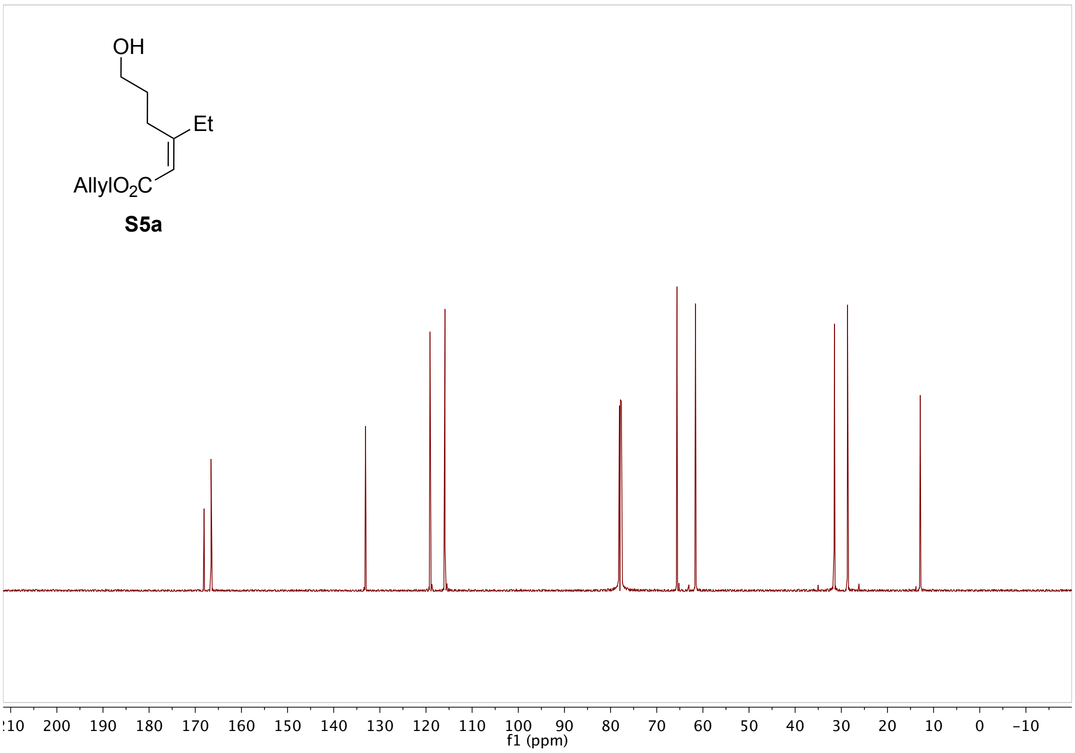
OH
Et
AllylO2C
S5a
200 190 180 170 160 150 140 130 120 110 100 90 80 70 60 50 40 30 20 10 0 −10
f1 (ppm)

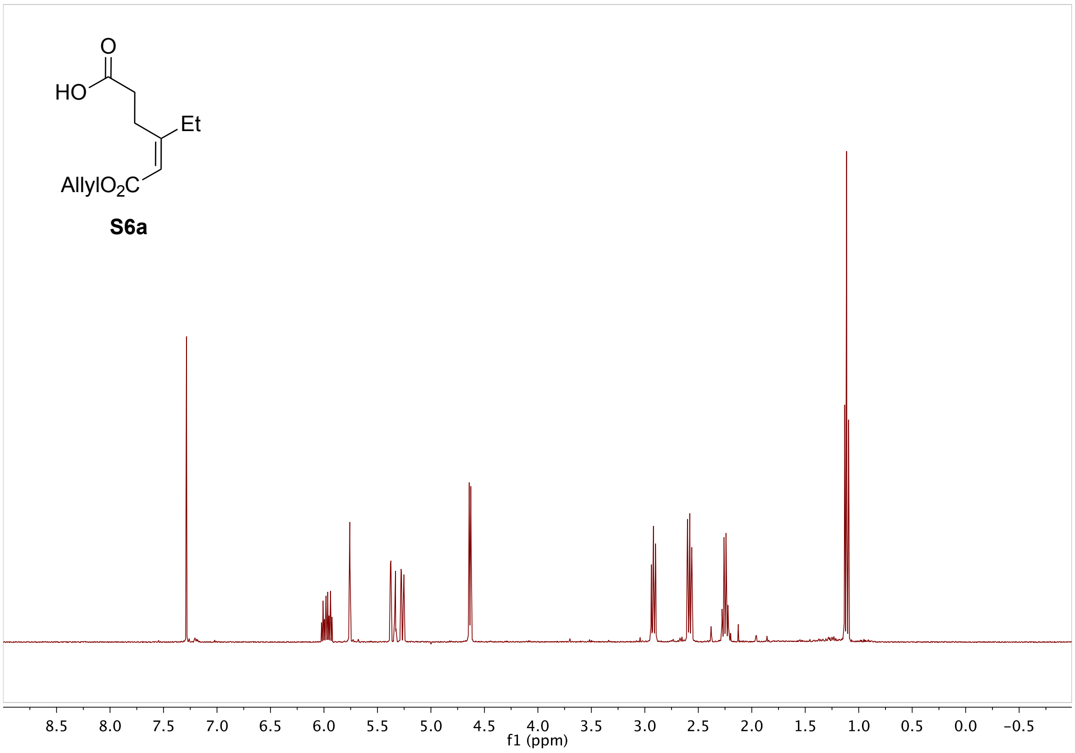
HO
O
Et
AllylO2C
S6a
8.5 8.0 7.5 7.0 6.5 6.0 5.5 5.0 4.5 4.0 3.5 3.0 2.5 2.0 1.5 1.0 0.5 0.0 −0.5
f1 (ppm)

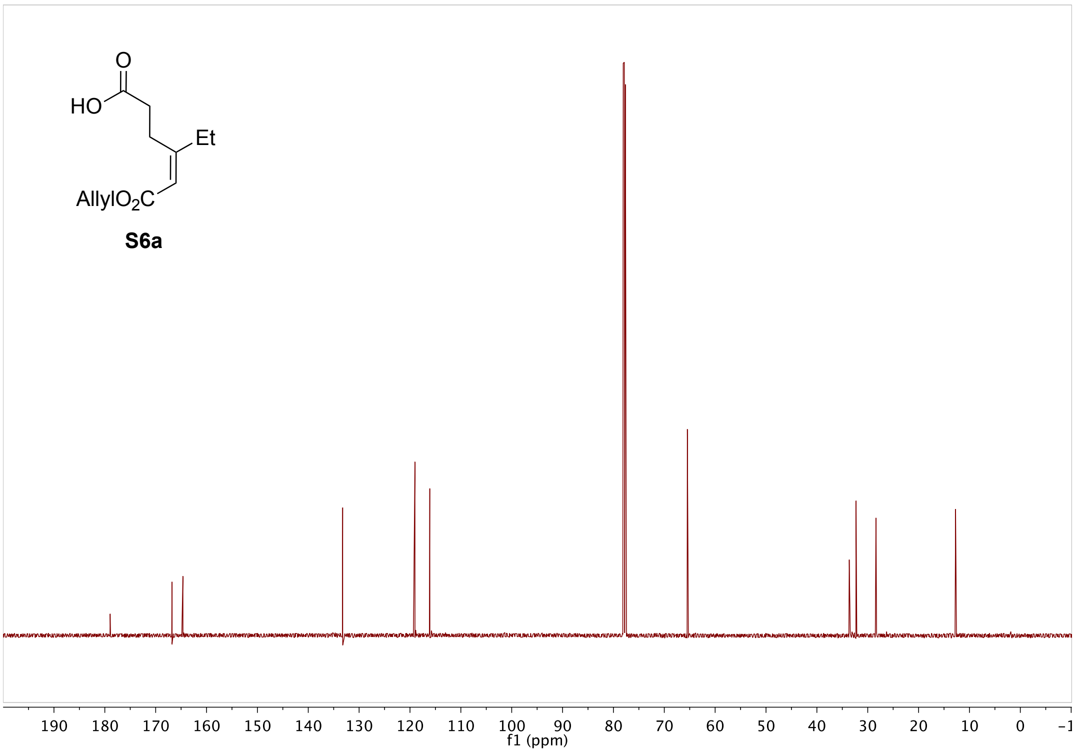
HO
O
Et
AllylO2C
S6a
190 180 170 160 150 140 130 120 110 100 90 80 70 60 50 40 30 20 10 0
f1 (ppm)

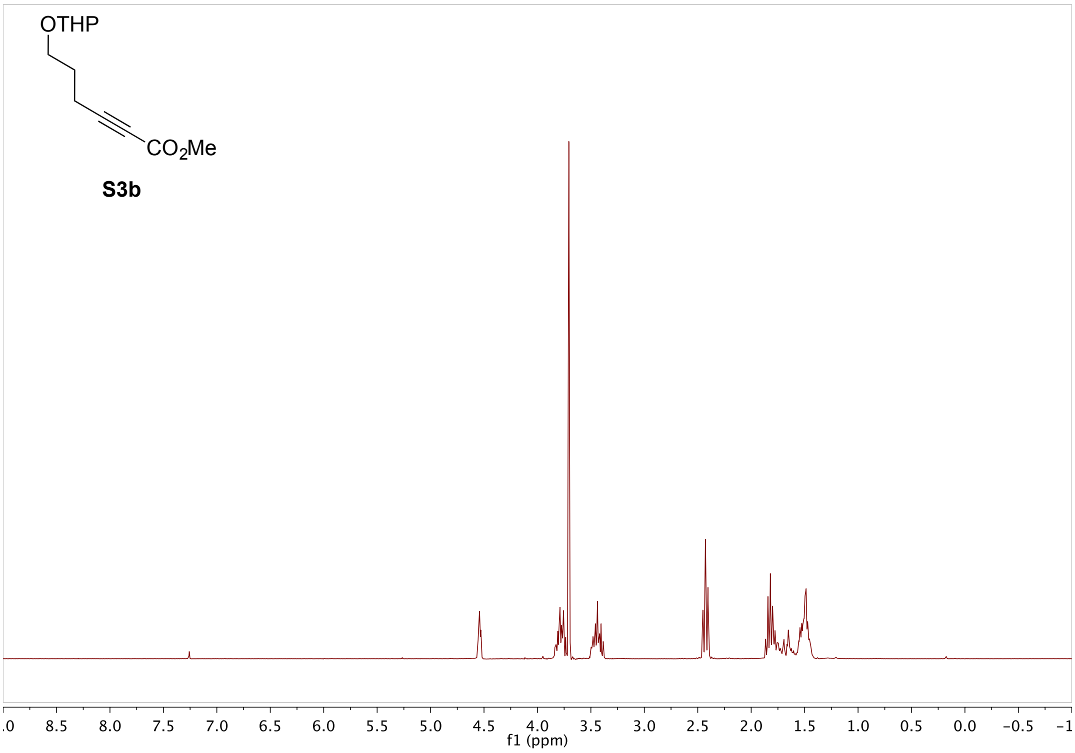
OTHP
CO_2Me
S3b
f1 (ppm)

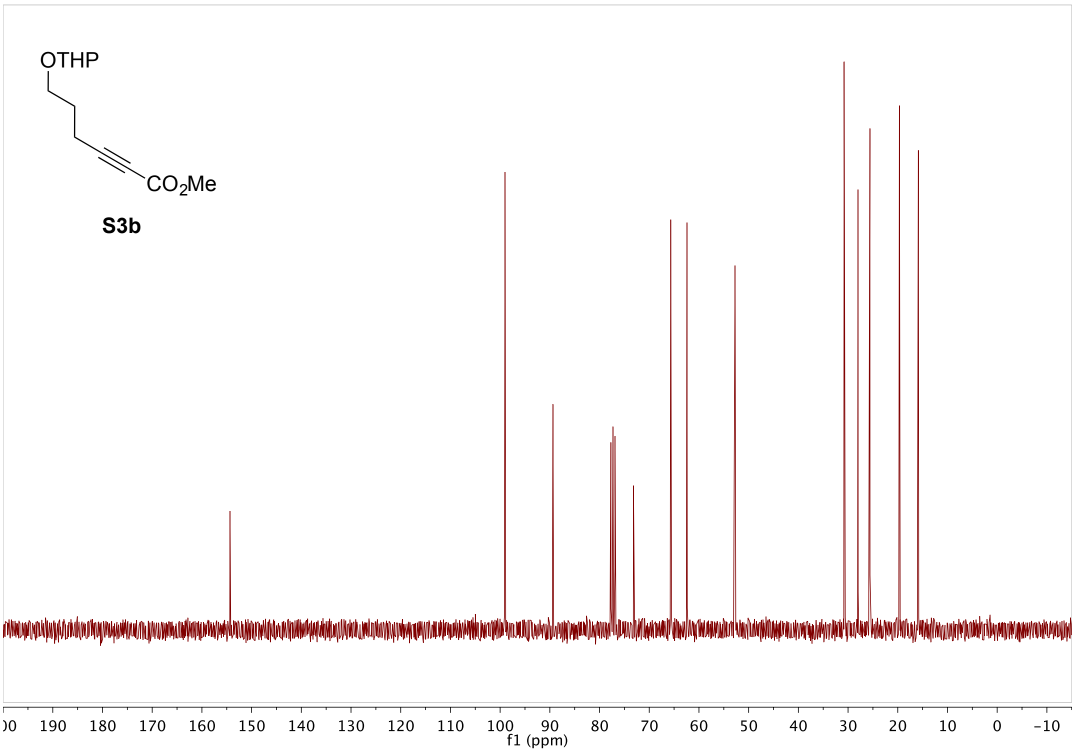
OTHP
CO_2Me
S3b
f1 (ppm)

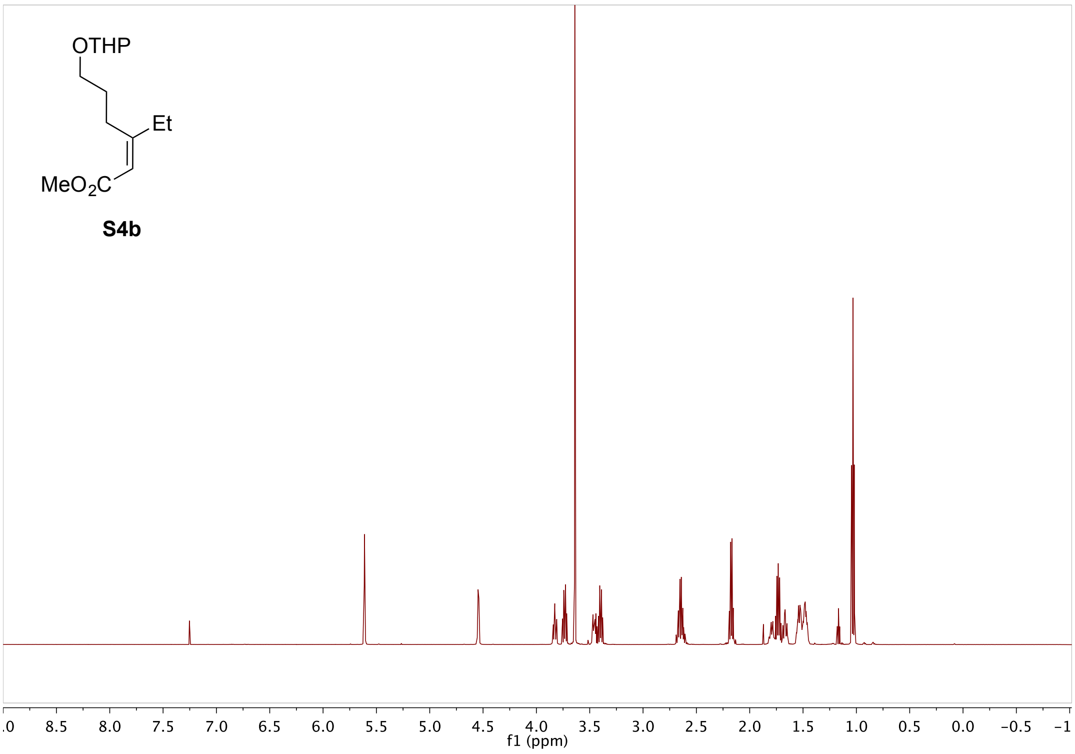
OTHP
Et
MeO_2C
S4b
f1 (ppm)

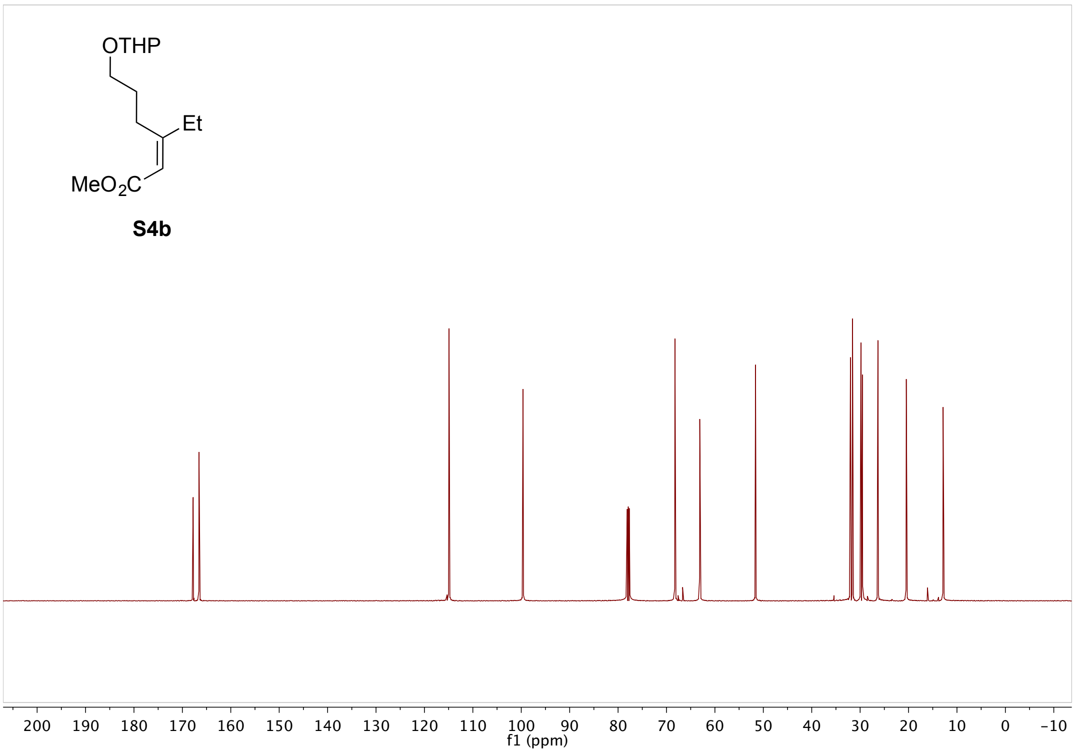
OTHP
Et
MeO_2C
S4b
f1 (ppm)

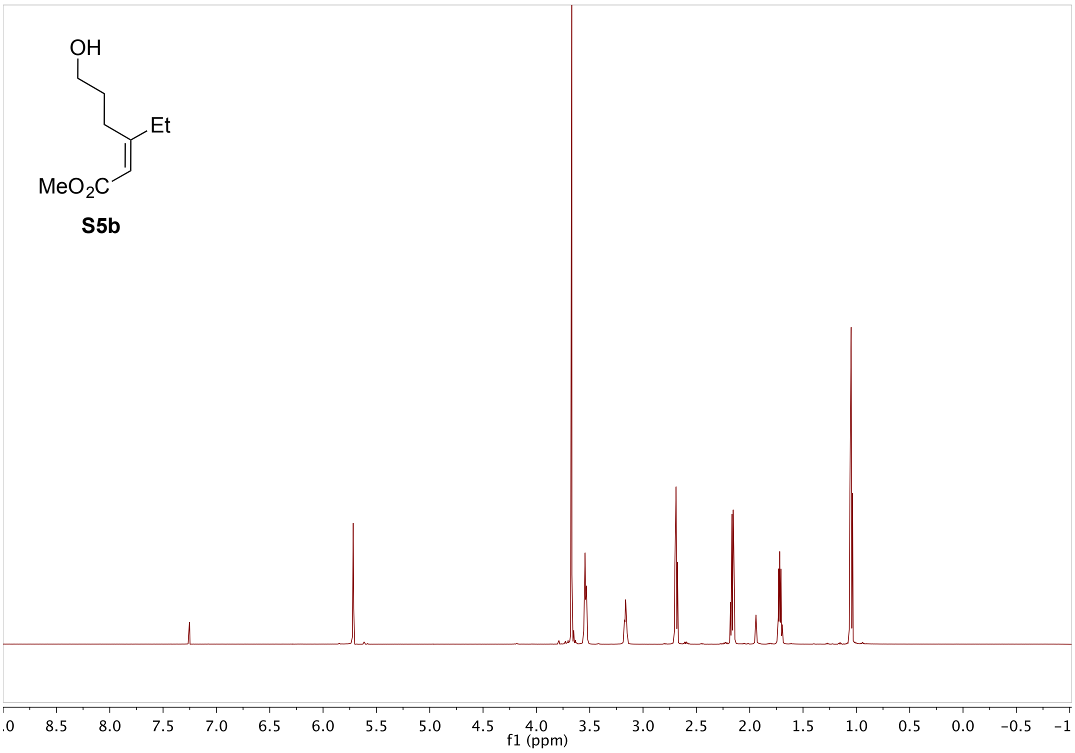
OH
Et
MeO2C
S5b
f1 (ppm)

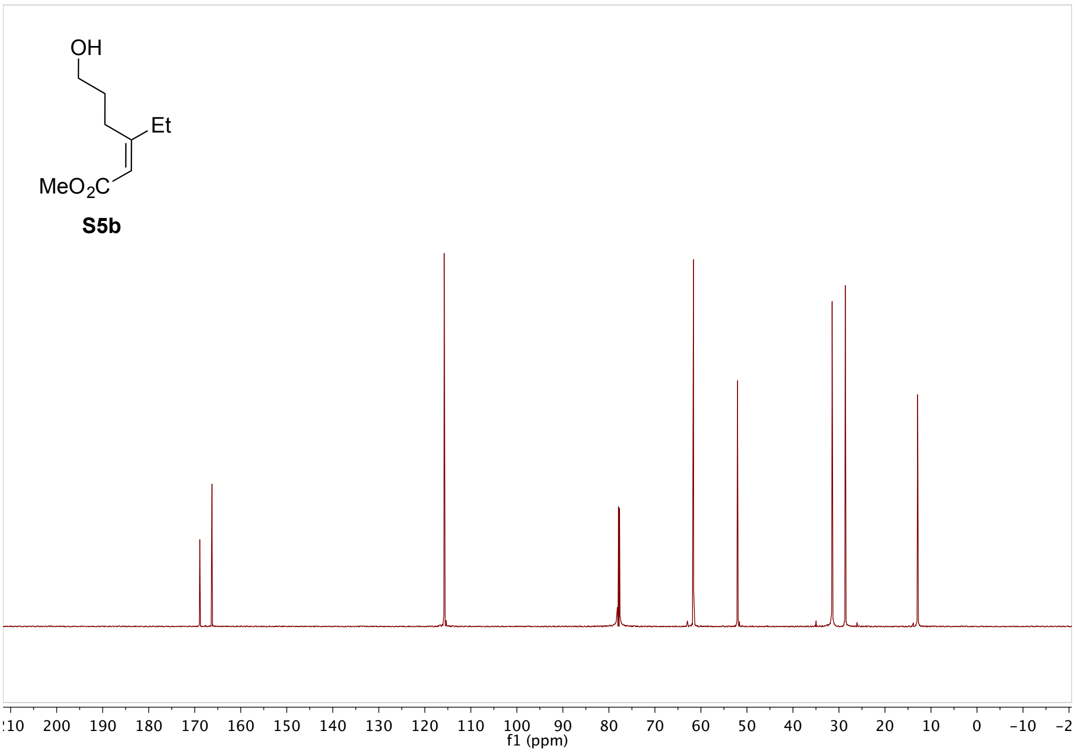
OH
Et
MeO2C
S5b
f1 (ppm)

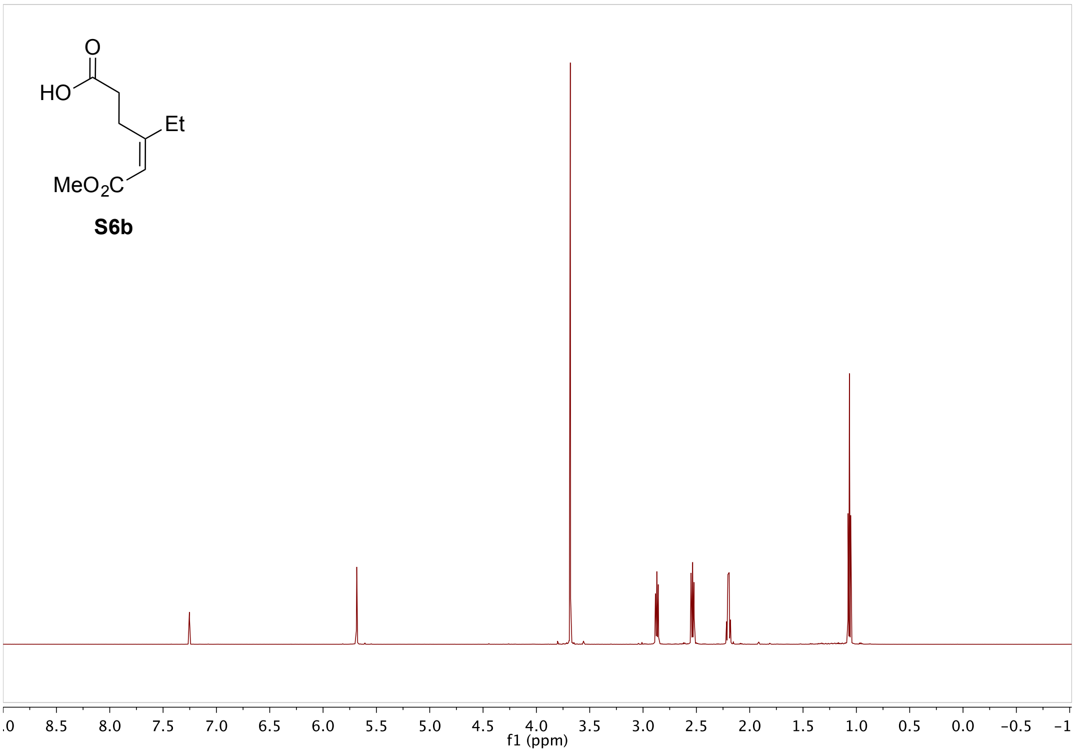
HO
O
Et
MeO2C
S6b
f1 (ppm)

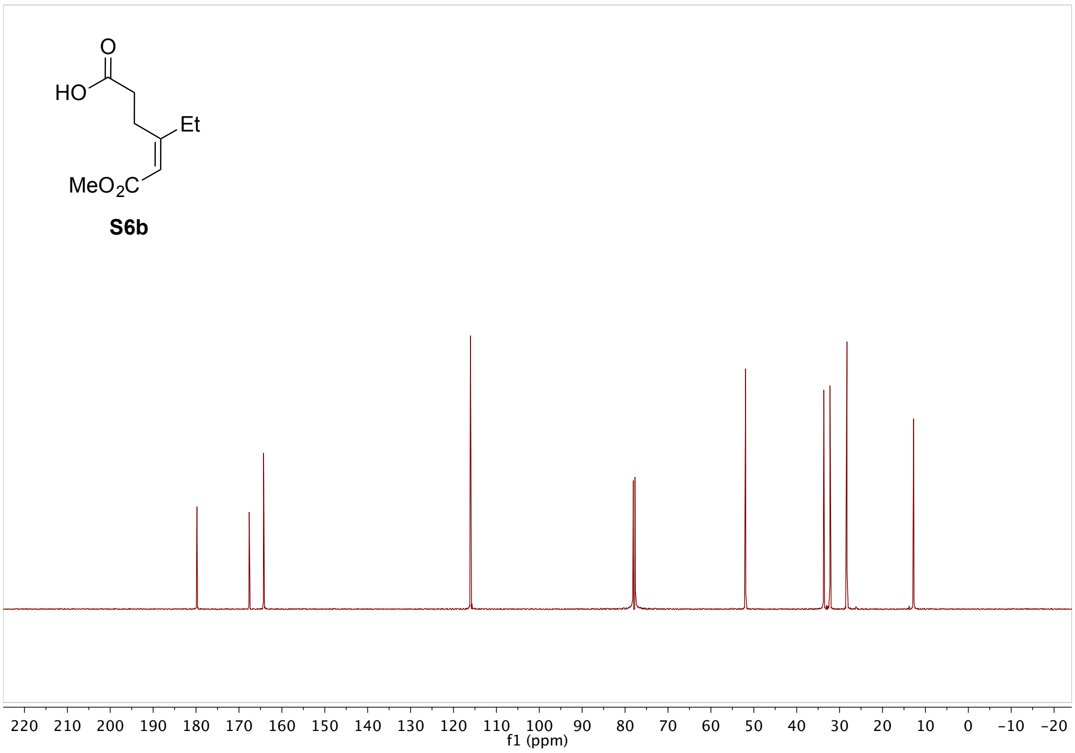
HO
O
Et
MeO2C
S6b
f1 (ppm)

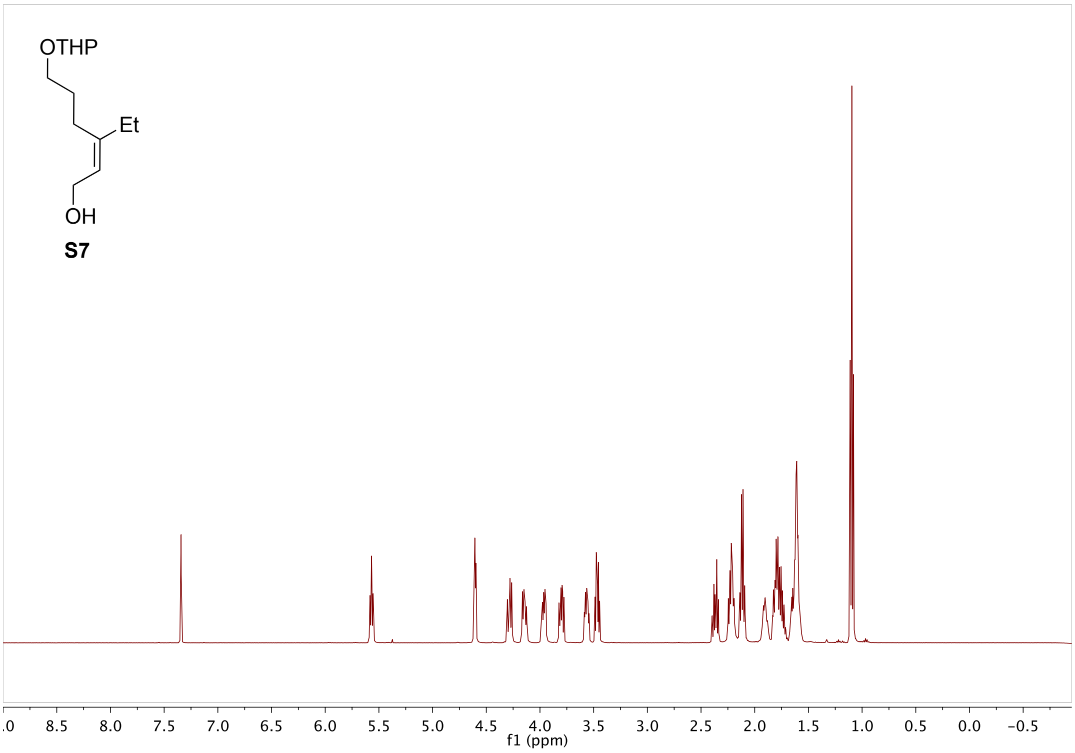
OTHP
Et
OH
S7
.0 8.5 8.0 7.5 7.0 6.5 6.0 5.5 5.0 4.5 4.0 3.5 3.0 2.5 2.0 1.5 1.0 0.5 0.0 -0.5
f1 (ppm)

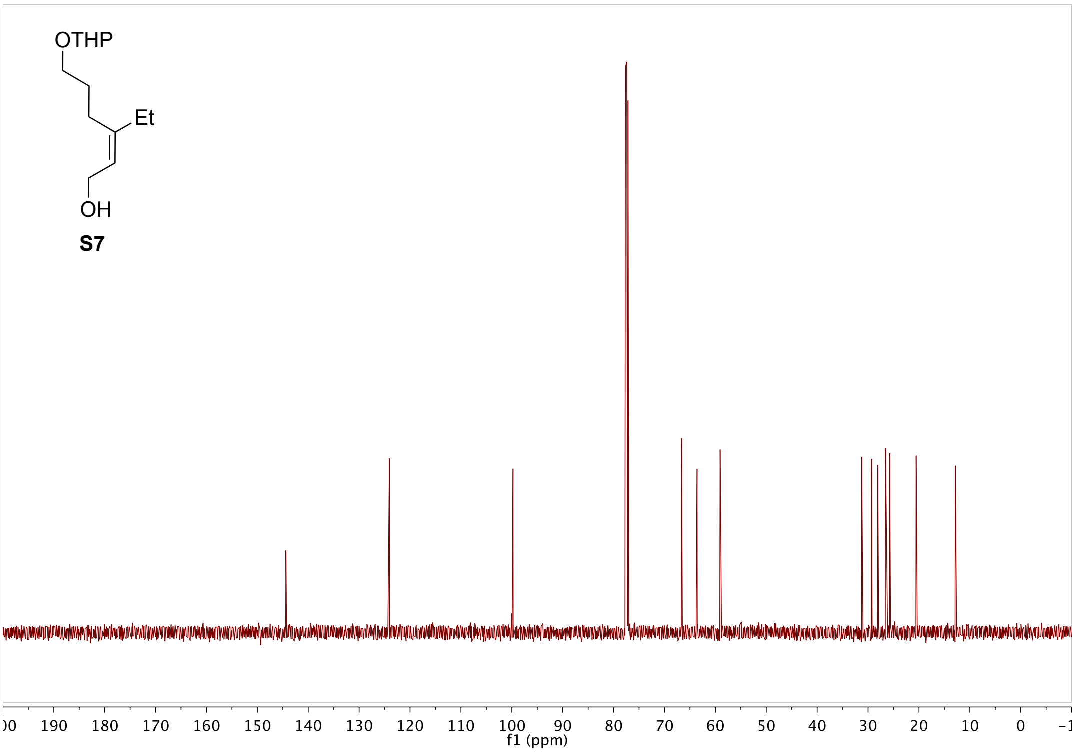
OTHP
Et
OH
S7
)0 190 180 170 160 150 140 130 120 110 100 90 80 70 60 50 40 30 20 10 0 -1
f1 (ppm)

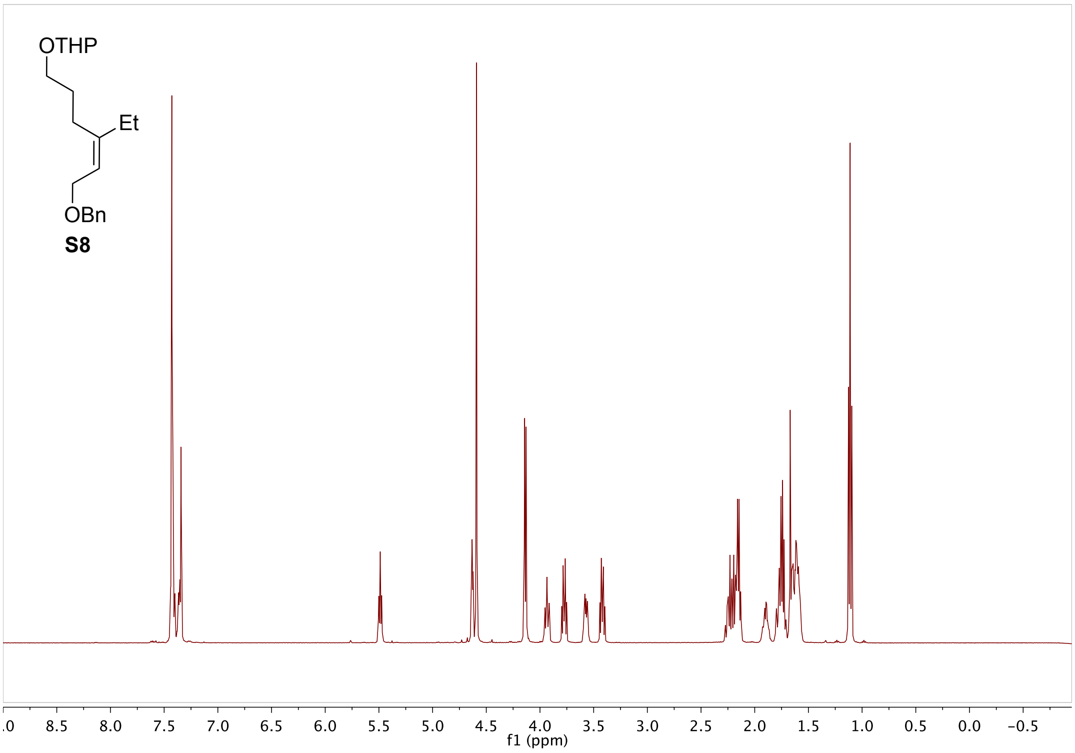
OTHP
Et
OBn
S8
9.0 8.5 8.0 7.5 7.0 6.5 6.0 5.5 5.0 4.5 4.0 3.5 3.0 2.5 2.0 1.5 1.0 0.5 0.0 −0.5
f1 (ppm)

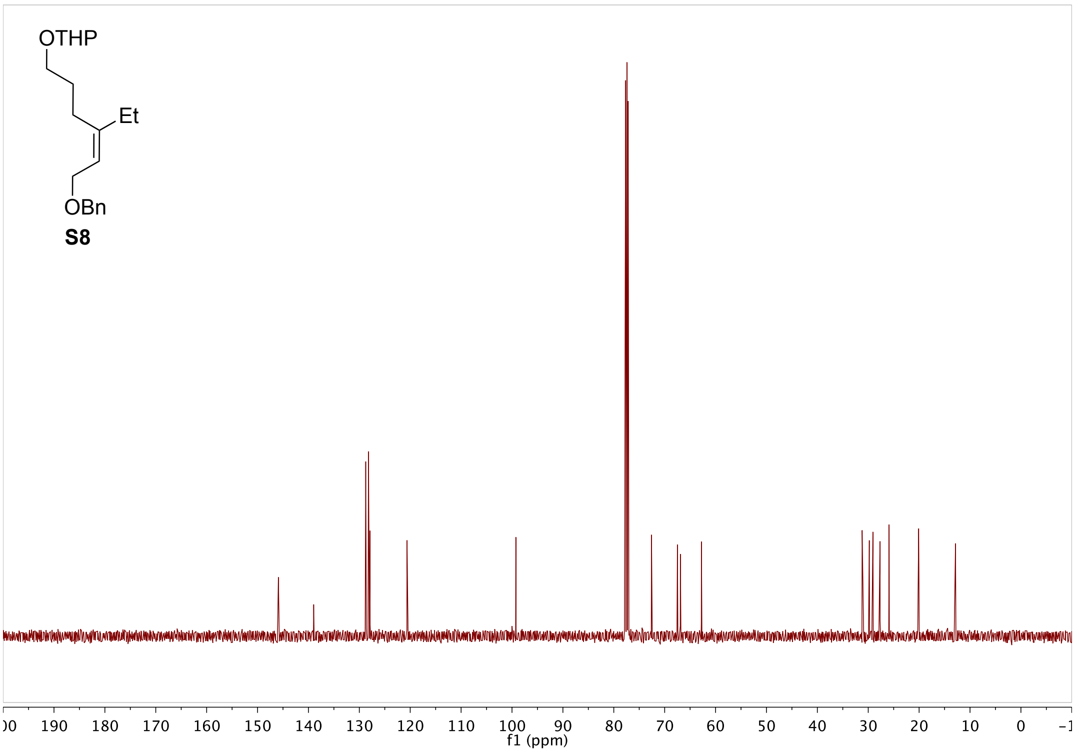
OTHP
Et
OBn
S8
190 180 170 160 150 140 130 120 110 100 90 80 70 60 50 40 30 20 10 0
f1 (ppm)

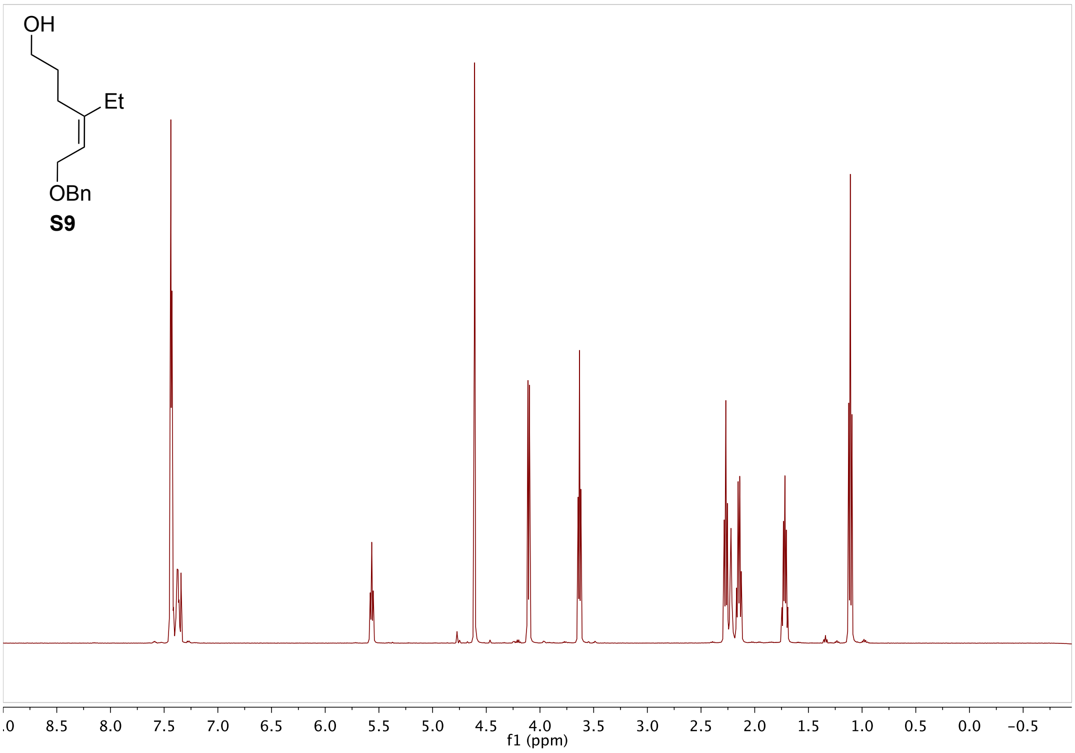
OH
Et
OBn
S9
.0
8.5
8.0
7.5
7.0
6.5
6.0
5.5
5.0
4.5
4.0
3.5
3.0
2.5
2.0
1.5
1.0
0.5
0.0
−0.5
f1 (ppm)

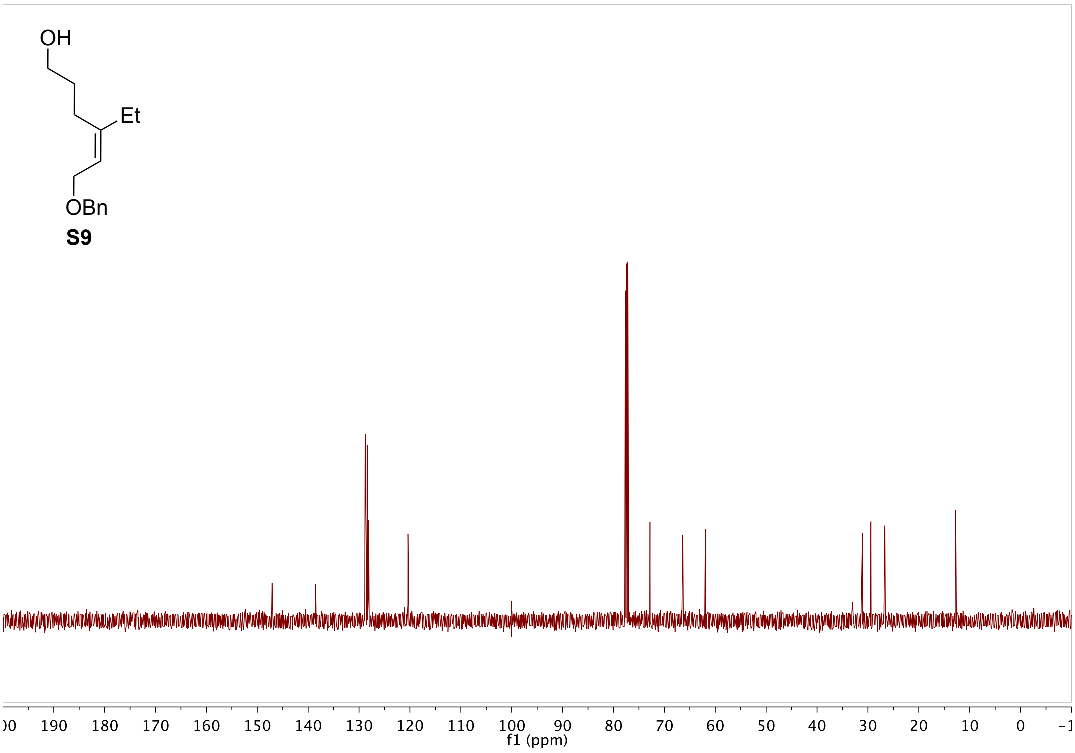
OH
Et
OBn
S9
190
180
170
160
150
140
130
120
110
100
90
80
70
60
50
40
30
20
10
0
f1 (ppm)

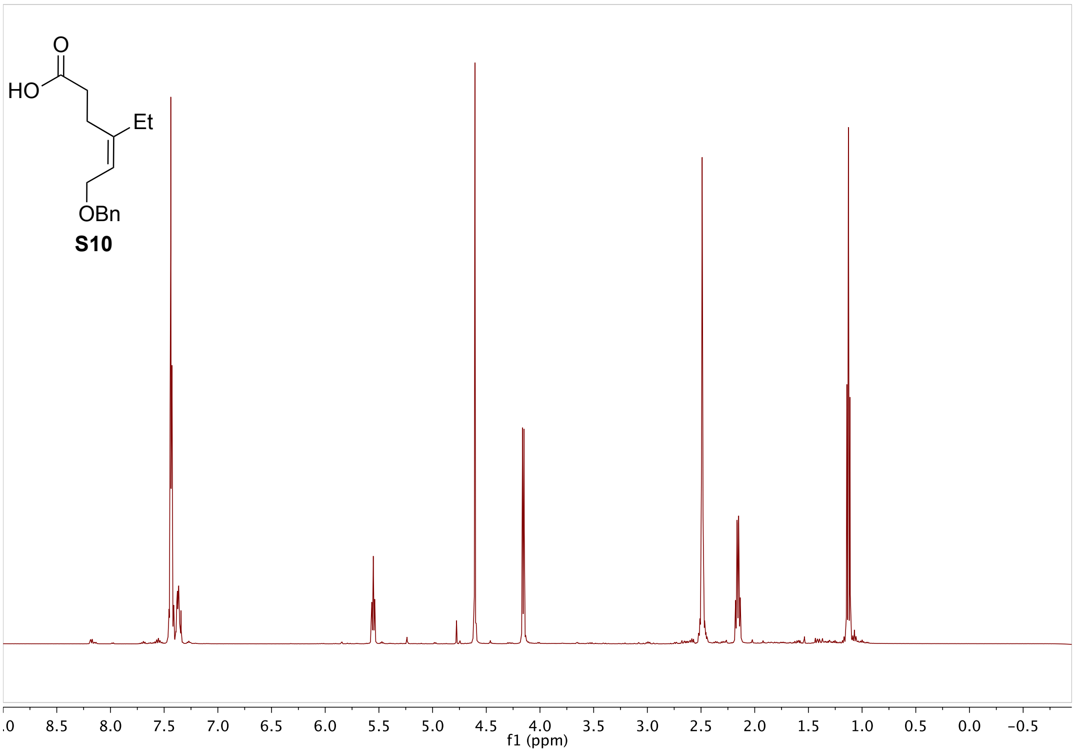
HO
O
Et
OBn
S10
8.5
8.0
7.5
7.0
6.5
6.0
5.5
5.0
4.5
4.0
3.5
3.0
2.5
2.0
1.5
1.0
0.5
0.0
−0.5
f1 (ppm)

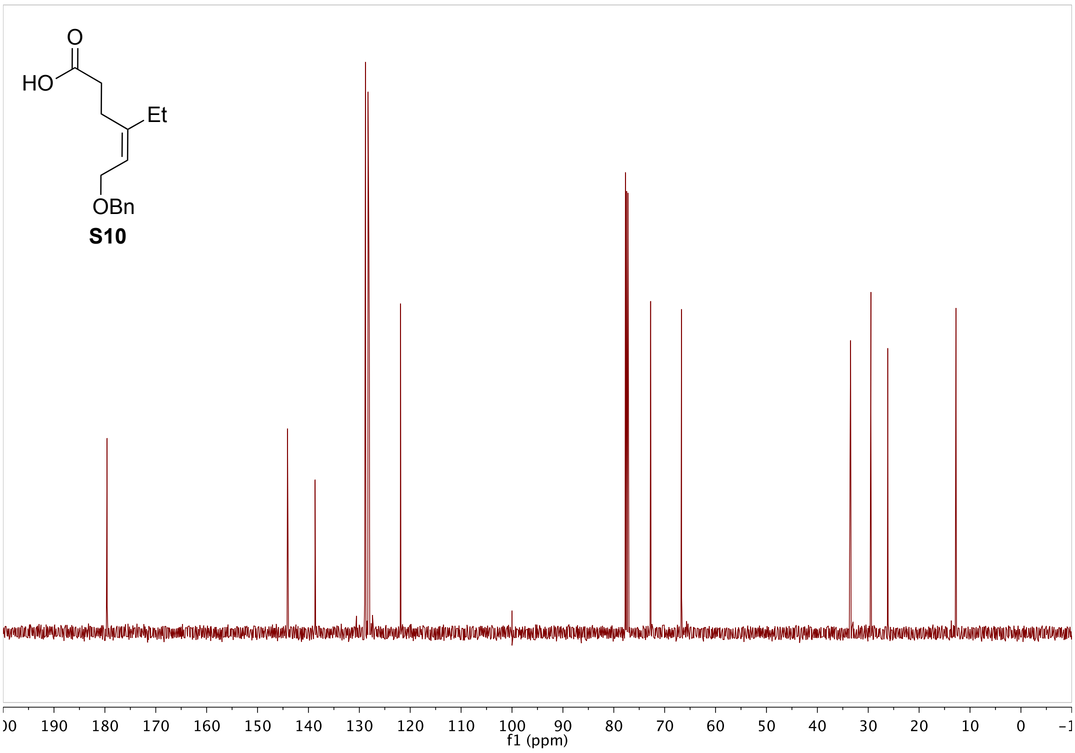
HO
O
Et
OBn
S10
190
180
170
160
150
140
130
120
110
100
90
80
70
60
50
40
30
20
10
0
f1 (ppm)

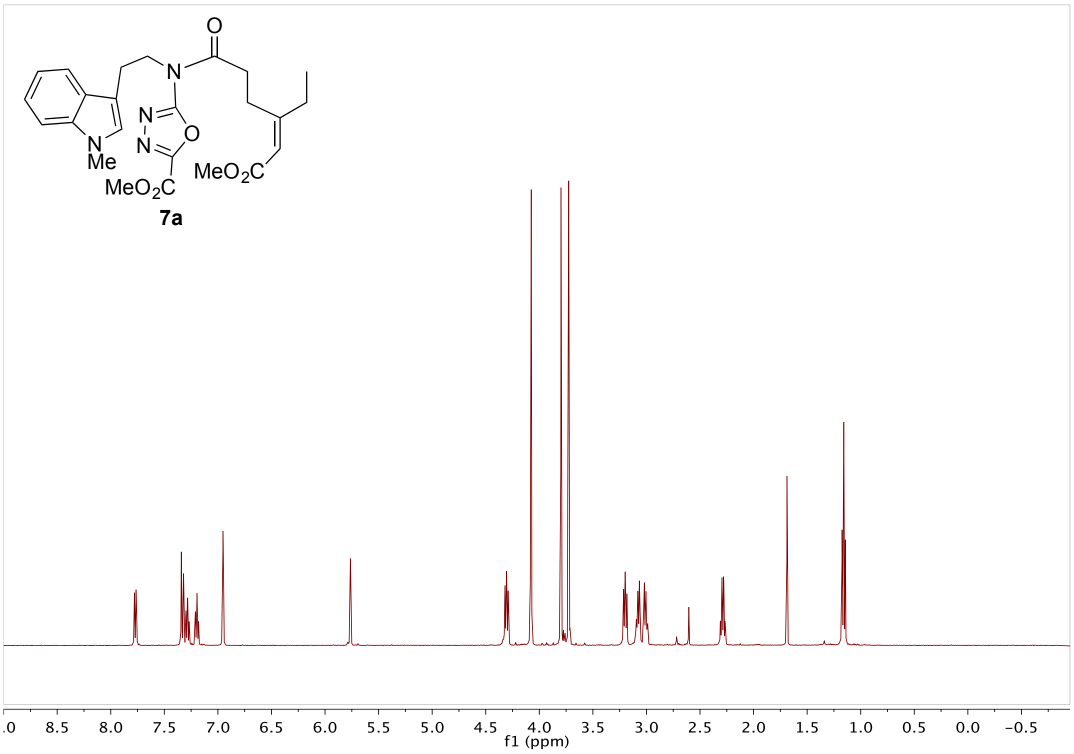
O
N
N
N
O
N
Me
MeO2C
MeO2C
7a
f1 (ppm)

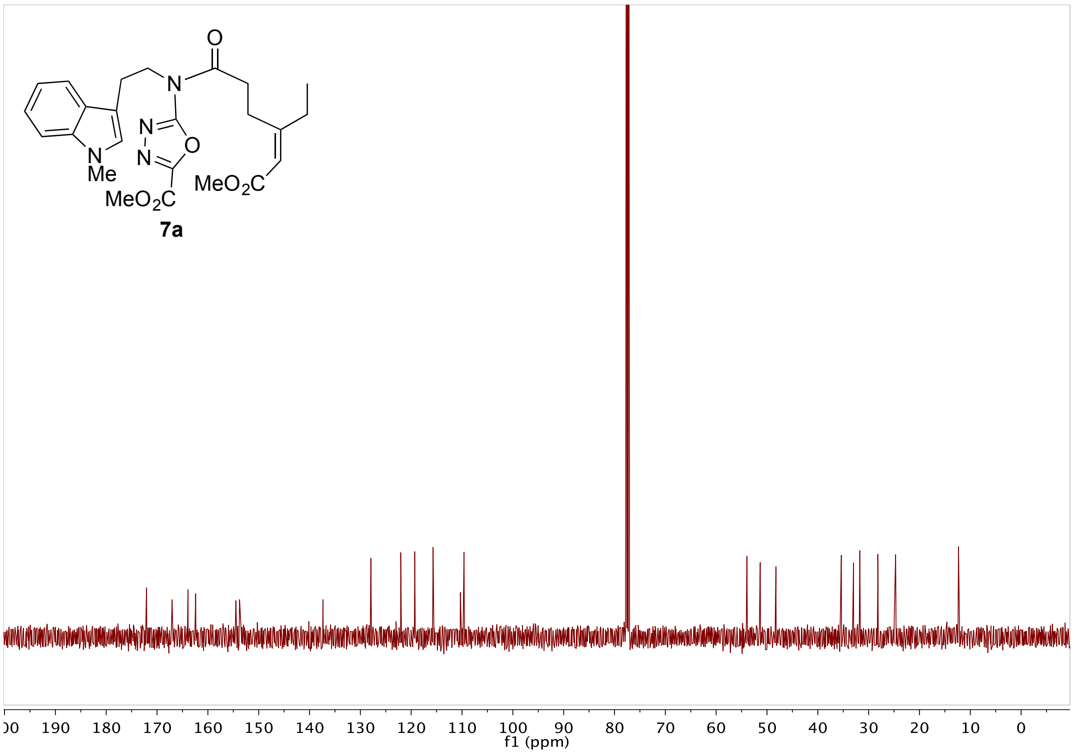
O
N
N
N
O
N
Me
MeO2C
MeO2C
7a
f1 (ppm)

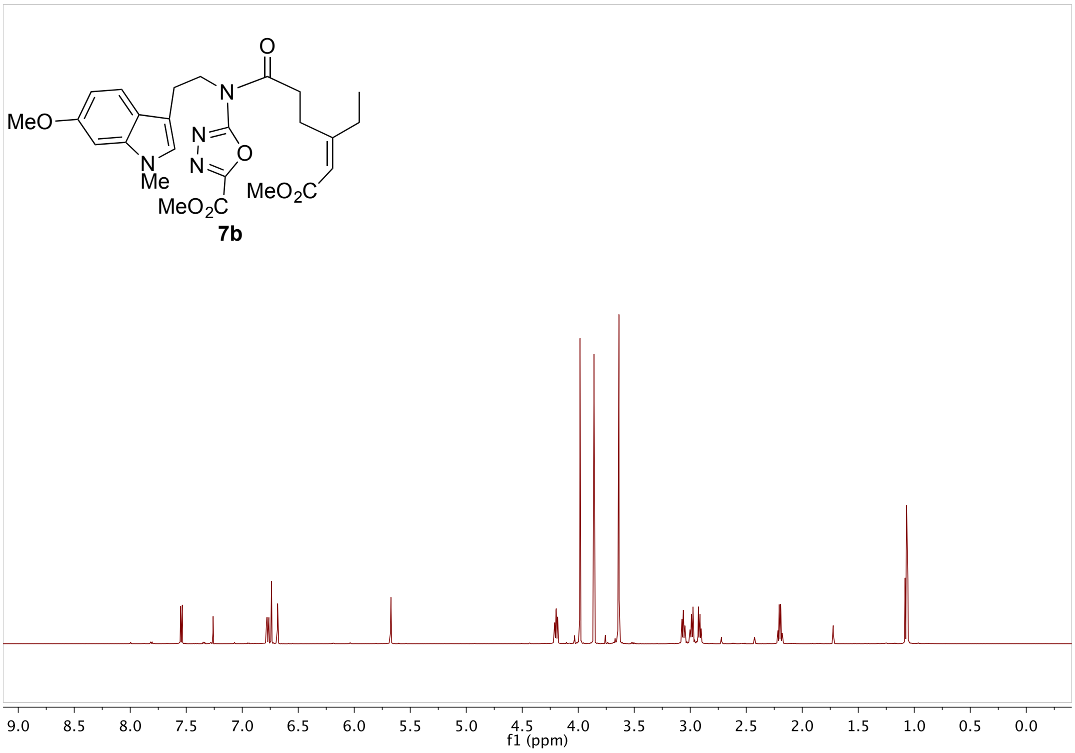
MeO
N
Me
N
N
N
O
O
MeO2C
MeO2C
7b
9.0
8.5
8.0
7.5
7.0
6.5
6.0
5.5
5.0
4.5
4.0
3.5
3.0
2.5
2.0
1.5
1.0
0.5
0.0
f1 (ppm)

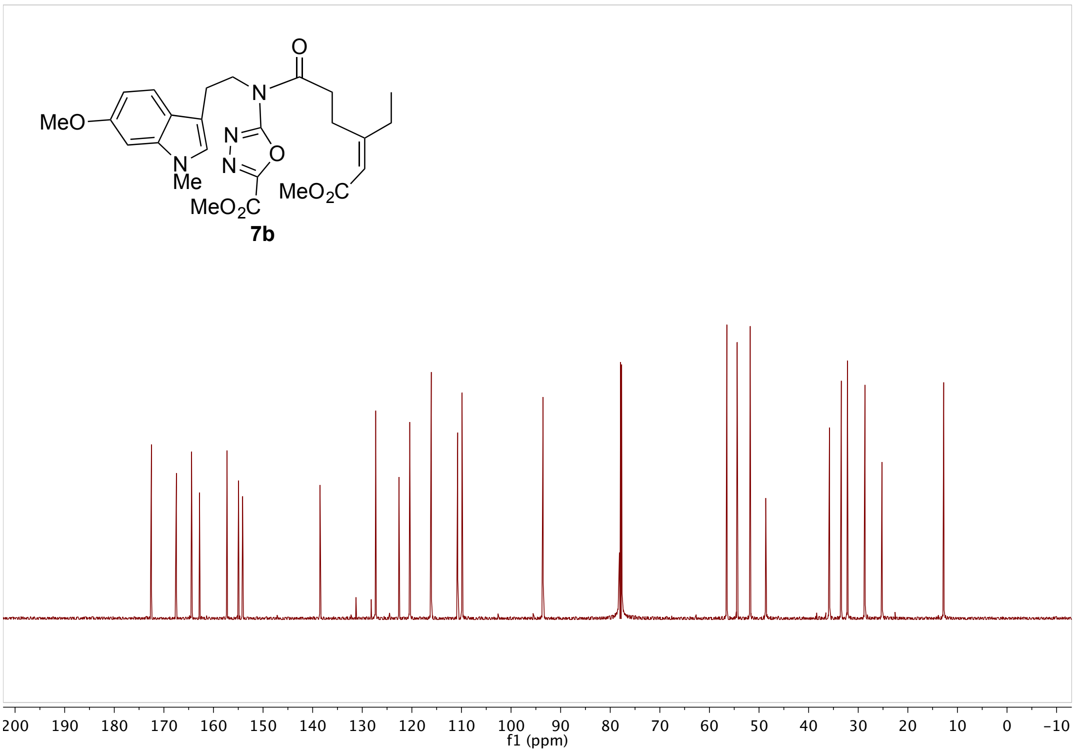
MeO
N
Me
N
N
N
O
O
MeO2C
MeO2C
7b
200
190
180
170
160
150
140
130
120
110
100
90
80
70
60
50
40
30
20
10
0
−10
f1 (ppm)

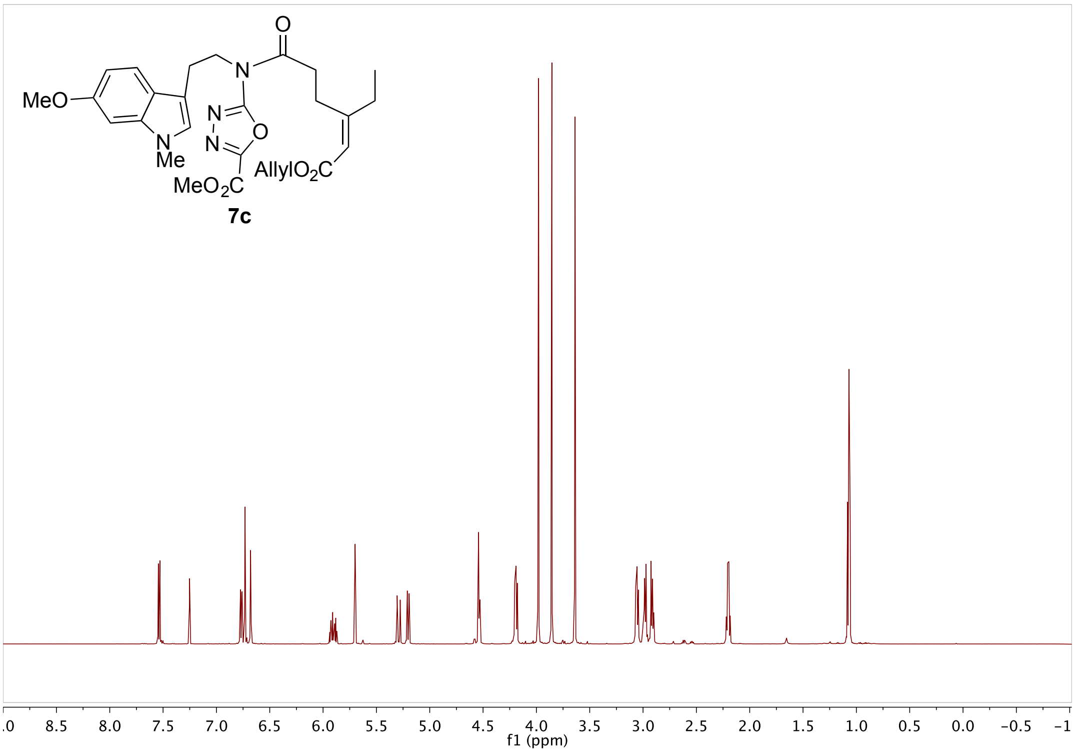
MeO
N
Me
N
N
O
O
MeO2C
AllylO2C
7c
.0 8.5 8.0 7.5 7.0 6.5 6.0 5.5 5.0 4.5 4.0 3.5 3.0 2.5 2.0 1.5 1.0 0.5 0.0 −0.5 −1
f1 (ppm)

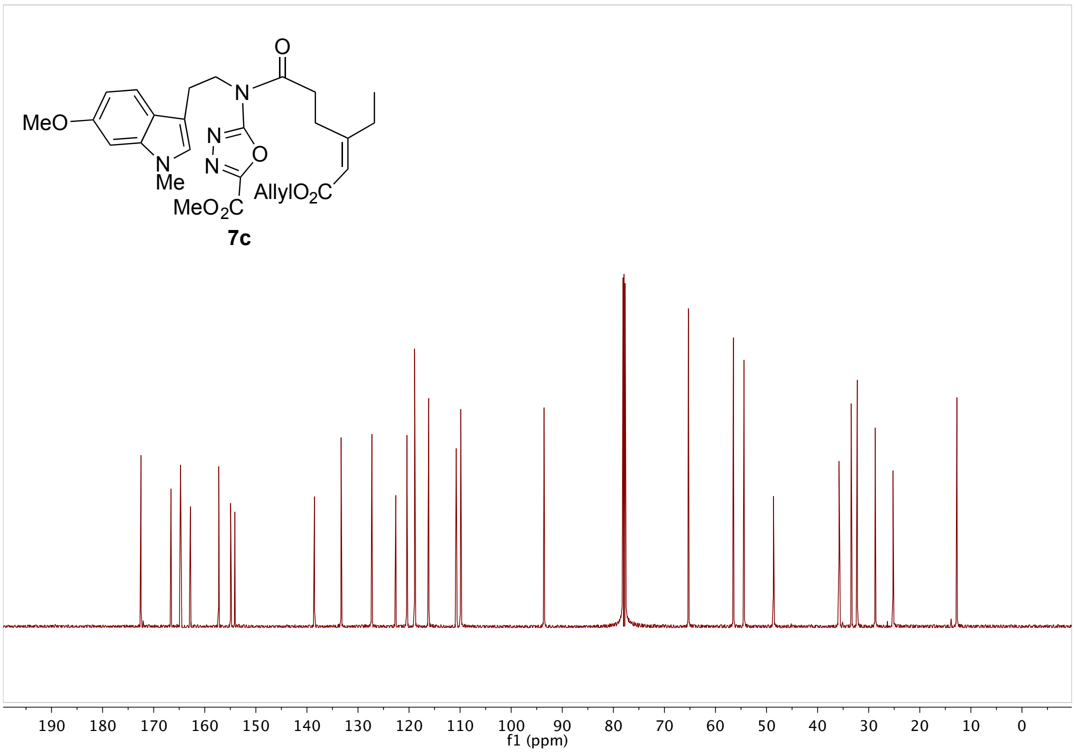
MeO
N
Me
N
N
O
O
MeO2C
AllylO2C
7c
190 180 170 160 150 140 130 120 110 100 90 80 70 60 50 40 30 20 10 0
f1 (ppm)

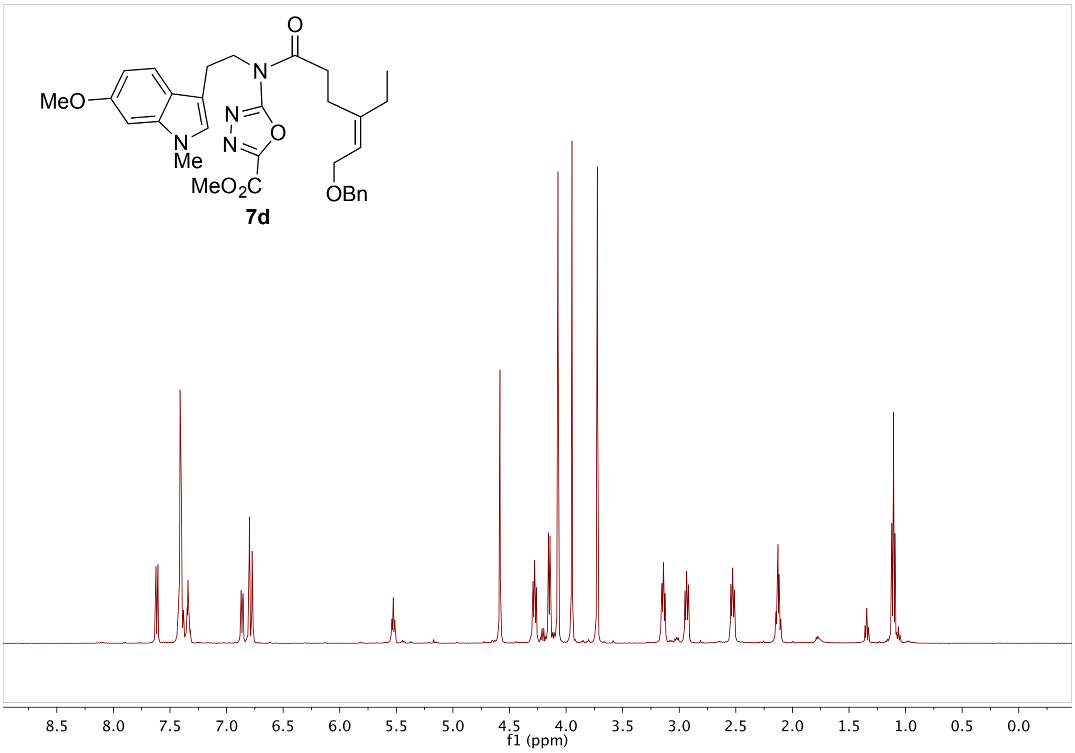
MeO
N
Me
N
N
O
MeO2C
N
O
OBn
7d
8.5
8.0
7.5
7.0
6.5
6.0
5.5
5.0
4.5
4.0
3.5
3.0
2.5
2.0
1.5
1.0
0.5
0.0
f1 (ppm)

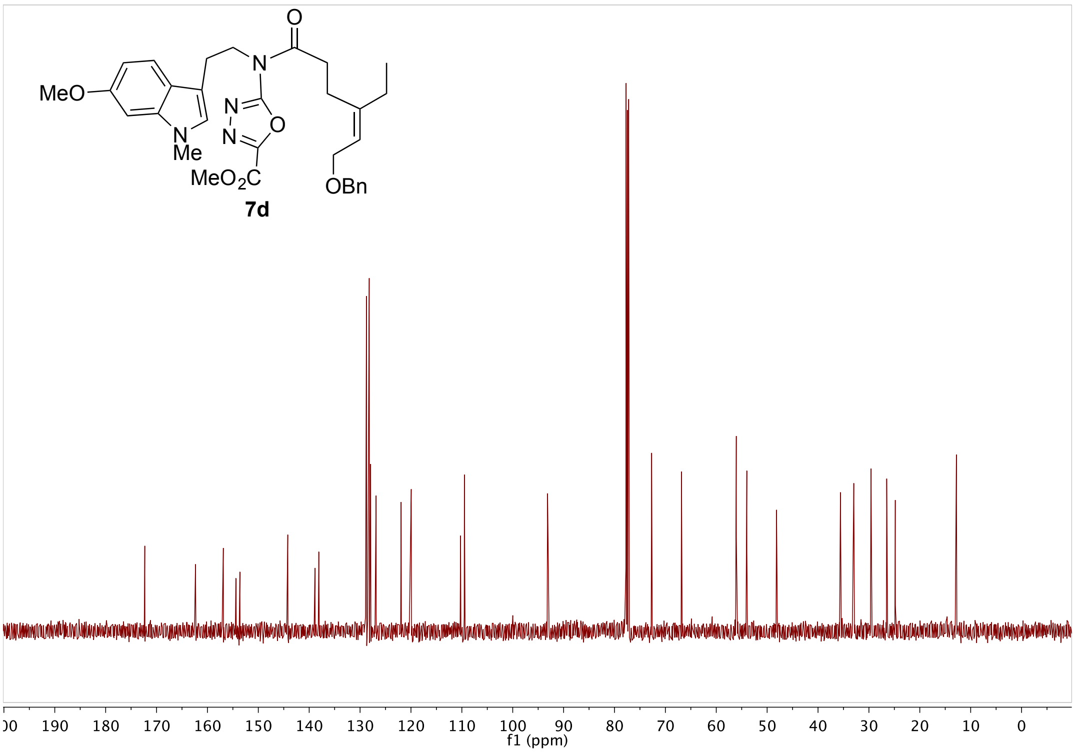
MeO
N
Me
N
N
O
MeO2C
N
O
OBn
7d
190
180
170
160
150
140
130
120
110
100
90
80
70
60
50
40
30
20
10
0
f1 (ppm)

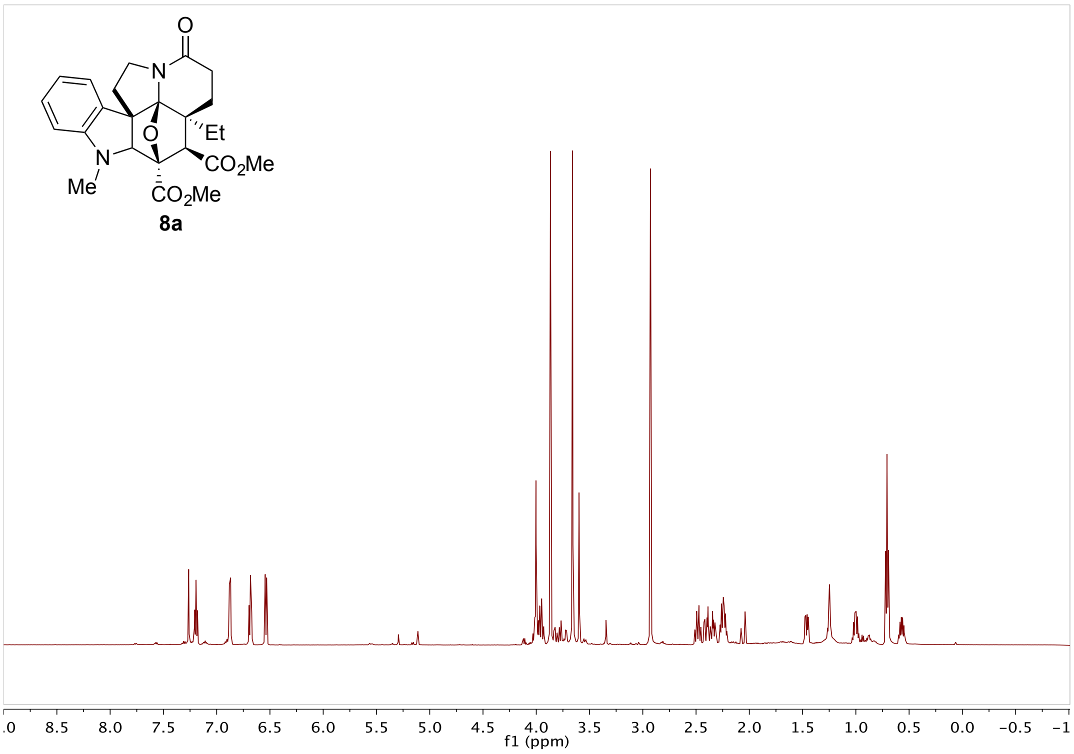

8a
f1 (ppm)

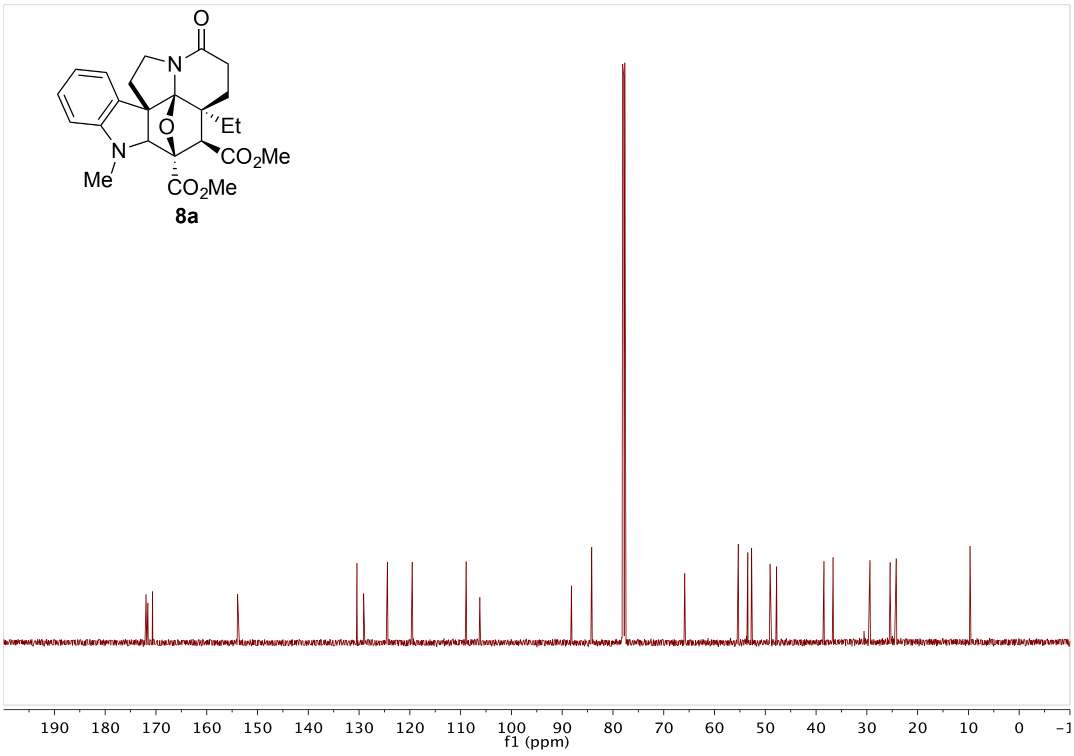

8a
f1 (ppm)

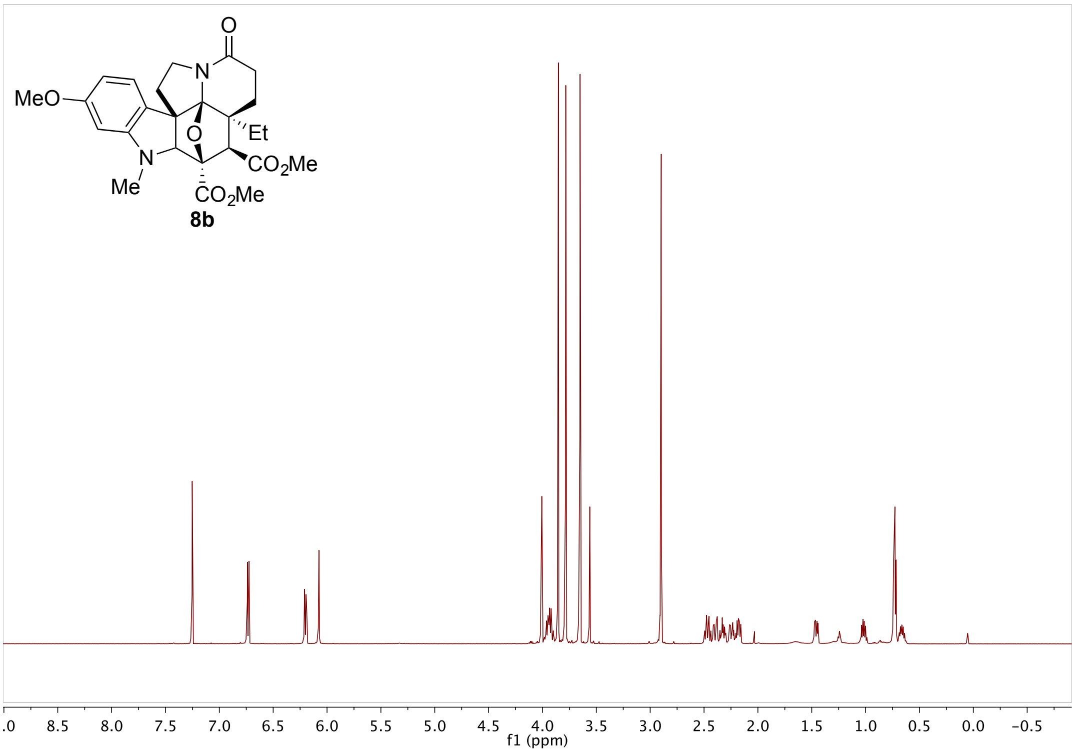
MeO
N
O
Et
CO2Me
N
Me
CO2Me
8b
.0
8.5
8.0
7.5
7.0
6.5
6.0
5.5
5.0
4.5
4.0
3.5
3.0
2.5
2.0
1.5
1.0
0.5
0.0
-0.5
f1 (ppm)

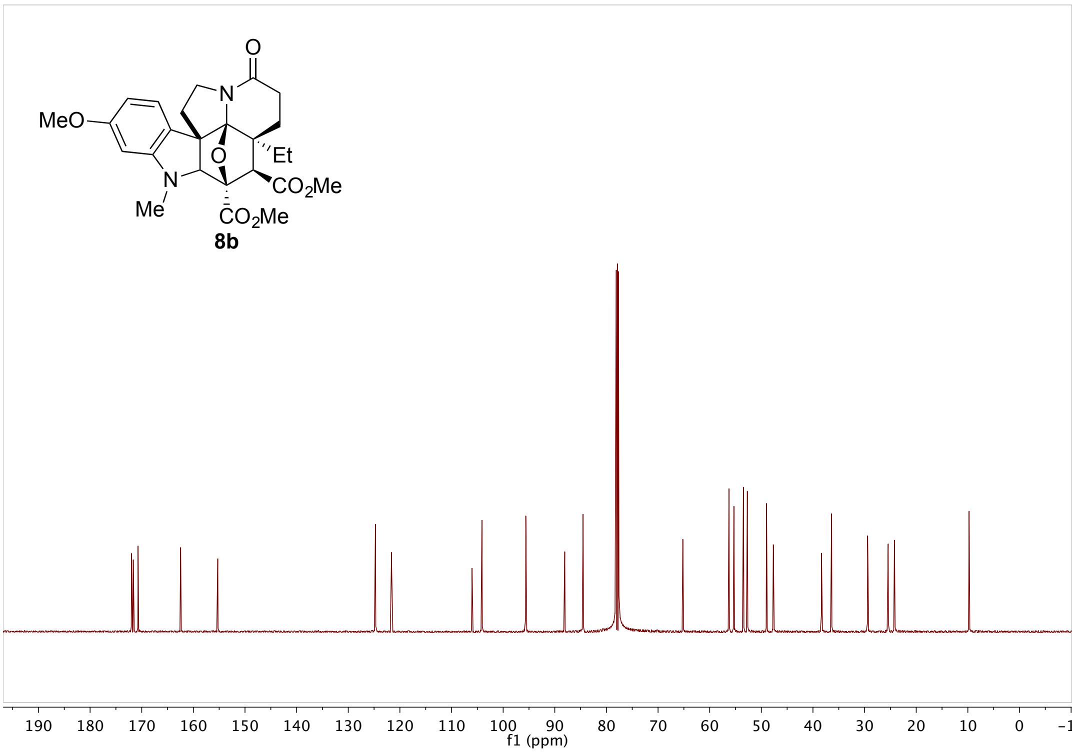
MeO
N
O
Et
CO2Me
N
Me
CO2Me
8b
190
180
170
160
150
140
130
120
110
100
90
80
70
60
50
40
30
20
10
0
-1
f1 (ppm)

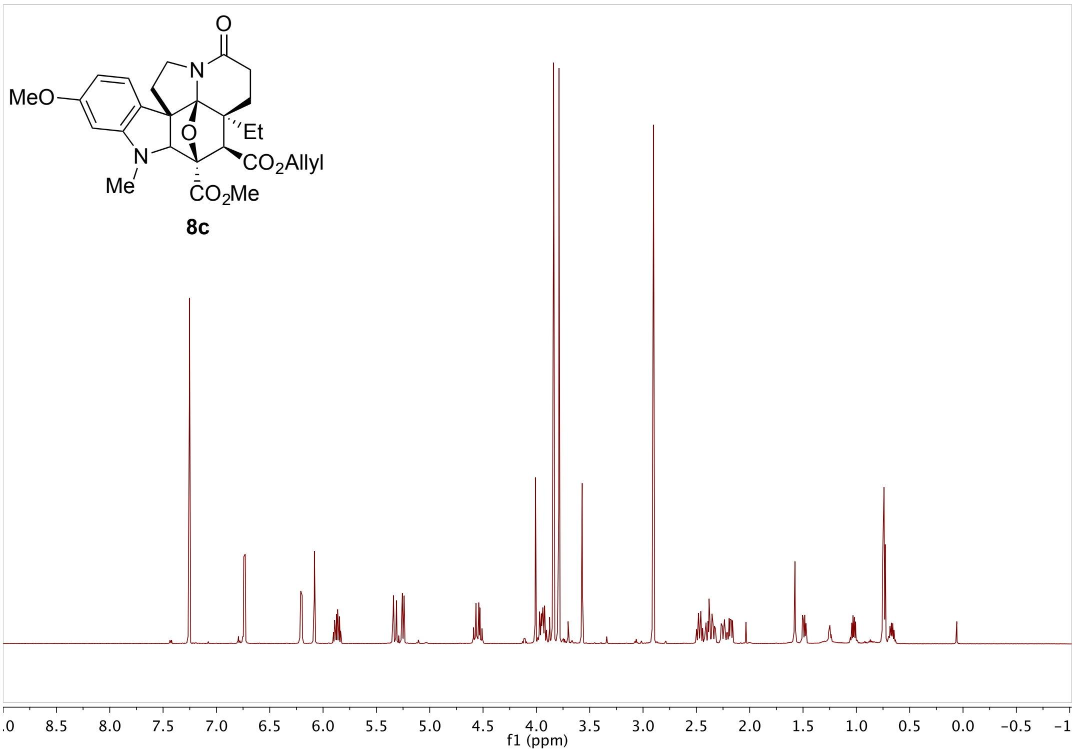
MeO
N
Me
O
N
O
Et
CO2Allyl
CO2Me
8c
.0 8.5 8.0 7.5 7.0 6.5 6.0 5.5 5.0 4.5 4.0 3.5 3.0 2.5 2.0 1.5 1.0 0.5 0.0 -0.5 -1
f1 (ppm)

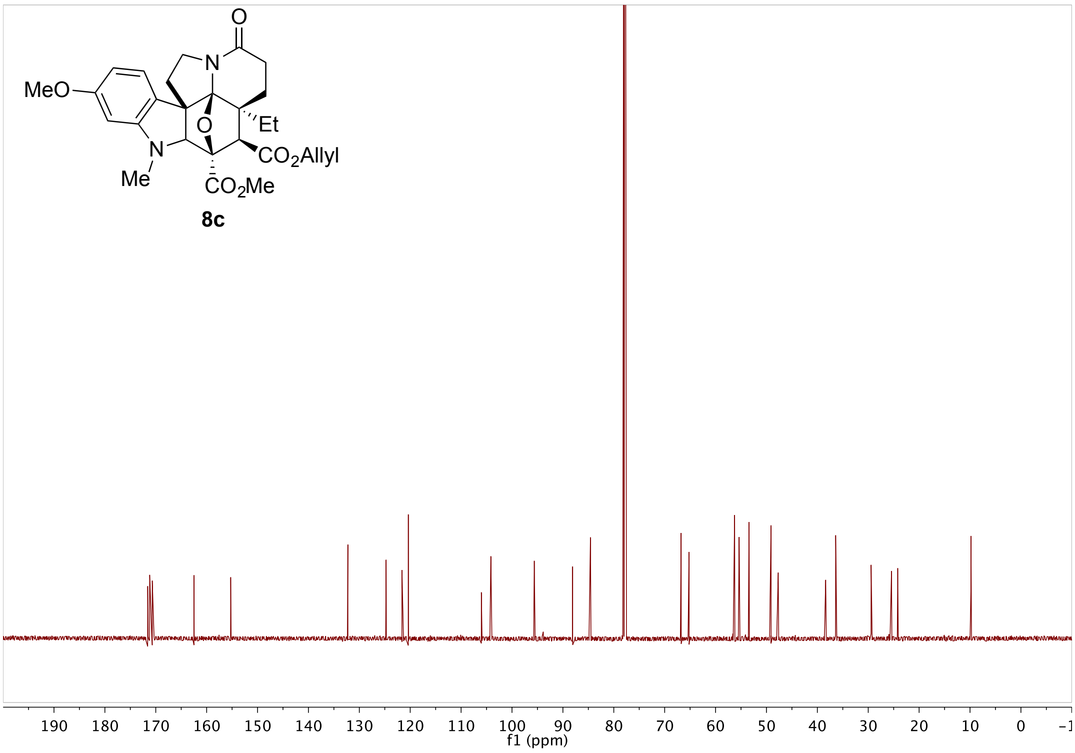
MeO
N
Me
O
N
O
Et
CO2Allyl
CO2Me
8c
190 180 170 160 150 140 130 120 110 100 90 80 70 60 50 40 30 20 10 0 -1
f1 (ppm)

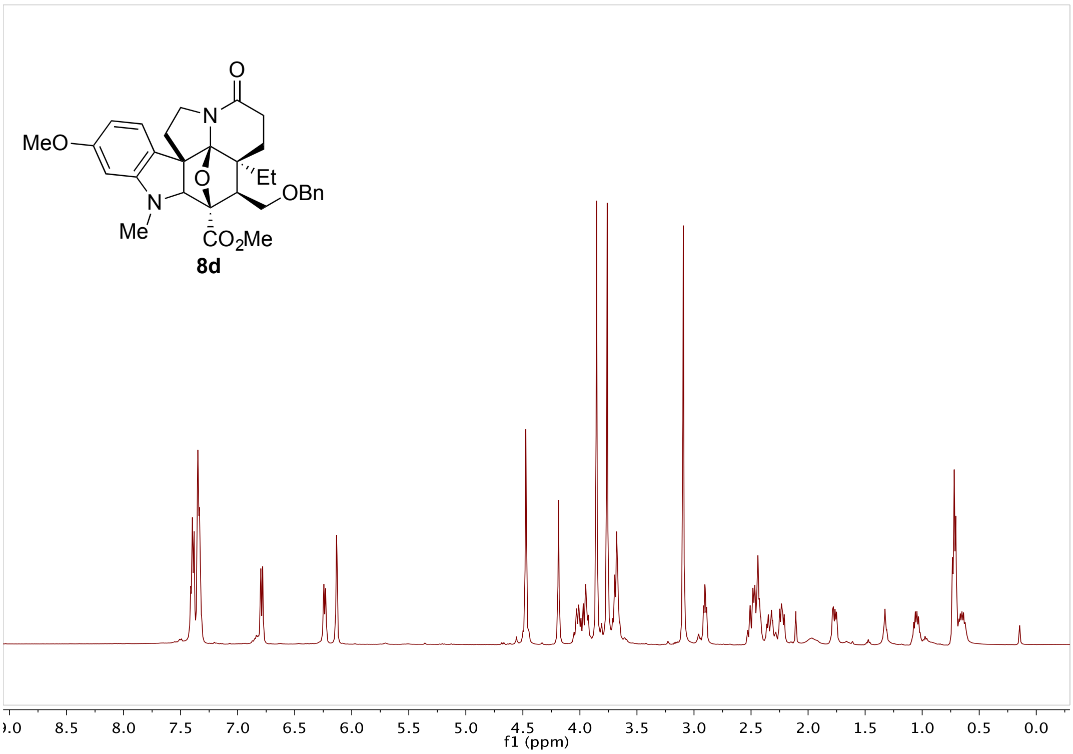
MeO
O
N
Et
OBn
Me
CO2Me
8d
f1 (ppm)

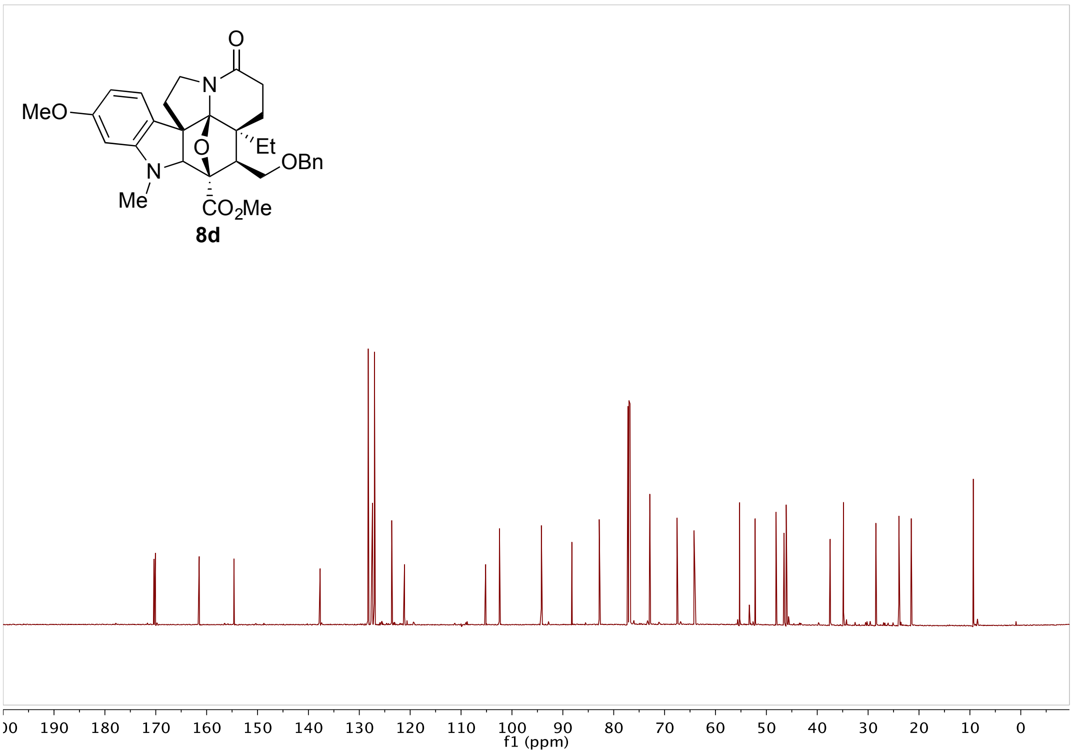
MeO
O
N
Et
OBn
Me
CO2Me
8d
f1 (ppm)

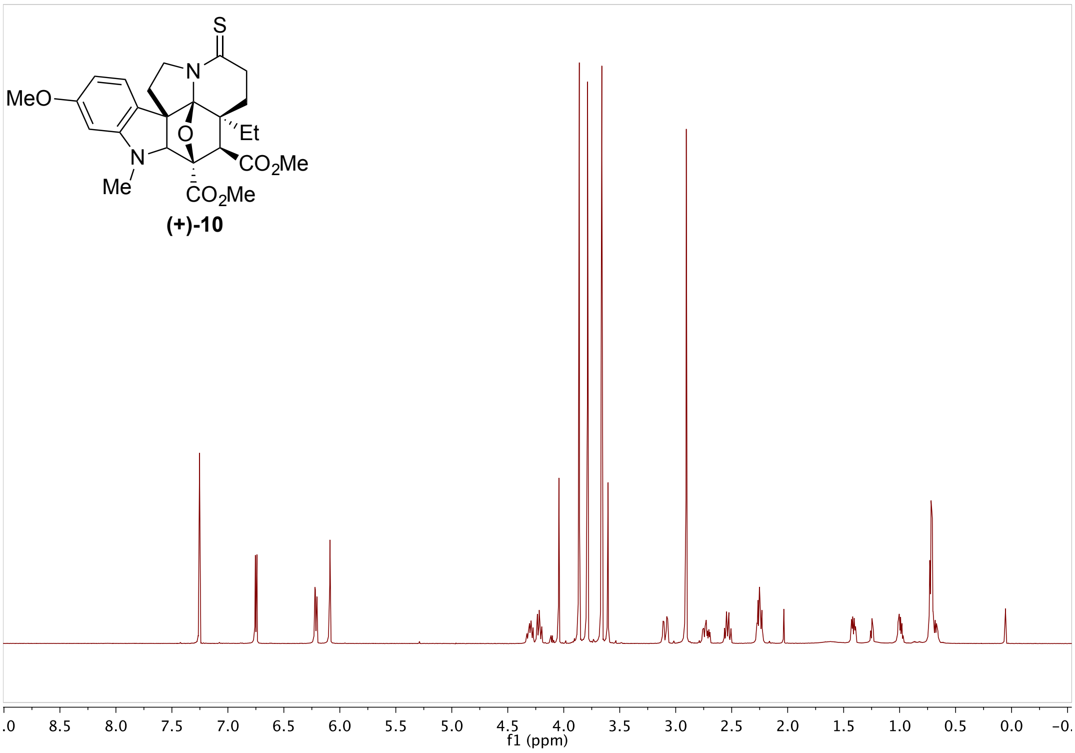

MeO
N
Me
S
N
O
Et
CO_2Me
CO_2Me
(+)-10
8.5
8.0
7.5
7.0
6.5
6.0
5.5
5.0
4.5
4.0
3.5
3.0
2.5
2.0
1.5
1.0
0.5
0.0
f1 (ppm)

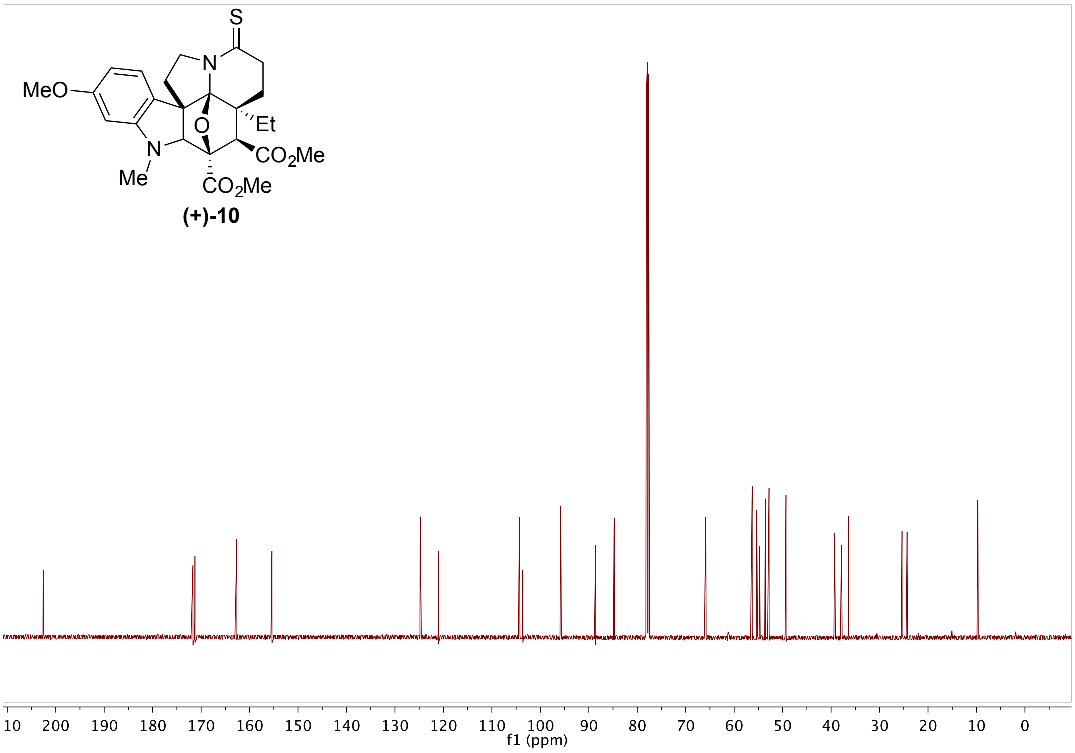

MeO
N
Me
S
N
O
Et
CO_2Me
CO_2Me
(+)-10
200
190
180
170
160
150
140
130
120
110
100
90
80
70
60
50
40
30
20
10
0
f1 (ppm)

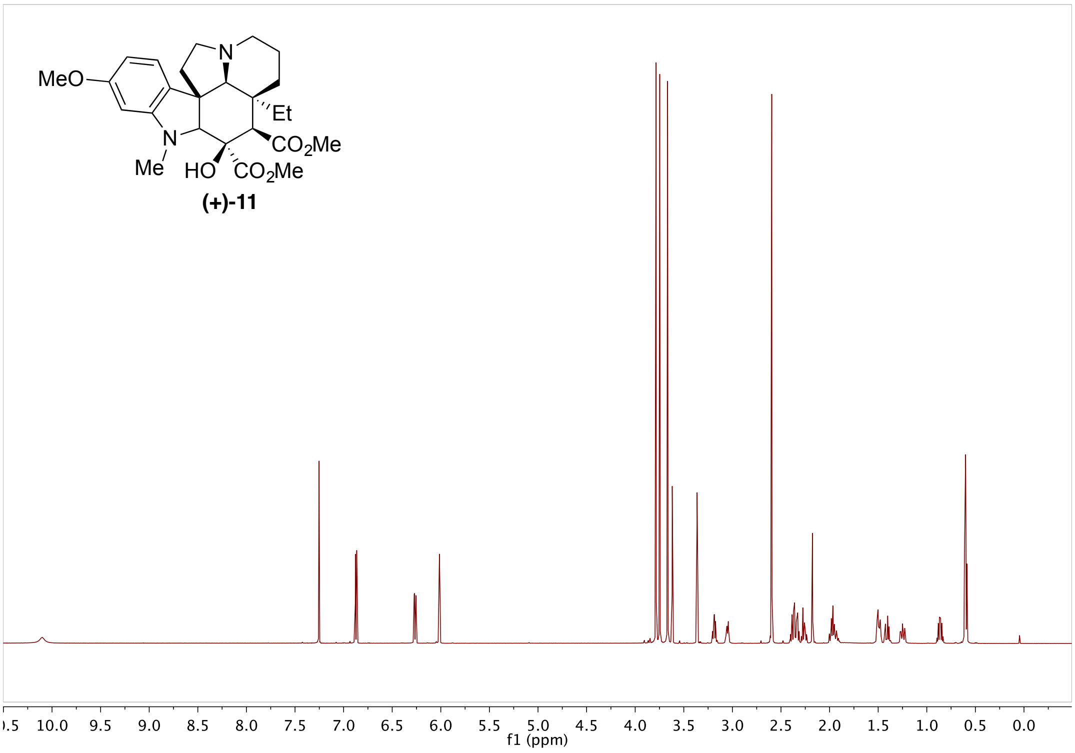
MeO
N
Me
HO
CO2Me
CO2Me
Et
N
(+)-11
f1 (ppm)

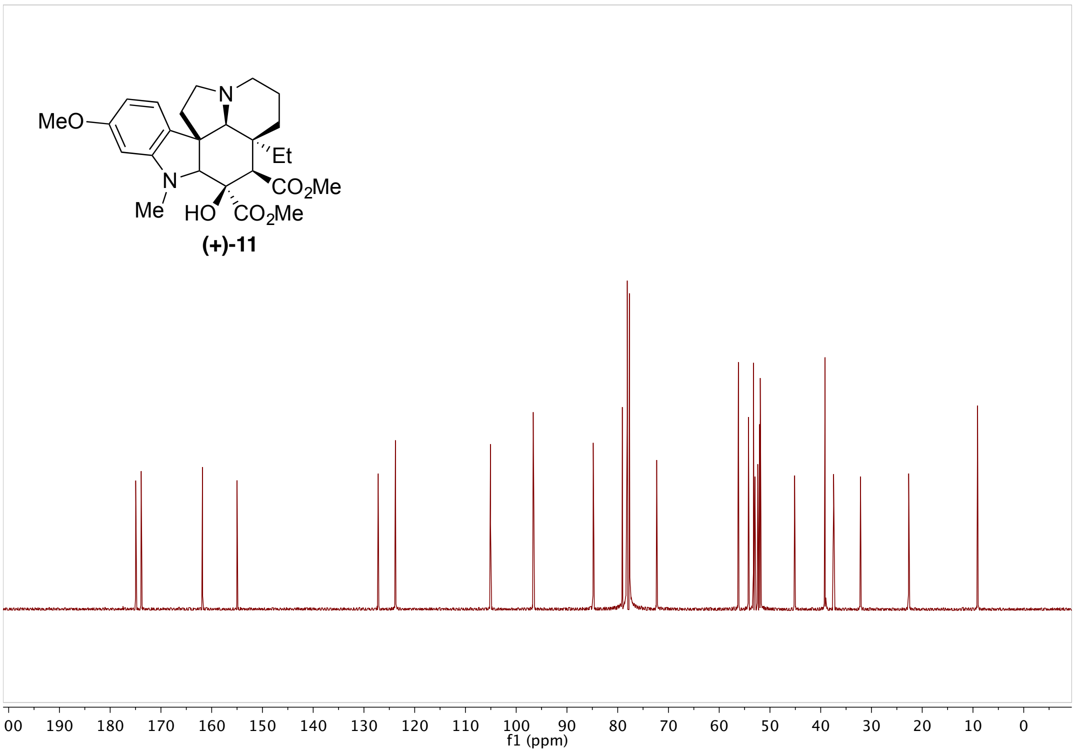
MeO
N
Me
HO
CO2Me
CO2Me
Et
N
(+)-11
f1 (ppm)

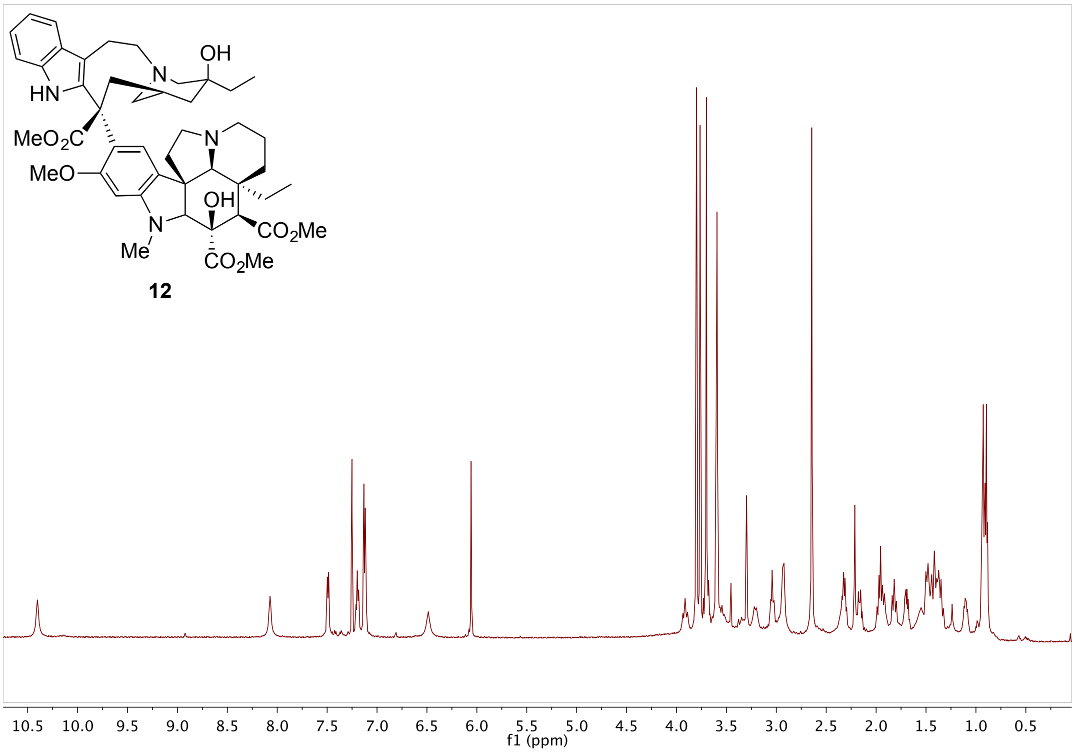
OH
N
HN
MeO2C
MeO
N
OH
CO2Me
Me
CO2Me
12
10.5
10.0
9.5
9.0
8.5
8.0
7.5
7.0
6.5
6.0
5.5
5.0
4.5
4.0
3.5
3.0
2.5
2.0
1.5
1.0
0.5
f1 (ppm)

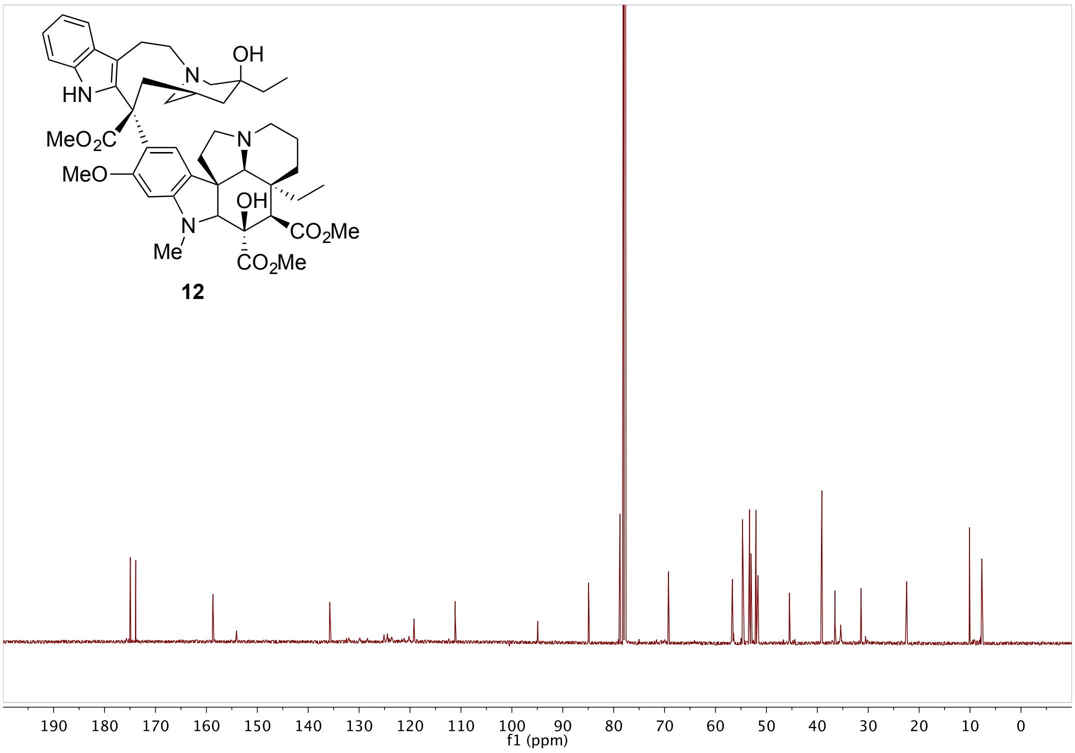
OH
N
HN
MeO2C
MeO
N
OH
CO2Me
Me
CO2Me
12
190
180
170
160
150
140
130
120
110
100
90
80
70
60
50
40
30
20
10
0
f1 (ppm)

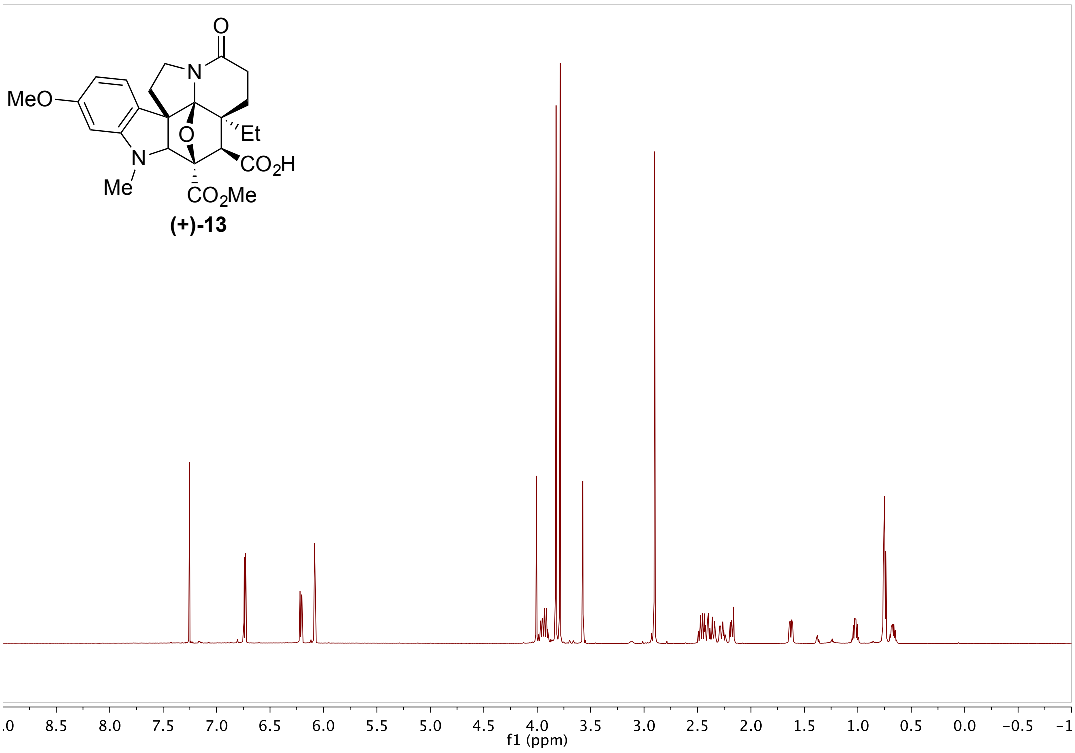
MeO
N
Me
O
N
O
Et
CO2H
CO2Me
(+)-13
f1 (ppm)

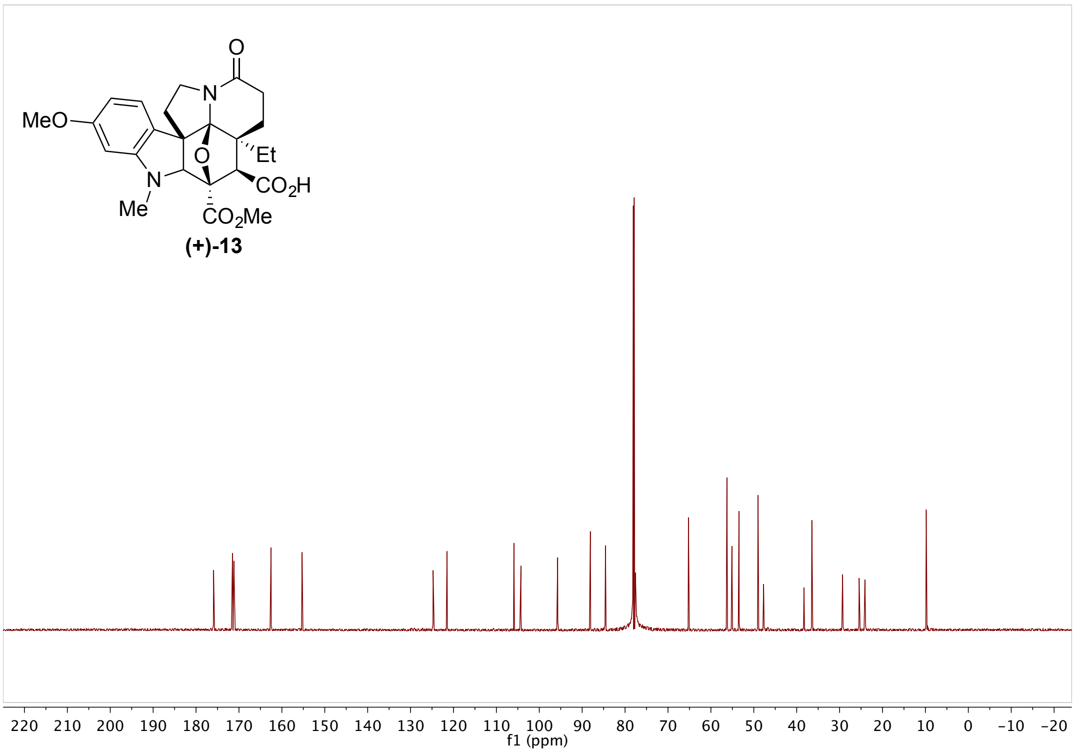
MeO
N
Me
O
N
O
Et
CO2H
CO2Me
(+)-13
f1 (ppm)

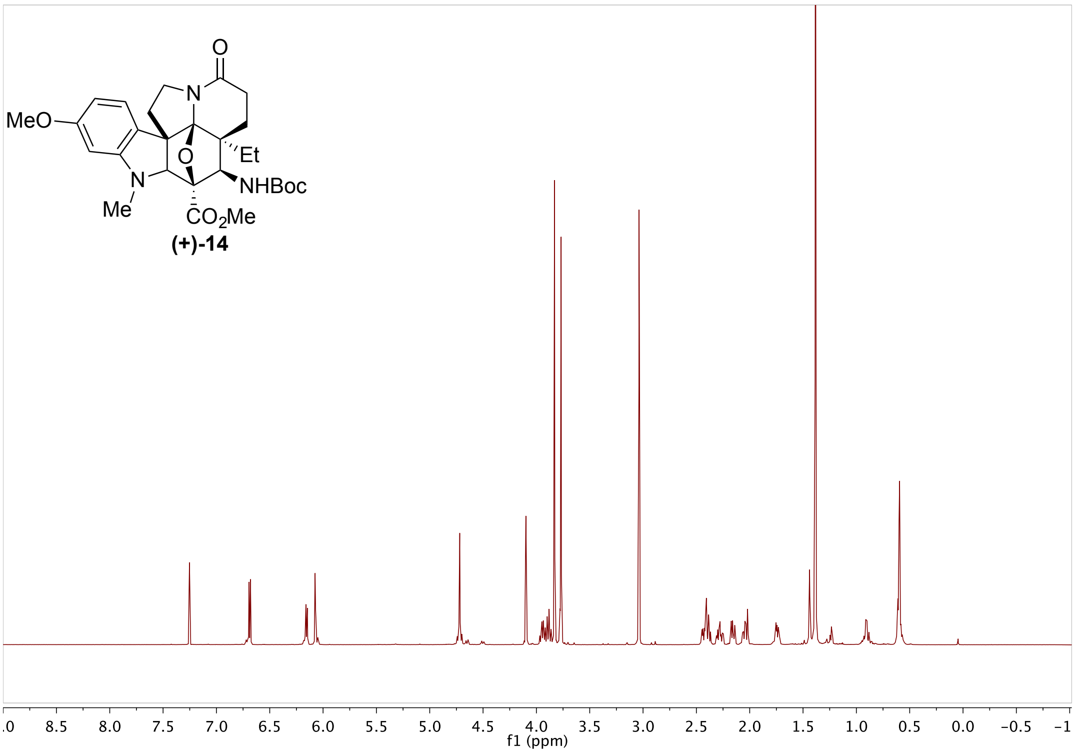
MeO
N
Me
O
N
O
Et
NHBoc
CO2Me
(+)-14
f1 (ppm)

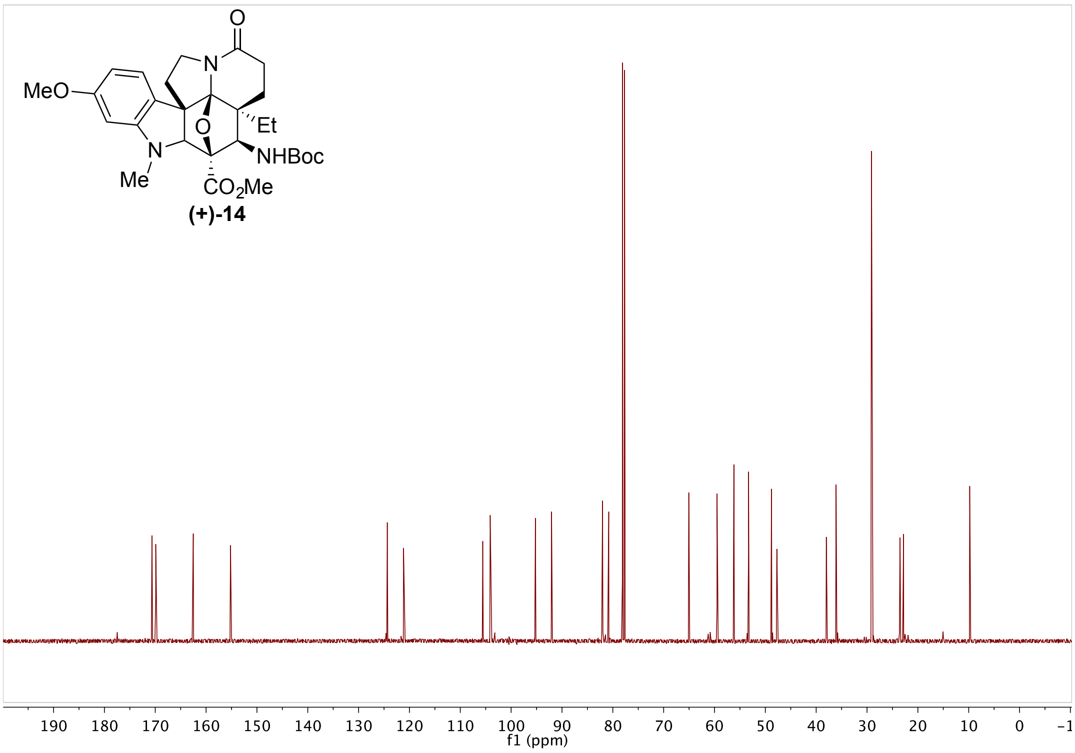
MeO
N
Me
O
N
O
Et
NHBoc
CO2Me
(+)-14
f1 (ppm)

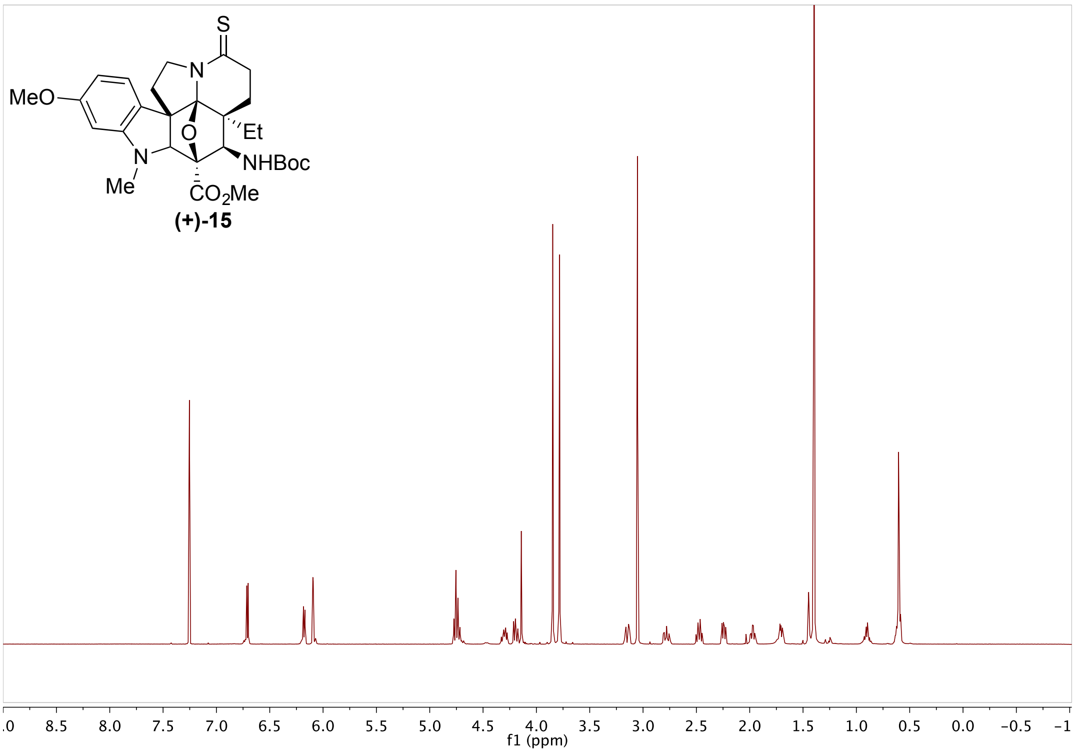
MeO
N
S
O
Et
NHBoc
N
Me
CO2Me
(+)-15
.0 8.5 8.0 7.5 7.0 6.5 6.0 5.5 5.0 4.5 4.0 3.5 3.0 2.5 2.0 1.5 1.0 0.5 0.0 −0.5 −1
f1 (ppm)

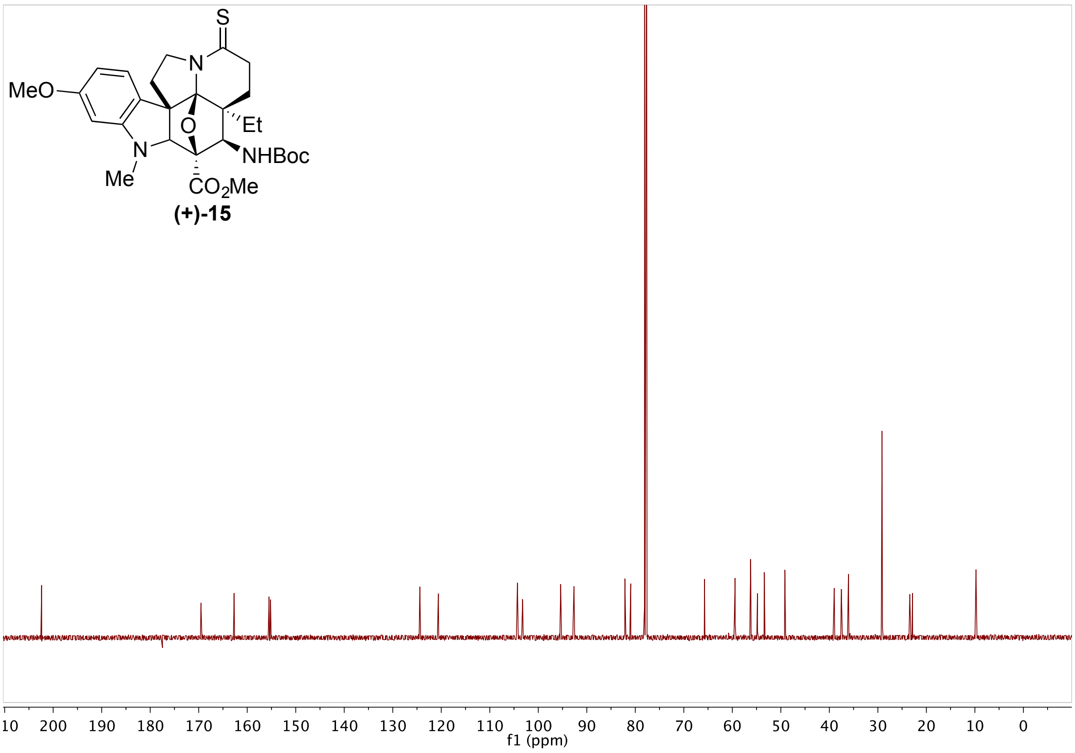
MeO
N
S
O
Et
NHBoc
N
Me
CO2Me
(+)-15
10 200 190 180 170 160 150 140 130 120 110 100 90 80 70 60 50 40 30 20 10 0
f1 (ppm)

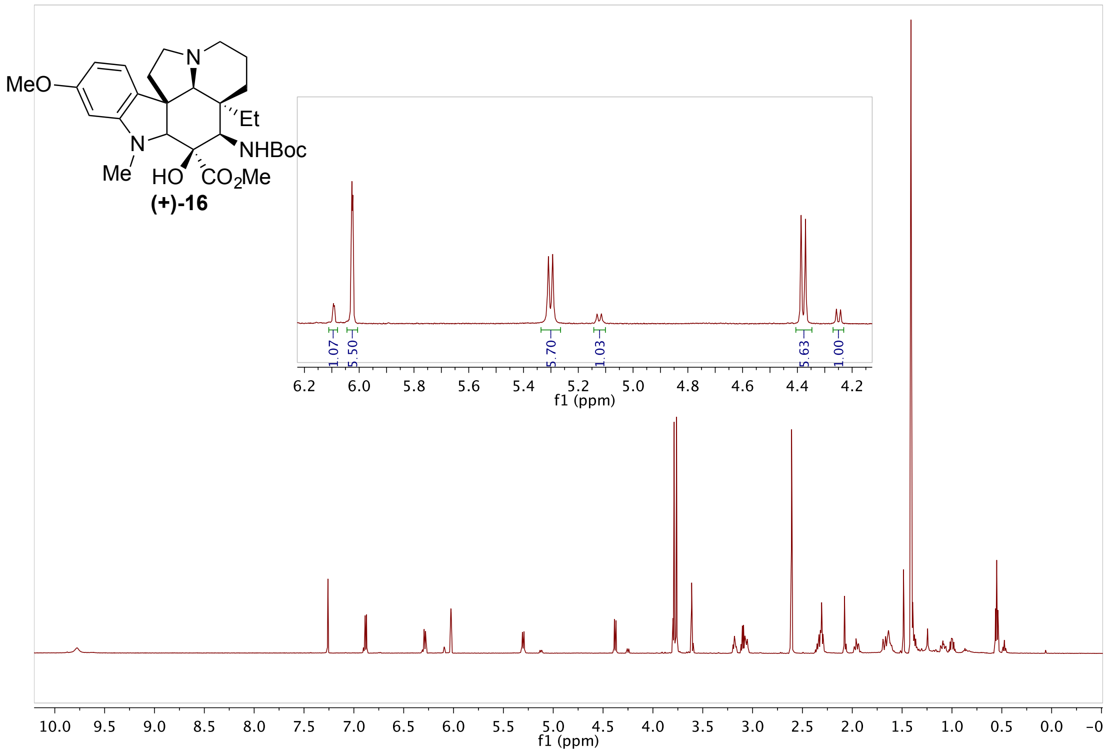
MeO
N
Et
NHBoc
N
Me
HO
CO_2Me
(+)-16
1.07
5.50
5.70
1.03
5.63
1.00
6.2 6.0 5.8 5.6 5.4 5.2 5.0 4.8 4.6 4.4 4.2
f1 (ppm)
10.0 9.5 9.0 8.5 8.0 7.5 7.0 6.5 6.0 5.5 5.0 4.5 4.0 3.5 3.0 2.5 2.0 1.5 1.0 0.5 0.0 -0
f1 (ppm)

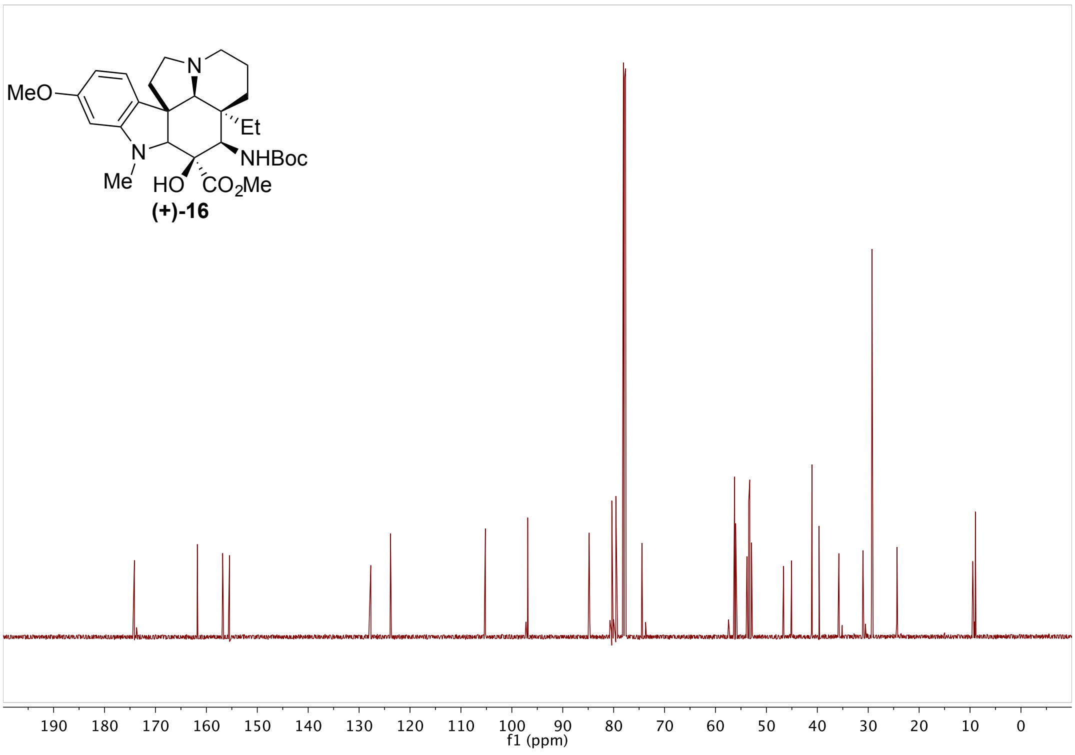
MeO
N
Et
NHBoc
N
Me
HO
CO_2Me
(+)-16
190 180 170 160 150 140 130 120 110 100 90 80 70 60 50 40 30 20 10 0
f1 (ppm)

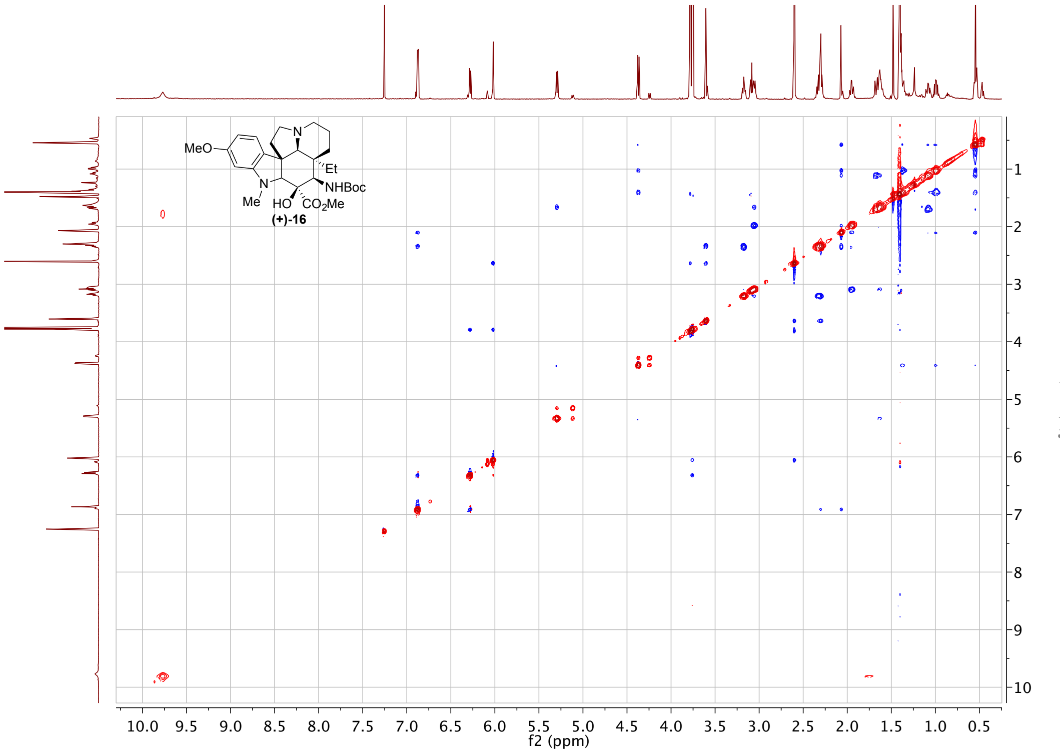
MeO
N
Et
NHBoc
Me
HO
CO2Me
(+)-16
f2 (ppm)

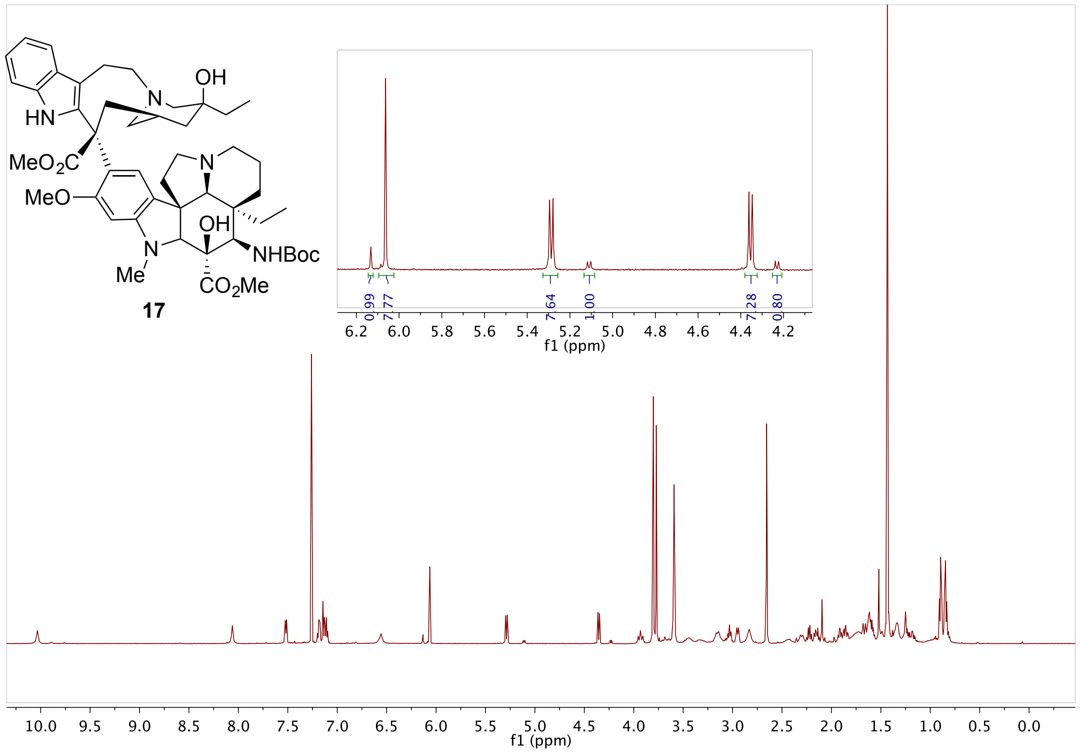
HN
N
OH
MeO2C
MeO
N
OH
NHBoc
Me
CO2Me
f1 (ppm)

17

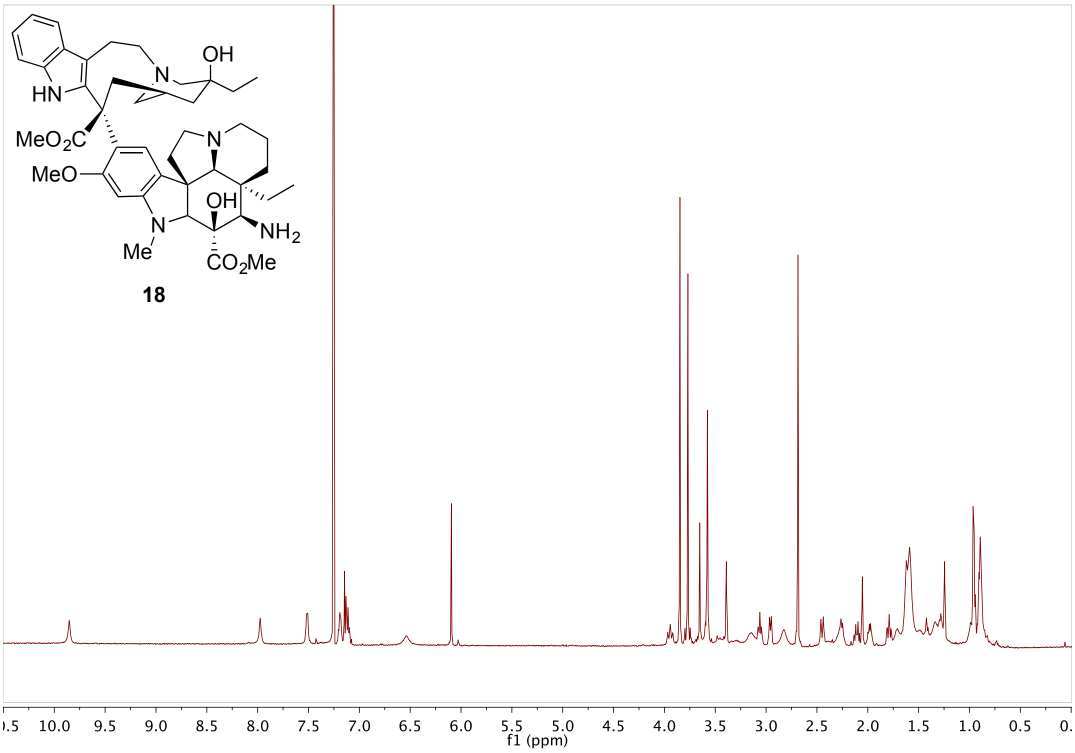
OH
HN
N
MeO2C
MeO
N
OH
NH2
N
Me
CO2Me
18
10.0 9.5 9.0 8.5 8.0 7.5 7.0 6.5 6.0 5.5 5.0 4.5 4.0 3.5 3.0 2.5 2.0 1.5 1.0 0.5
f1 (ppm)

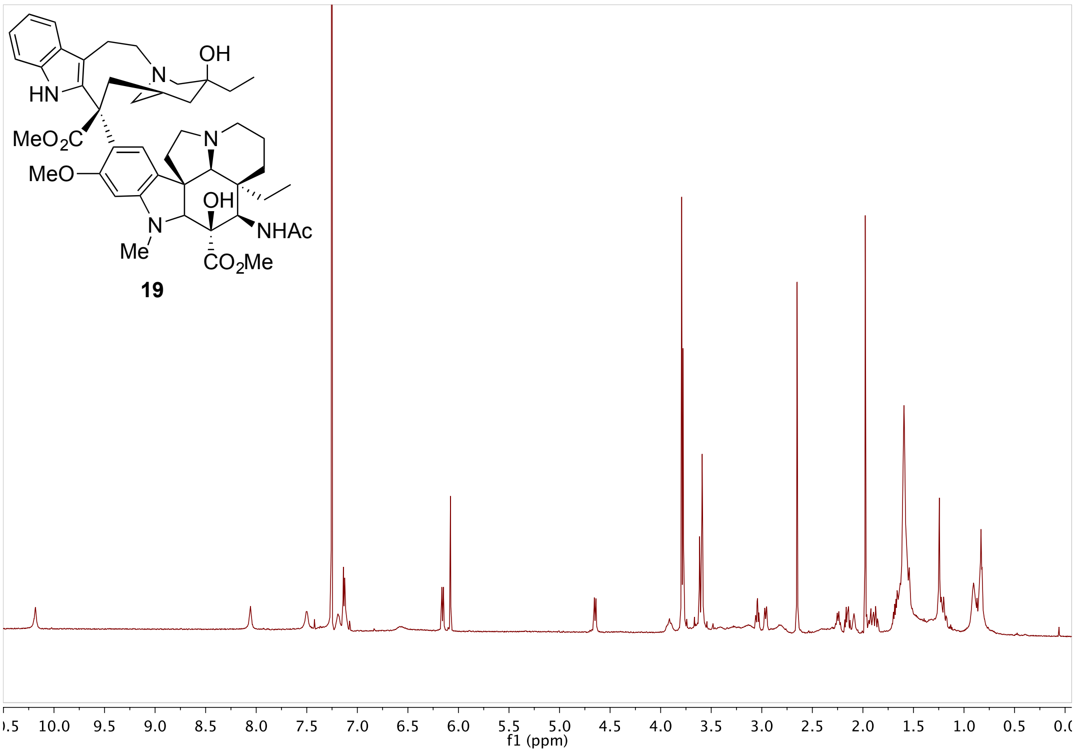
OH
HN
N
MeO2C
MeO
N
OH
NHAc
N
Me
CO2Me
19
10.0 9.5 9.0 8.5 8.0 7.5 7.0 6.5 6.0 5.5 5.0 4.5 4.0 3.5 3.0 2.5 2.0 1.5 1.0 0.5
f1 (ppm)

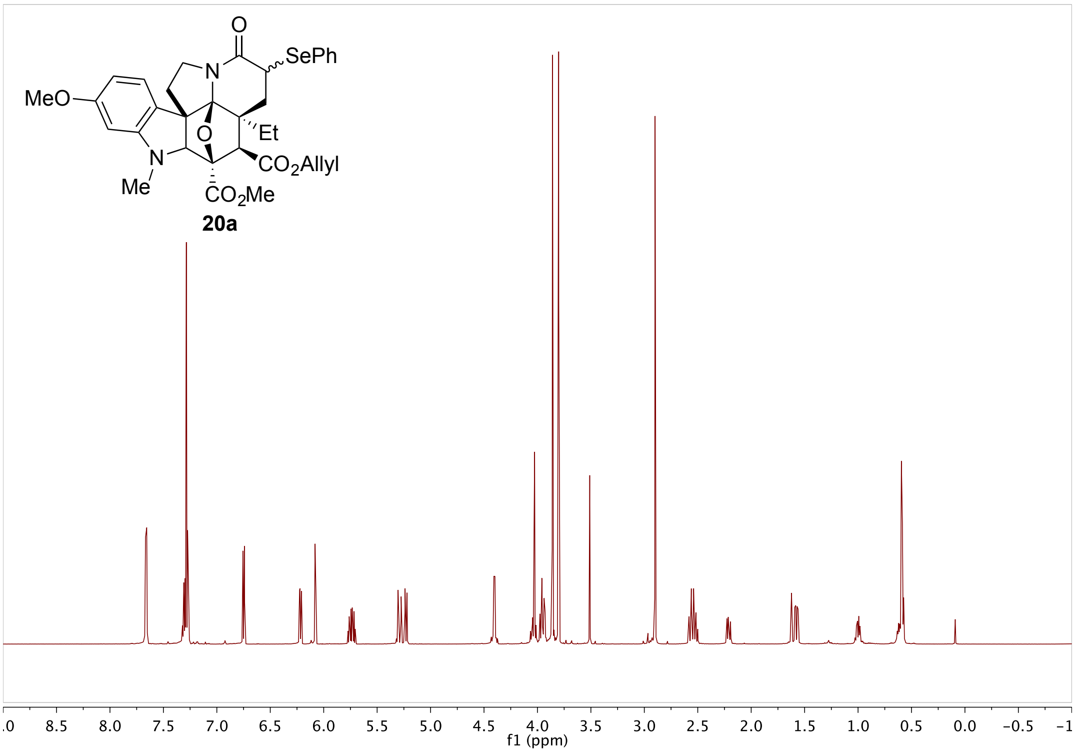

O
SePh
MeO
N
O
Et
N
CO2Allyl
Me
CO2Me
20a
.0
8.5
8.0
7.5
7.0
6.5
6.0
5.5
5.0
4.5
4.0
3.5
3.0
2.5
2.0
1.5
1.0
0.5
0.0
-0.5
-1
f1 (ppm)

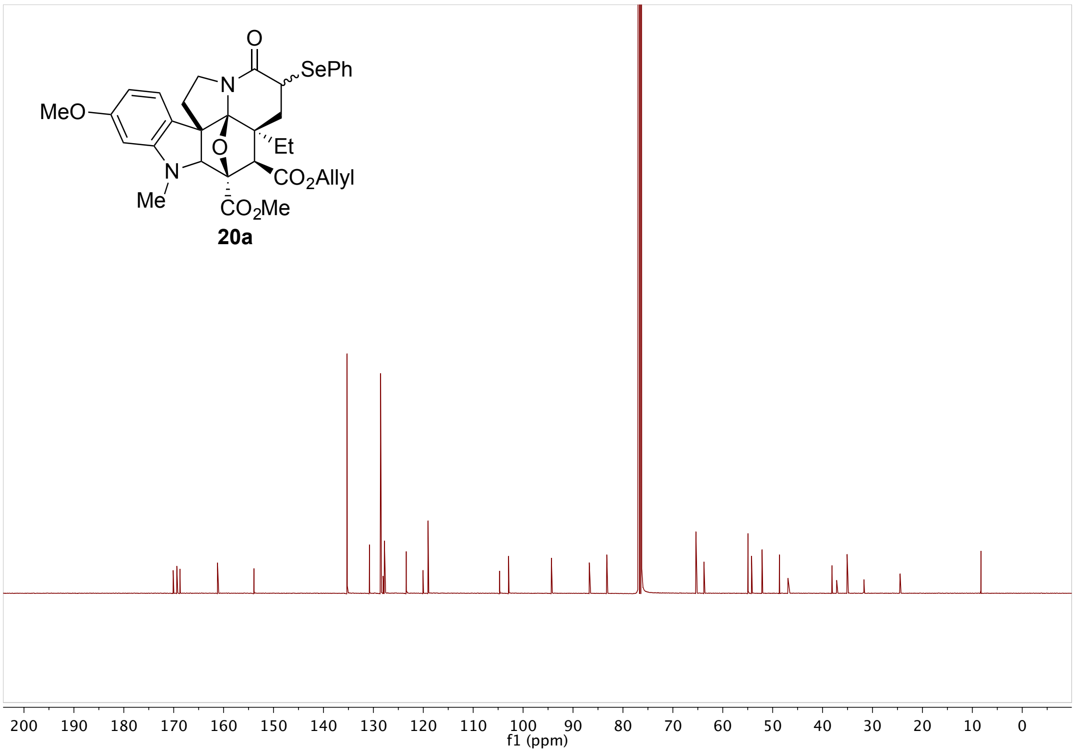

O
SePh
MeO
N
O
Et
N
CO2Allyl
Me
CO2Me
20a
200
190
180
170
160
150
140
130
120
110
100
90
80
70
60
50
40
30
20
10
0
f1 (ppm)

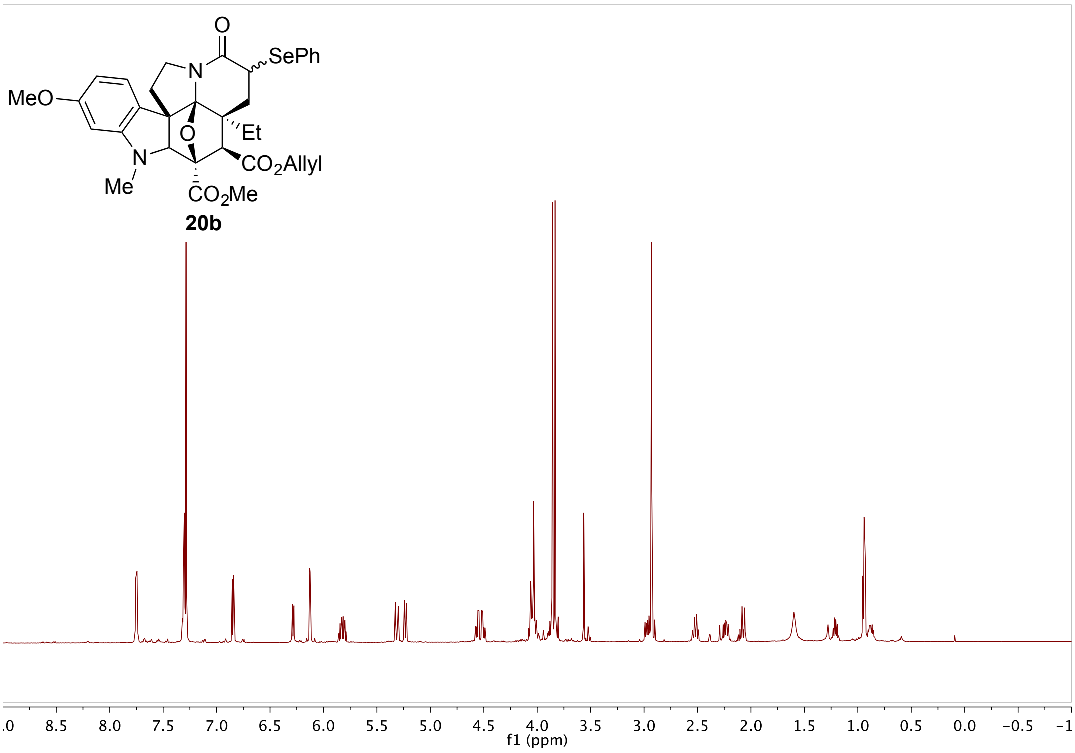
O
SePh
MeO
N
O
Et
CO_2Allyl
N
Me
CO_2Me
20b
f1 (ppm)

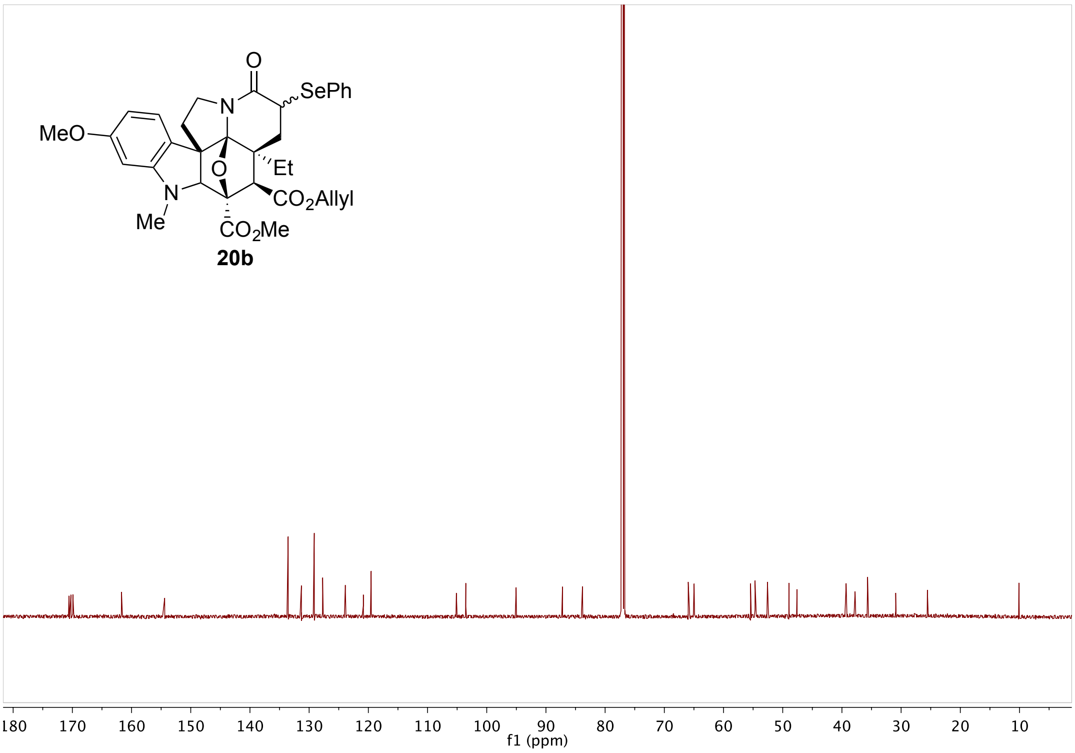
O
SePh
MeO
N
O
Et
CO_2Allyl
N
Me
CO_2Me
20b
f1 (ppm)

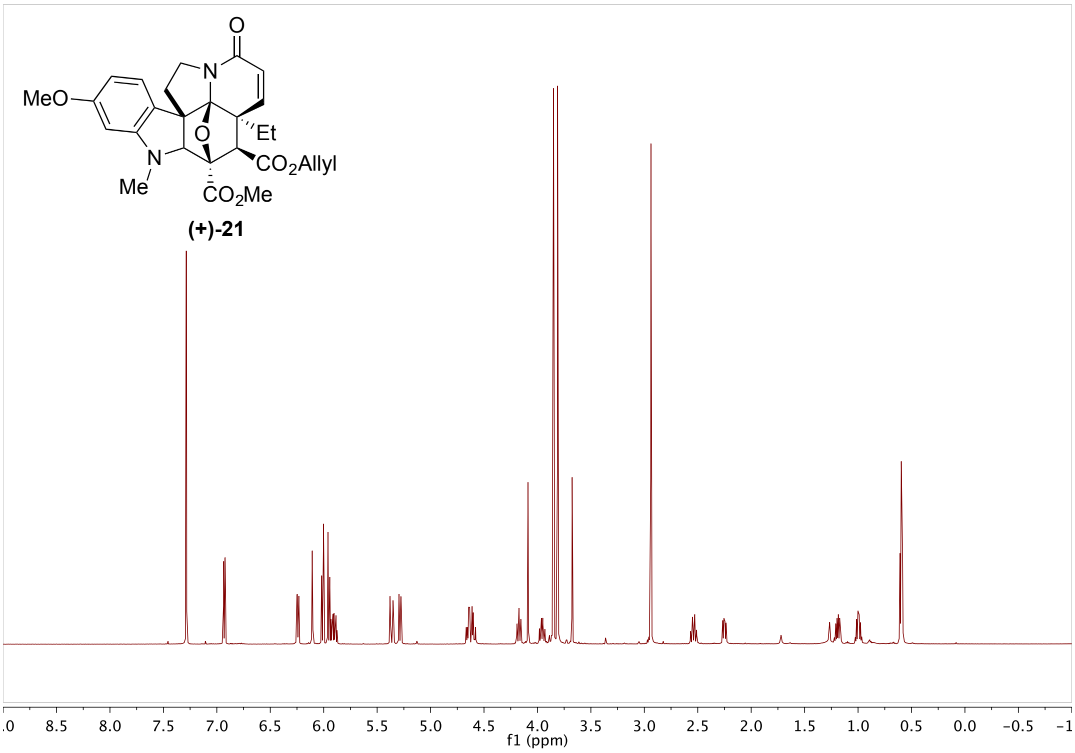
MeO
N
Me
O
N
O
Et
CO2Allyl
CO2Me
(+)-21
f1 (ppm)

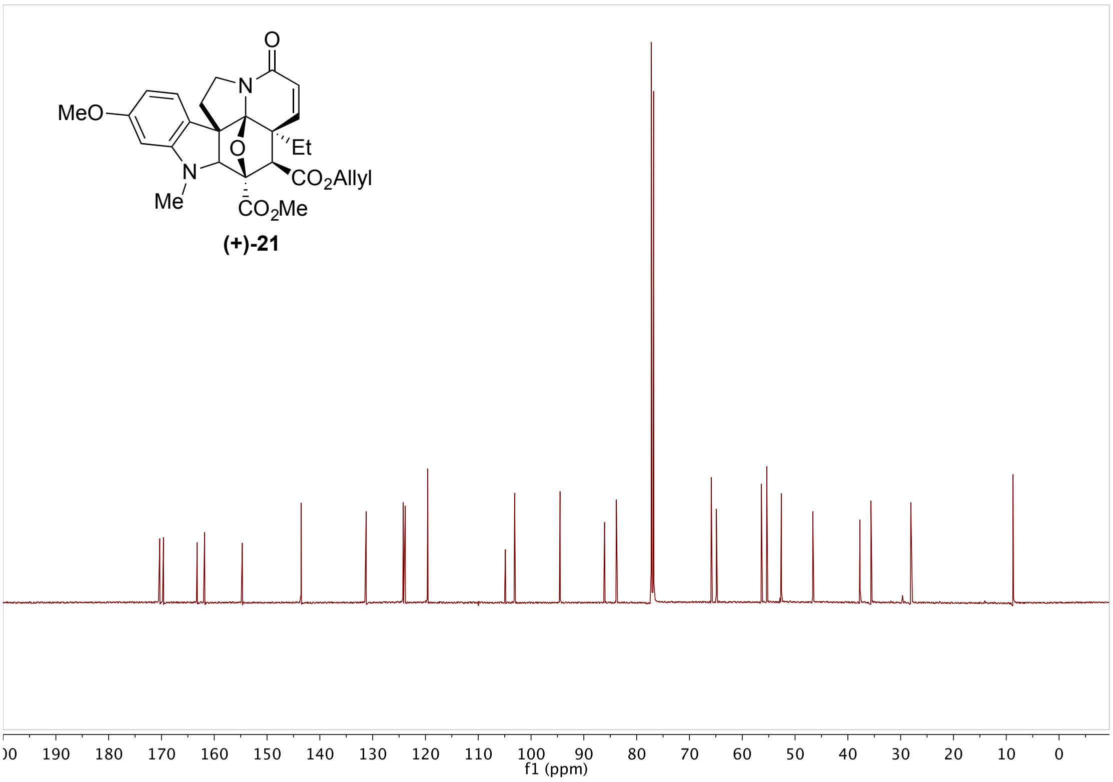
MeO
N
Me
O
N
O
Et
CO2Allyl
CO2Me
(+)-21
f1 (ppm)

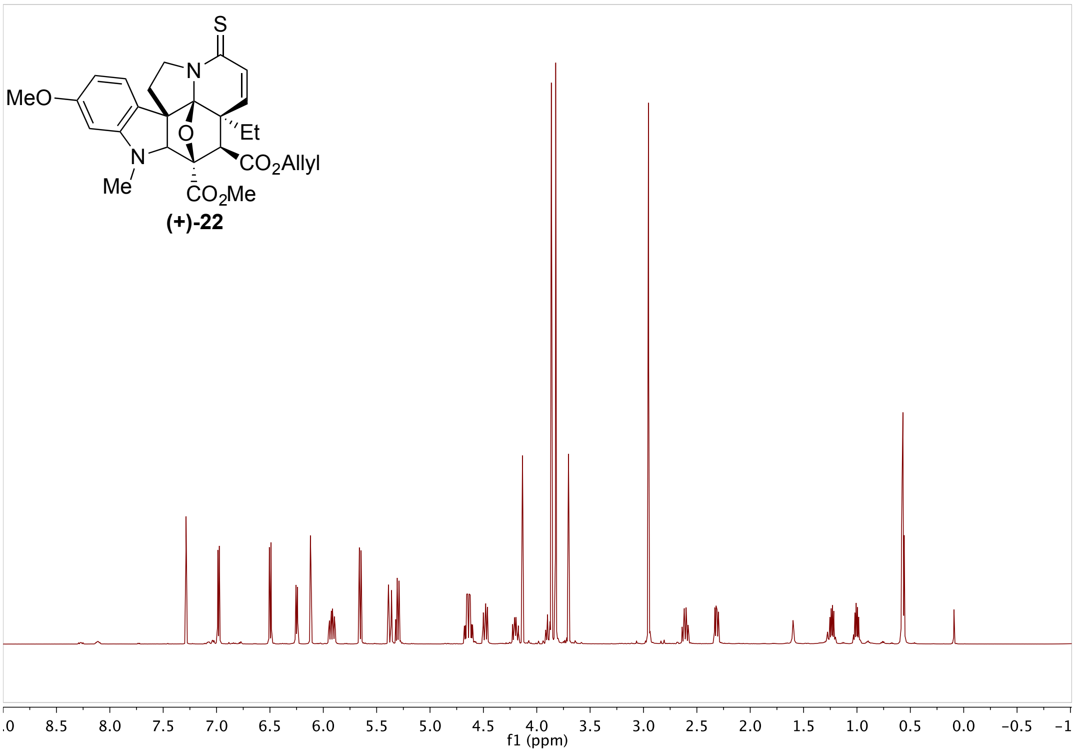
MeO
N
Me
S
O
Et
CO2Allyl
CO2Me
(+)-22
8.5 8.0 7.5 7.0 6.5 6.0 5.5 5.0 4.5 4.0 3.5 3.0 2.5 2.0 1.5 1.0 0.5 0.0 −0.5
f1 (ppm)

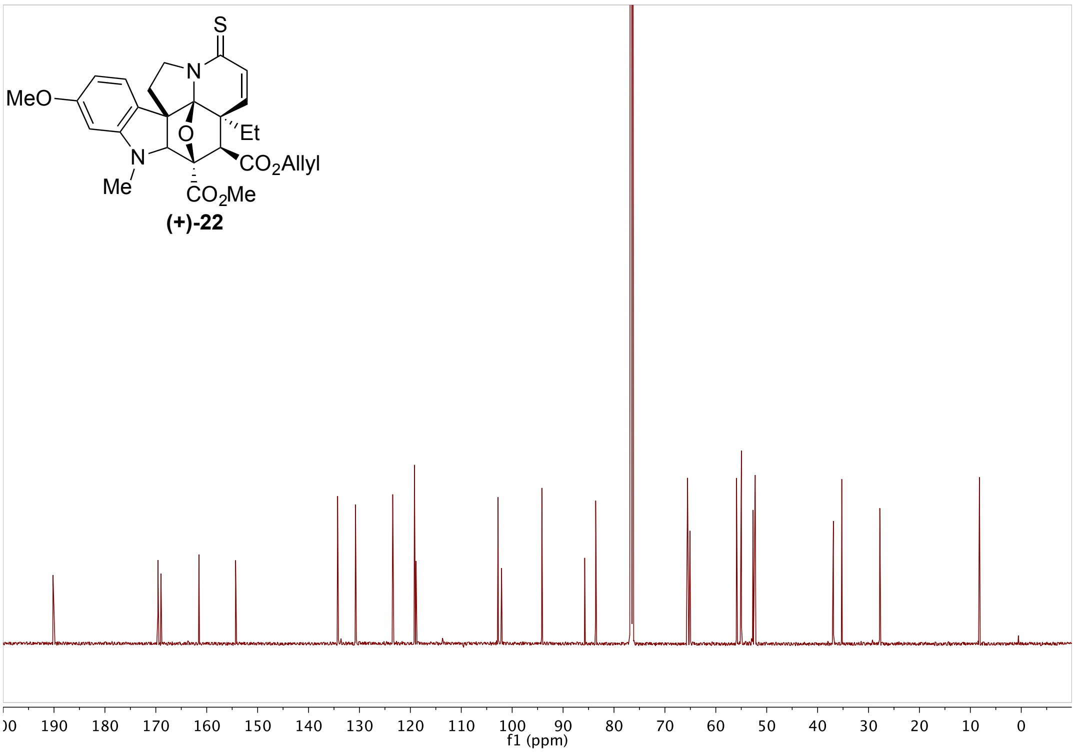
MeO
N
Me
S
O
Et
CO2Allyl
CO2Me
(+)-22
190 180 170 160 150 140 130 120 110 100 90 80 70 60 50 40 30 20 10 0
f1 (ppm)

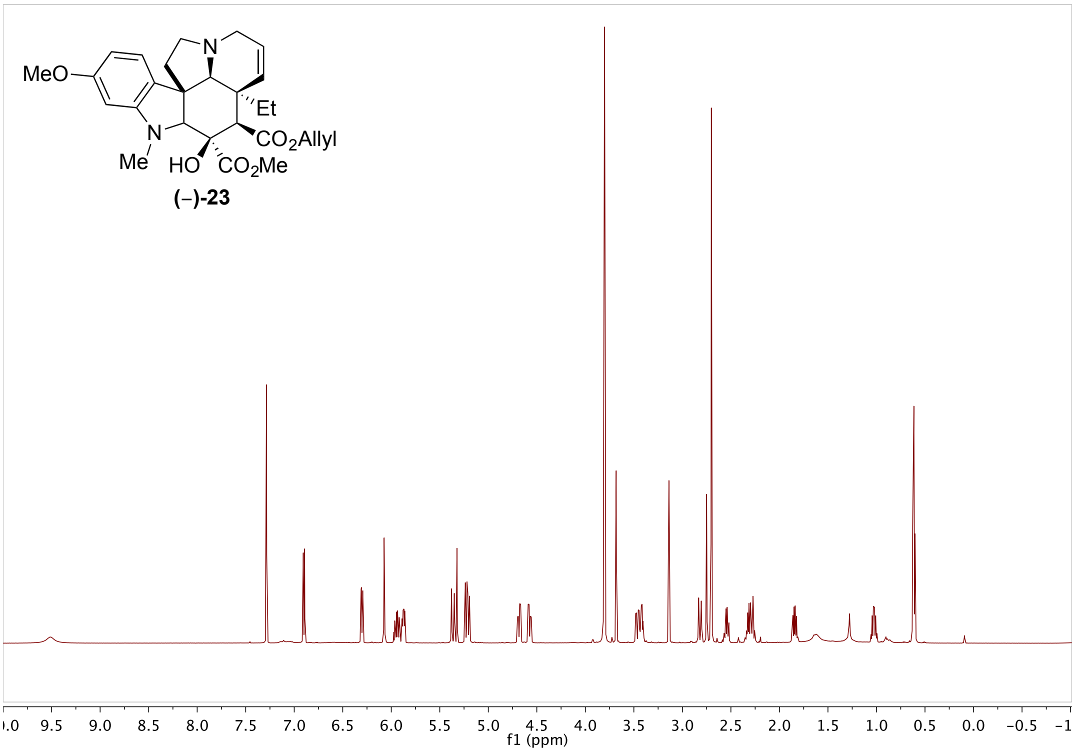
MeO
N
N
Me
HO
Et
CO2Allyl
CO2Me
(–)-23
f1 (ppm)

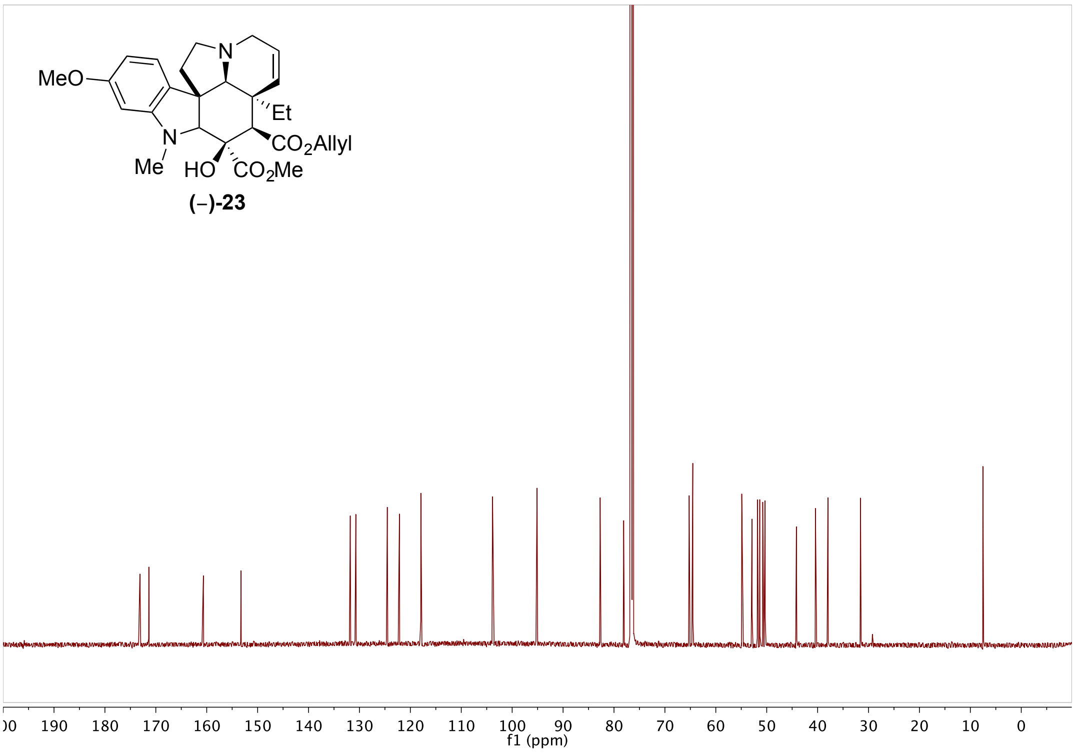
MeO
N
N
Me
HO
Et
CO2Allyl
CO2Me
(–)-23
f1 (ppm)

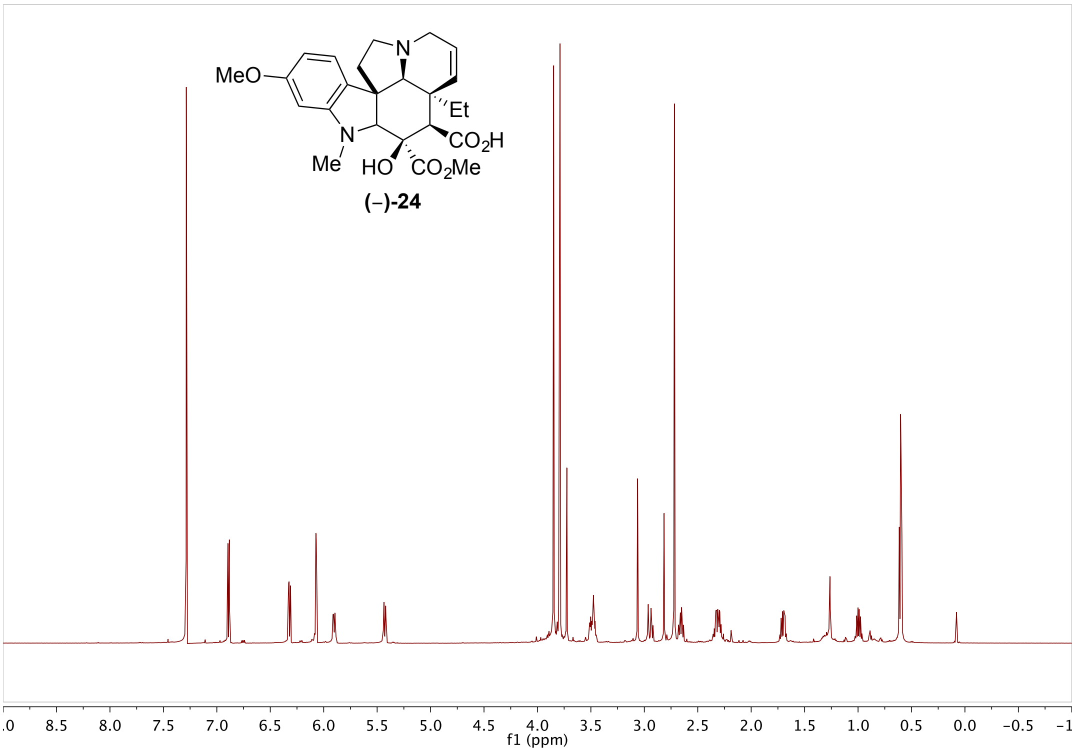
MeO
N
N
Me
HO
Et
CO_2H
CO_2Me
(–)-24
f1 (ppm)

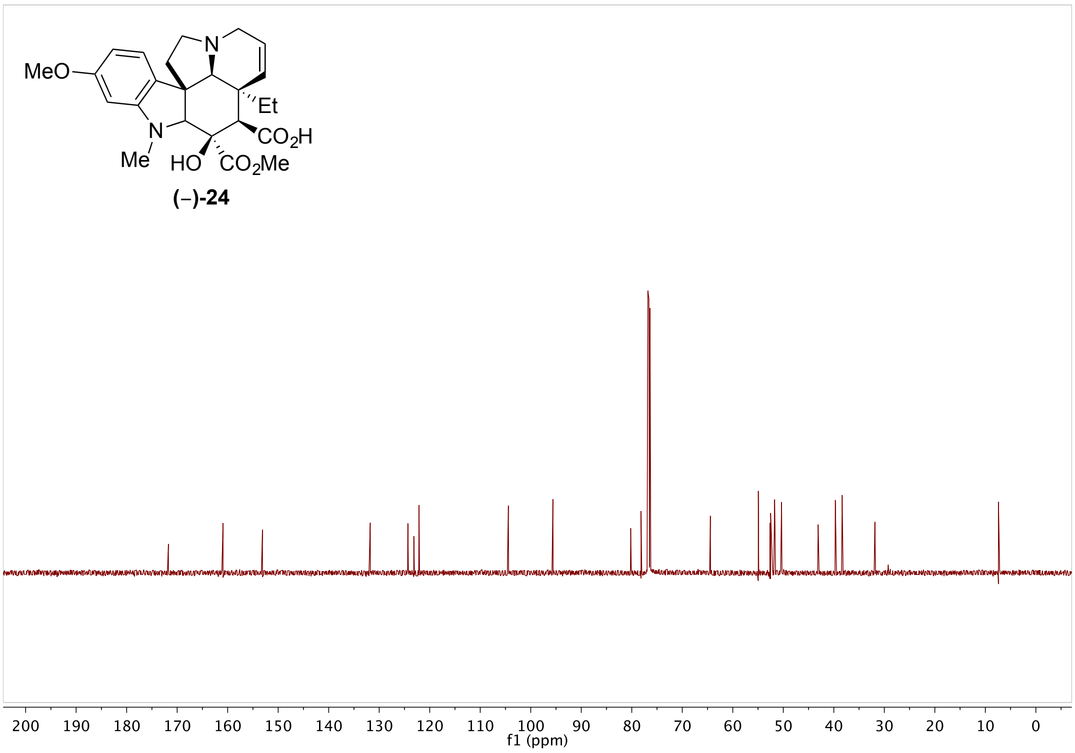
MeO
N
N
Me
HO
Et
CO_2H
CO_2Me
(–)-24
f1 (ppm)

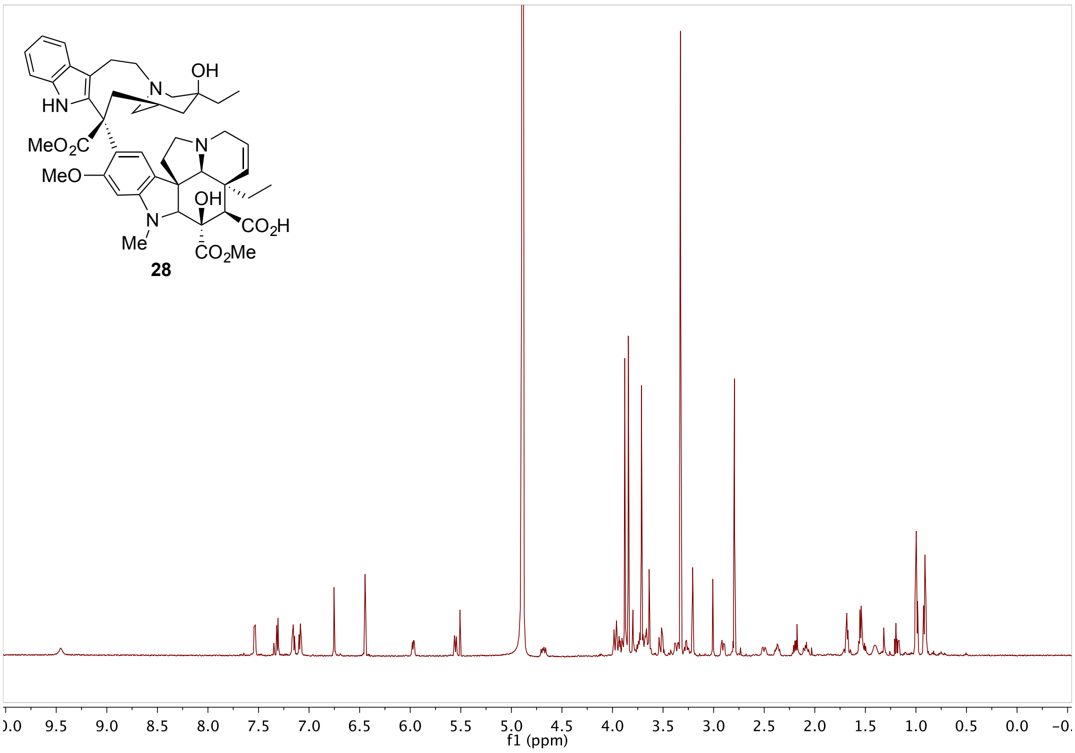

OH
N
HN
MeO2C
MeO
OH
CO2H
N
Me
CO2Me
28
0.0 9.5 9.0 8.5 8.0 7.5 7.0 6.5 6.0 5.5 5.0 4.5 4.0 3.5 3.0 2.5 2.0 1.5 1.0 0.5 0.0 −0.
f1 (ppm)

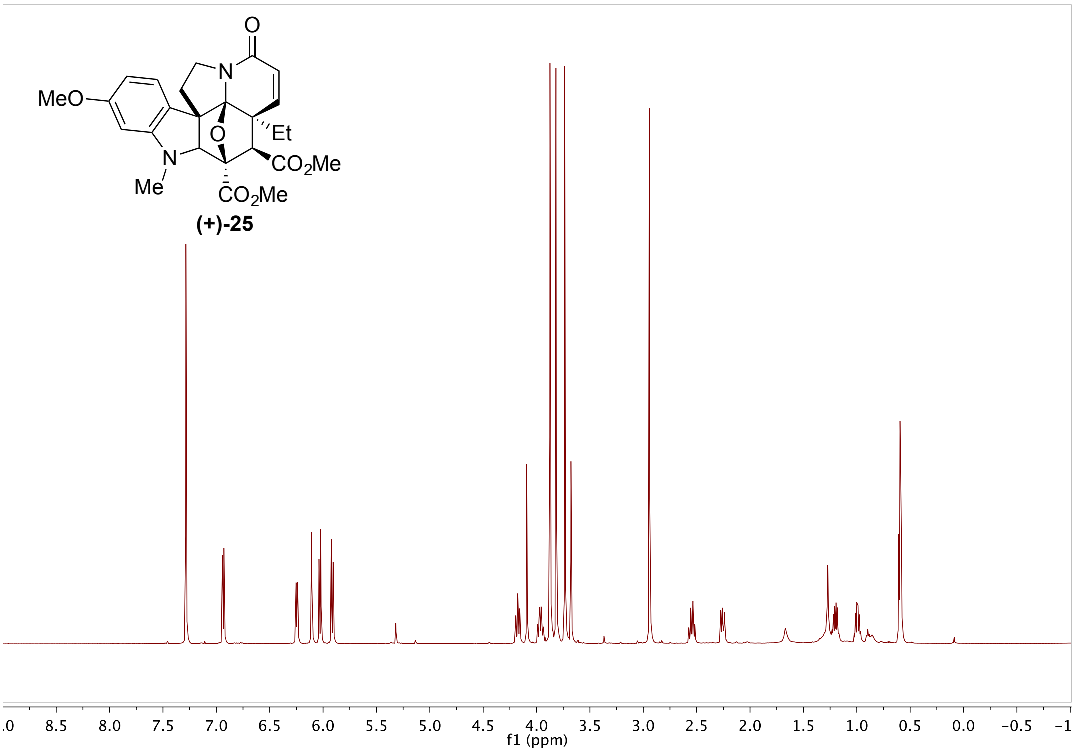

O
N
MeO
O
Et
N
CO2Me
Me
CO2Me
(+)-25
.0 8.5 8.0 7.5 7.0 6.5 6.0 5.5 5.0 4.5 4.0 3.5 3.0 2.5 2.0 1.5 1.0 0.5 0.0 −0.5 −1
f1 (ppm)

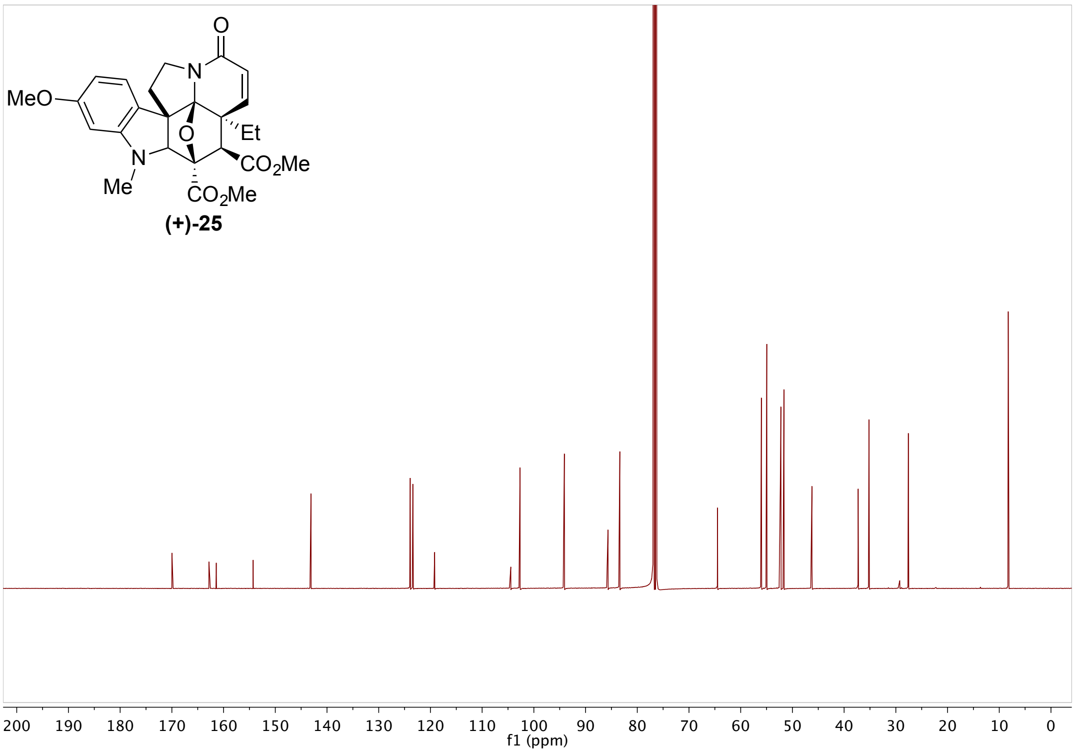
MeO
N
O
Et
CO_2Me
Me
CO_2Me
(+)-25
200 190 180 170 160 150 140 130 120 110 100 90 80 70 60 50 40 30 20 10 0
f1 (ppm)

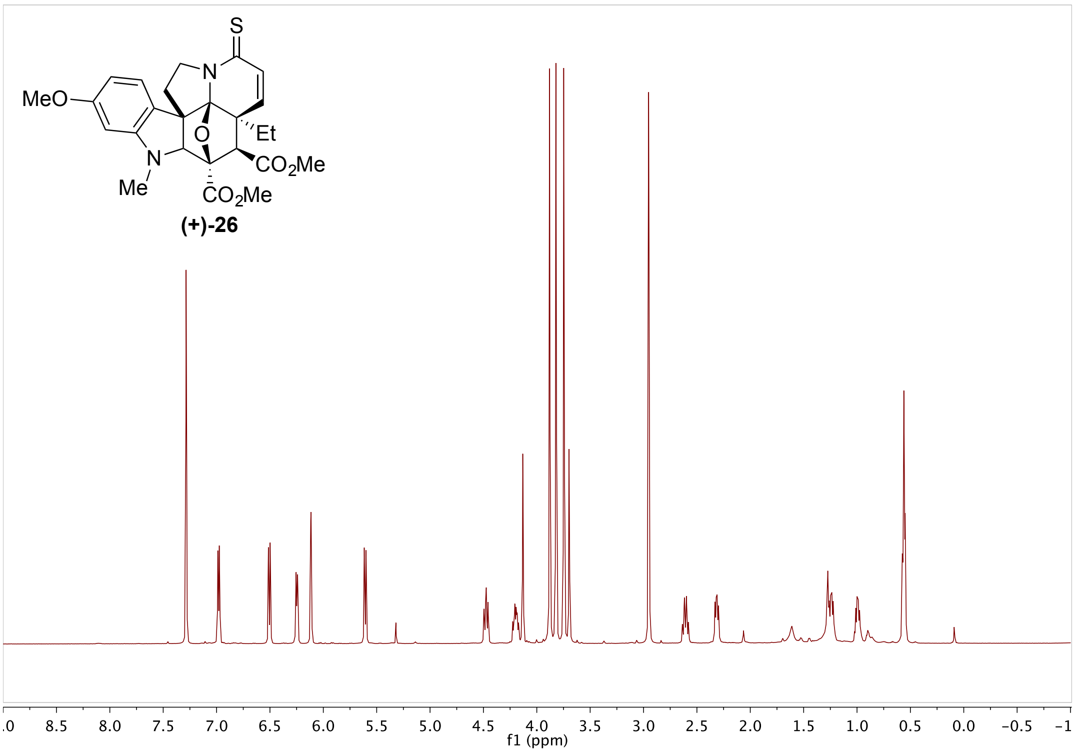
S
MeO
N
O
Et
CO_2Me
Me
CO_2Me
(+)-26
8.5 8.0 7.5 7.0 6.5 6.0 5.5 5.0 4.5 4.0 3.5 3.0 2.5 2.0 1.5 1.0 0.5 0.0 −0.5
f1 (ppm)

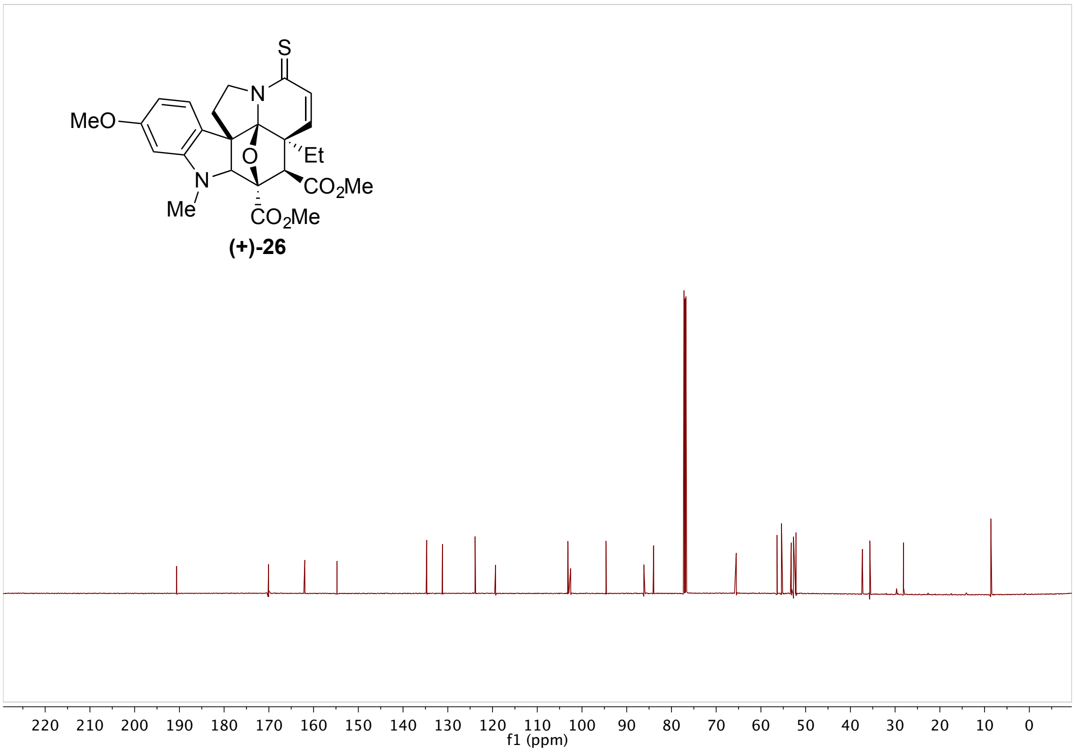
S
MeO
N
O
Et
CO2Me
Me
CO2Me
(+)-26
220 210 200 190 180 170 160 150 140 130 120 110 100 90 80 70 60 50 40 30 20 10 0
f1 (ppm)

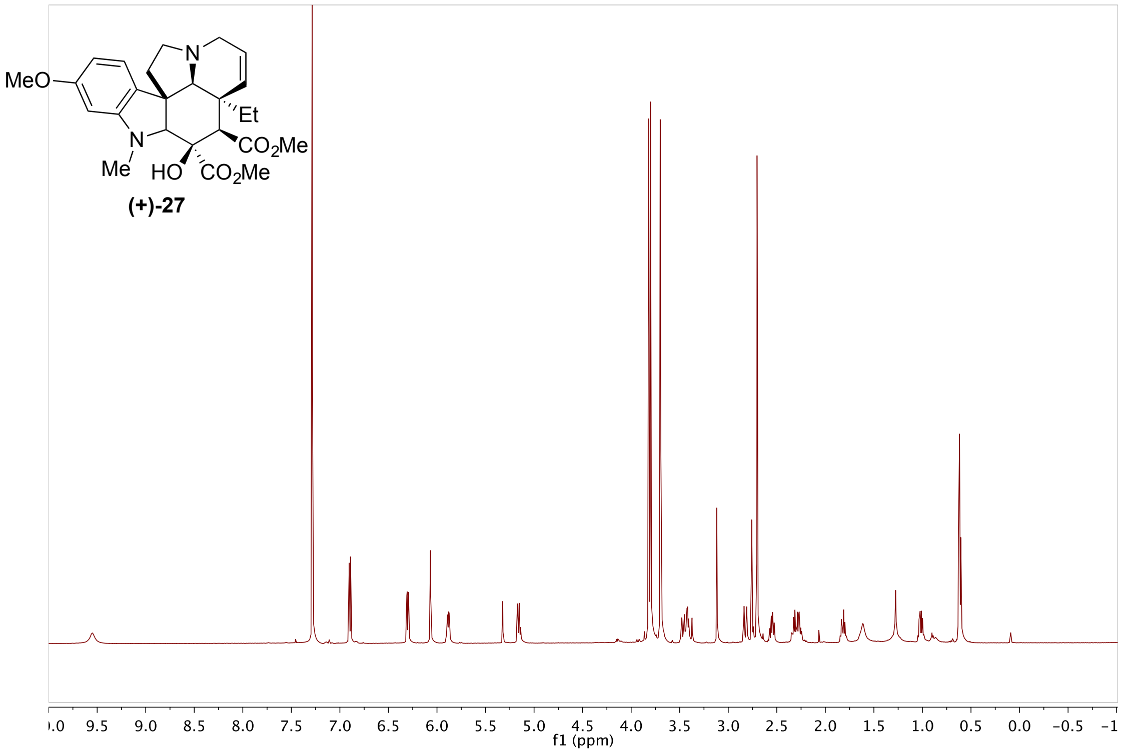
MeO
N
Et
CO2Me
N
Me
HO
CO2Me
(+)-27
9.5 9.0 8.5 8.0 7.5 7.0 6.5 6.0 5.5 5.0 4.5 4.0 3.5 3.0 2.5 2.0 1.5 1.0 0.5 0.0 −0.5
f1 (ppm)

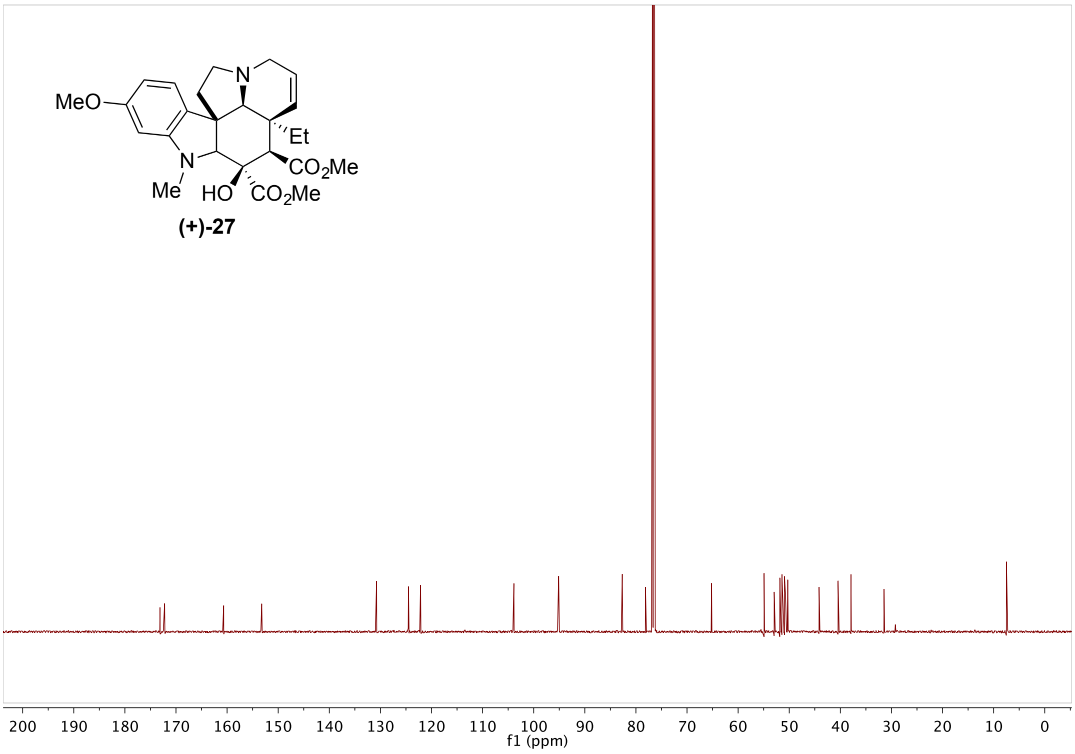
MeO
N
Me
HO
CO2Me
CO2Me
Et
N
(+)-27
200 190 180 170 160 150 140 130 120 110 100 90 80 70 60 50 40 30 20 10 0
f1 (ppm)

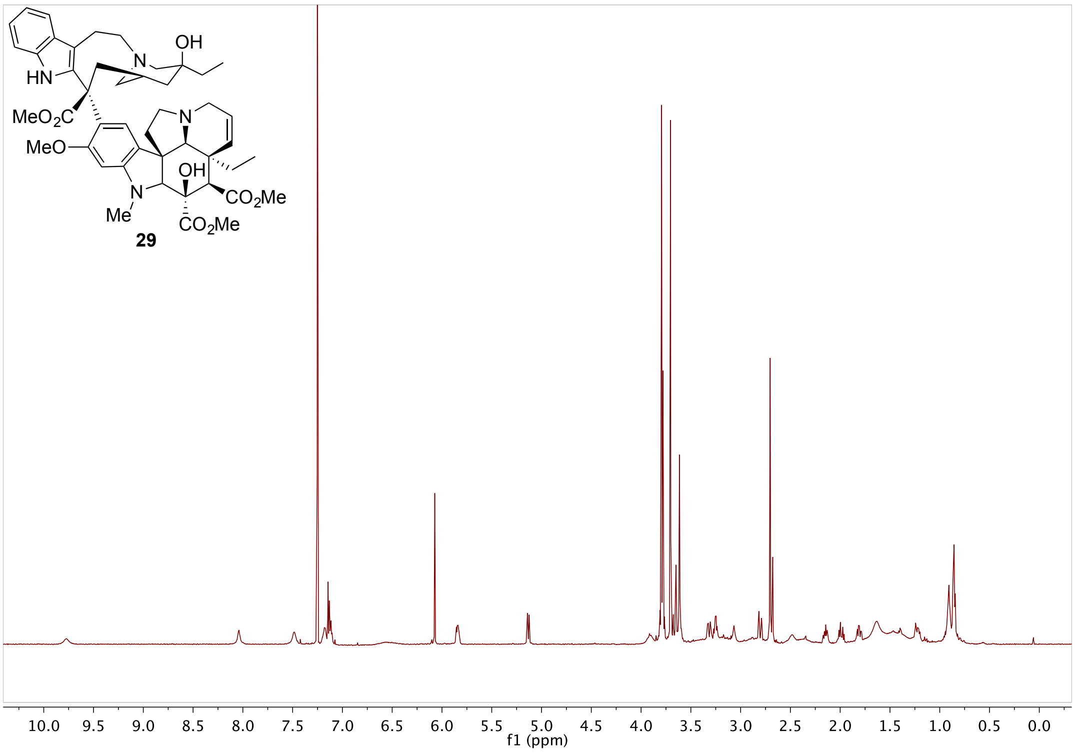
HN
N
OH
MeO2C
MeO
N
Me
OH
CO2Me
CO2Me
N
29
10.0 9.5 9.0 8.5 8.0 7.5 7.0 6.5 6.0 5.5 5.0 4.5 4.0 3.5 3.0 2.5 2.0 1.5 1.0 0.5 0.0
f1 (ppm)

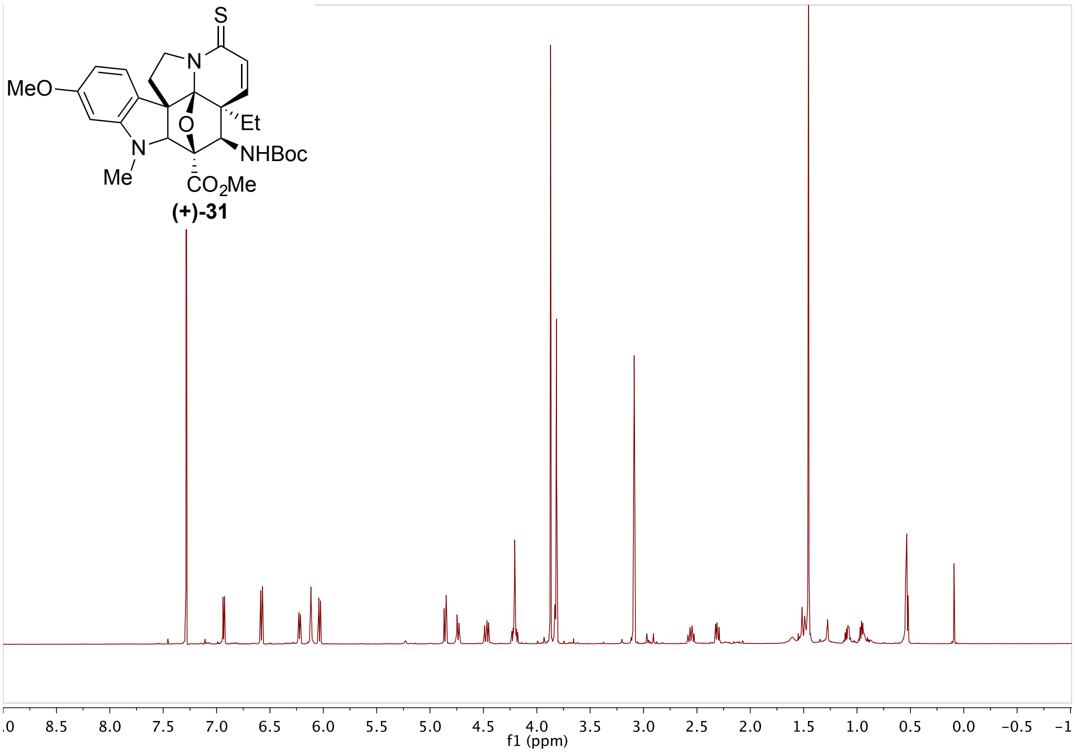

MeO
S
N
O
Et
NHBoc
N
Me
CO2Me
(+)-31
f1 (ppm)

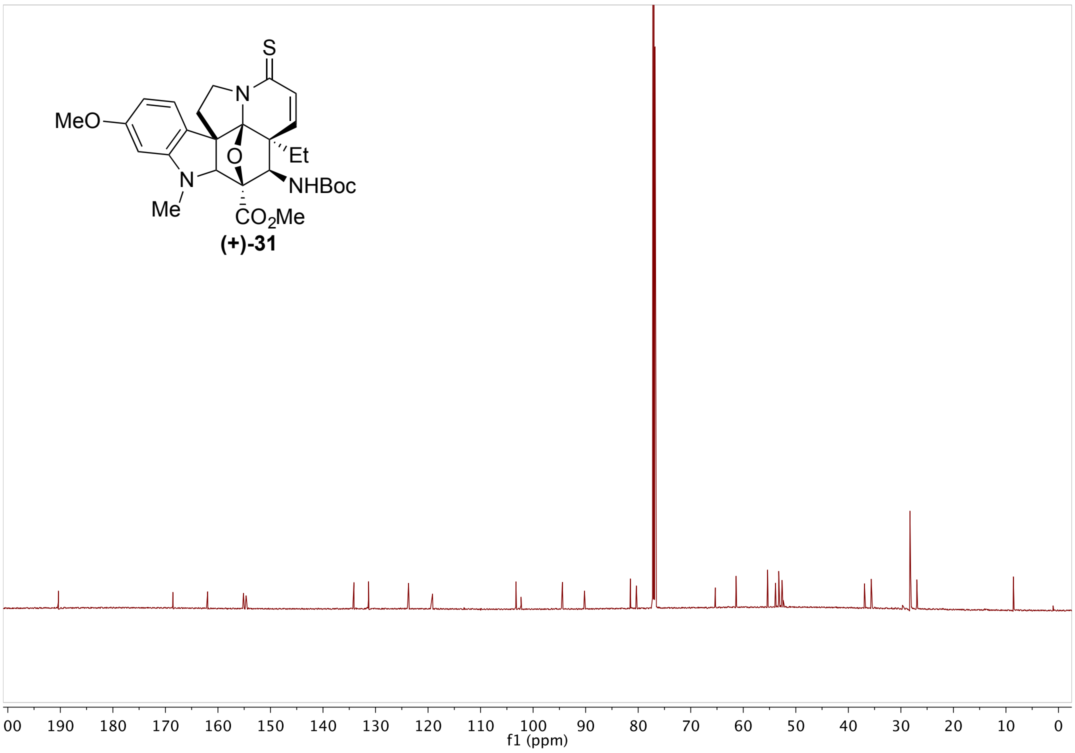

MeO
S
N
O
Et
NHBoc
N
Me
CO2Me
(+)-31
f1 (ppm)

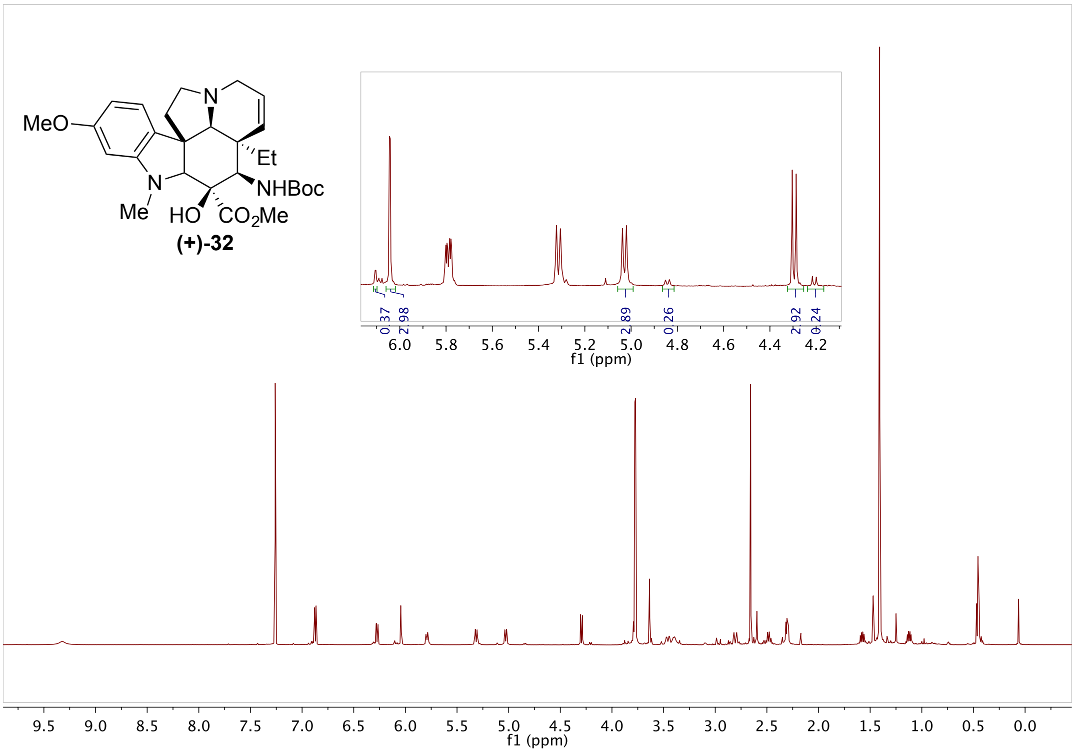
MeO
N
Et
NHBoc
N
Me
HO
CO2Me
(+)-32
0.37
2.98
2.89
0.26
2.92
0.24
6.0 5.8 5.6 5.4 5.2 5.0 4.8 4.6 4.4 4.2
f1 (ppm)
9.5 9.0 8.5 8.0 7.5 7.0 6.5 6.0 5.5 5.0 4.5 4.0 3.5 3.0 2.5 2.0 1.5 1.0 0.5 0.0
f1 (ppm)

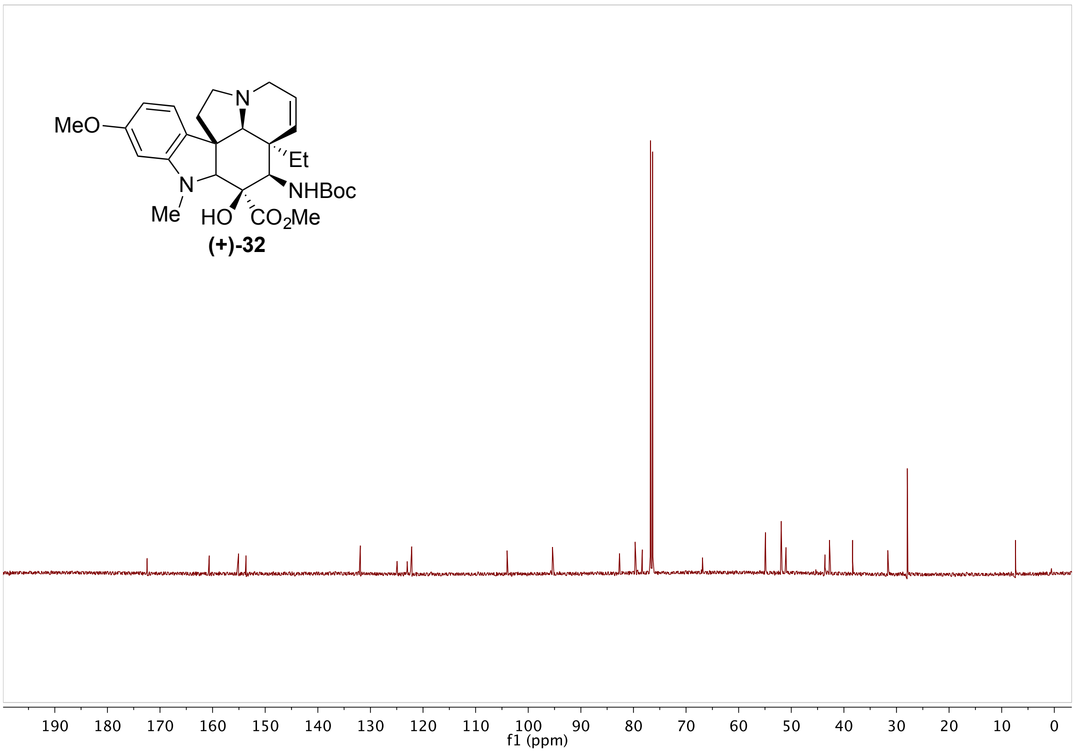
MeO
N
Et
NHBoc
N
Me
HO
CO2Me
(+)-32
190 180 170 160 150 140 130 120 110 100 90 80 70 60 50 40 30 20 10 0
f1 (ppm)

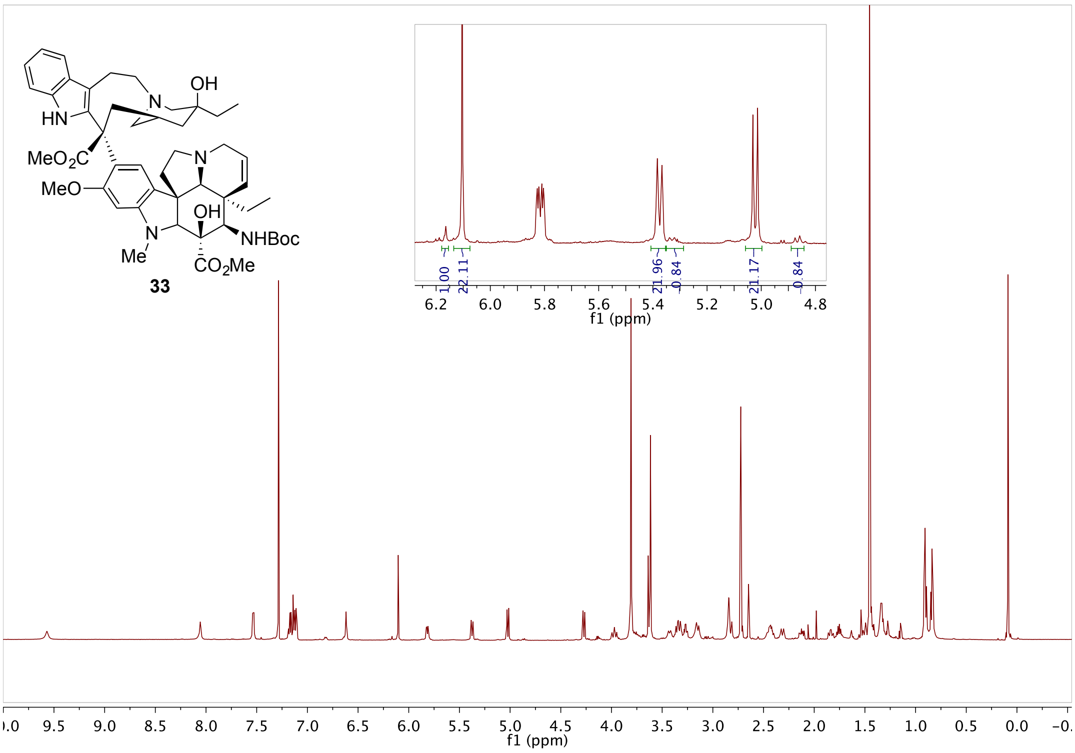
OH
N
HN
MeO2C
MeO
OH
NHBoc
N
Me
CO2Me
33
1.00
22.11
21.96
0.84
21.17
0.84
6.2 6.0 5.8 5.6 5.4 5.2 5.0 4.8
f1 (ppm)
9.5 9.0 8.5 8.0 7.5 7.0 6.5 6.0 5.5 5.0 4.5 4.0 3.5 3.0 2.5 2.0 1.5 1.0 0.5 0.0
f1 (ppm)

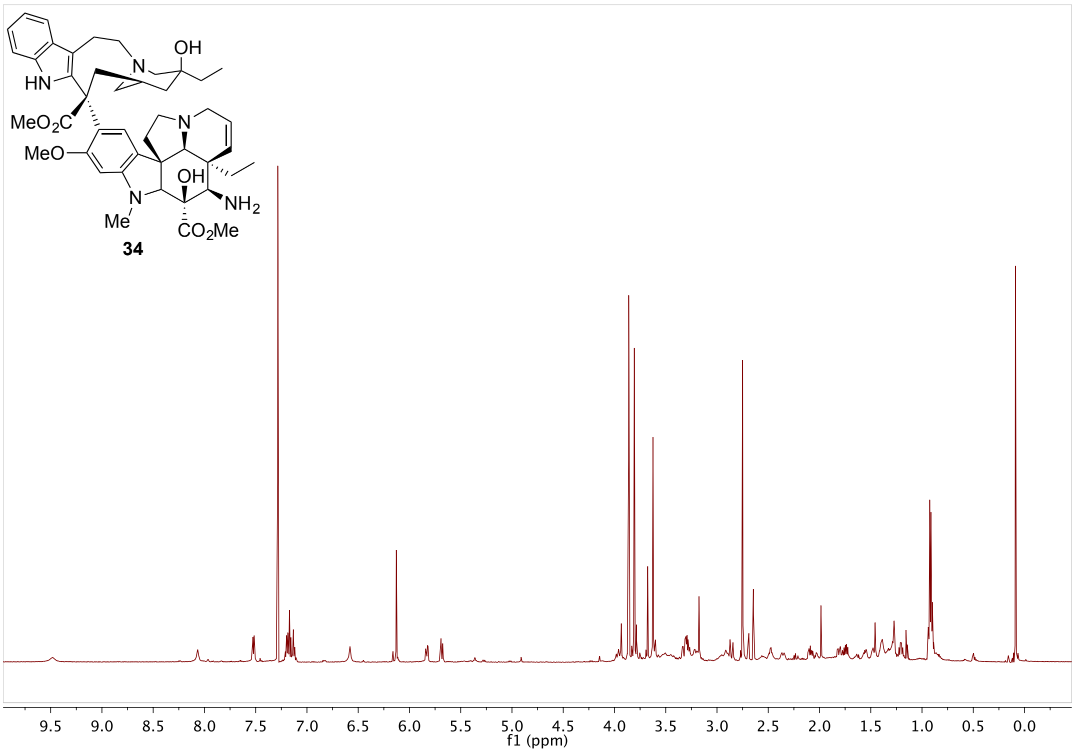
OH
N
HN
MeO2C
MeO
OH
NH2
N
Me
CO2Me
34
9.5 9.0 8.5 8.0 7.5 7.0 6.5 6.0 5.5 5.0 4.5 4.0 3.5 3.0 2.5 2.0 1.5 1.0 0.5 0.0
f1 (ppm)

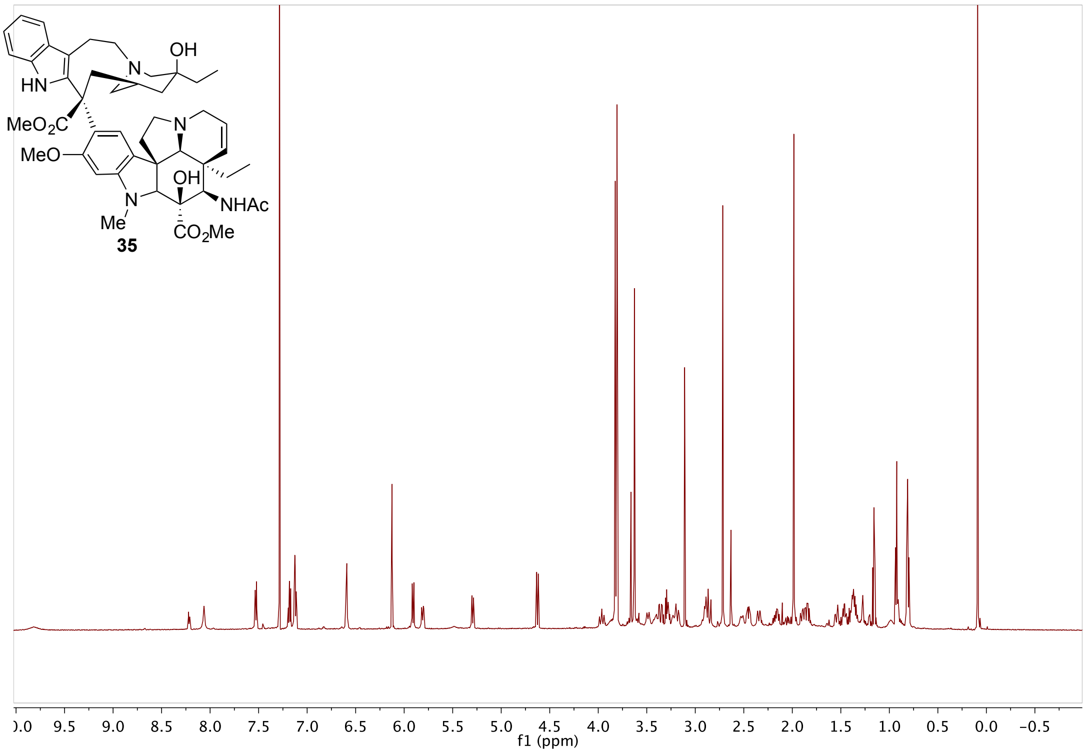
OH
HN
N
MeO2C
MeO
OH
NHAc
Me
CO2Me
35
f1 (ppm)

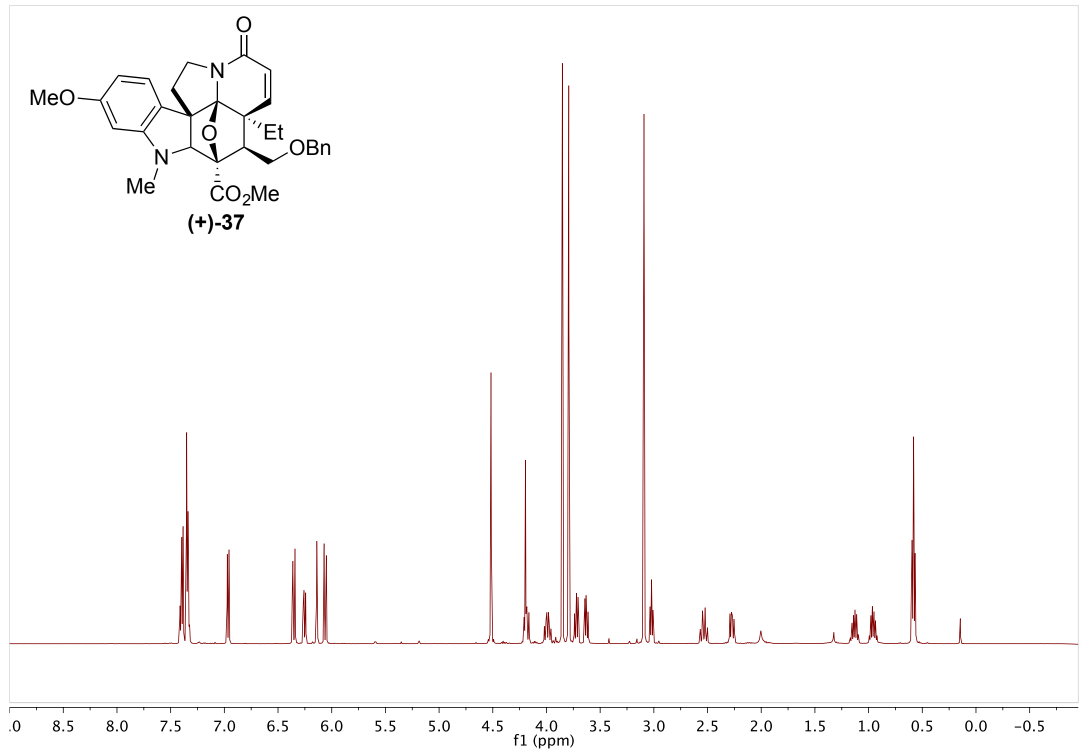
O
N
MeO
Et
O
OBn
N
Me
CO2Me
(+)-37
f1 (ppm)

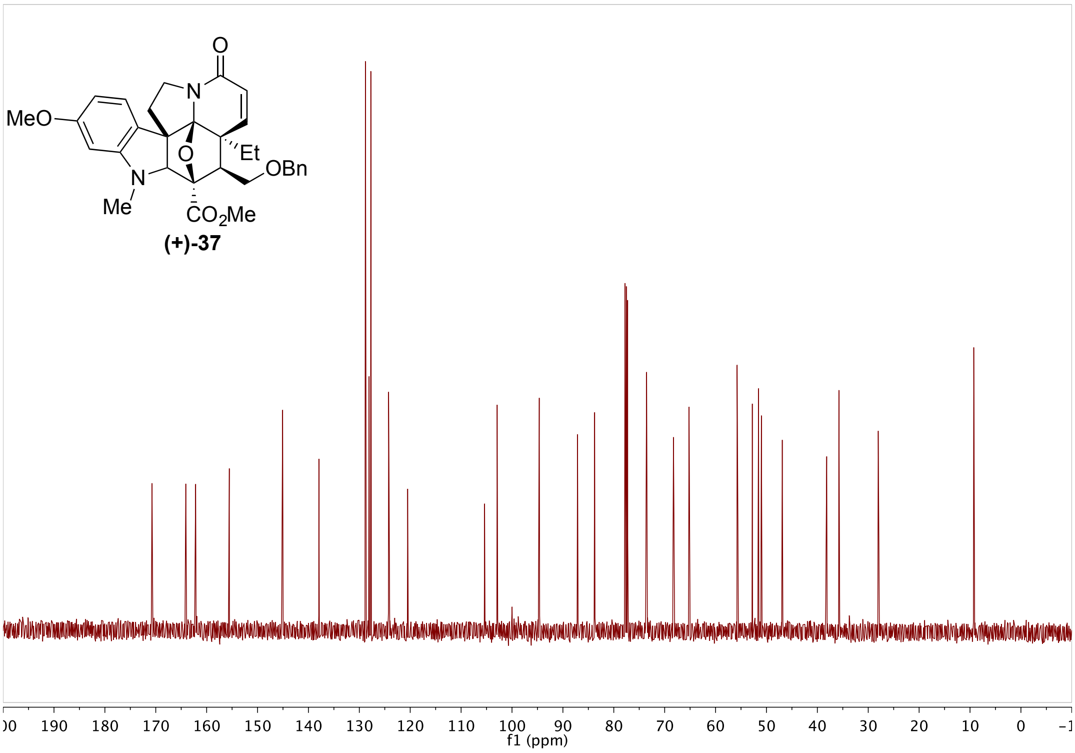

MeO
O
N
O
Et
OBn
N
Me
CO2Me
(+)-37
f1 (ppm)

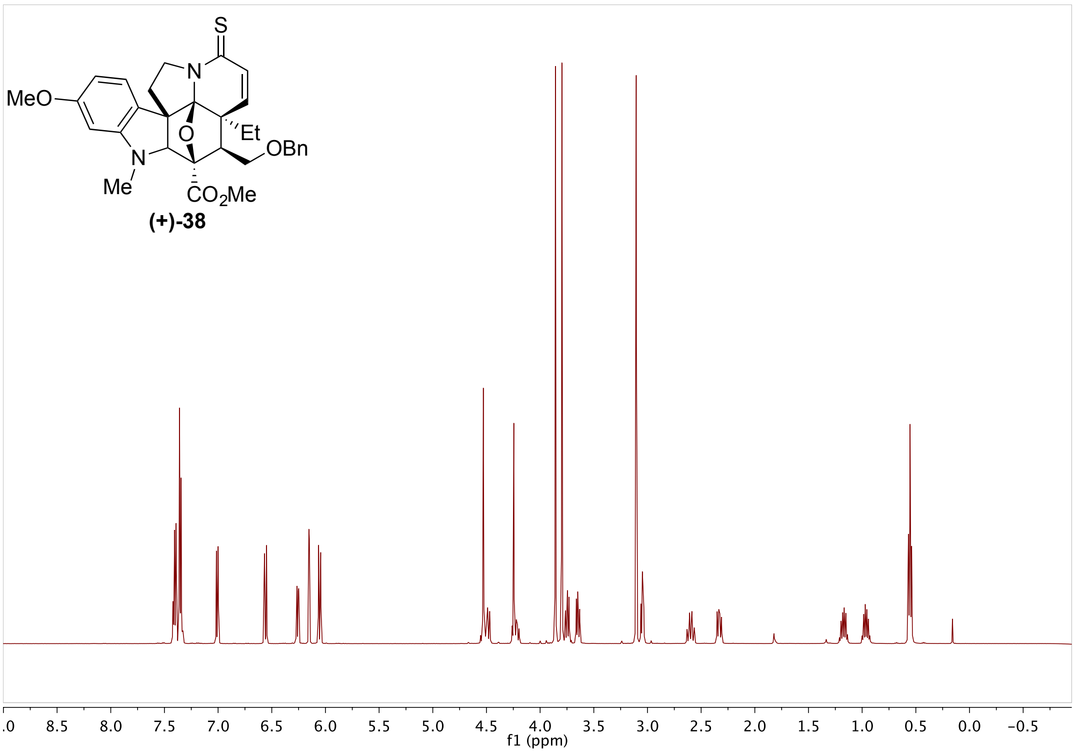

MeO
S
N
O
Et
OBn
N
Me
CO2Me
(+)-38
f1 (ppm)

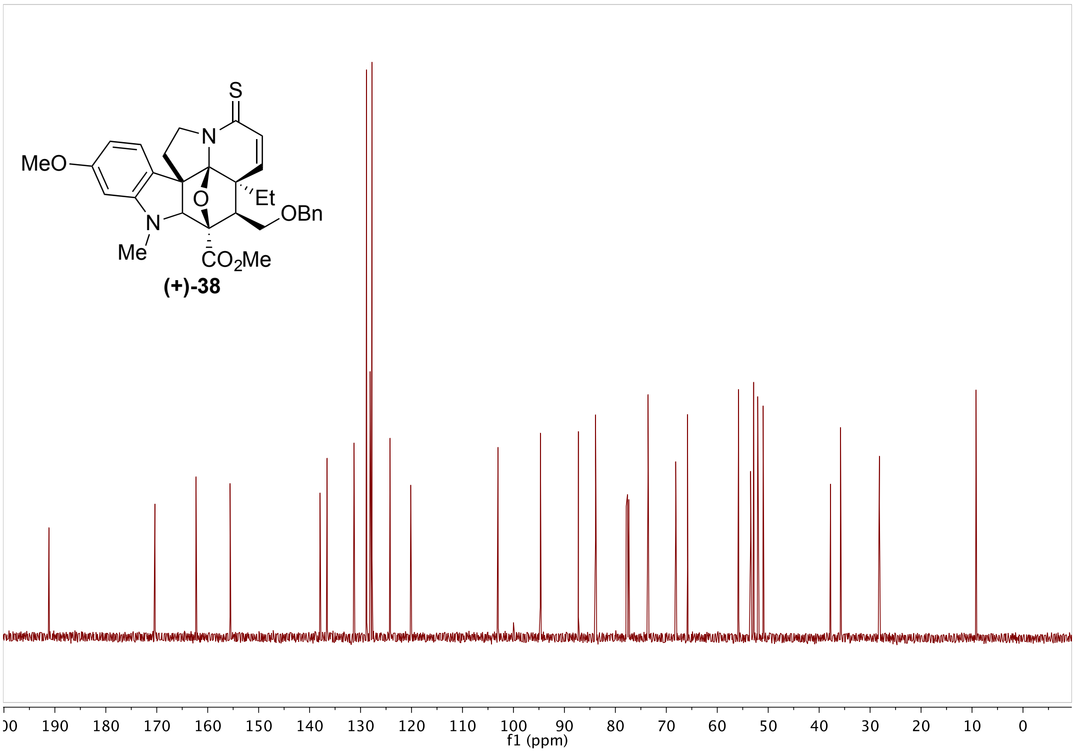
S
N
MeO
O
Et
OBn
N
Me
CO_2Me
(+)-38
190 180 170 160 150 140 130 120 110 100 90 80 70 60 50 40 30 20 10 0
f1 (ppm)

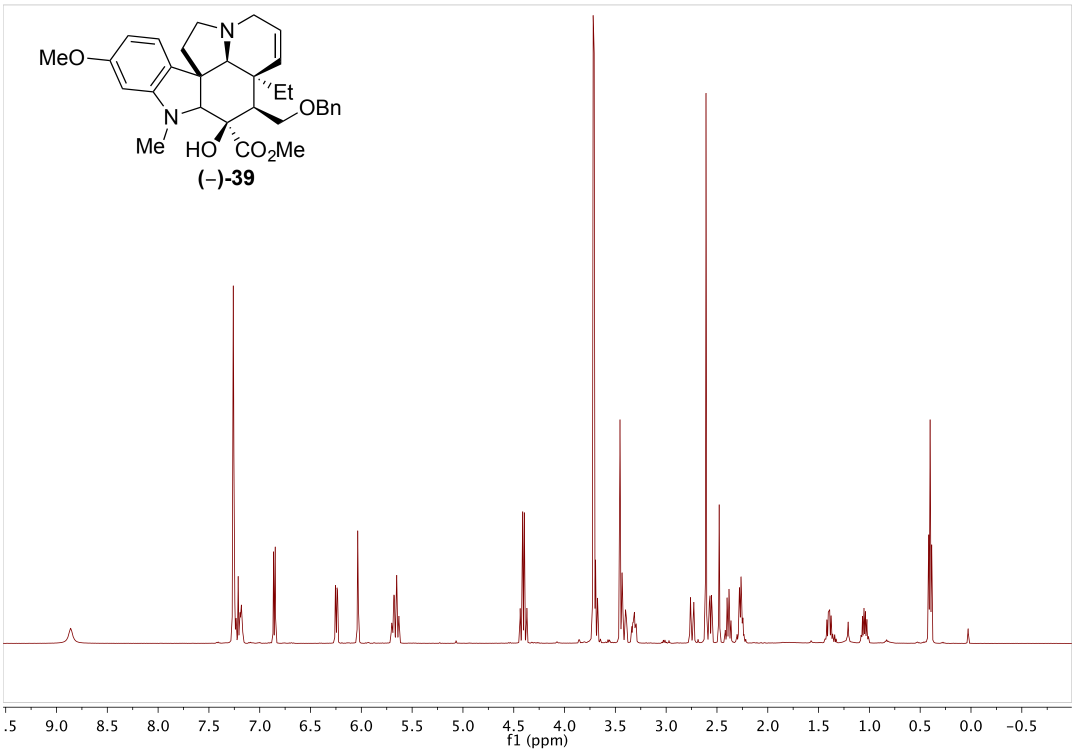
N
MeO
Et
OBn
N
Me
HO
CO_2Me
(−)-39
9.0 8.5 8.0 7.5 7.0 6.5 6.0 5.5 5.0 4.5 4.0 3.5 3.0 2.5 2.0 1.5 1.0 0.5 0.0 −0.5
f1 (ppm)

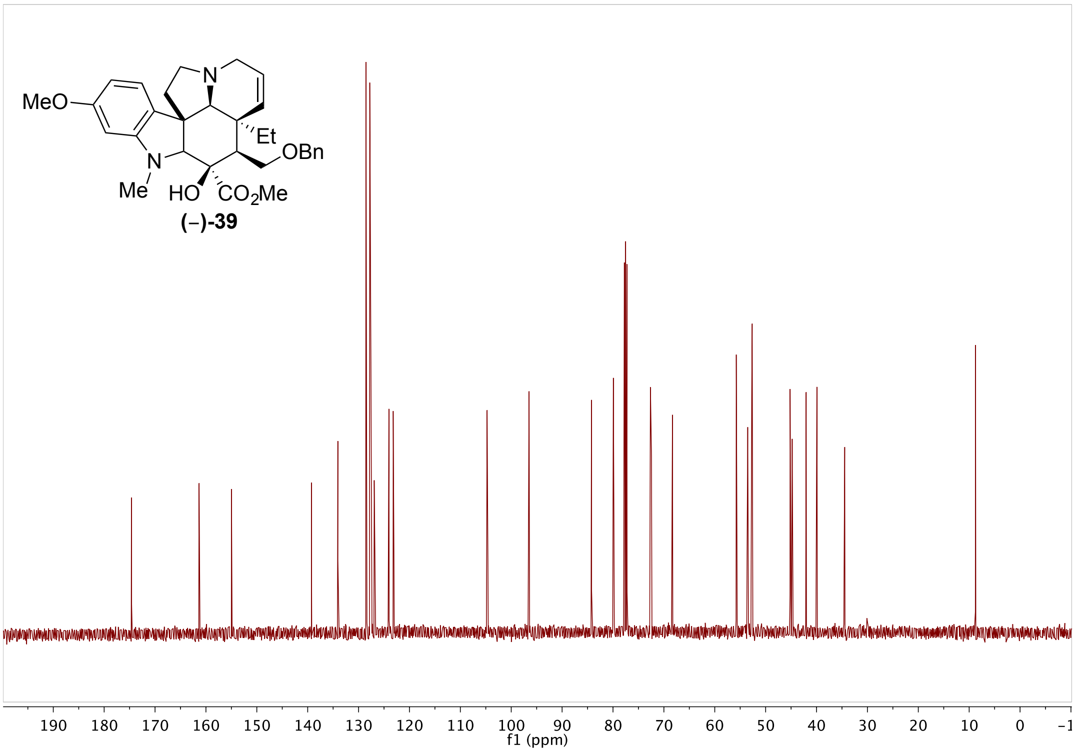
MeO
N
Me
HO
CO2Me
Et
OBn
N
(–)-39
190 180 170 160 150 140 130 120 110 100 90 80 70 60 50 40 30 20 10 0
f1 (ppm)

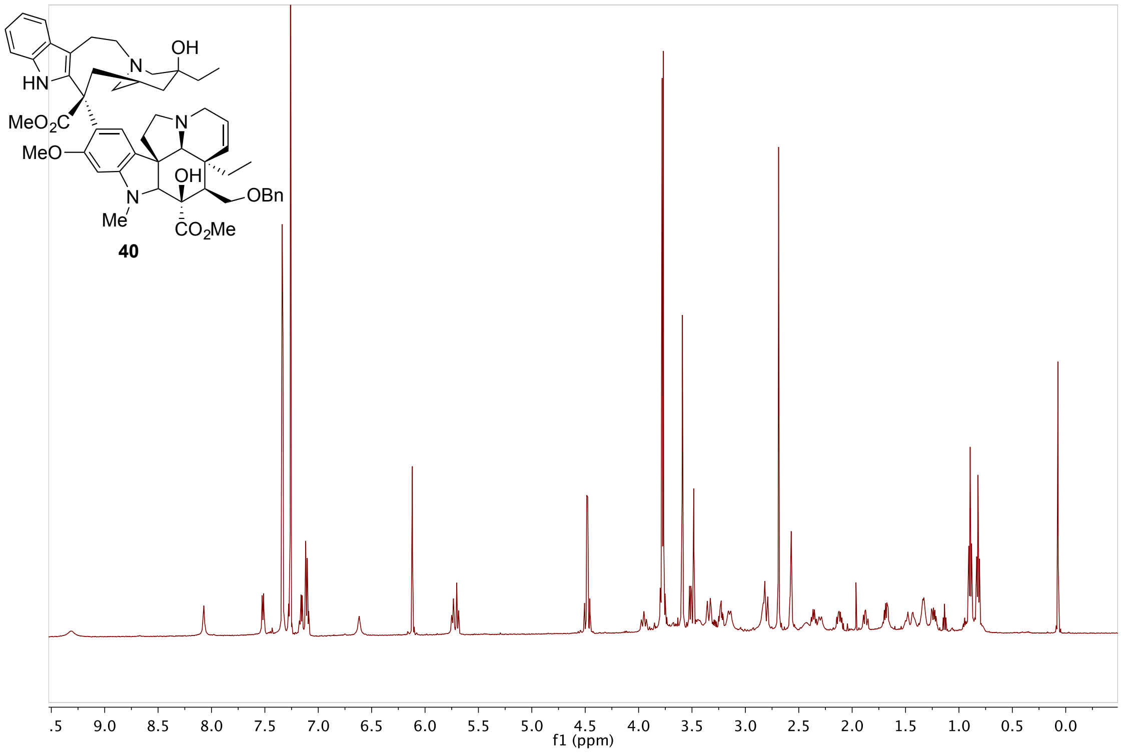
HN
OH
N
MeO2C
MeO
N
Me
OH
CO2Me
OBn
N
40
9.0 8.5 8.0 7.5 7.0 6.5 6.0 5.5 5.0 4.5 4.0 3.5 3.0 2.5 2.0 1.5 1.0 0.5 0.0
f1 (ppm)

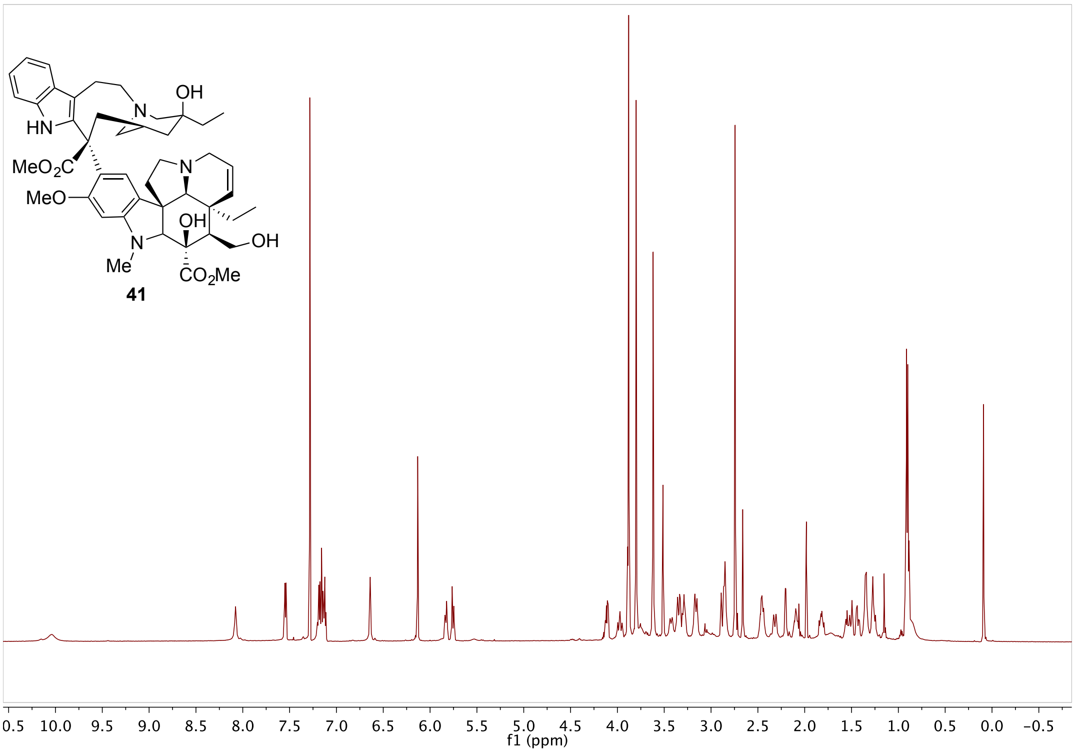
OH
HN
N
MeO2C
MeO
N
OH
OH
Me
CO2Me
41
0.5 10.0 9.5 9.0 8.5 8.0 7.5 7.0 6.5 6.0 5.5 5.0 4.5 4.0 3.5 3.0 2.5 2.0 1.5 1.0 0.5 0.0 −0.5
f1 (ppm)

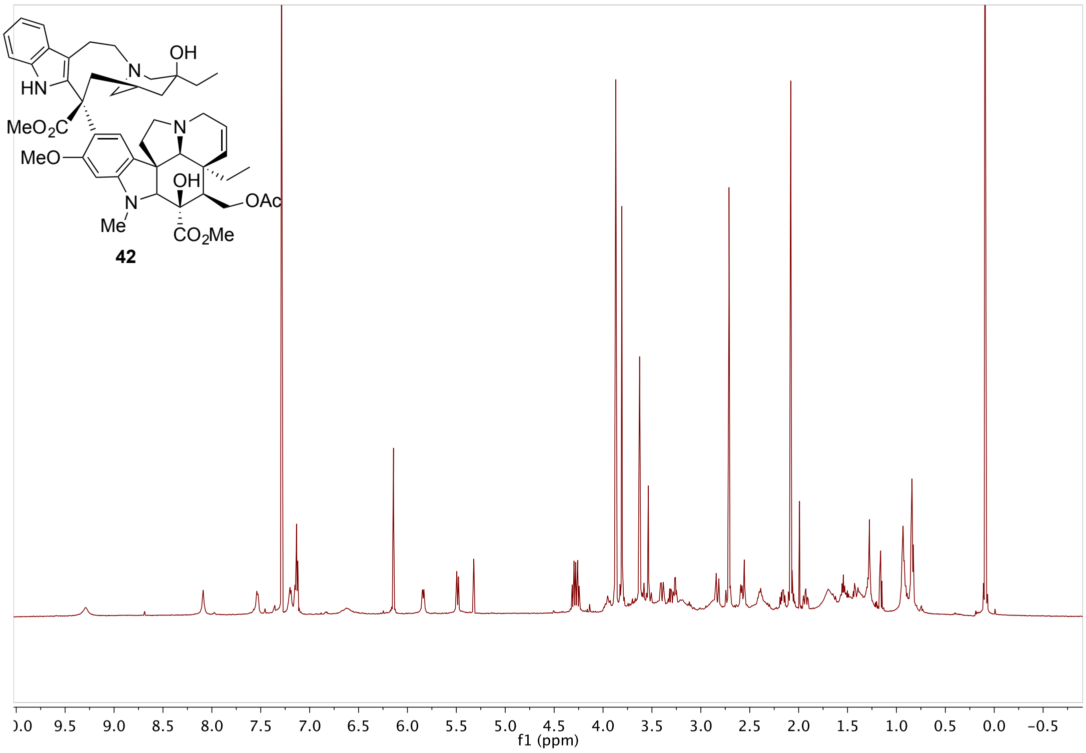
OH
HN
N
MeO2C
MeO
N
OH
OAc
Me
CO2Me
42
).0 9.5 9.0 8.5 8.0 7.5 7.0 6.5 6.0 5.5 5.0 4.5 4.0 3.5 3.0 2.5 2.0 1.5 1.0 0.5 0.0 −0.5
f1 (ppm)

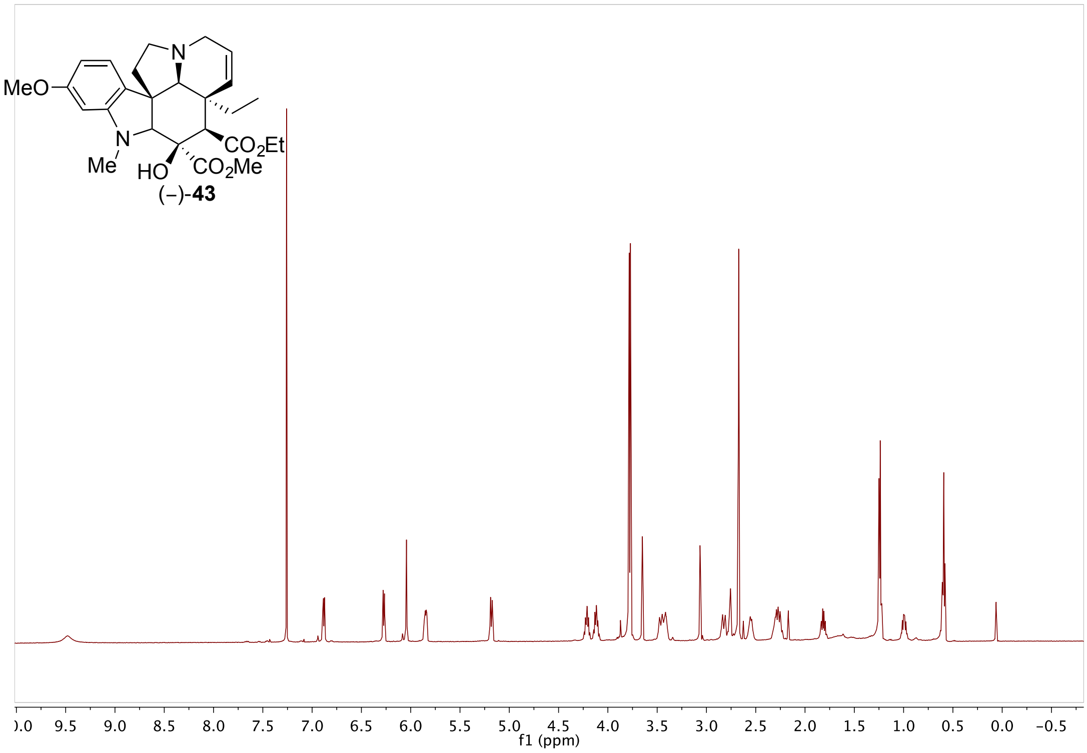
MeO
N
Me
HO
CO2Et
CO2Me
(–)-43
0.0 9.5 9.0 8.5 8.0 7.5 7.0 6.5 6.0 5.5 5.0 4.5 4.0 3.5 3.0 2.5 2.0 1.5 1.0 0.5 0.0 –0.5
f1 (ppm)

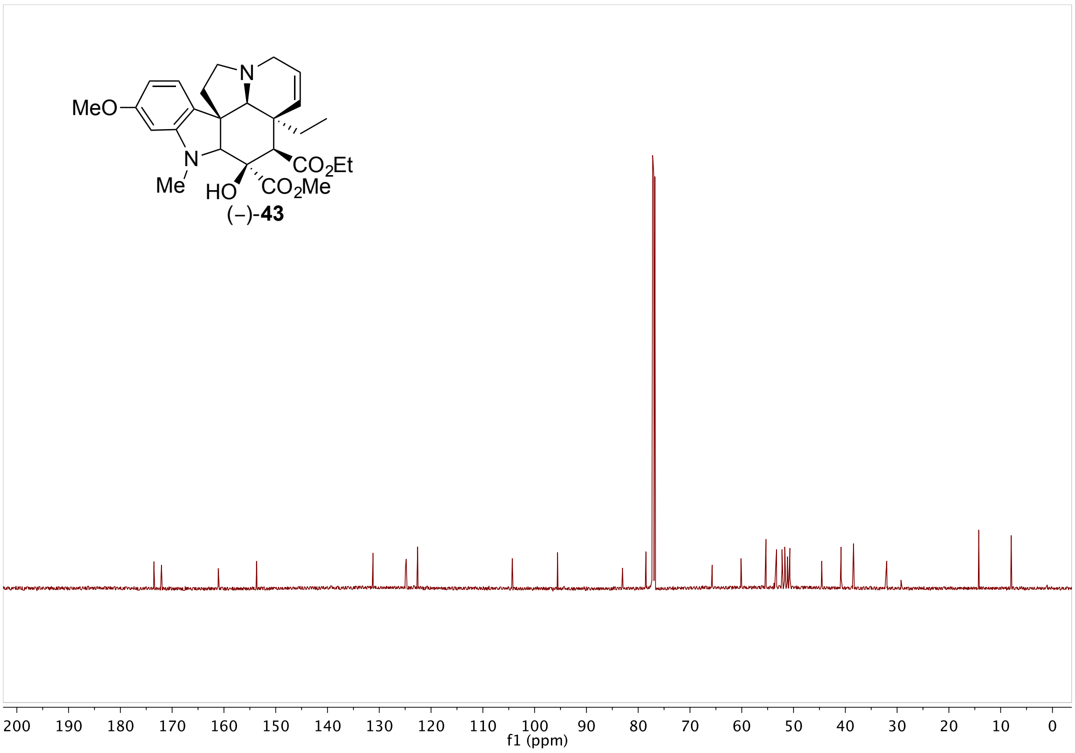
MeO
N
Me
HO
CO2Et
CO2Me
(–)-43
200 190 180 170 160 150 140 130 120 110 100 90 80 70 60 50 40 30 20 10 0
f1 (ppm)

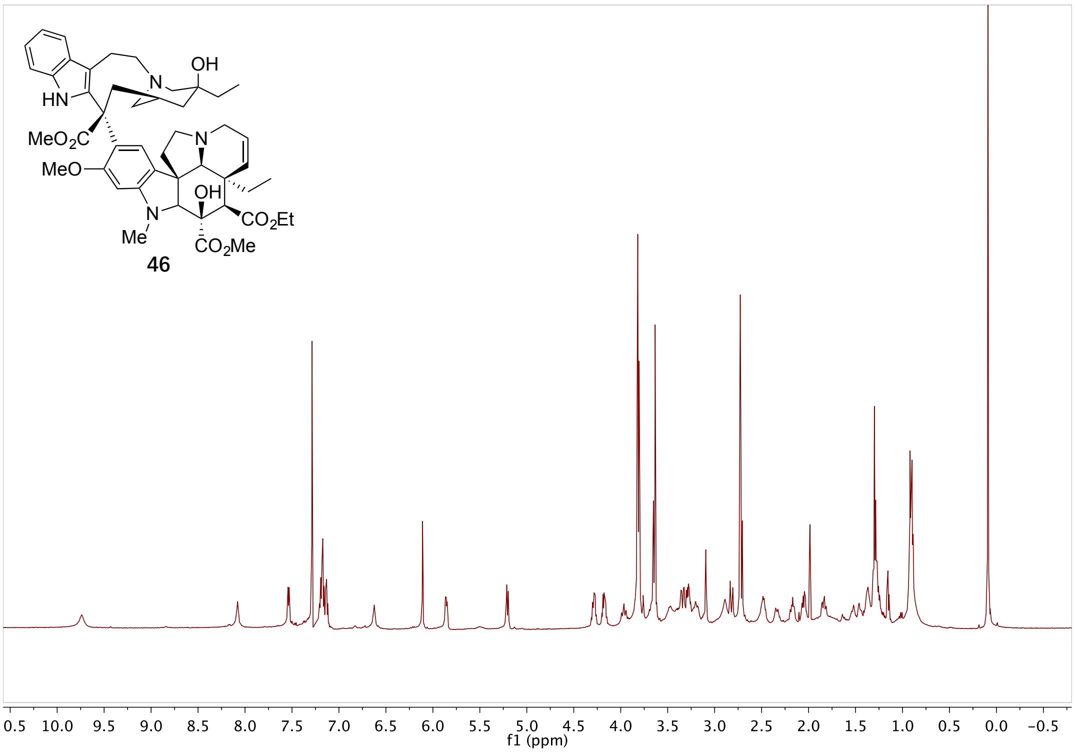
OH
N
HN
MeO2C
MeO
N
OH
N
CO2Et
Me
CO2Me
46
0.5 10.0 9.5 9.0 8.5 8.0 7.5 7.0 6.5 6.0 5.5 5.0 4.5 4.0 3.5 3.0 2.5 2.0 1.5 1.0 0.5 0.0 –0.5
f1 (ppm)

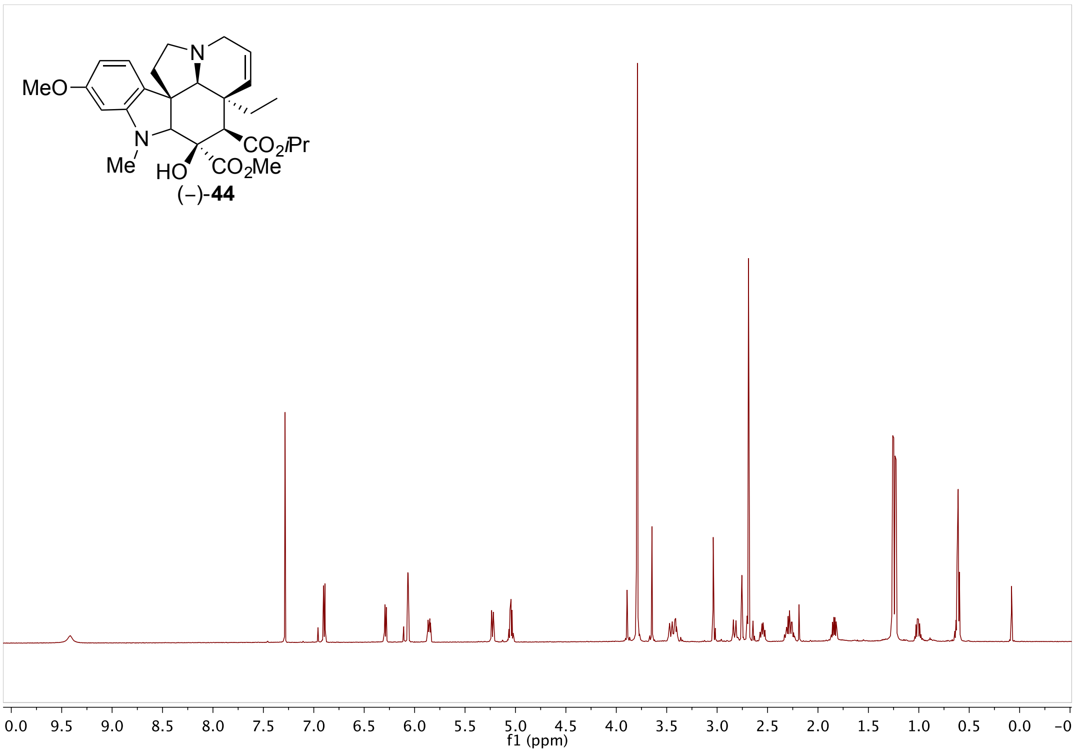
N
MeO
N
CO2iPr
Me
HO
CO2Me
(–)-44
0.0 9.5 9.0 8.5 8.0 7.5 7.0 6.5 6.0 5.5 5.0 4.5 4.0 3.5 3.0 2.5 2.0 1.5 1.0 0.5 0.0 –0
f1 (ppm)

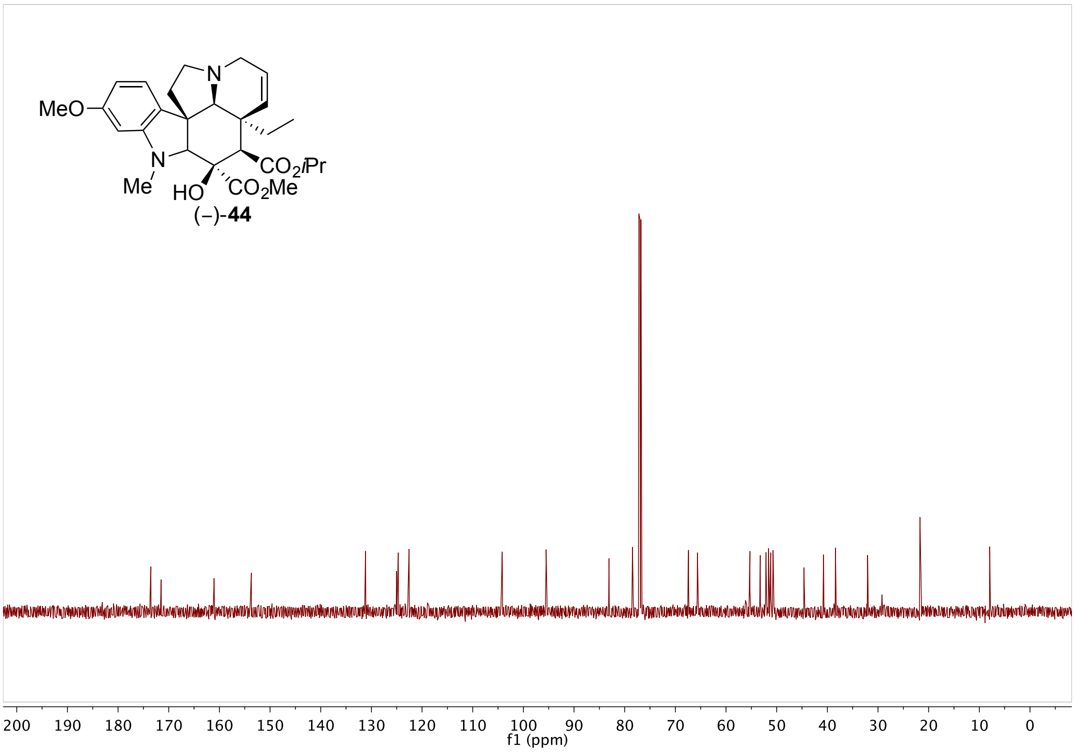
MeO
N
Me
HO
CO2iPr
CO2Me
(–)-44
200 190 180 170 160 150 140 130 120 110 100 90 80 70 60 50 40 30 20 10 0
f1 (ppm)

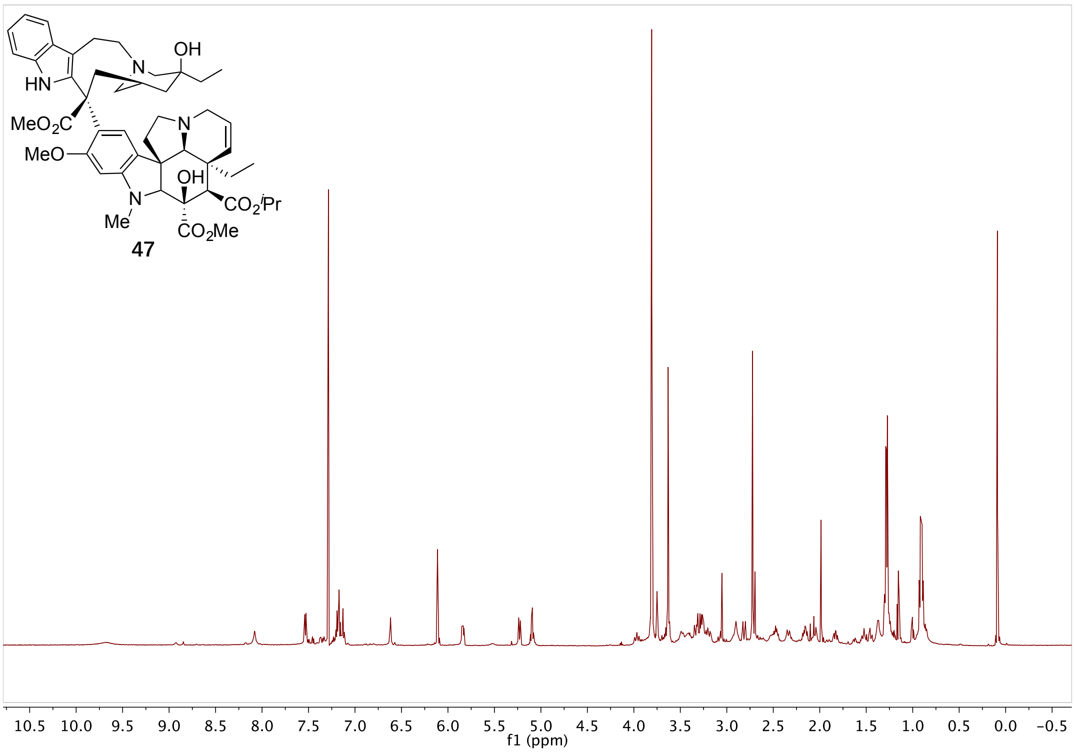
HN
N
OH
MeO2C
MeO
N
Me
OH
CO2iPr
CO2Me
47
10.5 10.0 9.5 9.0 8.5 8.0 7.5 7.0 6.5 6.0 5.5 5.0 4.5 4.0 3.5 3.0 2.5 2.0 1.5 1.0 0.5 0.0 –0.5
f1 (ppm)

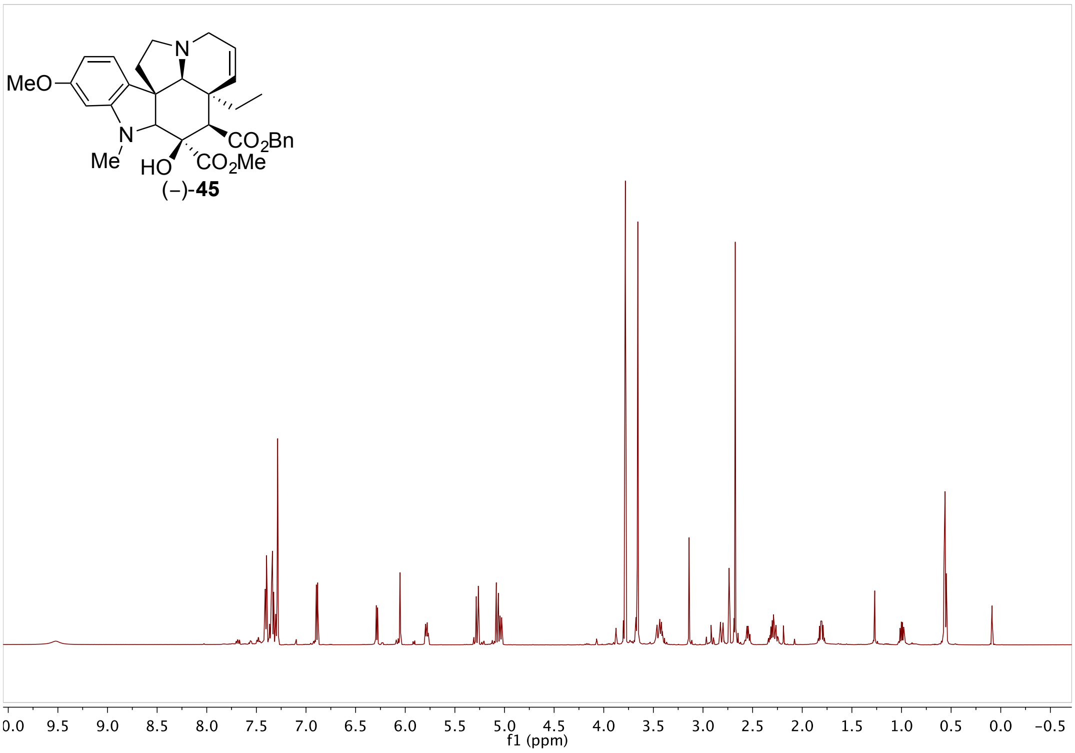
MeO
N
Me
HO
CO2Bn
CO2Me
(–)-45
f1 (ppm)

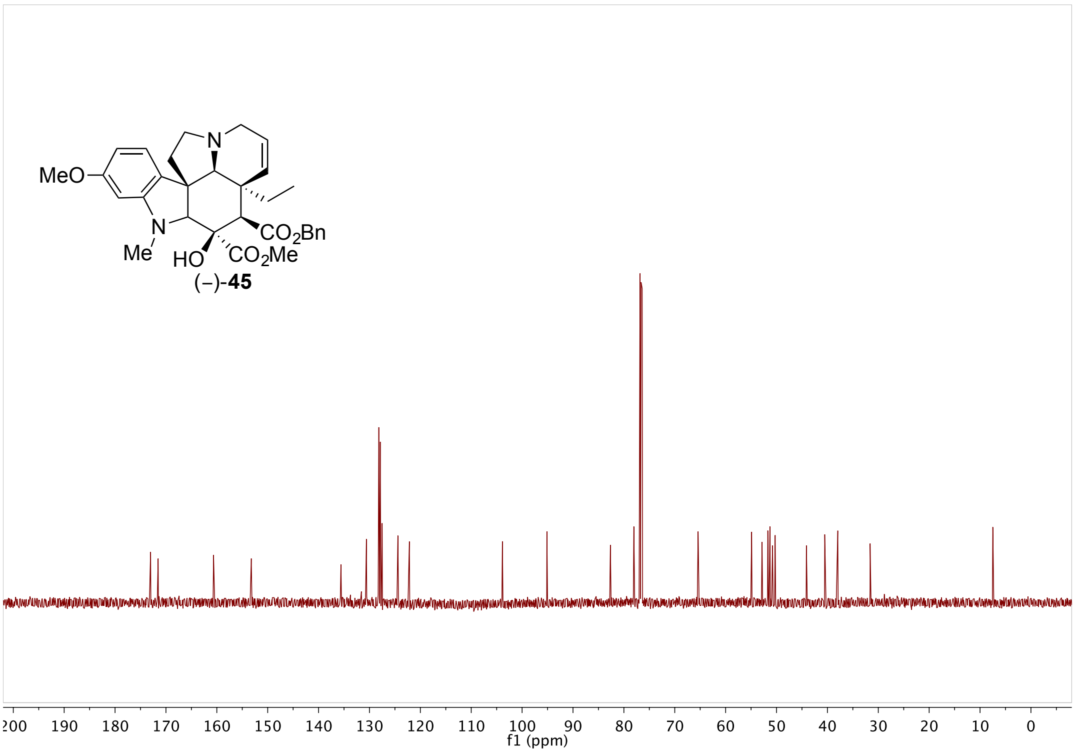
MeO
N
Me
HO
CO2Bn
CO2Me
(–)-45
f1 (ppm)

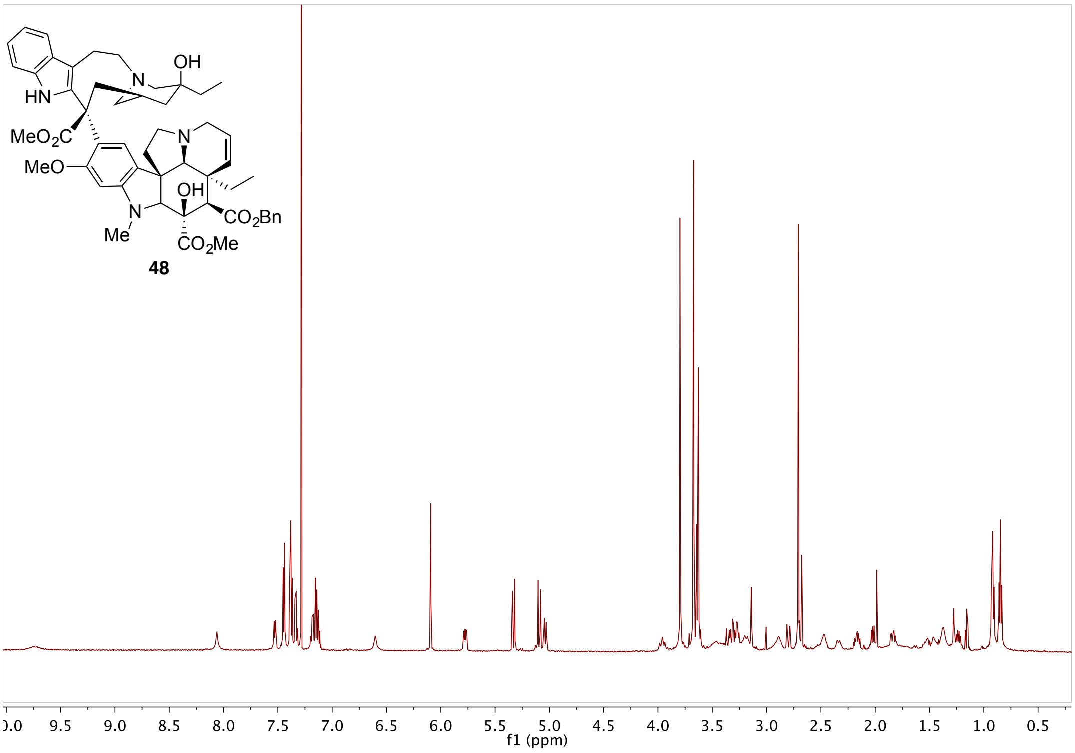
OH
HN
N
MeO2C
MeO
N
OH
CO2Bn
N
Me
CO2Me
48
f1 (ppm)

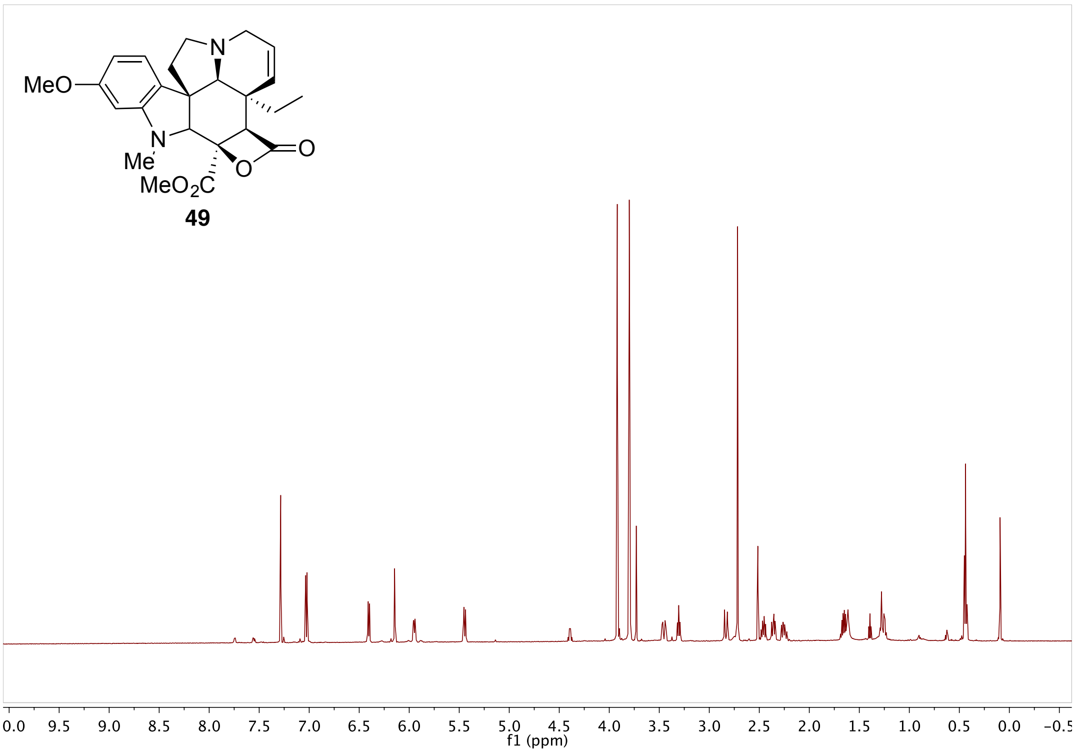
MeO
N
N
Me
O
O
MeO2C
49
f1 (ppm)

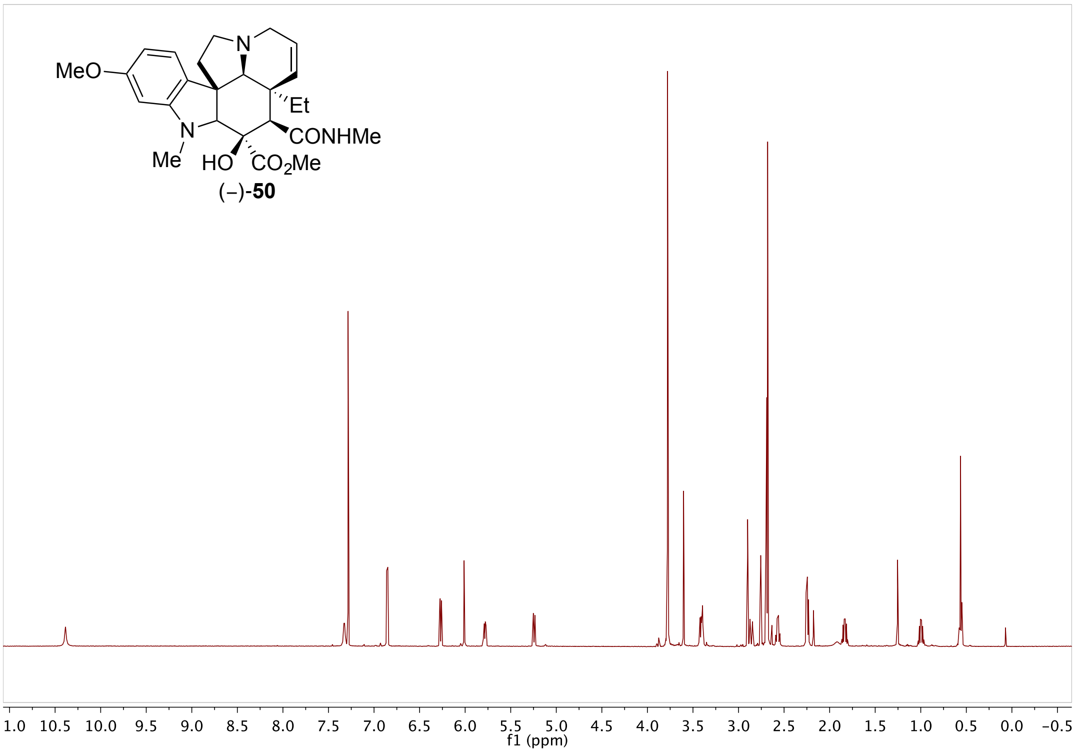

MeO
N
Et
CONHMe
Me
HO
CO2Me
(–)-50
1.0 10.5 10.0 9.5 9.0 8.5 8.0 7.5 7.0 6.5 6.0 5.5 5.0 4.5 4.0 3.5 3.0 2.5 2.0 1.5 1.0 0.5 0.0 –0.5
f1 (ppm)

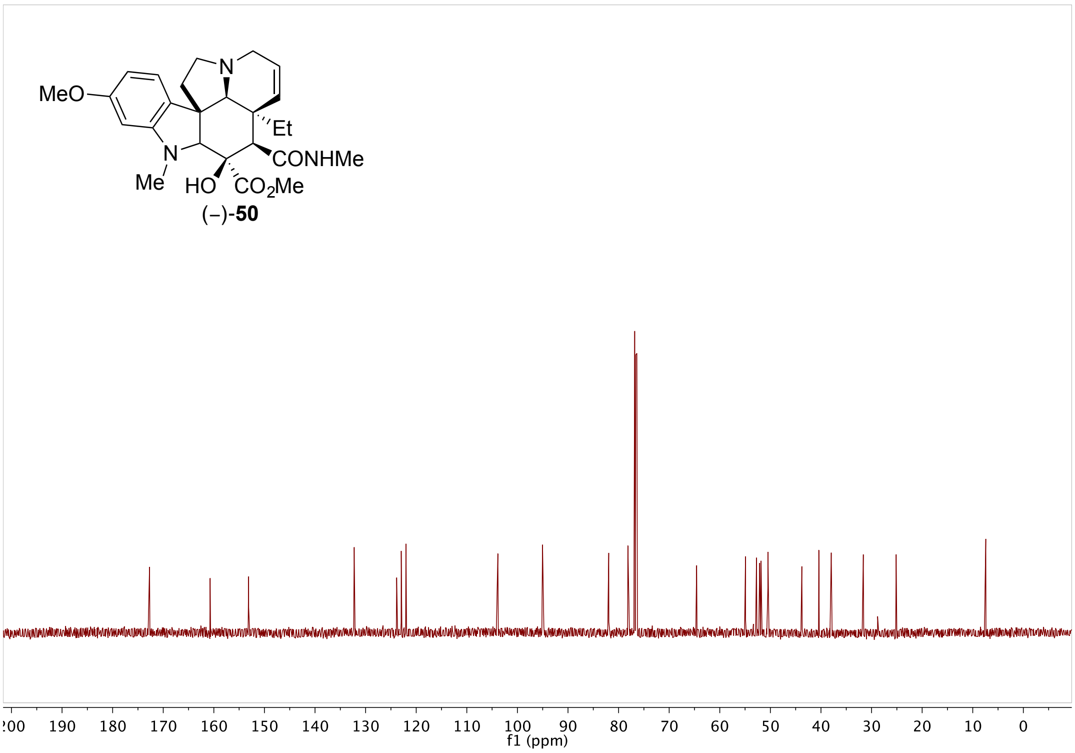

MeO
N
Et
CONHMe
Me
HO
CO2Me
(–)-50
!00 190 180 170 160 150 140 130 120 110 100 90 80 70 60 50 40 30 20 10 0
f1 (ppm)

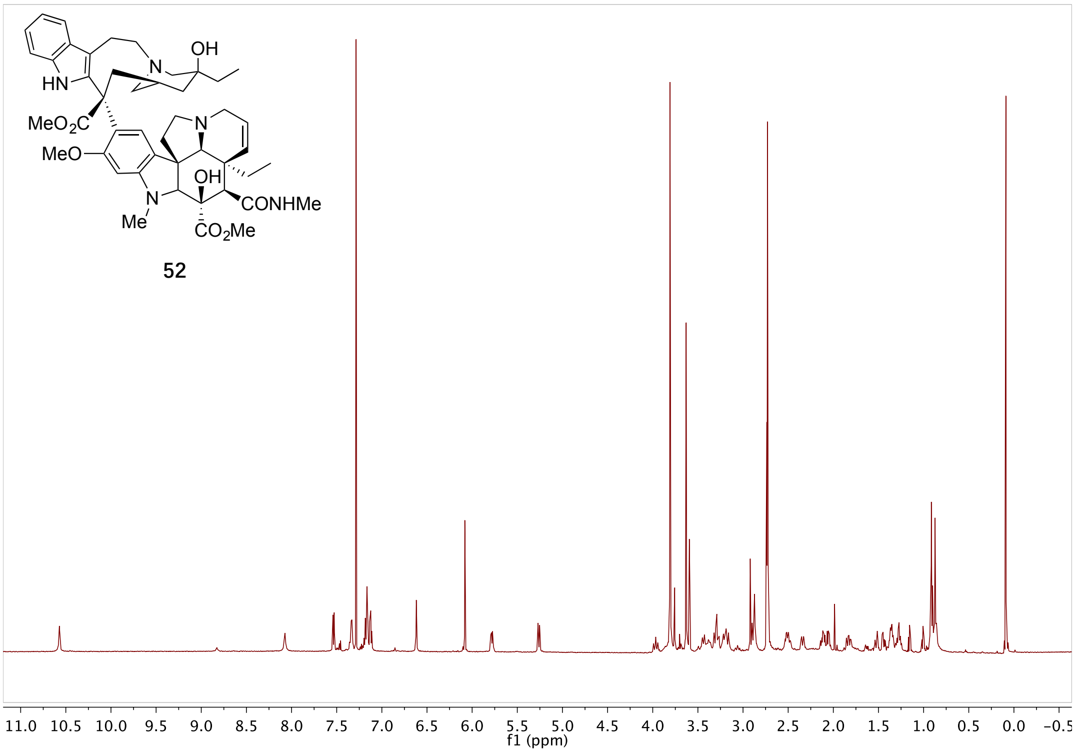

OH
HN
N
MeO2C
MeO
OH
CONHMe
Me
CO2Me
52
11.0 10.5 10.0 9.5 9.0 8.5 8.0 7.5 7.0 6.5 6.0 5.5 5.0 4.5 4.0 3.5 3.0 2.5 2.0 1.5 1.0 0.5 0.0 -0.5
f1 (ppm)

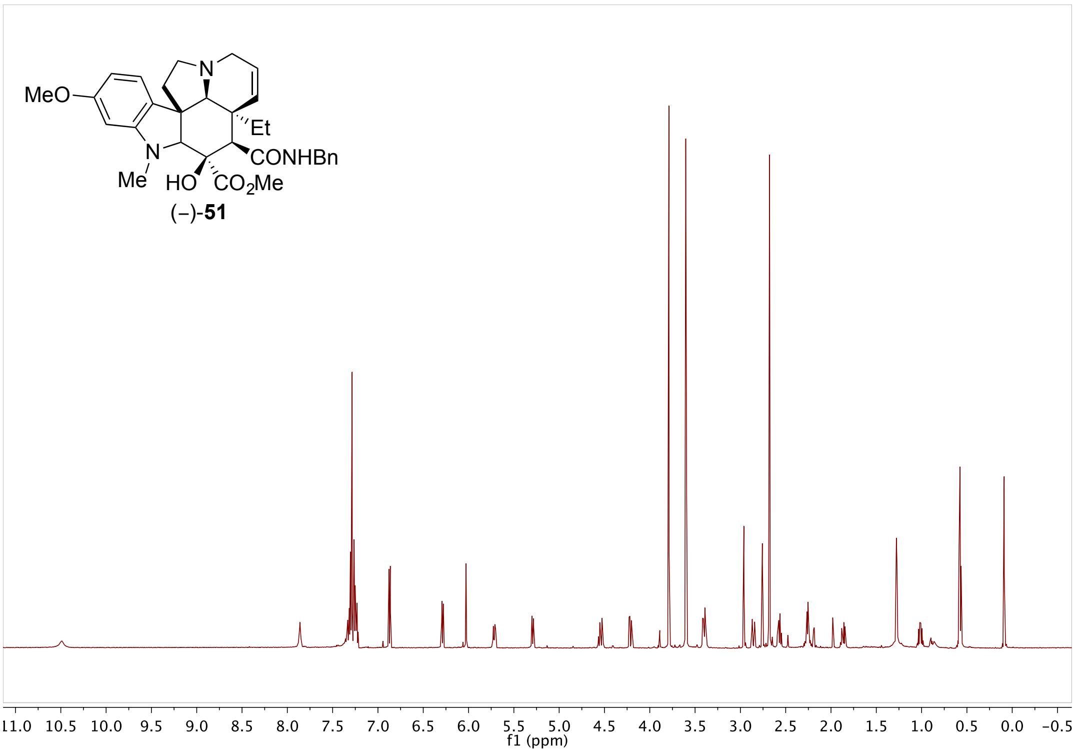

MeO
N
Et
CONHBn
Me
HO
CO2Me
(–)-51
11.0 10.5 10.0 9.5 9.0 8.5 8.0 7.5 7.0 6.5 6.0 5.5 5.0 4.5 4.0 3.5 3.0 2.5 2.0 1.5 1.0 0.5 0.0 -0.5
f1 (ppm)

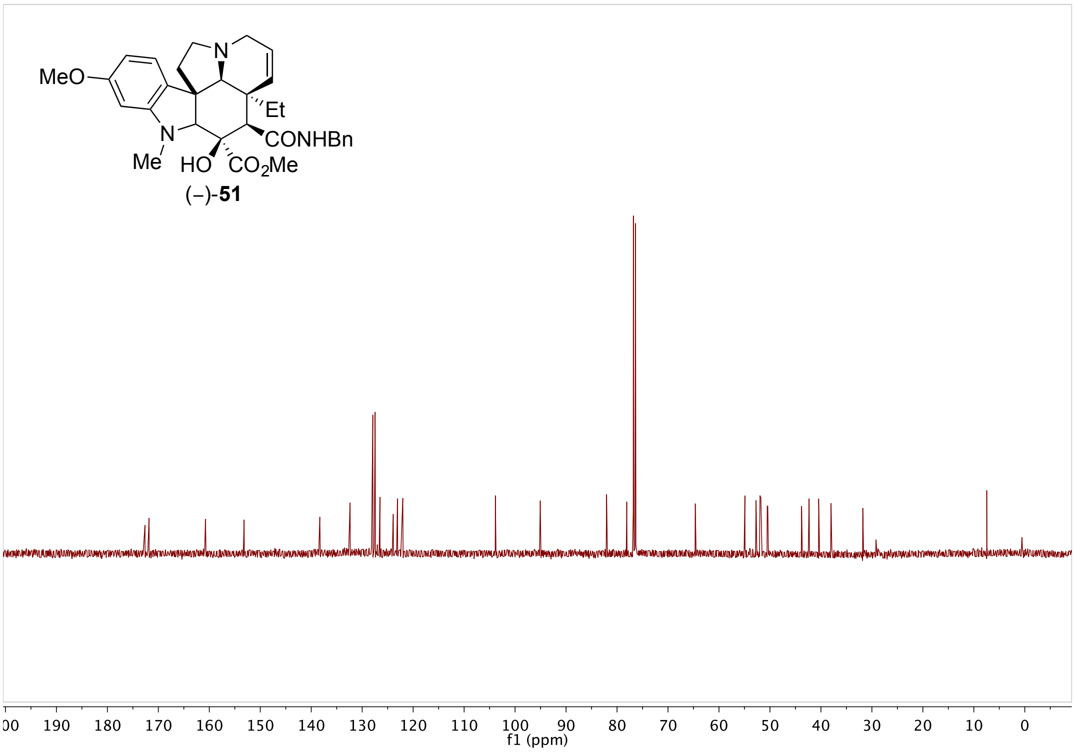
MeO
N
Et
CONHBn
Me
HO
CO2Me
(–)-51
00 190 180 170 160 150 140 130 120 110 100 90 80 70 60 50 40 30 20 10 0
f1 (ppm)

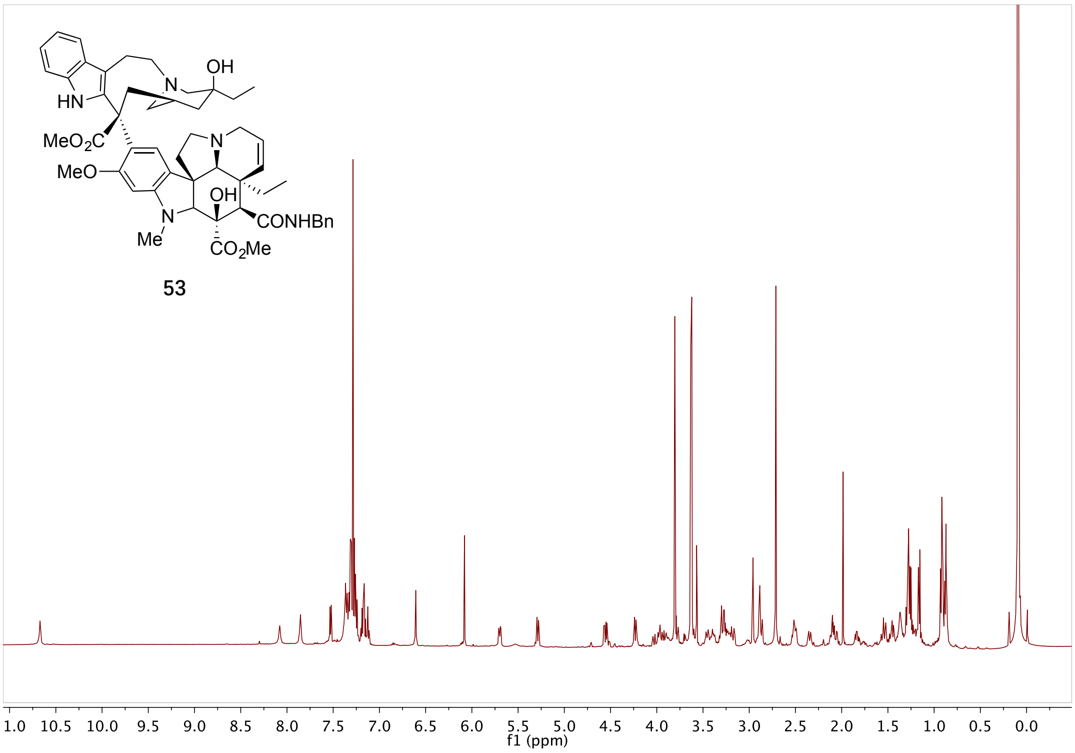
OH
N
HN
MeO2C
MeO
N
OH
CONHBn
Me
CO2Me
53
1.0 10.5 10.0 9.5 9.0 8.5 8.0 7.5 7.0 6.5 6.0 5.5 5.0 4.5 4.0 3.5 3.0 2.5 2.0 1.5 1.0 0.5 0.0
f1 (ppm)
